# Supplementary material for: Persistence in risk and effect of COVID-19 vaccination on long-term health consequences after SARS-CoV-2 infection
Source: Nat Commun. 2024 Feb 26;15:1716. doi: 10.1038/s41467-024-45953-1 (PMC10894867; doi:10.1038/s41467-024-45953-1)
Supplement: Supplementary file 1 — Supplementary Information [file 41467_2024_45953_MOESM1_ESM.pdf]

Supplementary Material

Persistence in risk and effect of COVID-19 vaccination on long-term health consequences after SARS-CoV-2 infection

Supplementary Table 1. Full disease diagnosis codes for health outcomes following SARS-CoV-2 infection and pre-existing morbidities 3

Supplementary Table 2. Baseline characteristics of patients with COVID-19 identified by a positive laboratory confirmed polymerase chain reaction COVID-19 screening test result stratified by vaccine status and controls from the Hong Kong Hospital Authority before propensity-score weighting ... 5

Supplementary Table 3. Incidence rate and hazard ratio of clinical sequelae in COVID-19 and non-COVID-19 cohort after weighting during 0-30, 31-90, 91-180, 181-270, 271-365 days of SARS-CoV-2 infection (Patients with a positive laboratory confirmed polymerase chain reaction COVID-19 screening test result) 7

Supplementary Table 4. Baseline characteristics of patients with COVID-19 from the Omicron wave in Hong Kong stratified by vaccine status and controls from the Hong Kong Hospital Authority before propensity-score weighting. 12

Supplementary Table 5. Incidence rate and hazard ratio of clinical sequelae in COVID-19 and non-COVID-19 cohort after weighting during 0-30, 31-90, 91-180, 181-270,271-365 days of SARS-CoV-2 infection (Cases of SARS-COV-2 infection from the Omicron wave in Hong Kong) 14

Supplementary Table 6. Baseline characteristics of unvaccinated patients with COVID-19 and matched control with the same vaccination status from the Hong Kong Hospital Authority before propensity-score weighting. 19

Supplementary Table 7. Incidence rate and hazard ratio of clinical sequelae in COVID-19 and non-COVID-19 cohort after weighting during 0-30, 31-90, 91-180, 181-270,271-365 days of SARS-CoV-2 infection (unvaccinated patients with COVID-19 and matched control with the same vaccination status) 21

Supplementary Table 8. Baseline characteristics of patients with SARS-CoV-2 infection less than 6 months after the date of last dose of vaccination stratified by vaccine status and controls from the Hong Kong Hospital Authority before propensity-score weighting. 24

Supplementary Table 9. Incidence rate and hazard ratio of clinical sequelae in COVID-19 and non-COVID-19 cohort after weighting during 0-30, 31-90, 91-180, 181-270,271-365 days of SARS-CoV-2 infection (Excluding patients with incident SARS-CoV-2 infection over 6 months after the last dose of COVID-19 vaccination) 26

Supplementary Table 10. Incidence rate and hazard ratio of clinical sequelae in COVID-19 and non-COVID-19 cohort after weighting during 0-30, 31-90, 91-180, 181-270,271-365 days of SARS-CoV-2 infection (Adjusting for the likely variant of SARS-COV-2 responsible for the infection) 31

Supplementary Table 11. P-value, rank and critical values of clinical sequelae estimated by the Benjamini–Hochberg procedure 36

Supplementary Table 12. Incidence rate and hazard ratio of lung cancer, brain cancer and lymphoma in COVID-19 and non-COVID-19 cohort after weighting during 0-30, 31-90, 91-180, 181-270, 271-365 days of SARS-CoV-2 infection 41

Supplementary Table 13. Incidence rate and hazard ratio of clinical sequelae in COVID-19 and non-COVID-19 cohort after weighting during 0-30, 31-90, 91-180, 181-270, 271-365 days of SARS-CoV-2 infection (Patients aged over 65) 42

Supplementary Table 14. Incidence rate and hazard ratio of clinical sequelae in COVID-19 and non-COVID-19 cohort after weighting during 0-30, 31-90, 91-180, 181-270, 271-365 days of SARS-CoV-2 infection (Patients aged 65 or under) 47

Supplementary Table 15. Incidence rate and hazard ratio of clinical sequelae in COVID-19 and non-COVID-19 cohort after weighting during 0-30, 31-90, 91-180, 181-270, 271-365 days of SARS-CoV-2 infection (Patients with Charlson Co-morbidity Index <4) 52

Supplementary Table 16. Incidence rate and hazard ratio of clinical sequelae in COVID-19 and non-COVID-19 cohort after weighting during 0-30, 31-90, 91-180, 181-270, 271-365 days of SARS-CoV-2 infection (Patients with Charlson Co-morbidity Index >=4) 57

Supplementary Table 17. Incidence rate and hazard ratio of clinical sequelae in COVID-19 and non-COVID-19 cohort after weighting during 0-30, 31-90, 91-180, 181-270, 271-365 days of SARS-CoV-2 infection (Male patients) 62

Supplementary Table 18. Incidence rate and hazard ratio of clinical sequelae in COVID-19 and non-COVID-19 cohort after weighting during 0-30, 31-90, 91-180, 181-270, 271-365 days of SARS-CoV-2 infection (Female patients) 67

Supplementary Figure 1. Cumulative incidence plot for clinical sequelae during 0-30, 31-90, 91-180, 181-270, 271-365 days of SARS-CoV-2 infection 72

Supplementary Table 1. Full disease diagnosis codes for health outcomes following SARS-CoV-2 infection and pre-existing morbidities

Disease	ICD-9-CM
**Health outcomes following SARS-CoV-2**	
Myocardial infarction	410
Heart failure	428,398·91,402·01,402·11,402·91,404·01,404·03, 404·11,404·13,404·91,404·93
Stroke	430-438
Atrial fibrillation	427·3
Coronary artery disease	410-414, 36·0, 36·1
Deep vein thrombosis	453
Acute respiratory distress syndrome	518·8x, 518·51-518·53
Chronic pulmonary disease	490-496
Seizure	333·2, 345, 649·4, 780·3, 779·0, 780·3
Pancreatitis	577·0, 577·1
Acute kidney injury	584·5-584·9
End-stage renal disease	585·6, 586
Lung cancer	162-164, 197
Brain cancer	191
Lymphoma	200-209
**Pre-existing morbidities**	
Diabetes	250
Cardiovascular disease	398·91, 401-405, 410-412, 425·4, 425·5, 425·7-425·9, 427·3, 428, 433, 434, 436-438, 453·8
Dementia	290
Congestive heart failure	398·91, 402·01, 402·11, 402·91, 404·01, 404·03, 404·11, 404·13, 404·91, 404·93, 428
Peripheral vascular disease	441, 443·9, 785·4
Cerebrovascular disease	430-438
Rheumatoid arthritis and other inflammatory polyarthropathies	710·0, 710·1, 710·4, 714·0, 714·1, 714·2, 714·81, 725
Chronic pulmonary disease	490-496, 500-505, 506·4
Hemiplegia or paraplegia	342, 344·1
Chronic renal failure	582, 585, 586, 588, 583·0-583·2, 583·4, 583·6, 583·7

Supplementary Table 1. Full disease diagnosis codes for health outcomes following SARS-CoV-2 infection and pre-existing morbidities (Continued)

Disease	ICD-9-CM
Liver disease, mild	571·2, 571·4-571·6
Diabetes without chronic complications	250·0-250·3, 250·7
Diabetes with chronic complications	250·4-250·6
Ulcers	531-534
Metastatic solid tumour	196-199
Malignancy	140-149, 150-159, 180-189, 170-172, 174, 175, 176, 179, 160-165, 190-195, 200-208
Liver disease, moderate to severe	456·0-456·2, 572·2-572·4· 572·8
Cancer	140-149, 150-159, 180-189,170-172, 174-176, 179, 160-165, 190-195, 200-208
Chronic Kidney Disease	582, 585, 586, 588, 583·0-583·2, 583·4, 583·6, 583·7
Respiratory disease	416·8, 416·9, 490-496, 500-505, 506·4, 508·1, 508·8

Note: ICD-9-CM: International Classification of Diseases, Ninth Revision, clinical modification

Supplementary Table 2. Baseline characteristics of patients with COVID-19 identified by a positive laboratory confirmed polymerase chain reaction COVID-19 screening test result stratified by vaccine status and controls from the Hong Kong Hospital Authority before propensity-score weighting

	Hong Kong Hospital Authority (April 1st, 2020 to October 31st, 2022)					
		COVID-19				
**Baseline characteristics**	**Controls** (N=1,599,711)	**Un-vaccinated** (N=89,246)	**Incomplete vaccination** (N=64,208)	**Fully vaccinated** (N= 303,213)	**Receieved booster dose** (N= 248,478)	**SMD[b]**
	**N / Mean (% / SD)**	**N / Mean (% / SD)**	**N / Mean (% / SD)**	**N / Mean (% / SD)**	**N / Mean (% / SD)**	
Age (years)	56.2 (18.03)	61.9 (21.78)	61.0 (19.57)	50.9 (17.49)	54.4 (15.66)	0.306
Sex, male	737,158 (46.1)	41,886 (46.9)	30,549 (47.6)	140,764 (46.4)	120,899 (48.7)	0.025
Charlson Comorbidity Index	1.80 (1.83)	2.83 (2.60)	2.55 (2.25)	1.39 (1.72)	1.53 (1.64)	0.375
**Pre-existing morbidities**						
Respiratory disease	31,209 (2.0)	4,807 (5.4)	2,701 (4.2)	5,997 (2.0)	4,689 (1.9)	0.102
Diabetes	196,566 (12.3)	16,591 (18.6)	12,676 (19.7)	34,697 (11.4)	27,746 (11.2)	0.136
Cardiovascular disease	131,334 (8.2)	18,901 (21.2)	10,236 (15.9)	19,408 (6.4)	15,924 (6.4)	0.238
Myocardial Infarction	11,830 (0.7)	2,495 (2.8)	1,039 (1.6)	1,919 (0.6)	1,388 (0.6)	0.090
Chronic heart failure	16,446 (1.0)	4,846 (5.4)	1,951 (3.0)	2,618 (0.9)	1,644 (0.7)	0.147
Peripheral vascular disease	4,597 (0.3)	1,016 (1.1)	428 (0.7)	590 (0.2)	478 (0.2)	0.062
Cerebrovascular disease	60,849 (3.8)	10,109 (11.3)	5,132 (8.0)	8,876 (2.9)	6,599 (2.7)	0.185
COPD	31,102 (1.9)	4,777 (5.4)	2,687 (4.2)	5,983 (2.0)	4,681 (1.9)	0.101
Dementia	4,846 (0.3)	2,211 (2.5)	898 (1.4)	876 (0.3)	413 (0.2)	0.109
Paralysis	3,561 (0.2)	875 (1.0)	330 (0.5)	535 (0.2)	320 (0.1)	0.059
Diabetes without chronic complication	187,911 (11.7)	15,257 (17.1)	11,935 (18.6)	33,255 (11.0)	26,715 (10.8)	0.125
Diabetes with chronic complication	12,603 (0.8)	2,069 (2.3)	1,104 (1.7)	2,187 (0.7)	1,551 (0.6)	0.075
Chronic Kidney Disease	19,741 (1.2)	4,223 (4.7)	2,048 (3.2)	3,214 (1.1)	2,521 (1.0)	0.121
Mild liver disease	2,369 (0.1)	438 (0.5)	191 (0.3)	460 (0.2)	306 (0.1)	0.033
Moderate-severe liver disease	1,563 (0.1)	345 (0.4)	132 (0.2)	346 (0.1)	205 (0.1)	0.031
Ulcers	16,784 (1.0)	2,269 (2.5)	1,262 (2.0)	3,055 (1.0)	2,394 (1.0)	0.064
Rheumatoid arthritis and other inflammatory polyarthropathies	6,444 (0.4)	676 (0.8)	415 (0.6)	1,206 (0.4)	1,001 (0.4)	0.026
Acquired Immune Deficiency Syndrome (AIDS)	2 (0.0)	0 (0.0)	0 (0.0)	0 (0.0)	0 (0.0)	0.001
Malignancy	47,736 (3.0)	5,764 (6.5)	3,510 (5.5)	7,911 (2.6)	5,976 (2.4)	0.109

Metastatic solid tumour	7,566 (0.5)	1,445 (1.6)	717 (1.1)	1,305 (0.4)	792 (0.3)	0.070
**Medication history**						
Renin-angiotensin-system agents	239,892 (15.0)	20,662 (23.2)	15,413 (24.0)	41,080 (13.5)	34,314 (13.8)	0.157
Beta blockers	151,174 (9.5)	15,373 (17.2)	10,640 (16.6)	25,550 (8.4)	19,690 (7.9)	0.163
Calcium channel blockers	338,241 (21.1)	27,343 (30.6)	20,777 (32.4)	58,106 (19.2)	48,538 (19.5)	0.174
Diuretics	50,242 (3.1)	9,656 (10.8)	4,704 (7.3)	8,521 (2.8)	6,378 (2.6)	0.177
Nitrates	37,099 (2.3)	5,911 (6.6)	3,115 (4.9)	5,782 (1.9)	4,904 (2.0)	0.127
Lipid lowering agents	347,668 (21.7)	26,742 (30.0)	20,715 (32.3)	56,458 (18.6)	50,766 (20.4)	0.171
Insulins	27,766 (1.7)	4,836 (5.4)	2,521 (3.9)	5,138 (1.7)	3,582 (1.4)	0.116
Antidiabetic drugs	181,371 (11.3)	14,770 (16.5)	12,031 (18.7)	33,368 (11.0)	26,794 (10.8)	0.123
Oral anticoagulants	20,176 (1.3)	3,869 (4.3)	1,946 (3.0)	2,938 (1.0)	2,262 (0.9)	0.117
Antiplatelets	135,488 (8.5)	17,449 (19.6)	10,370 (16.2)	20,919 (6.9)	17,487 (7.0)	0.210
Immunosuppressants	7,060 (0.4)	986 (1.1)	560 (0.9)	1,366 (0.5)	1,250 (0.5)	0.041
**Number of past healthcare visits**						
Hospital admission	0.3 (2.0)	1.5 (6.1)	1.0 (5.6)	0.4 (2.9)	0.3 (3.1)	0.130
Doctor consultation	2.8 (3.8)	4.0 (5.1)	4.3 (4.8)	3.0 (4.1)	2.9 (3.9)	0.187
**Doses of COVID-19 vaccines received**						
0	289,728 (18.1)	89,246 (100.0)	0 (0.0)	0 (0.0)	0 (0.0)	
1	136,684 (8.5)	0 (0.0)	64,208 (100.0)	0 (0.0)	0 (0.0)	
2	605,296 (37.8)	0 (0.0)	0 (0.0)	303,213 (100.0)	0 (0.0)	
3 or above	568,003 (35.5)	0 (0.0)	0 (0.0)	0 (0.0)	248,478 (100.0)	

Note: SMD: Standard mean difference; COPD: Chronic obstructive pulmonary disease
Age, Charlson Comorbidity Index, Inpatient hospital and Specialist and general outpatient visit are presented in mean ± SD
SMD ≤ 0.1 is considered good balance between cohorts

Supplementary Table 3. Incidence rate and hazard ratio of clinical sequelae in COVID-19 and non-COVID-19 cohort after weighting during 0-30, 31-90, 91-180, 181-270, 271-365 days of SARS-CoV-2 infection (Patients with a positive laboratory confirmed polymerase chain reaction COVID-19 screening test result)

0-30 days

	Controls (REF)		COVID-19											
			Unvaccinated			Received single dose			Received two doses			Received booster dose(s)		
**Clinical sequelae**	**Event**	**Incidence per 1,000 person-years**	**Event**	**Incidence per 1,000 person-years**	**Hazard ratio**	**Event**	**Incidence per 1,000 person-years**	**Hazard ratio**	**Event**	**Incidence per 1,000 person-years**	**Hazard ratio**	**Event**	**Incidence per 1,000 person-years**	**Hazard ratio**
Major CVD	832	4.64 (4.33,4.96)	4,969	28.76 (27.97,29.56)	6.16 (5.25,7.22)	3,753	21.48 (20.80,22.17)	4.61 (3.79,5.61)	2,971	16.71 (16.12,17.32)	3.60 (3.11,4.16)	2,638	14.71 (14.16,15.28)	3.17 (2.70,3.72)
Stroke	531	2.82 (2.59,3.07)	3,017	16.73 (16.14,17.33)	5.87 (4.73,7.29)	2,272	12.43 (11.93,12.95)	4.38 (3.38,5.68)	2,078	11.17 (10.69,11.65)	3.95 (3.25,4.79)	1,760	9.33 (8.90,9.78)	3.31 (2.65,4.13)
Myocardial infarction	194	1.00 (0.86,1.14)	1,631	8.65 (8.24,9.08)	8.60 (6.54,11.30)	1,420	7.47 (7.09,7.87)	7.46 (5.35,10.40)	930	4.83 (4.53,5.15)	4.84 (3.64,6.43)	956	4.90 (4.60,5.22)	4.92 (3.71,6.52)
Heart Failure	193	1.00 (0.87,1.15)	1,082	5.78 (5.44,6.13)	5.76 (4.20,7.90)	854	4.52 (4.22,4.83)	4.51 (3.09,6.60)	557	2.91 (2.68,3.16)	2.91 (2.07,4.08)	389	2.00 (1.81,2.21)	2.00 (1.34,3.01)
Atrial fibrillation	99	0.51 (0.42,0.62)	565	3.04 (2.80,3.30)	5.90 (3.81,9.12)	482	2.57 (2.35,2.81)	5.00 (3.04,8.25)	349	1.83 (1.65,2.03)	3.57 (2.32,5.50)	483	2.50 (2.29,2.73)	4.88 (3.33,7.15)
Coronary Artery Disease	295	1.57 (1.40,1.75)	1,810	9.88 (9.43,10.35)	6.25 (4.84,8.05)	1,559	8.47 (8.06,8.90)	5.38 (3.97,7.28)	1,161	6.23 (5.88,6.60)	3.96 (3.12,5.04)	1,067	5.68 (5.34,6.03)	3.62 (2.82,4.64)
Deep vein thrombosis	33	0.17 (0.12,0.24)	204	1.08 (0.94,1.23)	6.32 (2.81,14.23)	202	1.06 (0.92,1.21)	6.24 (2.68,14.51)	92	0.47 (0.38,0.58)	2.79 (1.17,6.65)	74	0.37 (0.30,0.47)	2.21 (0.91,5.37)
Cardiovascular mortality	796	4.06 (3.78,4.35)	5,105	26.84 (26.11,27.58)	6.57 (5.64,7.65)	3,002	15.67 (15.11,16.23)	3.85 (3.14,4.72)	1,917	9.87 (9.44,10.32)	2.43 (2.01,2.94)	1,100	5.59 (5.26,5.93)	1.38 (1.04,1.82)
Chronic pulmonary disease	74	0.39 (0.31,0.48)	317	1.71 (1.53,1.91)	4.41 (2.51,7.74)	650	3.48 (3.22,3.76)	9.00 (5.19,15.59)	372	1.96 (1.77,2.17)	5.07 (3.24,7.95)	244	1.27 (1.12,1.44)	3.30 (2.02,5.38)
Acute respiratory distress syndrome	128	0.66 (0.55,0.78)	1,227	6.51 (6.15,6.88)	9.82 (6.89,14.01)	705	3.71 (3.44,3.99)	5.61 (3.63,8.68)	410	2.12 (1.92,2.33)	3.22 (2.17,4.78)	480	2.45 (2.24,2.68)	3.73 (2.56,5.44)
Seizure	71	0.36 (0.28,0.45)	648	3.46 (3.20,3.74)	9.47 (5.82,15.42)	489	2.58 (2.36,2.82)	7.09 (3.79,13.27)	194	1.00 (0.87,1.15)	2.77 (1.63,4.68)	236	1.21 (1.06,1.37)	3.33 (1.70,6.50)
End-stage renal disease	17	0.09 (0.05,0.13)	208	1.10 (0.96,1.25)	12.54 (5.49,28.66)	55	0.29 (0.22,0.37)	3.33 (0.81,13.74)	89	0.46 (0.37,0.56)	5.31 (2.12,13.29)	90	0.46 (0.37,0.56)	5.31 (1.28,21.92)
Acute kidney injury	57	0.29 (0.23,0.38)	676	3.58 (3.32,3.86)	12.18 (7.69,19.29)	803	4.22 (3.93,4.52)	14.37 (8.73,23.66)	305	1.58 (1.41,1.76)	5.38 (3.21,9.04)	381	1.95 (1.76,2.15)	6.65 (3.98,11.12)
Pancreatitis	35	0.18 (0.12,0.24)	235	1.24 (1.09,1.41)	6.94 (3.26,14.79)	135	0.71 (0.59,0.83)	3.96 (1.25,12.51)	125	0.64 (0.54,0.76)	3.62 (1.75,7.47)	138	0.70 (0.59,0.82)	3.94 (2.03,7.66)
All-cause mortality	4,137	21.09 (20.46,21.74)	82,112	431.67 (428.72,434.63)	20.37 (19.20,21.62)	43,871	228.94 (226.80,231.09)	10.83 (10.09,11.62)	19,499	100.37 (98.97,101.78)	4.75 (4.42,5.12)	9,190	46.66 (45.71,47.62)	2.21 (1.96,2.50)

Note: CI: confidence interval, REF: Reference group; Major CVD: Major Cardiovascular Disease; Composite outcome of stroke, heart failure and coronary artery disease

Hazard Ratio (HR) and 95% Confidence Interval (95% CI) were estimated by Cox regression, HR >1 (or <1) indicates patients with COVID-19 had higher (lower) risk of sequelae compared to the non-COVID-19 control cohort

Un-vaccinated, Incomplete vaccinated, Fully Vaccinated and Received booster dose of COVID-19 vaccines refers to patients who have received 0 dose, 1 doses, 2 doses and $\geq$3 doses of BioNtech or CoronaVac COVID-19 vaccines, respectively

Supplementary Table 3. Incidence rate and hazard ratio of clinical sequelae in COVID-19 and non-COVID-19 cohort after weighting during 0-30, 31-90, 91-180, 181-270, 271-365 days of SARS-CoV-2 infection (Patients with a positive laboratory confirmed polymerase chain reaction COVID-19 screening test result, continued)

31-90 days

	Controls (REF)		COVID-19											
			Unvaccinated			Received single dose			Received two doses			Received booster dose(s)		
Clinical sequelae	Event	Incidence per 1,000 person-years	Event	Incidence per 1,000 person-years	Hazard ratio	Event	Incidence per 1,000 person-years	Hazard ratio	Event	Incidence per 1,000 person-years	Hazard ratio	Event	Incidence per 1,000 person-years	Hazard ratio
Major CVD	1,989	5.66 (5.41,5.91)	4,078	12.15 (11.78,12.53)	2.15 (1.83,2.53)	3,193	9.33 (9.01,9.65)	1.65 (1.35,2.01)	2,237	6.43 (6.17,6.70)	1.14 (0.98,1.31)	2,397	6.85 (6.58,7.13)	1.21 (1.05,1.39)
Stroke	1,115	3.03 (2.86,3.21)	1,891	5.39 (5.15,5.64)	1.78 (1.41,2.25)	1,898	5.30 (5.07,5.54)	1.75 (1.34,2.28)	1,181	3.25 (3.07,3.44)	1.07 (0.88,1.31)	1,121	3.05 (2.88,3.23)	1.01 (0.82,1.24)
Myocardial infarction	453	1.19 (1.09,1.31)	839	2.28 (2.13,2.44)	1.91 (1.39,2.64)	450	1.21 (1.10,1.33)	1.01 (0.59,1.73)	490	1.30 (1.19,1.42)	1.09 (0.80,1.49)	499	1.31 (1.20,1.44)	1.10 (0.79,1.53)
Heart Failure	488	1.29 (1.18,1.41)	1,813	4.97 (4.75,5.21)	3.86 (3.06,4.86)	887	2.39 (2.24,2.56)	1.86 (1.32,2.61)	837	2.23 (2.09,2.39)	1.73 (1.33,2.26)	772	2.04 (1.90,2.19)	1.58 (1.15,2.18)
Atrial fibrillation	318	0.84 (0.75,0.94)	858	2.37 (2.22,2.53)	2.81 (2.02,3.91)	532	1.45 (1.33,1.57)	1.71 (1.09,2.71)	405	1.09 (0.99,1.20)	1.29 (0.92,1.81)	368	0.98 (0.88,1.08)	1.16 (0.79,1.70)
Coronary Artery Disease	798	2.17 (2.03,2.33)	1,601	4.49 (4.27,4.71)	2.06 (1.61,2.65)	886	2.46 (2.30,2.62)	1.13 (0.79,1.62)	960	2.64 (2.47,2.81)	1.21 (0.97,1.52)	1,109	3.03 (2.86,3.21)	1.39 (1.13,1.72)
Deep vein thrombosis	82	0.22 (0.17,0.27)	337	0.91 (0.82,1.01)	4.23 (2.35,7.62)	223	0.60 (0.52,0.68)	2.76 (1.38,5.53)	156	0.41 (0.35,0.48)	1.91 (1.05,3.47)	45	0.12 (0.09,0.15)	0.54 (0.22,1.37)
Cardiovascular mortality	927	2.42 (2.27,2.58)	2,029	5.48 (5.24,5.72)	2.26 (1.84,2.78)	1,118	2.98 (2.81,3.16)	1.23 (0.92,1.64)	1,028	2.71 (2.55,2.88)	1.12 (0.88,1.42)	615	1.60 (1.48,1.73)	0.66 (0.46,0.96)
Chronic pulmonary disease	209	0.55 (0.48,0.63)	620	1.72 (1.59,1.86)	3.09 (2.04,4.68)	450	1.23 (1.12,1.35)	2.22 (1.22,4.01)	318	0.86 (0.77,0.95)	1.54 (1.03,2.31)	179	0.48 (0.41,0.55)	0.86 (0.51,1.45)
Acute respiratory distress syndrome	328	0.86 (0.77,0.96)	759	2.07 (1.93,2.22)	2.40 (1.64,3.53)	687	1.84 (1.71,1.98)	2.14 (1.37,3.35)	556	1.47 (1.35,1.60)	1.71 (1.26,2.33)	580	1.52 (1.40,1.65)	1.77 (1.20,2.61)
Seizure	190	0.50 (0.43,0.57)	486	1.33 (1.22,1.45)	2.66 (1.58,4.47)	481	1.29 (1.18,1.41)	2.59 (1.44,4.67)	225	0.60 (0.52,0.68)	1.19 (0.76,1.87)	189	0.50 (0.43,0.57)	0.99 (0.54,1.83)
End-stage renal disease	50	0.13 (0.10,0.17)	87	0.24 (0.19,0.29)	1.81 (0.60,5.46)	134	0.36 (0.30,0.42)	2.75 (0.97,7.80)	60	0.16 (0.12,0.20)	1.22 (0.46,3.23)	17	0.04 (0.03,0.07)	0.33 (0.08,1.45)
Acute kidney injury	142	0.37 (0.32,0.44)	607	1.65 (1.52,1.79)	4.41 (2.89,6.74)	403	1.08 (0.98,1.19)	2.89 (1.60,5.21)	219	0.58 (0.51,0.66)	1.55 (0.92,2.62)	209	0.55 (0.48,0.63)	1.47 (0.81,2.66)
Pancreatitis	93	0.24 (0.20,0.30)	361	0.98 (0.88,1.08)	4.01 (2.14,7.49)	197	0.53 (0.46,0.60)	2.15 (0.95,4.91)	90	0.24 (0.19,0.29)	0.97 (0.46,2.03)	108	0.28 (0.23,0.34)	1.15 (0.60,2.22)
All-cause mortality	5,467	14.27 (13.89,14.65)	23,694	63.95 (63.14,64.76)	4.47 (4.17,4.79)	11,685	31.13 (30.57,31.70)	2.18 (1.97,2.41)	7,377	19.44 (19.00,19.89)	1.36 (1.24,1.49)	5,930	15.47 (15.08,15.87)	1.08 (0.94,1.25)

Note: CI: confidence interval, REF: Reference group; Major CVD: Major Cardiovascular Disease; Composite outcome of stroke, heart failure and coronary artery disease

Hazard Ratio (HR) and 95% Confidence Interval (95% CI) were estimated by Cox regression, HR >1 (or <1) indicates patients with COVID-19 had higher (lower) risk of sequelae compared to the non-COVID-19 control cohort

Un-vaccinated, Incomplete vaccinated, Fully Vaccinated and Received booster dose of COVID-19 vaccines refers to patients who have received 0 dose, 1 doses, 2 doses and ≥3 doses of BioNtech or CoronaVac COVID-19 vaccines, respectively

Supplementary Table 3. Incidence rate and hazard ratio of clinical sequelae in COVID-19 and non-COVID-19 cohort after weighting during 0-30, 31-90, 91-180, 181-270, 271-365 days of SARS-CoV-2 infection (Patients with a positive laboratory confirmed polymerase chain reaction COVID-19 screening test result, continued)

91-180 days

	Controls (REF)		COVID-19											
			Unvaccinated			Received single dose			Received two doses			Received booster dose(s)		
**Clinical sequelae**	**Event**	**Incidence per 1,000 person-years**	**Event**	**Incidence per 1,000 person-years**	**Hazard ratio**	**Event**	**Incidence per 1,000 person-years**	**Hazard ratio**	**Event**	**Incidence per 1,000 person-years**	**Hazard ratio**	**Event**	**Incidence per 1,000 person-years**	**Hazard ratio**
Major CVD	2,869	5.97 (5.75,6.19)	4,165	8.63 (8.37,8.90)	1.45 (1.23,1.71)	4,187	8.32 (8.07,8.58)	1.40 (1.17,1.68)	3,171	6.27 (6.05,6.49)	1.05 (0.94,1.19)	2,376	5.59 (5.37,5.82)	0.93 (0.81,1.07)
Stroke	1,664	3.31 (3.15,3.47)	2,038	4.04 (3.86,4.21)	1.22 (0.97,1.54)	2,190	4.16 (3.99,4.34)	1.26 (0.98,1.62)	1,730	3.27 (3.12,3.43)	0.99 (0.84,1.17)	1,127	2.54 (2.39,2.69)	0.76 (0.62,0.92)
Myocardial infarction	643	1.24 (1.15,1.34)	967	1.83 (1.71,1.94)	1.47 (1.05,2.06)	894	1.63 (1.53,1.74)	1.32 (0.90,1.94)	601	1.10 (1.01,1.19)	0.89 (0.67,1.18)	657	1.44 (1.33,1.55)	1.16 (0.86,1.57)
Heart Failure	569	1.10 (1.01,1.19)	1,473	2.81 (2.67,2.95)	2.56 (1.98,3.31)	1,271	2.33 (2.21,2.46)	2.13 (1.55,2.94)	972	1.79 (1.68,1.90)	1.63 (1.29,2.07)	583	1.28 (1.18,1.38)	1.15 (0.84,1.57)
Atrial fibrillation	460	0.89 (0.82,0.98)	702	1.35 (1.25,1.45)	1.50 (1.04,2.18)	689	1.27 (1.18,1.37)	1.42 (0.96,2.11)	682	1.26 (1.17,1.36)	1.41 (1.08,1.84)	449	0.99 (0.90,1.09)	1.11 (0.77,1.59)
Coronary Artery Disease	1,171	2.33 (2.20,2.47)	1,719	3.35 (3.19,3.51)	1.44 (1.12,1.84)	1,729	3.26 (3.11,3.42)	1.40 (1.07,1.84)	1,212	2.29 (2.17,2.43)	0.98 (0.81,1.19)	1,114	2.52 (2.38,2.67)	1.08 (0.87,1.33)
Deep vein thrombosis	99	0.19 (0.16,0.23)	112	0.21 (0.17,0.25)	1.11 (0.47,2.64)	229	0.42 (0.36,0.47)	2.19 (0.96,4.98)	66	0.12 (0.09,0.15)	0.63 (0.28,1.43)	138	0.30 (0.25,0.35)	1.58 (0.63,3.96)
Cardiovascular mortality	1,177	2.25 (2.13,2.38)	1,985	3.72 (3.56,3.89)	1.65 (1.33,2.05)	1,285	2.33 (2.21,2.46)	1.04 (0.77,1.39)	748	1.36 (1.26,1.46)	0.60 (0.47,0.78)	371	0.80 (0.73,0.89)	0.36 (0.24,0.54)
Chronic pulmonary disease	280	0.54 (0.48,0.61)	727	1.40 (1.30,1.51)	2.58 (1.71,3.89)	579	1.08 (0.99,1.17)	1.99 (1.19,3.33)	391	0.73 (0.66,0.80)	1.34 (0.94,1.90)	339	0.75 (0.68,0.84)	1.37 (0.89,2.09)
Acute respiratory distress syndrome	497	0.96 (0.87,1.04)	932	1.76 (1.65,1.88)	1.85 (1.31,2.62)	801	1.46 (1.36,1.56)	1.54 (1.02,2.31)	680	1.24 (1.15,1.34)	1.31 (1.00,1.70)	357	0.78 (0.70,0.86)	0.81 (0.56,1.17)
Seizure	268	0.52 (0.46,0.58)	787	1.50 (1.40,1.61)	2.93 (1.96,4.39)	764	1.40 (1.30,1.50)	2.75 (1.74,4.36)	305	0.56 (0.50,0.62)	1.09 (0.74,1.61)	295	0.64 (0.57,0.72)	1.21 (0.60,2.44)
End-stage renal disease	68	0.13 (0.10,0.16)	39	0.07 (0.05,0.10)	0.57 (0.16,2.00)	137	0.25 (0.21,0.29)	1.92 (0.64,5.78)	38	0.07 (0.05,0.09)	0.53 (0.17,1.60)	0	NA	NA
Acute kidney injury	236	0.45 (0.40,0.51)	475	0.90 (0.82,0.98)	1.99 (1.24,3.18)	350	0.64 (0.57,0.71)	1.42 (0.83,2.44)	335	0.61 (0.55,0.68)	1.36 (0.91,2.03)	103	0.22 (0.18,0.27)	0.49 (0.21,1.11)
Pancreatitis	116	0.22 (0.18,0.26)	167	0.31 (0.27,0.36)	1.41 (0.65,3.09)	40	0.07 (0.05,0.10)	0.33 (0.10,1.10)	146	0.27 (0.23,0.31)	1.20 (0.66,2.16)	94	0.20 (0.17,0.25)	0.93 (0.44,1.93)
All-cause mortality	7,161	13.69 (13.37,14.01)	16,508	30.92 (30.45,31.40)	2.26 (2.10,2.45)	10,136	18.37 (18.02,18.73)	1.35 (1.21,1.50)	6,664	12.10 (11.82,12.40)	0.89 (0.81,0.97)	4,789	10.40 (10.11,10.70)	0.75 (0.64,0.89)

Note: CI: confidence interval, REF: Reference group; Major CVD: Major Cardiovascular Disease; Composite outcome of stroke, heart failure and coronary artery disease

Hazard Ratio (HR) and 95% Confidence Interval (95% CI) were estimated by Cox regression, HR >1 (or <1) indicates patients with COVID-19 had higher (lower) risk of sequelae compared to the non-COVID-19 control cohort

Un-vaccinated, Incomplete vaccinated, Fully Vaccinated and Received booster dose of COVID-19 vaccines refers to patients who have received 0 dose, 1 doses, 2 doses and ≥3 doses of BioNtech or CoronaVac COVID-19 vaccines, respectively

Supplementary Table 3. Incidence rate and hazard ratio of clinical sequelae in COVID-19 and non-COVID-19 cohort after weighting during 0-30, 31-90, 91-180, 181-270, 271-365 days of SARS-CoV-2 infection (Patients with a positive laboratory confirmed polymerase chain reaction COVID-19 screening test result, continued)

181-270 days

	Controls (REF)		COVID-19											
			Unvaccinated			Received single dose			Received two doses			Received booster dose(s)		
**Clinical sequelae**	**Event**	**Incidence per 1,000 person-years**	**Event**	**Incidence per 1,000 person-years**	**Hazard ratio**	**Event**	**Incidence per 1,000 person-years**	**Hazard ratio**	**Event**	**Incidence per 1,000 person-years**	**Hazard ratio**	**Event**	**Incidence per 1,000 person-years**	**Hazard ratio**
Major CVD	2,500	6.04 (5.80,6.28)	3,807	9.34 (9.05,9.64)	1.55 (1.31,1.83)	3,599	8.54 (8.26,8.82)	1.41 (1.18,1.70)	2,555	6.05 (5.82,6.29)	1.00 (0.89,1.13)	2,039	5.38 (5.15,5.62)	0.89 (0.74,1.06)
Stroke	1,383	3.20 (3.03,3.37)	2,132	5.01 (4.80,5.22)	1.57 (1.25,1.96)	1,589	3.61 (3.43,3.79)	1.13 (0.86,1.48)	1,408	3.19 (3.03,3.36)	1.00 (0.84,1.18)	1,141	2.88 (2.71,3.05)	0.90 (0.71,1.14)
Myocardial infarction	612	1.37 (1.26,1.48)	802	1.80 (1.68,1.93)	1.31 (0.95,1.82)	914	2.00 (1.87,2.13)	1.46 (1.01,2.10)	503	1.11 (1.01,1.21)	0.81 (0.61,1.07)	655	1.60 (1.48,1.73)	1.17 (0.64,2.14)
Heart Failure	541	1.21 (1.11,1.32)	1,547	3.49 (3.32,3.67)	2.88 (2.25,3.68)	1,262	2.77 (2.62,2.93)	2.28 (1.70,3.07)	562	1.24 (1.14,1.34)	1.02 (0.77,1.36)	306	0.75 (0.67,0.84)	0.62 (0.35,1.10)
Atrial fibrillation	404	0.91 (0.83,1.00)	726	1.65 (1.54,1.78)	1.81 (1.27,2.59)	397	0.88 (0.80,0.97)	0.96 (0.59,1.58)	600	1.33 (1.23,1.44)	1.46 (1.11,1.93)	291	0.72 (0.64,0.80)	0.78 (0.45,1.35)
Coronary Artery Disease	1,081	2.50 (2.36,2.65)	1,268	2.93 (2.77,3.09)	1.17 (0.90,1.52)	1,618	3.65 (3.48,3.83)	1.46 (1.12,1.91)	1,163	2.64 (2.49,2.80)	1.06 (0.88,1.27)	911	2.31 (2.16,2.46)	0.92 (0.70,1.20)
Deep vein thrombosis	78	0.17 (0.14,0.21)	63	0.14 (0.11,0.18)	0.81 (0.31,2.14)	70	0.15 (0.12,0.19)	0.88 (0.33,2.38)	95	0.21 (0.17,0.25)	1.20 (0.63,2.29)	51	0.12 (0.09,0.16)	0.72 (0.26,2.02)
Cardiovascular mortality	738	1.64 (1.53,1.76)	1,373	3.05 (2.90,3.22)	1.86 (1.46,2.38)	774	1.68 (1.56,1.80)	1.03 (0.73,1.44)	423	0.92 (0.84,1.02)	0.56 (0.41,0.78)	437	1.06 (0.96,1.16)	0.64 (0.36,1.13)
Chronic pulmonary disease	259	0.59 (0.52,0.66)	708	1.62 (1.50,1.74)	2.75 (1.86,4.07)	527	1.17 (1.07,1.27)	2.00 (1.25,3.20)	263	0.59 (0.52,0.66)	1.00 (0.68,1.47)	169	0.42 (0.36,0.49)	0.72 (0.41,1.25)
Acute respiratory distress syndrome	388	0.87 (0.78,0.96)	590	1.32 (1.22,1.44)	1.53 (1.00,2.33)	339	0.74 (0.67,0.82)	0.85 (0.49,1.48)	357	0.78 (0.70,0.87)	0.90 (0.65,1.26)	471	1.15 (1.05,1.26)	1.34 (0.61,2.96)
Seizure	241	0.54 (0.47,0.61)	719	1.63 (1.51,1.75)	3.03 (2.01,4.56)	744	1.63 (1.52,1.75)	3.04 (1.93,4.78)	175	0.38 (0.33,0.44)	0.71 (0.43,1.18)	182	0.44 (0.38,0.51)	0.82 (0.21,3.23)
End-stage renal disease	36	0.08 (0.06,0.11)	68	0.15 (0.12,0.19)	1.89 (0.63,5.71)	106	0.23 (0.19,0.28)	2.87 (1.18,6.99)	72	0.16 (0.12,0.20)	1.98 (0.89,4.39)	0	NA	NA
Acute kidney injury	220	0.49 (0.43,0.56)	338	0.76 (0.68,0.84)	1.54 (0.96,2.46)	263	0.57 (0.51,0.65)	1.17 (0.64,2.14)	204	0.45 (0.39,0.51)	0.91 (0.56,1.47)	103	0.25 (0.20,0.30)	0.51 (0.23,1.11)
Pancreatitis	129	0.29 (0.24,0.34)	243	0.54 (0.48,0.61)	1.89 (0.98,3.64)	161	0.35 (0.30,0.41)	1.22 (0.47,3.21)	102	0.22 (0.18,0.27)	0.78 (0.43,1.41)	44	0.11 (0.08,0.14)	0.37 (0.13,1.00)
All-cause mortality	5,985	13.30 (12.97,13.64)	12,301	27.35 (26.87,27.84)	2.06 (1.89,2.24)	6,660	14.44 (14.10,14.79)	1.09 (0.97,1.22)	4,141	9.04 (8.76,9.31)	0.68 (0.61,0.76)	6,580	15.95 (15.57,16.34)	1.19 (0.76,1.87)

Note: CI: confidence interval, REF: Reference group; Major CVD: Major Cardiovascular Disease; Composite outcome of stroke, heart failure and coronary artery disease

Hazard Ratio (HR) and 95% Confidence Interval (95% CI) were estimated by Cox regression, HR >1 (or <1) indicates patients with COVID-19 had higher (lower) risk of sequelae compared to the non-COVID-19 control cohort

Un-vaccinated, Incomplete vaccinated, Fully Vaccinated and Received booster dose of COVID-19 vaccines refers to patients who have received 0 dose, 1 doses, 2 doses and ≥3 doses of BioNtech or CoronaVac COVID-19 vaccines, respectively

Supplementary Table 3. Incidence rate and hazard ratio of clinical sequelae in COVID-19 and non-COVID-19 cohort after weighting during 0-30, 31-90, 91-180, 181-270, 271-365 days of SARS-CoV-2 infection (Patients with a positive laboratory confirmed polymerase chain reaction COVID-19 screening test result, continued)

271-365 days

	Controls (REF)		COVID-19												
			Unvaccinated			Received single dose			Received two doses			Received booster dose(s)			
**Clinical sequelae**	**Event**	**Incidence per 1,000 person-years**	**Event**	**Incidence per 1,000 person-years**	**Hazard ratio**	**Event**	**Incidence per 1,000 person-years**	**Hazard ratio**	**Event**	**Incidence per 1,000 person-years**	**Hazard ratio**	**Event**	**Incidence per 1,000 person-years**	**Hazard ratio**
Major CVD	1,532	6.12 (5.81,6.43)	1,889	7.02 (6.71,7.34)	1.24 (0.98,1.56)	1,659	7.00 (6.67,7.35)	1.13 (0.87,1.46)	1,445	5.87 (5.58,6.18)	0.96 (0.81,1.12)	1,105	4.64 (4.37,4.92)	0.74 (0.58,0.95)
Stroke	821	3.14 (2.93,3.36)	917	3.28 (3.07,3.50)	1.10 (0.79,1.53)	622	2.52 (2.32,2.72)	0.79 (0.51,1.22)	762	2.98 (2.77,3.19)	0.94 (0.76,1.18)	563	2.24 (2.06,2.43)	0.70 (0.48,1.01)
Myocardial infarction	382	1.42 (1.28,1.57)	423	1.46 (1.32,1.60)	1.12 (0.71,1.76)	435	1.70 (1.55,1.87)	1.18 (0.72,1.92)	338	1.28 (1.15,1.43)	0.90 (0.63,1.28)	198	0.76 (0.66,0.87)	0.53 (0.32,0.88)
Heart Failure	430	1.60 (1.46,1.76)	794	2.75 (2.57,2.95)	1.88 (1.35,2.62)	697	2.73 (2.54,2.94)	1.68 (1.18,2.41)	539	2.05 (1.88,2.23)	1.27 (0.94,1.73)	193	0.75 (0.64,0.86)	0.46 (0.24,0.86)
Atrial fibrillation	216	0.81 (0.70,0.92)	417	1.45 (1.32,1.60)	1.91 (1.18,3.09)	315	1.24 (1.11,1.39)	1.53 (0.82,2.84)	261	1.00 (0.88,1.13)	1.23 (0.82,1.83)	265	1.03 (0.91,1.16)	1.26 (0.57,2.78)
Coronary Artery Disease	628	2.41 (2.22,2.60)	659	2.33 (2.16,2.51)	1.03 (0.70,1.51)	732	2.95 (2.74,3.17)	1.22 (0.83,1.79)	532	2.08 (1.91,2.26)	0.86 (0.66,1.13)	550	2.21 (2.03,2.40)	0.90 (0.58,1.41)
Deep vein thrombosis	41	0.15 (0.11,0.21)	68	0.23 (0.18,0.29)	1.61 (0.51,5.05)	35	0.14 (0.09,0.18)	0.88 (0.21,3.67)	30	0.11 (0.08,0.16)	0.74 (0.25,2.17)	27	0.10 (0.07,0.15)	0.67 (0.16,2.81)
Cardiovascular mortality	82	0.30 (0.24,0.38)	184	0.63 (0.54,0.73)	1.93 (0.89,4.19)	16	0.06 (0.04,0.10)	0.20 (0.03,1.46)	8	0.03 (0.01,0.05)	0.10 (0.01,0.70)	0	NA	NA
Chronic pulmonary disease	224	0.84 (0.73,0.95)	326	1.14 (1.02,1.27)	1.49 (0.84,2.63)	430	1.71 (1.55,1.87)	2.01 (1.15,3.51)	270	1.04 (0.92,1.16)	1.23 (0.80,1.87)	80	0.31 (0.25,0.39)	0.37 (0.17,0.79)
Acute respiratory distress syndrome	220	0.82 (0.71,0.93)	320	1.10 (0.99,1.23)	1.49 (0.82,2.68)	385	1.50 (1.36,1.66)	1.81 (1.10,2.96)	251	0.95 (0.84,1.07)	1.16 (0.75,1.79)	490	1.89 (1.73,2.07)	2.26 (0.57,8.88)
Seizure	136	0.51 (0.43,0.60)	226	0.78 (0.69,0.89)	1.68 (0.87,3.26)	75	0.29 (0.23,0.36)	0.57 (0.18,1.86)	128	0.49 (0.41,0.58)	0.95 (0.58,1.57)	123	0.47 (0.39,0.56)	0.92 (0.25,3.36)
End-stage renal disease	41	0.15 (0.11,0.20)	160	0.55 (0.47,0.64)	4.09 (1.59,10.51)	45	0.17 (0.13,0.23)	1.14 (0.26,5.11)	4	0.02 (0.01,0.04)	0.11 (0.01,0.81)	55	0.21 (0.16,0.27)	1.36 (0.18,10.44)
Acute kidney injury	111	0.41 (0.34,0.49)	109	0.37 (0.31,0.45)	0.99 (0.42,2.36)	47	0.18 (0.13,0.24)	0.43 (0.10,1.87)	74	0.28 (0.22,0.35)	0.67 (0.32,1.43)	379	1.45 (1.31,1.60)	3.37 (0.59,19.24)
Pancreatitis	65	0.24 (0.19,0.31)	103	0.35 (0.29,0.43)	1.62 (0.62,4.22)	78	0.30 (0.24,0.37)	1.23 (0.31,4.90)	28	0.11 (0.07,0.15)	0.44 (0.15,1.27)	19	0.07 (0.04,0.11)	0.29 (0.04,2.10)
All-cause mortality	3,502	12.92 (12.49,13.35)	7,446	25.46 (24.89,26.05)	2.13 (1.91,2.39)	3,285	12.76 (12.33,13.20)	0.97 (0.82,1.15)	2,480	9.34 (8.98,9.71)	0.72 (0.63,0.82)	856	3.27 (3.05,3.49)	0.25 (0.15,0.41)

Note: CI: confidence interval, REF: Reference group; Major CVD: Major Cardiovascular Disease; Composite outcome of stroke, heart failure and coronary artery disease

Hazard Ratio (HR) and 95% Confidence Interval (95% CI) were estimated by Cox regression, HR >1 (or <1) indicates patients with COVID-19 had higher (lower) risk of sequelae compared to the non-COVID-19 control cohort

Un-vaccinated, Incomplete vaccinated, Fully Vaccinated and Received booster dose of COVID-19 vaccines refers to patients who have received 0 dose, 1 doses, 2 doses and ≥3 doses of BioNtech or CoronaVac COVID-19 vaccines, respectively

Supplementary Table 4. Baseline characteristics of patients with COVID-19 from the Omicron wave in Hong Kong stratified by vaccine status and controls from the Hong Kong Hospital Authority before propensity-score weighting.

	Hong Kong Hospital Authority (April 1st, 2020 to October 31st, 2022)					
		COVID-19				
**Baseline characteristics**	**Controls** (N= 2,626,293)	**Un-vaccinated** (N= 114,677)	**Incomplete vaccination** (N= 101,366)	**Fully vaccinated** (N= 457,596)	**Receieved booster dose** (N= 491,549)	**SMD**[b]
	**N / Mean (% / SD)**	**N / Mean (% / SD)**	**N / Mean (% / SD)**	**N / Mean (% / SD)**	**N / Mean (% / SD)**	
Age (years)	54.6 (18.17)	61.2 (21.98)	59.6 (19.88)	50.2 (17.70)	52.1 (16.07)	0.312
Sex, male	1,148,258 (43.7)	50,527 (44.1)	44,546 (43.9)	201,769 (44.1)	227,567 (46.3)	0.021
Charlson Comorbidity Index	1.68 (1.80)	2.74 (2.56)	2.38 (2.20)	1.33 (1.69)	1.35 (1.57)	0.375
**Pre-existing morbidities**						
Respiratory disease	48,037 (1.8)	5,811 (5.1)	3,759 (3.7)	8,283 (1.8)	7,877 (1.6)	0.101
Diabetes	302,972 (11.5)	20,849 (18.2)	18,406 (18.2)	49,337 (10.8)	48,954 (10.0)	0.138
Cardiovascular disease	197,529 (7.5)	23,641 (20.6)	14,776 (14.6)	27,796 (6.1)	27,318 (5.6)	0.241
Myocardiac Infarction	17,864 (0.7)	2,975 (2.6)	1,443 (1.4)	2,592 (0.6)	2,256 (0.5)	0.089
Chronic heart failure	24,219 (0.9)	5,778 (5.0)	2,613 (2.6)	3,518 (0.8)	2,455 (0.5)	0.145
Peripheral vascular disease	6,853 (0.3)	1,176 (1.0)	574 (0.6)	859 (0.2)	749 (0.2)	0.060
Cerebrovascular disease	91,244 (3.5)	12,733 (11.1)	7,461 (7.4)	12,617 (2.8)	10,975 (2.2)	0.190
COPD	47,867 (1.8)	5,769 (5.0)	3,742 (3.7)	8,263 (1.8)	7,868 (1.6)	0.101
Dementia	7,006 (0.3)	2,773 (2.4)	1,359 (1.3)	1,308 (0.3)	581 (0.1)	0.112
Paralysis	5,321 (0.2)	1,099 (1.0)	485 (0.5)	720 (0.2)	499 (0.1)	0.061
Diabetes without chronic complication	289,744 (11.0)	19,231 (16.8)	17,417 (17.2)	47,368 (10.4)	47,199 (9.6)	0.127
Diabetes with chronic complication	19,264 (0.7)	2,467 (2.2)	1,476 (1.5)	2,973 (0.6)	2,562 (0.5)	0.074
Chronic Kidney Disease	29,397 (1.1)	4,885 (4.3)	2,740 (2.7)	4,304 (0.9)	3,862 (0.8)	0.118
Mild liver disease	3,631 (0.1)	511 (0.4)	255 (0.3)	608 (0.1)	493 (0.1)	0.033
Moderate-severe liver disease	2,399 (0.1)	384 (0.3)	168 (0.2)	436 (0.1)	315 (0.1)	0.029
Ulcers	25,657 (1.0)	2,695 (2.4)	1,722 (1.7)	4,179 (0.9)	3,949 (0.8)	0.064
Rheumatoid arthritis and other inflammatory polyarthropathies	10,593 (0.4)	895 (0.8)	626 (0.6)	1,815 (0.4)	1,858 (0.4)	0.028
Acquired Immune Deficiency Syndrome (AIDS)	6 (0.0)	0 (0.0)	0 (0.0)	0 (0.0)	0 (0.0)	0.001

Malignancy	74,608 (2.8)	7,033 (6.1)	4,994 (4.9)	11,612 (2.5)	11,003 (2.2)	0.104
Metastatic solid tumour	11,601 (0.4)	1,677 (1.5)	937 (0.9)	1,771 (0.4)	1,324 (0.3)	0.066
**Medication history**						
Renin-angiotensin-system agents	48,037 (1.8)	5,811 (5.1)	3,759 (3.7)	8,283 (1.8)	7,877 (1.6)	0.101
Beta blockers	302,972 (11.5)	20,849 (18.2)	18,406 (18.2)	49,337 (10.8)	48,954 (10.0)	0.138
Calcium channel blockers	197,529 (7.5)	23,641 (20.6)	14,776 (14.6)	27,796 (6.1)	27,318 (5.6)	0.241
Diuretics	367,754 (14.0)	26,237 (22.9)	22,748 (22.4)	59,411 (13.0)	61,874 (12.6)	0.159
Nitrates	232,012 (8.8)	19,478 (17.0)	15,683 (15.5)	37,186 (8.1)	35,296 (7.2)	0.168
Lipid lowering agents	519,967 (19.8)	34,747 (30.3)	31,214 (30.8)	84,324 (18.4)	86,862 (17.7)	0.180
Insulins	76,051 (2.9)	11,512 (10.0)	6,563 (6.5)	11,773 (2.6)	10,650 (2.2)	0.173
Antidiabetic drugs	55,622 (2.1)	7,300 (6.4)	4,482 (4.4)	8,262 (1.8)	8,424 (1.7)	0.127
Oral anticoagulants	534,461 (20.4)	34,337 (29.9)	30,985 (30.6)	82,672 (18.1)	92,071 (18.7)	0.170
Antiplatelets	42,736 (1.6)	5,710 (5.0)	3,434 (3.4)	7,038 (1.5)	5,991 (1.2)	0.113
Immunosuppressants	279,426 (10.6)	18,780 (16.4)	17,637 (17.4)	47,595 (10.4)	47,592 (9.7)	0.126
**Number of past healthcare visits**						
Hospital admission	0.3 (1.95)	1.3 (5.50)	0.8 (4.61)	0.3 (2.47)	0.3 (2.30)	0.130
Doctor consultation	2.7 (3.72)	4.0 (4.91)	4.0 (4.63)	2.9 (3.97)	2.7 (3.64)	0.193
**Doses of COVID-19 vaccines received**						
0	419,380 (16.0)	114,677 (100.0)	0 (0.0)	0 (0.0)	0 (0.0)	
1	198,031 (7.5)	0 (0.0)	101,366 (100.0)	0 (0.0)	0 (0.0)	
2	914,115 (34.8)	0 (0.0)	0 (0.0)	457,596 (100.0)	0 (0.0)	
3 or above	1,094,767 (41.7)	0 (0.0)	0 (0.0)	0 (0.0)	491,549 (100.0)	

Supplementary Table 5. Incidence rate and hazard ratio of clinical sequelae in COVID-19 and non-COVID-19 cohort after weighting during 0-30, 31-90, 91-180, 181-270,271-365 days of SARS-CoV-2 infection (Cases of SARS-COV-2 infection from the Omicron wave in Hong Kong)

0-30 days

	Controls (REF)		COVID-19												
			Unvaccinated			Received single dose			Received two doses			Received booster dose(s)		
**Clinical sequelae**	**Event**	**Incidence per 1,000 person-years**	**Event**	**Incidence per 1,000 person-years**	**Hazard ratio**	**Event**	**Incidence per 1,000 person-years**	**Hazard ratio**	**Event**	**Incidence per 1,000 person-years**	**Hazard ratio**	**Event**	**Incidence per 1,000 person-years**	**Hazard ratio**
Major CVD	1,297	4.36 (4.13,4.60)	5,913	20.49 (19.97,21.01)	4.67 (4.02,5.44)	4,012	13.72 (13.31,14.16)	3.14 (2.61,3.78)	3,278	11.08 (10.70,11.46)	2.54 (2.22,2.90)	2,595	8.69 (8.36,9.03)	1.99 (1.73,2.30)
Stroke	812	2.62 (2.44,2.80)	3,522	11.74 (11.35,12.13)	4.45 (3.66,5.42)	2,422	7.96 (7.65,8.28)	3.03 (2.40,3.83)	2,272	7.37 (7.07,7.68)	2.81 (2.39,3.31)	1,690	5.42 (5.16,5.68)	2.07 (1.71,2.51)
Myocardial infarction	297	0.93 (0.83,1.04)	1,924	6.15 (5.88,6.43)	6.58 (5.10,8.49)	1,550	4.92 (4.68,5.17)	5.28 (3.88,7.18)	974	3.06 (2.88,3.26)	3.30 (2.54,4.28)	919	2.86 (2.68,3.05)	3.08 (2.39,3.97)
Heart Failure	267	0.84 (0.74,0.94)	1,368	4.40 (4.17,4.64)	5.24 (3.89,7.05)	907	2.89 (2.71,3.08)	3.45 (2.38,4.98)	639	2.02 (1.86,2.18)	2.41 (1.76,3.28)	352	1.10 (0.99,1.22)	1.31 (0.89,1.94)
Atrial fibrillation	153	0.48 (0.41,0.57)	684	2.21 (2.05,2.38)	4.57 (3.03,6.90)	594	1.91 (1.76,2.06)	3.94 (2.52,6.17)	423	1.34 (1.22,1.48)	2.78 (1.89,4.08)	506	1.59 (1.45,1.73)	3.29 (2.34,4.61)
Coronary Artery Disease	478	1.54 (1.41,1.68)	2,144	7.04 (6.74,7.34)	4.54 (3.57,5.77)	1,658	5.42 (5.16,5.68)	3.50 (2.64,4.66)	1,259	4.08 (3.86,4.31)	2.64 (2.12,3.29)	1,092	3.52 (3.31,3.73)	2.28 (1.83,2.84)
Deep vein thrombosis	40	0.12 (0.09,0.17)	307	0.97 (0.87,1.09)	7.85 (3.82,16.15)	271	0.86 (0.76,0.96)	6.91 (3.24,14.73)	110	0.34 (0.28,0.41)	2.77 (1.28,5.98)	71	0.22 (0.17,0.28)	1.78 (0.78,4.05)
Cardiovascular mortality	1,082	3.36 (3.16,3.56)	6,269	19.88 (19.39,20.38)	5.89 (5.13,6.76)	3,419	10.77 (10.41,11.13)	3.20 (2.66,3.85)	2,106	6.57 (6.30,6.86)	1.96 (1.64,2.33)	1,054	3.25 (3.06,3.45)	0.97 (0.74,1.26)
Chronic pulmonary disease	115	0.36 (0.30,0.43)	372	1.21 (1.09,1.34)	3.32 (1.93,5.70)	682	2.20 (2.04,2.37)	6.05 (3.62,10.12)	397	1.26 (1.14,1.39)	3.48 (2.32,5.24)	232	0.73 (0.64,0.83)	2.01 (1.29,3.14)
Acute respiratory distress syndrome	189	0.59 (0.51,0.68)	1,108	3.54 (3.34,3.75)	5.97 (4.17,8.56)	710	2.25 (2.09,2.42)	3.81 (2.51,5.76)	446	1.40 (1.27,1.53)	2.37 (1.65,3.41)	428	1.33 (1.21,1.46)	2.25 (1.58,3.21)
Seizure	100	0.31 (0.25,0.38)	789	2.54 (2.37,2.73)	8.11 (5.10,12.91)	568	1.81 (1.66,1.96)	5.78 (3.25,10.27)	232	0.73 (0.64,0.82)	2.33 (1.45,3.73)	298	0.92 (0.82,1.03)	2.96 (1.55,5.67)
End-stage renal disease	35	0.11 (0.08,0.15)	240	0.76 (0.67,0.86)	7.06 (3.00,16.62)	55	0.17 (0.13,0.22)	1.61 (0.39,6.64)	93	0.29 (0.24,0.36)	2.72 (1.06,6.95)	86	0.27 (0.22,0.33)	2.49 (0.66,9.38)
Acute kidney injury	87	0.27 (0.22,0.33)	824	2.63 (2.46,2.82)	9.70 (6.42,14.65)	813	2.57 (2.40,2.76)	9.50 (5.98,15.08)	312	0.98 (0.88,1.09)	3.62 (2.24,5.85)	385	1.19 (1.08,1.32)	4.41 (2.76,7.05)
Pancreatitis	56	0.18 (0.13,0.23)	241	0.76 (0.67,0.87)	4.34 (2.08,9.09)	136	0.43 (0.36,0.51)	2.45 (0.80,7.55)	145	0.45 (0.39,0.53)	2.59 (1.37,4.87)	132	0.41 (0.34,0.48)	2.33 (1.29,4.18)
All-cause mortality	5,338	16.57 (16.13,17.02)	99,525	315.57 (313.62,317.54)	18.97 (18.14,19.84)	47,276	148.90 (147.56,150.25)	8.97 (8.47,9.50)	21,029	65.63 (64.75,66.52)	3.96 (3.72,4.21)	9,377	28.93 (28.35,29.52)	1.75 (1.51,2.02)

Note: CI: confidence interval, REF: Reference group; Major CVD: Major Cardiovascular Disease; Composite outcome of stroke, heart failure and coronary artery disease

Hazard Ratio (HR) and 95% Confidence Interval (95% CI) were estimated by Cox regression, HR >1 (or <1) indicates patients with COVID-19 had higher (lower) risk of sequelae compared to the non-COVID-19 control cohort

Un-vaccinated, Incomplete vaccinated, Fully Vaccinated and Received booster dose of COVID-19 vaccines refers to patients who have received 0 dose, 1 doses, 2 doses and ≥3 doses of BioNtech or CoronaVac COVID-19 vaccines, respectively

Supplementary Table 5. Incidence rate and hazard ratio of clinical sequelae in COVID-19 and non-COVID-19 cohort after weighting during 0-30, 31-90, 91-180, 181-270,271-365 days of SARS-CoV-2 infection (Cases of SARS-COV-2 infection from the Omicron wave in Hong Kong, continued)

31-90 days

	Controls (REF)		COVID-19											
			Unvaccinated			Received single dose			Received two doses			Received booster dose(s)		
**Clinical sequelae**	**Event**	**Incidence per 1,000 person-years**	**Event**	**Incidence per 1,000 person-years**	**Hazard ratio**	**Event**	**Incidence per 1,000 person-years**	**Hazard ratio**	**Event**	**Incidence per 1,000 person-years**	**Hazard ratio**	**Event**	**Incidence per 1,000 person-years**	**Hazard ratio**
Major CVD	3,085	5.29 (5.10,5.48)	5,763	10.24 (9.97,10.50)	1.94 (1.67,2.24)	4,369	7.62 (7.39,7.85)	1.44 (1.21,1.71)	3,273	5.64 (5.45,5.83)	1.07 (0.94,1.20)	3,429	5.88 (5.68,6.08)	1.11 (0.99,1.25)
Stroke	1,754	2.89 (2.75,3.02)	2,851	4.87 (4.69,5.05)	1.69 (1.37,2.07)	2,385	4.00 (3.84,4.16)	1.38 (1.10,1.75)	1,748	2.89 (2.76,3.03)	1.00 (0.85,1.18)	1,584	2.60 (2.47,2.73)	0.90 (0.75,1.08)
Myocardial infarction	682	1.09 (1.01,1.17)	1,208	1.97 (1.86,2.09)	1.81 (1.35,2.43)	679	1.10 (1.02,1.18)	1.01 (0.65,1.56)	711	1.14 (1.06,1.23)	1.05 (0.81,1.36)	696	1.11 (1.03,1.19)	1.02 (0.78,1.32)
Heart Failure	718	1.15 (1.07,1.24)	2,294	3.77 (3.62,3.93)	3.28 (2.65,4.06)	1,216	1.98 (1.87,2.09)	1.72 (1.29,2.29)	1,022	1.65 (1.55,1.75)	1.43 (1.13,1.81)	978	1.56 (1.47,1.66)	1.36 (1.05,1.76)
Atrial fibrillation	473	0.76 (0.70,0.83)	1,108	1.83 (1.73,1.94)	2.41 (1.76,3.28)	701	1.15 (1.06,1.23)	1.51 (1.01,2.25)	629	1.02 (0.94,1.10)	1.34 (1.01,1.78)	504	0.81 (0.74,0.88)	1.06 (0.78,1.45)
Coronary Artery Disease	1,208	1.99 (1.88,2.10)	2,296	3.86 (3.70,4.02)	1.94 (1.55,2.42)	1,523	2.54 (2.41,2.66)	1.27 (0.95,1.71)	1,334	2.21 (2.09,2.33)	1.11 (0.92,1.34)	1,594	2.63 (2.50,2.76)	1.32 (1.12,1.56)
Deep vein thrombosis	117	0.19 (0.15,0.22)	451	0.73 (0.67,0.80)	3.95 (2.30,6.81)	220	0.35 (0.31,0.40)	1.91 (0.97,3.75)	236	0.38 (0.33,0.43)	2.04 (1.25,3.31)	105	0.17 (0.14,0.20)	0.90 (0.48,1.69)
Cardiovascular mortality	1,312	2.08 (1.97,2.20)	2,591	4.20 (4.04,4.37)	2.02 (1.66,2.45)	1,561	2.51 (2.38,2.63)	1.20 (0.94,1.55)	1,247	1.99 (1.88,2.10)	0.96 (0.77,1.18)	691	1.09 (1.01,1.18)	0.52 (0.38,0.72)
Chronic pulmonary disease	307	0.50 (0.44,0.55)	911	1.51 (1.42,1.61)	3.05 (2.08,4.48)	628	1.03 (0.95,1.12)	2.08 (1.26,3.44)	383	0.62 (0.56,0.69)	1.26 (0.88,1.80)	274	0.44 (0.39,0.50)	0.89 (0.60,1.33)
Acute respiratory distress syndrome	534	0.85 (0.78,0.92)	892	1.46 (1.36,1.56)	1.71 (1.18,2.48)	886	1.43 (1.34,1.53)	1.68 (1.14,2.48)	622	1.00 (0.92,1.08)	1.17 (0.88,1.56)	710	1.13 (1.05,1.22)	1.33 (0.97,1.82)
Seizure	286	0.46 (0.40,0.51)	747	1.23 (1.14,1.32)	2.70 (1.73,4.21)	533	0.86 (0.79,0.94)	1.90 (1.09,3.29)	349	0.56 (0.50,0.62)	1.22 (0.84,1.78)	196	0.31 (0.27,0.36)	0.68 (0.40,1.16)
End-stage renal disease	62	0.10 (0.08,0.13)	109	0.18 (0.15,0.21)	1.79 (0.59,5.43)	138	0.22 (0.19,0.26)	2.25 (0.86,5.90)	63	0.10 (0.08,0.13)	1.01 (0.42,2.45)	21	0.03 (0.02,0.05)	0.34 (0.10,1.09)
Acute kidney injury	202	0.32 (0.28,0.37)	762	1.24 (1.16,1.33)	3.86 (2.60,5.71)	509	0.82 (0.75,0.90)	2.55 (1.54,4.22)	251	0.40 (0.35,0.45)	1.24 (0.77,2.00)	258	0.41 (0.36,0.46)	1.27 (0.76,2.10)
Pancreatitis	145	0.23 (0.19,0.27)	483	0.78 (0.72,0.86)	3.42 (1.92,6.10)	263	0.42 (0.37,0.48)	1.85 (0.90,3.80)	153	0.24 (0.21,0.29)	1.07 (0.60,1.91)	175	0.28 (0.24,0.32)	1.21 (0.74,1.96)
All-cause mortality	7,641	12.11 (11.85,12.39)	28,350	45.96 (45.43,46.50)	3.79 (3.56,4.04)	13,384	21.49 (21.13,21.85)	1.77 (1.62,1.94)	8,501	13.55 (13.26,13.84)	1.12 (1.03,1.22)	6,713	10.62 (10.36,10.87)	0.88 (0.77,1.00)

Note: CI: confidence interval, REF: Reference group; Major CVD: Major Cardiovascular Disease; Composite outcome of stroke, heart failure and coronary artery disease

Hazard Ratio (HR) and 95% Confidence Interval (95% CI) were estimated by Cox regression, HR >1 (or <1) indicates patients with COVID-19 had higher (lower) risk of sequelae compared to the non-COVID-19 control cohort

Un-vaccinated, Incomplete vaccinated, Fully Vaccinated and Received booster dose of COVID-19 vaccines refers to patients who have received 0 dose, 1 doses, 2 doses and ≥3 doses of BioNtech or CoronaVac COVID-19 vaccines, respectively

Supplementary Table 5. Incidence rate and hazard ratio of clinical sequelae in COVID-19 and non-COVID-19 cohort after weighting during 0-30, 31-90, 91-180, 181-270,271-365 days of SARS-CoV-2 infection (Cases of SARS-COV-2 infection from the Omicron wave in Hong Kong, continued)

91-180 days

	Controls (REF)		COVID-19											
			Unvaccinated			Received single dose			Received two doses			Received booster dose(s)		
**Clinical sequelae**	**Event**	**Incidence per 1,000 person-years**	**Event**	**Incidence per 1,000 person-years**	**Hazard ratio**	**Event**	**Incidence per 1,000 person-years**	**Hazard ratio**	**Event**	**Incidence per 1,000 person-years**	**Hazard ratio**	**Event**	**Incidence per 1,000 person-years**	**Hazard ratio**
Major CVD	4,194	5.58 (5.41,5.75)	6,652	8.57 (8.37,8.78)	1.54 (1.33,1.78)	5,781	6.93 (6.75,7.11)	1.25 (1.07,1.46)	4,679	5.64 (5.48,5.81)	1.02 (0.92,1.12)	3,201	5.01 (4.84,5.19)	0.89 (0.80,0.99)
Stroke	2,433	3.10 (2.98,3.23)	3,365	4.16 (4.02,4.30)	1.34 (1.10,1.64)	3,176	3.65 (3.53,3.78)	1.18 (0.96,1.46)	2,621	3.04 (2.92,3.15)	0.98 (0.86,1.13)	1,551	2.33 (2.22,2.45)	0.74 (0.64,0.87)
Myocardial infarction	905	1.12 (1.05,1.20)	1,477	1.75 (1.66,1.84)	1.56 (1.16,2.09)	1,148	1.27 (1.20,1.35)	1.14 (0.82,1.58)	932	1.05 (0.98,1.12)	0.93 (0.74,1.17)	893	1.31 (1.22,1.39)	1.17 (0.93,1.47)
Heart Failure	825	1.03 (0.96,1.10)	2,153	2.56 (2.45,2.67)	2.51 (2.00,3.15)	1,507	1.68 (1.60,1.77)	1.65 (1.24,2.20)	1,292	1.46 (1.38,1.54)	1.43 (1.17,1.76)	713	1.05 (0.97,1.12)	1.01 (0.77,1.31)
Atrial fibrillation	670	0.84 (0.78,0.90)	1,047	1.25 (1.18,1.33)	1.50 (1.08,2.07)	1,065	1.20 (1.12,1.27)	1.43 (1.03,1.97)	950	1.08 (1.01,1.15)	1.29 (1.03,1.61)	793	1.17 (1.09,1.25)	1.40 (0.96,2.04)
Coronary Artery Disease	1,686	2.15 (2.05,2.26)	2,718	3.30 (3.18,3.43)	1.53 (1.23,1.91)	2,387	2.73 (2.62,2.84)	1.27 (1.01,1.59)	1,844	2.14 (2.04,2.24)	0.99 (0.85,1.16)	1,503	2.27 (2.16,2.39)	1.05 (0.89,1.25)
Deep vein thrombosis	148	0.18 (0.16,0.21)	269	0.32 (0.28,0.36)	1.73 (0.91,3.30)	341	0.38 (0.34,0.42)	2.06 (1.05,4.03)	98	0.11 (0.09,0.13)	0.60 (0.31,1.17)	135	0.20 (0.16,0.23)	1.08 (0.47,2.50)
Cardiovascular mortality	1,538	1.89 (1.80,1.99)	2,956	3.46 (3.34,3.59)	1.83 (1.51,2.21)	1,663	1.83 (1.75,1.92)	0.97 (0.75,1.25)	1,207	1.35 (1.27,1.43)	0.71 (0.58,0.88)	396	0.58 (0.52,0.64)	0.31 (0.21,0.45)
Chronic pulmonary disease	426	0.53 (0.49,0.59)	963	1.16 (1.09,1.23)	2.18 (1.49,3.21)	857	0.97 (0.90,1.03)	1.83 (1.19,2.82)	555	0.63 (0.58,0.69)	1.19 (0.89,1.60)	422	0.63 (0.57,0.69)	1.15 (0.79,1.69)
Acute respiratory distress syndrome	707	0.87 (0.81,0.94)	1,412	1.67 (1.58,1.76)	1.91 (1.41,2.59)	1,010	1.12 (1.05,1.19)	1.28 (0.89,1.84)	1,016	1.14 (1.07,1.21)	1.30 (1.04,1.63)	593	0.87 (0.80,0.94)	0.99 (0.70,1.41)
Seizure	386	0.48 (0.43,0.53)	1,063	1.27 (1.19,1.34)	2.67 (1.85,3.86)	929	1.03 (0.97,1.10)	2.20 (1.45,3.33)	409	0.46 (0.42,0.50)	0.97 (0.70,1.36)	299	0.44 (0.39,0.49)	0.89 (0.48,1.68)
End-stage renal disease	76	0.09 (0.07,0.12)	141	0.17 (0.14,0.19)	1.79 (0.62,5.15)	181	0.20 (0.17,0.23)	2.18 (0.83,5.67)	39	0.04 (0.03,0.06)	0.47 (0.16,1.36)	0	NA	NA
Acute kidney injury	354	0.44 (0.39,0.48)	659	0.78 (0.72,0.84)	1.79 (1.18,2.70)	495	0.55 (0.50,0.60)	1.26 (0.81,1.98)	439	0.49 (0.45,0.54)	1.13 (0.80,1.61)	144	0.21 (0.18,0.25)	0.47 (0.26,0.87)
Pancreatitis	179	0.22 (0.19,0.26)	166	0.20 (0.17,0.23)	0.89 (0.42,1.89)	184	0.20 (0.18,0.23)	0.93 (0.38,2.29)	193	0.22 (0.19,0.25)	0.98 (0.59,1.64)	103	0.15 (0.12,0.18)	0.68 (0.36,1.28)
All-cause mortality	9,882	12.15 (11.91,12.39)	22,827	26.74 (26.40,27.09)	2.20 (2.05,2.36)	12,939	14.26 (14.02,14.51)	1.18 (1.07,1.29)	8,715	9.73 (9.53,9.94)	0.80 (0.74,0.87)	5,528	8.03 (7.83,8.25)	0.66 (0.58,0.76)

Note: CI: confidence interval, REF: Reference group; Major CVD: Major Cardiovascular Disease; Composite outcome of stroke, heart failure and coronary artery disease

Hazard Ratio (HR) and 95% Confidence Interval (95% CI) were estimated by Cox regression, HR >1 (or <1) indicates patients with COVID-19 had higher (lower) risk of sequelae compared to the non-COVID-19 control cohort

Un-vaccinated, Incomplete vaccinated, Fully Vaccinated and Received booster dose of COVID-19 vaccines refers to patients who have received 0 dose, 1 doses, 2 doses and ≥3 doses of BioNtech or CoronaVac COVID-19 vaccines, respectively

Supplementary Table 5. Incidence rate and hazard ratio of clinical sequelae in COVID-19 and non-COVID-19 cohort after weighting during 0-30, 31-90, 91-180, 181-270,271-365 days of SARS-CoV-2 infection (Cases of SARS-COV-2 infection from the Omicron wave in Hong Kong, continued)

181-270 days

	Controls (REF)		COVID-19											
			Unvaccinated			Received single dose			Received two doses			Received booster dose(s)		
**Clinical sequelae**	**Event**	**Incidence per 1,000 person-years**	**Event**	**Incidence per 1,000 person-years**	**Hazard ratio**	**Event**	**Incidence per 1,000 person-years**	**Hazard ratio**	**Event**	**Incidence per 1,000 person-years**	**Hazard ratio**	**Event**	**Incidence per 1,000 person-years**	**Hazard ratio**
Major CVD	3,415	5.79 (5.60,5.98)	5,038	8.58 (8.34,8.82)	1.48 (1.27,1.72)	4,679	7.56 (7.35,7.78)	1.31 (1.12,1.52)	3,544	5.79 (5.60,5.98)	1.00 (0.90,1.11)	2,506	4.83 (4.64,5.02)	0.83 (0.71,0.98)
Stroke	1,896	3.08 (2.94,3.22)	2,921	4.77 (4.60,4.94)	1.55 (1.27,1.89)	2,259	3.50 (3.36,3.65)	1.14 (0.91,1.41)	1,918	3.01 (2.88,3.15)	0.98 (0.85,1.13)	1,299	2.39 (2.27,2.53)	0.78 (0.62,0.97)
Myocardial infarction	817	1.29 (1.20,1.38)	853	1.33 (1.24,1.42)	1.03 (0.75,1.42)	1,212	1.81 (1.71,1.92)	1.40 (1.04,1.89)	689	1.05 (0.97,1.13)	0.81 (0.64,1.04)	1,080	1.93 (1.82,2.05)	1.51 (0.60,3.79)
Heart Failure	761	1.20 (1.12,1.29)	1,942	3.05 (2.91,3.18)	2.53 (2.02,3.17)	1,489	2.24 (2.13,2.35)	1.86 (1.44,2.40)	812	1.24 (1.16,1.33)	1.03 (0.81,1.31)	679	1.21 (1.13,1.31)	1.01 (0.51,2.00)
Atrial fibrillation	535	0.85 (0.78,0.93)	943	1.49 (1.40,1.59)	1.75 (1.27,2.42)	742	1.12 (1.04,1.21)	1.32 (0.92,1.92)	782	1.20 (1.12,1.29)	1.41 (1.11,1.80)	381	0.68 (0.62,0.76)	0.80 (0.51,1.25)
Coronary Artery Disease	1,445	2.35 (2.23,2.48)	1,668	2.67 (2.55,2.80)	1.14 (0.89,1.46)	1,999	3.08 (2.95,3.22)	1.31 (1.04,1.65)	1,587	2.49 (2.37,2.62)	1.06 (0.91,1.24)	1,099	2.04 (1.92,2.16)	0.87 (0.69,1.10)
Deep vein thrombosis	109	0.17 (0.14,0.20)	116	0.18 (0.15,0.22)	1.06 (0.52,2.14)	86	0.13 (0.10,0.16)	0.75 (0.31,1.81)	136	0.21 (0.17,0.24)	1.21 (0.70,2.08)	48	0.09 (0.06,0.11)	0.50 (0.18,1.40)
Cardiovascular mortality	973	1.52 (1.43,1.62)	1,770	2.73 (2.61,2.86)	1.80 (1.43,2.25)	1,146	1.70 (1.61,1.80)	1.12 (0.85,1.48)	641	0.97 (0.90,1.05)	0.64 (0.49,0.84)	467	0.83 (0.76,0.91)	0.54 (0.31,0.92)
Chronic pulmonary disease	360	0.57 (0.52,0.63)	869	1.38 (1.29,1.47)	2.40 (1.66,3.48)	630	0.96 (0.88,1.03)	1.67 (1.11,2.52)	378	0.58 (0.53,0.64)	1.02 (0.74,1.40)	194	0.35 (0.30,0.40)	0.62 (0.37,1.01)
Acute respiratory distress syndrome	557	0.88 (0.80,0.95)	870	1.36 (1.27,1.45)	1.55 (1.08,2.23)	577	0.86 (0.79,0.93)	0.98 (0.65,1.49)	489	0.74 (0.68,0.81)	0.85 (0.64,1.12)	466	0.83 (0.76,0.91)	0.96 (0.45,2.07)
Seizure	312	0.49 (0.44,0.55)	1,095	1.72 (1.62,1.83)	3.51 (2.46,5.01)	772	1.16 (1.08,1.24)	2.36 (1.56,3.58)	265	0.40 (0.36,0.45)	0.82 (0.56,1.21)	212	0.38 (0.33,0.43)	0.77 (0.26,2.28)
End-stage renal disease	52	0.08 (0.06,0.11)	101	0.16 (0.13,0.19)	1.90 (0.77,4.70)	108	0.16 (0.13,0.19)	1.96 (0.87,4.38)	91	0.14 (0.11,0.17)	1.67 (0.83,3.36)	0	NA	NA
Acute kidney injury	300	0.47 (0.42,0.53)	420	0.65 (0.59,0.72)	1.38 (0.90,2.12)	348	0.52 (0.47,0.58)	1.10 (0.66,1.83)	270	0.41 (0.36,0.46)	0.87 (0.57,1.32)	98	0.18 (0.14,0.21)	0.37 (0.17,0.81)
Pancreatitis	172	0.27 (0.23,0.31)	237	0.37 (0.32,0.42)	1.36 (0.70,2.66)	161	0.24 (0.21,0.28)	0.89 (0.37,2.16)	147	0.22 (0.19,0.26)	0.83 (0.50,1.35)	82	0.14 (0.12,0.18)	0.54 (0.24,1.18)
All-cause mortality	7,977	12.48 (12.21,12.76)	16,415	25.36 (24.98,25.75)	2.03 (1.88,2.20)	9,602	14.26 (13.97,14.54)	1.14 (1.04,1.26)	5,567	8.41 (8.20,8.64)	0.67 (0.61,0.74)	7,956	14.09 (13.78,14.40)	1.13 (0.77,1.65)

Note: CI: confidence interval, REF: Reference group; Major CVD: Major Cardiovascular Disease; Composite outcome of stroke, heart failure and coronary artery disease

Hazard Ratio (HR) and 95% Confidence Interval (95% CI) were estimated by Cox regression, HR >1 (or <1) indicates patients with COVID-19 had higher (lower) risk of sequelae compared to the non-COVID-19 control cohort

Un-vaccinated, Incomplete vaccinated, Fully Vaccinated and Received booster dose of COVID-19 vaccines refers to patients who have received 0 dose, 1 doses, 2 doses and ≥3 doses of BioNtech or CoronaVac COVID-19 vaccines, respectively

Supplementary Table 5. Incidence rate and hazard ratio of clinical sequelae in COVID-19 and non-COVID-19 cohort after weighting during 0-30, 31-90, 91-180, 181-270,271-365 days of SARS-CoV-2 infection (Cases of SARS-COV-2 infection from the Omicron wave in Hong Kong, continued)

271-365 days

	Controls (REF)		COVID-19												
			Unvaccinated			Received single dose			Received two doses			Received booster dose(s)		
**Clinical sequelae**	**Event**	**Incidence per 1,000 person-years**	**Event**	**Incidence per 1,000 person-years**	**Hazard ratio**	**Event**	**Incidence per 1,000 person-years**	**Hazard ratio**	**Event**	**Incidence per 1,000 person-years**	**Hazard ratio**	**Event**	**Incidence per 1,000 person-years**	**Hazard ratio**
Major CVD	2,045	6.04 (5.78,6.30)	2,419	7.47 (7.18,7.77)	1.25 (1.01,1.53)	2,346	7.15 (6.87,7.45)	1.18 (0.95,1.46)	1,815	5.38 (5.14,5.63)	0.89 (0.78,1.03)	1,619	4.87 (4.64,5.12)	0.80 (0.60,1.07)
Stroke	1,072	3.04 (2.86,3.22)	1,143	3.39 (3.20,3.59)	1.12 (0.83,1.52)	925	2.71 (2.54,2.89)	0.89 (0.64,1.24)	963	2.75 (2.58,2.92)	0.91 (0.74,1.10)	998	2.85 (2.68,3.03)	0.93 (0.59,1.44)
Myocardial infarction	500	1.37 (1.26,1.50)	502	1.43 (1.31,1.56)	1.05 (0.68,1.62)	650	1.84 (1.70,1.99)	1.33 (0.89,1.99)	459	1.27 (1.16,1.39)	0.93 (0.69,1.26)	242	0.67 (0.59,0.75)	0.48 (0.30,0.77)
Heart Failure	569	1.57 (1.44,1.70)	1,089	3.11 (2.93,3.30)	2.01 (1.51,2.66)	938	2.67 (2.50,2.84)	1.69 (1.25,2.30)	654	1.82 (1.68,1.96)	1.16 (0.89,1.52)	391	1.08 (0.98,1.19)	0.68 (0.32,1.44)
Atrial fibrillation	300	0.83 (0.74,0.93)	498	1.43 (1.31,1.56)	1.73 (1.07,2.79)	410	1.17 (1.07,1.29)	1.42 (0.86,2.32)	318	0.89 (0.79,0.99)	1.07 (0.75,1.53)	562	1.56 (1.43,1.69)	1.87 (0.65,5.42)
Coronary Artery Disease	843	2.39 (2.23,2.56)	818	2.39 (2.23,2.56)	1.00 (0.70,1.42)	994	2.90 (2.72,3.08)	1.21 (0.87,1.69)	678	1.93 (1.79,2.08)	0.81 (0.64,1.03)	644	1.85 (1.71,2.00)	0.77 (0.51,1.16)
Deep vein thrombosis	57	0.15 (0.12,0.20)	143	0.40 (0.34,0.48)	2.64 (1.15,6.06)	31	0.09 (0.06,0.12)	0.56 (0.13,2.29)	29	0.08 (0.06,0.11)	0.53 (0.18,1.50)	28	0.08 (0.05,0.11)	0.48 (0.12,2.01)
Cardiovascular mortality	57	0.15 (0.12,0.20)	94	0.26 (0.21,0.32)	1.71 (0.68,4.31)	52	0.15 (0.11,0.19)	0.93 (0.28,3.07)	8	0.02 (0.01,0.04)	0.14 (0.02,0.99)	0	NA	NA
Chronic pulmonary disease	282	0.78 (0.69,0.88)	362	1.05 (0.94,1.16)	1.35 (0.79,2.30)	465	1.34 (1.22,1.46)	1.70 (1.04,2.79)	341	0.96 (0.86,1.06)	1.23 (0.85,1.76)	108	0.30 (0.25,0.36)	0.38 (0.19,0.76)
Acute respiratory distress syndrome	266	0.73 (0.64,0.82)	357	1.01 (0.91,1.12)	1.39 (0.79,2.45)	494	1.40 (1.28,1.53)	1.90 (1.23,2.93)	302	0.83 (0.74,0.93)	1.14 (0.77,1.69)	595	1.64 (1.51,1.78)	2.21 (0.63,7.75)
Seizure	176	0.48 (0.42,0.56)	270	0.77 (0.68,0.87)	1.61 (0.85,3.05)	312	0.89 (0.79,0.99)	1.82 (1.00,3.33)	142	0.39 (0.33,0.46)	0.81 (0.51,1.28)	125	0.34 (0.29,0.41)	0.70 (0.19,2.59)
End-stage renal disease	39	0.11 (0.08,0.15)	170	0.48 (0.41,0.55)	4.56 (1.90,10.91)	56	0.16 (0.12,0.20)	1.46 (0.43,4.96)	4	0.01 (0.00,0.03)	0.11 (0.01,0.83)	57	0.16 (0.12,0.20)	1.41 (0.19,10.56)
Acute kidney injury	145	0.40 (0.34,0.46)	182	0.52 (0.45,0.60)	1.31 (0.69,2.48)	92	0.26 (0.21,0.32)	0.65 (0.26,1.65)	95	0.26 (0.21,0.32)	0.66 (0.35,1.26)	419	1.15 (1.04,1.26)	2.80 (0.51,15.39)
Pancreatitis	79	0.22 (0.17,0.27)	207	0.59 (0.51,0.67)	2.72 (1.22,6.03)	117	0.33 (0.27,0.39)	1.50 (0.54,4.18)	34	0.09 (0.06,0.13)	0.43 (0.17,1.10)	19	0.05 (0.03,0.08)	0.24 (0.03,1.72)
All-cause mortality	4,495	12.27 (11.91,12.63)	9,819	27.68 (27.14,28.24)	2.27 (2.05,2.50)	4,375	12.31 (11.95,12.68)	1.00 (0.87,1.15)	3,374	9.28 (8.97,9.60)	0.76 (0.67,0.85)	1,423	3.88 (3.68,4.09)	0.31 (0.21,0.47)

Note: CI: confidence interval, REF: Reference group; Major CVD: Major Cardiovascular Disease; Composite outcome of stroke, heart failure and coronary artery disease

Hazard Ratio (HR) and 95% Confidence Interval (95% CI) were estimated by Cox regression, HR >1 (or <1) indicates patients with COVID-19 had higher (lower) risk of sequelae compared to the non-COVID-19 control cohort

Un-vaccinated, Incomplete vaccinated, Fully Vaccinated and Received booster dose of COVID-19 vaccines refers to patients who have received 0 dose, 1 doses, 2 doses and ≥3 doses of BioNtech or CoronaVac COVID-19 vaccines, respectively

Supplementary Table 6. Baseline characteristics of unvaccinated patients with COVID-19 and matched control with the same vaccination status from the Hong Kong Hospital Authority before propensity-score weighting.

Baseline characteristics	Hong Kong Hospital Authority (April 1st, 2020 to October 31st, 2022)		
	**Controls** (N=81,101)	**COVID-19** (N=124,443)	**SMD[b]**
	**N / Mean (% / SD)**	**N / Mean (% / SD)**	
Age (years)	64.8 (22.51)	60.2 (21.93)	0.208
Sex, male	33,467 (41.3)	55,091 (44.3)	0.061
Charlson Comorbidity Index	2.78 (2.34)	2.61 (2.53)	0.068
**Pre-existing morbidities**			
Respiratory disease	2,975 (3.7)	5,942 (4.8)	0.055
Diabetes	12,790 (15.8)	21,585 (17.3)	0.042
Cardiovascular disease	13,296 (16.4)	24,098 (19.4)	0.078
Myocardiac Infarction	1,349 (1.7)	3,019 (2.4)	0.054
Chronic heart failure	2,871 (3.5)	5,846 (4.7)	0.058
Peripheral vascular disease	537 (0.7)	1,185 (1.0)	0.032
Cerebrovascular disease	6,779 (8.4)	12,920 (10.4)	0.069
COPD	2,965 (3.7)	5,900 (4.7)	0.054
Dementia	1,192 (1.5)	2,795 (2.2)	0.058
Paralysis	463 (0.6)	1,105 (0.9)	0.037
Diabetes without chronic complication	12,012 (14.8)	19,919 (16.0)	0.033
Diabetes with chronic complication	1,061 (1.3)	2,523 (2.0)	0.056
Chronic Kidney Disease	2,386 (2.9)	4,957 (4.0)	0.057
Mild liver disease	167 (0.2)	523 (0.4)	0.038
Moderate-severe liver disease	126 (0.2)	396 (0.3)	0.034
Ulcers	1,314 (1.6)	2,754 (2.2)	0.043
Rheumatoid arthritis and other inflammatory polyarthropathies	411 (0.5)	912 (0.7)	0.029
Acquired Immune Deficiency Syndrome (AIDS)	0 (0.0)	0 (0.0)	<0.001
Malignancy	3,294 (4.1)	7,166 (5.8)	0.079
Metastatic solid tumour	728 (0.9)	1,698 (1.4)	0.044
**Medication history**			

Renin-angiotensin-system agents	15,154 (18.7)	27,128 (21.8)	0.078
Beta blockers	10,438 (12.9)	20,025 (16.1)	0.092
Calcium channel blockers	21,174 (26.1)	35,937 (28.9)	0.062
Diuretics	5,444 (6.7)	11,741 (9.4)	0.100
Nitrates	3,730 (4.6)	7,452 (6.0)	0.062
Lipid lowering agents	19,869 (24.5)	35,491 (28.5)	0.091
Insulins	2,226 (2.7)	5,867 (4.7)	0.104
Antidiabetic drugs	9,996 (12.3)	19,480 (15.7)	0.096
Oral anticoagulants	2,475 (3.1)	4,928 (4.0)	0.049
Antiplatelets	11,508 (14.2)	22,446 (18.0)	0.105
Immunosuppressants	406 (0.5)	1,241 (1.0)	0.058
**Number of past healthcare visits**			
Hospital admission	14,926 (18.4)	38,425 (30.9)	0.293
Doctor consultation	3 (3.87)	4 (4.83)	0.259

Supplementary Table 7. Incidence rate and hazard ratio of clinical sequelae in COVID-19 and non-COVID-19 cohort after weighting during 0-30, 31-90, 91-180, 181-270,271-365 days of SARS-CoV-2 infection (unvaccinated patients with COVID-19 and matched control with the same vaccination status)

	Day 0-30					Day 31-90				
	Non-COVID-19		COVID-19			Non-COVID-19		COVID-19		
Clinical sequelae	Event	Incidence per 1,000 person-years (95% CI)	Event	Incidence per 1,000 person-years (95% CI)	Hazard Ratio[a] (95% CI)	Event	Incidence per 1,000 person-years (95% CI)	Event	Incidence per 1,000 person-years (95% CI)	Hazard Ratio[a] (95% CI)
Major CVD	68	12.07 (9.50,15.26)	309	36.96 (32.99,41.24)	3.03 (2.29,4.01)	127	11.40 (9.58,13.56)	274	17.29 (15.31,19.41)	1.52 (1.21,1.89)
Stroke	42	6.73 (4.87,8.95)	188	20.64 (17.86,23.76)	3.02 (2.12,4.31)	72	5.91 (4.69,7.44)	147	8.51 (7.20,9.95)	1.44 (1.06,1.95)
Myocardial infarction	22	3.33 (2.17,4.96)	123	12.38 (10.37,14.75)	3.67 (2.32,5.79)	39	3.00 (2.18,4.06)	77	4.11 (3.29,5.13)	1.37 (0.91,2.06)
Heart Failure	25	3.83 (2.59,5.62)	85	8.77 (7.07,10.80)	2.27 (1.43,3.62)	54	4.18 (3.16,5.39)	143	7.81 (6.61,9.17)	1.86 (1.33,2.61)
Atrial fibrillation	3	0.53 (0.17,1.35)	44	4.53 (3.31,6.00)	8.42 (2.88,24.58)	28	2.21 (1.52,3.17)	63	3.43 (2.65,4.36)	1.55 (0.97,2.49)
Coronary Artery Disease	26	4.12 (2.81,6.02)	126	13.39 (11.22,15.90)	3.22 (2.05,5.05)	57	4.58 (3.49,5.87)	118	6.63 (5.53,7.93)	1.45 (1.03,2.04)
Deep vein thrombosis	7	0.98 (0.41,1.91)	15	1.52 (0.90,2.45)	1.54 (0.47,5.03)	8	0.57 (0.26,1.08)	20	1.07 (0.68,1.62)	1.87 (0.81,4.33)
Cardiovascular mortality	152	22.24 (18.94,26.01)	467	45.96 (41.88,50.22)	2.05 (1.67,2.51)	141	10.52 (8.88,12.36)	194	10.13 (8.76,11.61)	0.96 (0.76,1.22)
Chronic pulmonary disease	6	0.87 (0.33,1.77)	23	2.34 (1.50,3.43)	2.67 (0.80,8.91)	14	1.10 (0.65,1.82)	41	2.25 (1.65,3.04)	2.04 (1.02,4.11)
Acute respiratory distress syndrome	7	1.03 (0.42,1.93)	64	6.43 (5.02,8.18)	6.16 (2.59,14.69)	22	1.64 (1.04,2.43)	44	2.35 (1.74,3.13)	1.43 (0.84,2.42)
Seizure	6	0.85 (0.32,1.72)	32	3.23 (2.27,4.52)	3.78 (1.41,10.14)	15	1.15 (0.69,1.87)	26	1.39 (0.95,2.02)	1.21 (0.58,2.53)
End-stage renal disease	5	0.79 (0.32,1.71)	17	1.68 (1.05,2.69)	2.12 (0.78,5.75)	5	0.36 (0.12,0.77)	6	0.34 (0.15,0.68)	0.95 (0.29,3.08)
Acute kidney injury	5	0.78 (0.33,1.73)	54	5.43 (4.15,7.06)	6.97 (2.64,18.40)	18	1.36 (0.81,2.06)	47	2.49 (1.83,3.26)	1.83 (1.04,3.25)
Pancreatitis	1	0.13 (0.00,0.54)	14	1.40 (0.83,2.32)	10.36 (1.34,80.17)	2	0.16 (0.05,0.54)	18	0.92 (0.56,1.43)	5.69 (1.66,19.56)
All-cause mortality	835	121.86 (113.77,130.30)	7,445	732.67 (716.13,749.42)	5.97 (5.50,6.49)	813	60.82 (56.72,65.08)	1,950	101.90 (97.42,106.47)	1.67 (1.52,1.83)

Supplementary Table 7. Incidence rate and hazard ratio of clinical sequelae in COVID-19 and non-COVID-19 cohort after weighting during 0-30, 31-90, 91-180, 181-270,271-365 days of SARS-CoV-2 infection (unvaccinated patients with COVID-19 and matched control with the same vaccination status; continued)

	Day 91-180					Day 181-270				
	Non-COVID-19		COVID-19			Non-COVID-19		COVID-19		
**Clinical sequelae**	**Event**	**Incidence per 1,000 person-years (95% CI)**	**Event**	**Incidence per 1,000 person-years (95% CI)**	**Hazard Ratio[a] (95% CI)**	**Event**	**Incidence per 1,000 person-years (95% CI)**	**Event**	**Incidence per 1,000 person-years (95% CI)**	**Hazard Ratio[a] (95% CI)**
Major CVD	159	9.84 (8.37,11.43)	275	12.60 (11.16,14.13)	1.28 (1.04,1.56)	173	11.21 (9.65,13.00)	261	13.34 (11.78,15.01)	1.19 (0.97,1.46)
Stroke	94	5.31 (4.31,6.46)	144	6.06 (5.15,7.13)	1.14 (0.87,1.50)	105	6.24 (5.14,7.53)	154	7.24 (6.17,8.46)	1.16 (0.89,1.51)
Myocardial infarction	45	2.37 (1.73,3.12)	72	2.76 (2.18,3.46)	1.17 (0.78,1.74)	44	2.43 (1.81,3.25)	59	2.55 (1.97,3.28)	1.05 (0.70,1.58)
Heart Failure	61	3.28 (2.56,4.21)	124	4.87 (4.07,5.79)	1.48 (1.09,2.02)	75	4.22 (3.36,5.28)	130	5.70 (4.77,6.74)	1.35 (0.99,1.85)
Atrial fibrillation	36	1.93 (1.37,2.64)	58	2.30 (1.75,2.93)	1.19 (0.76,1.87)	45	2.54 (1.86,3.34)	56	2.47 (1.87,3.17)	0.97 (0.63,1.50)
Coronary Artery Disease	64	3.57 (2.75,4.50)	120	4.90 (4.08,5.84)	1.37 (0.99,1.91)	59	3.47 (2.67,4.44)	96	4.35 (3.53,5.27)	1.26 (0.89,1.77)
Deep vein thrombosis	5	0.28 (0.11,0.61)	13	0.48 (0.26,0.80)	1.75 (0.62,4.96)	7	0.41 (0.19,0.78)	9	0.38 (0.20,0.72)	0.94 (0.34,2.56)
Cardiovascular mortality	153	7.91 (6.72,9.23)	187	7.07 (6.10,8.13)	0.89 (0.71,1.12)	131	7.07 (5.93,8.35)	132	5.59 (4.70,6.61)	0.79 (0.60,1.03)
Chronic pulmonary disease	34	1.82 (1.26,2.48)	41	1.63 (1.20,2.20)	0.90 (0.55,1.46)	17	0.94 (0.55,1.45)	50	2.18 (1.64,2.86)	2.32 (1.28,4.23)
Acute respiratory distress syndrome	38	2.01 (1.45,2.73)	61	2.33 (1.79,2.97)	1.16 (0.75,1.80)	29	1.59 (1.11,2.28)	44	1.90 (1.40,2.53)	1.20 (0.73,1.98)
Seizure	21	1.10 (0.72,1.68)	42	1.62 (1.20,2.19)	1.46 (0.82,2.61)	22	1.22 (0.80,1.82)	45	1.95 (1.45,2.59)	1.59 (0.91,2.78)
End-stage renal disease	11	0.59 (0.32,1.02)	7	0.28 (0.13,0.55)	0.48 (0.18,1.29)	2	0.08 (0.01,0.30)	6	0.25 (0.09,0.49)	2.97 (0.58,15.12)
Acute kidney injury	32	1.66 (1.15,2.31)	38	1.47 (1.07,2.01)	0.88 (0.53,1.47)	35	1.90 (1.33,2.60)	37	1.57 (1.12,2.14)	0.83 (0.48,1.42)
Pancreatitis	9	0.46 (0.21,0.82)	13	0.48 (0.26,0.80)	1.04 (0.40,2.69)	2	0.16 (0.05,0.54)	17	0.71 (0.42,1.10)	1.72 (0.70,4.26)
All-cause mortality	979	50.76 (47.66,54.02)	1,427	54.04 (51.31,56.92)	1.06 (0.97,1.16)	813	60.82 (56.72,65.08)	1,192	50.31 (47.50,53.21)	0.93 (0.85,1.02)

Supplementary Table 7. Incidence rate and hazard ratio of clinical sequelae in COVID-19 and non-COVID-19 cohort after weighting during 0-30, 31-90, 91-180, 181-270,271-365 days of SARS-CoV-2 infection (unvaccinated patients with COVID-19 and matched control with the same vaccination status; continued)

	Day 271-365				
	Non-COVID-19		COVID-19		
**Clinical sequelae**	**Event**	**Incidence per 1,000 person-years (95% CI)**	**Event**	**Incidence per 1,000 person-years (95% CI)**	**Hazard Ratio[a] (95% CI)**
Major CVD	130	11.03 (9.24,13.03)	134	11.25 (9.44,13.25)	0.92 (0.71,1.19)
Stroke	71	5.59 (4.37,6.97)	68	5.28 (4.12,6.63)	0.86 (0.60,1.24)
Myocardial infarction	40	2.95 (2.12,3.95)	32	2.28 (1.58,3.18)	0.72 (0.44,1.16)
Heart Failure	58	4.37 (3.38,5.64)	75	5.53 (4.40,6.91)	1.14 (0.79,1.65)
Atrial fibrillation	18	1.38 (0.87,2.15)	27	2.02 (1.38,2.91)	1.41 (0.76,2.61)
Coronary Artery Disease	56	4.40 (3.36,5.67)	44	3.30 (2.43,4.39)	0.68 (0.45,1.03)
Deep vein thrombosis	6	0.41 (0.16,0.85)	8	0.59 (0.29,1.12)	1.30 (0.41,4.13)
Cardiovascular mortality	39	2.82 (2.02,3.81)	13	0.92 (0.54,1.58)	0.34 (0.17,0.70)
Chronic pulmonary disease	21	1.58 (1.04,2.41)	22	1.60 (1.02,2.37)	0.90 (0.48,1.72)
Acute respiratory distress syndrome	21	1.57 (1.02,2.36)	19	1.36 (0.82,2.05)	0.78 (0.40,1.50)
Seizure	6	0.41 (0.16,0.86)	14	1.00 (0.55,1.60)	2.20 (0.77,6.29)
End-stage renal disease	5	0.39 (0.16,0.85)	9	0.61 (0.29,1.12)	1.38 (0.40,4.70)
Acute kidney injury	20	1.48 (0.96,2.27)	18	1.32 (0.82,2.05)	0.81 (0.37,1.76)
Pancreatitis	6	0.45 (0.21,0.96)	10	0.68 (0.34,1.22)	1.41 (0.53,3.76)
All-cause mortality	628	45.80 (42.30,49.47)	735	52.23 (48.53,56.08)	1.04 (0.93,1.17)

Note: CI: confidence interval, REF: Reference group; Major CVD: Major Cardiovascular Disease; Composite outcome of stroke, heart failure and coronary artery disease
Hazard Ratio (HR) and 95% Confidence Interval (95% CI) were estimated by Cox regression, HR >1 (or <1) indicates patients with COVID-19 had higher (lower) risk of sequelae compared to the non-COVID-19 control cohort

Supplementary Table 8. Baseline characteristics of patients with SARS-CoV-2 infection less than 6 months after the date of last dose of vaccination stratified by vaccine status and controls from the Hong Kong Hospital Authority before propensity-score weighting.

	Hong Kong Hospital Authority (April 1st, 2020 to October 31st, 2022)					
		COVID-19				
**Baseline characteristics**	**Controls** (N= 1,958,380)	**Un-vaccinated** (N= 124,443)	**Incomplete vaccination** (N= 98,810)	**Fully vaccinated** (N= 247,162)	**Received booster dose** (N= 386,228)	**SMD**[b]
	**N / Mean (% / SD)**	**N / Mean (% / SD)**	**N / Mean (% / SD)**	**N / Mean (% / SD)**	**N / Mean (% / SD)**	
Age (years)	56.8 (18.46)	60.2 (21.93)	60.1 (19.69)	54.4 (18.51)	52.6 (16.50)	0.221
Sex, male	853,473 (43.6)	55,091 (44.3)	43,460 (44.0)	107,080 (43.3)	179,905 (46.6)	0.029
Charlson Comorbidity Index	1.89 (1.86)	2.61 (2.53)	2.41 (2.20)	1.78 (1.89)	1.42 (1.62)	0.296
**Pre-existing morbidities**						
Respiratory disease	39,696 (2.0)	5,942 (4.8)	3,711 (3.8)	6,008 (2.4)	6,537 (1.7)	0.091
Diabetes	249,200 (12.7)	21,585 (17.3)	18,226 (18.4)	36,897 (14.9)	41,022 (10.6)	0.115
Cardiovascular disease	167,816 (8.6)	24,098 (19.4)	14,549 (14.7)	22,393 (9.1)	23,196 (6.0)	0.204
Myocardiac Infarction	21,448 (1.1)	5,846 (4.7)	2,585 (2.6)	3,098 (1.3)	2,240 (0.6)	0.131
Chronic heart failure	5,789 (0.3)	1,185 (1.0)	562 (0.6)	718 (0.3)	667 (0.2)	0.052
Peripheral vascular disease	78,085 (4.0)	12,920 (10.4)	7,333 (7.4)	10,268 (4.2)	9,475 (2.5)	0.163
Cerebrovascular disease	39,568 (2.0)	5,900 (4.7)	3,694 (3.7)	5,995 (2.4)	6,528 (1.7)	0.091
COPD	6,585 (0.3)	2,795 (2.2)	1,352 (1.4)	1,191 (0.5)	552 (0.1)	0.105
Dementia	4,438 (0.2)	1,105 (0.9)	479 (0.5)	576 (0.2)	454 (0.1)	0.054
Paralysis	238,406 (12.2)	19,919 (16.0)	17,245 (17.5)	35,300 (14.3)	39,489 (10.2)	0.106
Diabetes without chronic complication	15,767 (0.8)	2,523 (2.0)	1,465 (1.5)	2,373 (1.0)	2,255 (0.6)	0.065
Diabetes with chronic complication	25,299 (1.3)	4,957 (4.0)	2,712 (2.7)	3,627 (1.5)	3,375 (0.9)	0.104
Chronic Kidney Disease	2,885 (0.1)	523 (0.4)	252 (0.3)	468 (0.2)	422 (0.1)	0.030
Mild liver disease	1,899 (0.1)	396 (0.3)	166 (0.2)	345 (0.1)	269 (0.1)	0.027
Moderate-severe liver disease	21,041 (1.1)	2,754 (2.2)	1,695 (1.7)	2,965 (1.2)	3,304 (0.9)	0.056
Ulcers	8,174 (0.4)	912 (0.7)	616 (0.6)	1,342 (0.5)	1,512 (0.4)	0.024
Rheumatoid arthritis and other inflammatory polyarthropathies	4 (0.0)	0 (0.0)	0 (0.0)	0 (0.0)	0 (0.0)	0.001

Acquired Immune Deficiency Syndrome (AIDS)	59,887 (3.1)	7,166 (5.8)	4,914 (5.0)	8,757 (3.5)	9,048 (2.3)	0.090
Malignancy	9,402 (0.5)	1,698 (1.4)	923 (0.9)	1,372 (0.6)	1,144 (0.3)	0.059
Metastatic solid tumour	21,448 (1.1)	5,846 (4.7)	2,585 (2.6)	3,098 (1.3)	2,240 (0.6)	0.131
**Medication history**						
Renin-angiotensin-system agents	303,826 (15.5)	27,128 (21.8)	22,485 (22.8)	44,418 (18.0)	51,359 (13.3)	0.132
Beta blockers	192,039 (9.8)	20,025 (16.1)	15,468 (15.7)	28,406 (11.5)	29,634 (7.7)	0.140
Calcium channel blockers	431,715 (22.0)	35,937 (28.9)	30,883 (31.3)	61,354 (24.8)	71,781 (18.6)	0.150
Diuretics	65,281 (3.3)	11,741 (9.4)	6,499 (6.6)	9,417 (3.8)	9,125 (2.4)	0.154
Nitrates	47,598 (2.4)	7,452 (6.0)	4,424 (4.5)	6,651 (2.7)	7,138 (1.8)	0.110
Lipid lowering agents	442,712 (22.6)	35,491 (28.5)	30,616 (31.0)	61,557 (24.9)	75,646 (19.6)	0.133
Insulins	35,073 (1.8)	5,867 (4.7)	3,400 (3.4)	5,602 (2.3)	5,177 (1.3)	0.101
Antidiabetic drugs	229,320 (11.7)	19,480 (15.7)	17,472 (17.7)	35,971 (14.6)	39,816 (10.3)	0.109
Oral anticoagulants	26,609 (1.4)	4,928 (4.0)	2,760 (2.8)	3,626 (1.5)	3,391 (0.9)	0.103
Antiplatelets	173,270 (8.8)	22,446 (18.0)	14,887 (15.1)	24,178 (9.8)	25,743 (6.7)	0.180
Immunosuppressants	8,568 (0.4)	1,241 (1.0)	806 (0.8)	1,550 (0.6)	1,899 (0.5)	0.035
**Number of past healthcare visits**						
Hospital admission	0 (2.0)	1 (5.3)	1 (4.7)	0 (3.6)	0 (2.8)	0.121
Doctor consultation	2.8 (3.8)	3.8 (4.8)	4.1 (4.6)	3.5 (4.3)	2.8 (3.7)	0.168
**Doses of COVID-19 vaccines received**						
0	342,110 (17.5)	124,443 (100.0)	0 (0.0)	0 (0.0)	0 (0.0)	
1	156,258 (8.0)	0 (0.0)	98,810 (100.0)	0 (0.0)	0 (0.0)	
2	672,157 (34.3)	0 (0.0)	0 (0.0)	247,162 (100.0)	0 (0.0)	
3 or above	787,855 (40.2)	0 (0.0)	0 (0.0)	0 (0.0)	386,228 (100.0)	

Supplementary Table 9. Incidence rate and hazard ratio of clinical sequelae in COVID-19 and non-COVID-19 cohort after weighting during 0-30, 31-90, 91-180, 181-270,271-365 days of SARS-CoV-2 infection (Excluding patients with incident SARS-CoV-2 infection over 6 months after the last dose of COVID-19 vaccination)

0-30 days

	Controls (REF)		COVID-19											
			Unvaccinated			Received single dose			Received two doses			Received booster dose(s)		
**Clinical sequelae**	**Event**	**Incidence per 1,000 person-years**	**Event**	**Incidence per 1,000 person-years**	**Hazard ratio**	**Event**	**Incidence per 1,000 person-years**	**Hazard ratio**	**Event**	**Incidence per 1,000 person-years**	**Hazard ratio**	**Event**	**Incidence per 1,000 person-years**	**Hazard ratio**
Major CVD	1,068	4.90 (4.61,5.20)	5,103	24.44 (23.78,25.12)	4.96 (4.27,5.76)	3,435	16.11 (15.58,16.65)	3.28 (2.72,3.95)	2,935	13.58 (13.09,14.08)	2.77 (2.37,3.23)	2,272	10.41 (9.99,10.84)	2.12 (1.81,2.49)
Stroke	680	2.97 (2.75,3.20)	3,091	14.14 (13.65,14.64)	4.72 (3.89,5.74)	2,098	9.39 (8.99,9.80)	3.15 (2.49,3.99)	2,043	9.00 (8.62,9.40)	3.03 (2.51,3.66)	1,469	6.39 (6.06,6.72)	2.15 (1.73,2.67)
Myocardial infarction	239	1.01 (0.88,1.14)	1,672	7.28 (6.93,7.63)	7.17 (5.54,9.29)	1,336	5.73 (5.43,6.05)	5.68 (4.16,7.75)	874	3.71 (3.47,3.96)	3.69 (2.74,4.95)	868	3.64 (3.40,3.89)	3.62 (2.76,4.75)
Heart Failure	247	1.05 (0.92,1.18)	1,177	5.16 (4.88,5.47)	4.92 (3.68,6.57)	803	3.46 (3.23,3.71)	3.30 (2.29,4.76)	530	2.26 (2.08,2.46)	2.16 (1.53,3.05)	323	1.36 (1.22,1.51)	1.30 (0.85,1.98)
Atrial fibrillation	135	0.58 (0.49,0.68)	597	2.64 (2.43,2.86)	4.56 (3.03,6.87)	512	2.23 (2.04,2.42)	3.86 (2.44,6.10)	351	1.51 (1.36,1.67)	2.61 (1.68,4.05)	407	1.72 (1.56,1.90)	2.99 (2.04,4.38)
Coronary Artery Disease	385	1.68 (1.52,1.86)	1,842	8.27 (7.90,8.65)	4.89 (3.84,6.21)	1,427	6.33 (6.01,6.67)	3.76 (2.82,5.01)	1,118	4.93 (4.64,5.22)	2.93 (2.27,3.77)	992	4.33 (4.07,4.61)	2.58 (2.03,3.27)
Deep vein thrombosis	38	0.16 (0.12,0.22)	238	1.03 (0.91,1.17)	6.38 (3.10,13.15)	238	1.01 (0.89,1.15)	6.29 (2.94,13.46)	70	0.30 (0.23,0.37)	1.85 (0.71,4.81)	62	0.26 (0.20,0.33)	1.60 (0.63,4.04)
Cardiovascular mortality	1,025	4.28 (4.03,4.55)	5,641	24.32 (23.69,24.96)	5.65 (4.92,6.48)	3,003	12.77 (12.32,13.24)	2.97 (2.47,3.58)	1,894	7.98 (7.63,8.35)	1.86 (1.54,2.25)	1,024	4.26 (4.00,4.52)	0.99 (0.75,1.32)
Chronic pulmonary disease	87	0.37 (0.30,0.45)	320	1.42 (1.27,1.58)	3.82 (2.21,6.61)	554	2.42 (2.22,2.63)	6.53 (3.90,10.92)	354	1.53 (1.38,1.70)	4.13 (2.55,6.70)	200	0.85 (0.74,0.97)	2.30 (1.41,3.74)
Acute respiratory distress syndrome	160	0.67 (0.58,0.79)	1,155	5.03 (4.74,5.32)	7.42 (5.30,10.37)	630	2.70 (2.49,2.91)	3.99 (2.62,6.09)	445	1.89 (1.72,2.07)	2.80 (1.90,4.13)	340	1.42 (1.28,1.58)	2.11 (1.41,3.16)
Seizure	75	0.32 (0.25,0.40)	586	2.57 (2.36,2.78)	8.07 (5.04,12.94)	441	1.89 (1.72,2.08)	5.98 (3.36,10.65)	241	1.02 (0.90,1.16)	3.24 (1.90,5.54)	242	1.01 (0.89,1.15)	3.21 (1.55,6.61)
End-stage renal disease	38	0.16 (0.11,0.21)	217	0.94 (0.82,1.07)	5.88 (2.11,16.37)	39	0.16 (0.12,0.22)	1.03 (0.17,6.11)	67	0.28 (0.22,0.36)	1.78 (0.55,5.83)	83	0.34 (0.28,0.42)	2.18 (0.51,9.24)
Acute kidney injury	74	0.31 (0.25,0.39)	721	3.14 (2.92,3.37)	10.02 (6.55,15.33)	670	2.87 (2.66,3.09)	9.18 (5.72,14.72)	301	1.28 (1.14,1.43)	4.09 (2.48,6.74)	357	1.49 (1.34,1.65)	4.78 (2.93,7.80)
Pancreatitis	51	0.21 (0.16,0.28)	229	0.99 (0.87,1.13)	4.62 (2.29,9.32)	111	0.47 (0.39,0.57)	2.20 (0.73,6.65)	116	0.49 (0.41,0.59)	2.29 (1.08,4.88)	87	0.36 (0.29,0.44)	1.68 (0.82,3.46)
All-cause mortality	4,989	20.84 (20.27,21.43)	90,249	389.18 (386.64,391.72)	18.59 (17.73,19.50)	42,585	181.10 (179.39,182.83)	8.67 (8.17,9.21)	19,044	80.22 (79.09,81.37)	3.85 (3.59,4.11)	9,002	37.41 (36.65,38.19)	1.79 (1.53,2.10)

Note: CI: confidence interval, REF: Reference group; Major CVD: Major Cardiovascular Disease; Composite outcome of stroke, heart failure and coronary artery disease
Hazard Ratio (HR) and 95% Confidence Interval (95% CI) were estimated by Cox regression, HR >1 (or <1) indicates patients with COVID-19 had higher (lower) risk of sequelae compared to the non-COVID-19 control cohort
Un-vaccinated, Incomplete vaccinated, Fully Vaccinated and Received booster dose of COVID-19 vaccines refers to patients who have received 0 dose, 1 doses, 2 doses and ≥3 doses of BioNtech or CoronaVac COVID-19 vaccines, respectively

Supplementary Table 9. Incidence rate and hazard ratio of clinical sequelae in COVID-19 and non-COVID-19 cohort after weighting during 0-30, 31-90, 91-180, 181-270,271-365 days of SARS-CoV-2 infection (Excluding patients with incident SARS-CoV-2 infection over 6 months after the last dose of COVID-19 vaccination; continued)

31-90 days

	Controls (REF)		COVID-19												
			Unvaccinated			Incompletely vaccinated			Fully vaccinated			Received booster dose		
Clinical sequelae	Event	Incidence per 1,000 person-years	Event	Incidence per 1,000 person-years	Hazard ratio	Event	Incidence per 1,000 person-years	Hazard ratio	Event	Incidence per 1,000 person-years	Hazard ratio	Event	Incidence per 1,000 person-years	Hazard ratio
Major CVD	2,542	5.95 (5.72,6.18)	4,954	12.12 (11.79,12.46)	2.04 (1.77,2.35)	3,665	8.75 (8.47,9.04)	1.47 (1.24,1.75)	2,701	6.38 (6.14,6.62)	1.07 (0.93,1.24)	2,865	6.72 (6.48,6.97)	1.13 (0.99,1.29)
Stroke	1,453	3.24 (3.08,3.41)	2,454	5.73 (5.50,5.96)	1.77 (1.45,2.16)	1,991	4.54 (4.34,4.74)	1.40 (1.11,1.77)	1,418	3.19 (3.03,3.36)	0.98 (0.81,1.20)	1,394	3.10 (2.94,3.27)	0.96 (0.78,1.18)
Myocardial infarction	573	1.23 (1.14,1.34)	1,027	2.28 (2.14,2.42)	1.84 (1.39,2.45)	562	1.23 (1.13,1.33)	1.00 (0.64,1.55)	580	1.26 (1.16,1.37)	1.02 (0.75,1.39)	584	1.26 (1.16,1.36)	1.02 (0.76,1.36)
Heart Failure	643	1.39 (1.29,1.50)	2,039	4.56 (4.36,4.76)	3.28 (2.65,4.05)	1,118	2.46 (2.31,2.60)	1.77 (1.33,2.35)	889	1.94 (1.82,2.07)	1.39 (1.08,1.81)	889	1.92 (1.80,2.05)	1.38 (1.05,1.82)
Atrial fibrillation	415	0.90 (0.82,0.99)	977	2.20 (2.07,2.34)	2.44 (1.81,3.30)	619	1.37 (1.27,1.48)	1.52 (1.02,2.26)	596	1.31 (1.21,1.42)	1.45 (1.06,1.98)	401	0.87 (0.79,0.96)	0.97 (0.67,1.38)
Coronary Artery Disease	989	2.21 (2.08,2.35)	1,976	4.52 (4.33,4.73)	2.05 (1.65,2.54)	1,243	2.81 (2.66,2.97)	1.27 (0.95,1.71)	1,070	2.41 (2.27,2.56)	1.09 (0.87,1.37)	1,236	2.77 (2.62,2.93)	1.25 (1.03,1.52)
Deep vein thrombosis	97	0.21 (0.17,0.25)	370	0.81 (0.73,0.90)	3.91 (2.28,6.72)	197	0.43 (0.37,0.49)	2.05 (1.04,4.05)	204	0.44 (0.38,0.50)	2.12 (1.23,3.64)	86	0.18 (0.15,0.23)	0.89 (0.44,1.78)
Cardiovascular mortality	1,225	2.62 (2.47,2.77)	2,346	5.15 (4.95,5.37)	1.97 (1.62,2.39)	1,416	3.07 (2.91,3.23)	1.17 (0.91,1.51)	1,097	2.36 (2.23,2.51)	0.90 (0.71,1.14)	686	1.46 (1.36,1.57)	0.56 (0.41,0.77)
Chronic pulmonary disease	258	0.56 (0.50,0.63)	737	1.67 (1.55,1.79)	2.96 (2.05,4.28)	471	1.05 (0.96,1.14)	1.86 (1.12,3.09)	394	0.87 (0.79,0.96)	1.55 (1.05,2.29)	219	0.48 (0.42,0.55)	0.85 (0.55,1.33)
Acute respiratory distress syndrome	452	0.97 (0.89,1.06)	791	1.75 (1.64,1.88)	1.81 (1.27,2.56)	742	1.62 (1.51,1.74)	1.67 (1.13,2.46)	494	1.07 (0.98,1.17)	1.10 (0.79,1.54)	623	1.34 (1.24,1.45)	1.38 (0.98,1.93)
Seizure	235	0.50 (0.44,0.57)	558	1.25 (1.15,1.35)	2.47 (1.59,3.84)	414	0.91 (0.82,1.00)	1.80 (1.03,3.13)	349	0.76 (0.68,0.84)	1.50 (0.99,2.28)	152	0.33 (0.28,0.38)	0.65 (0.35,1.20)
End-stage renal disease	51	0.11 (0.08,0.14)	81	0.18 (0.14,0.22)	1.63 (0.62,4.25)	117	0.25 (0.21,0.30)	2.34 (0.88,6.19)	52	0.11 (0.09,0.15)	1.03 (0.38,2.77)	20	0.04 (0.03,0.06)	0.39 (0.12,1.28)
Acute kidney injury	185	0.40 (0.34,0.46)	670	1.49 (1.38,1.60)	3.74 (2.53,5.54)	432	0.94 (0.86,1.04)	2.38 (1.43,3.95)	214	0.46 (0.40,0.53)	1.17 (0.70,1.95)	247	0.53 (0.47,0.60)	1.33 (0.79,2.25)
Pancreatitis	108	0.23 (0.19,0.28)	378	0.83 (0.75,0.92)	3.60 (2.05,6.32)	216	0.47 (0.41,0.54)	2.03 (1.01,4.09)	171	0.37 (0.32,0.43)	1.59 (0.87,2.92)	144	0.31 (0.26,0.36)	1.33 (0.78,2.28)
All-cause mortality	7,042	15.05 (14.70,15.40)	25,779	56.64 (55.95,57.34)	3.76 (3.53,4.00)	11,942	25.88 (25.42,26.34)	1.72 (1.57,1.88)	7,513	16.18 (15.81,16.54)	1.07 (0.98,1.18)	6,171	13.16 (12.83,13.49)	0.87 (0.76,1.00)

Note: CI: confidence interval, REF: Reference group; Major CVD: Major Cardiovascular Disease; Composite outcome of stroke, heart failure and coronary artery disease

Hazard Ratio (HR) and 95% Confidence Interval (95% CI) were estimated by Cox regression, HR >1 (or <1) indicates patients with COVID-19 had higher (lower) risk of sequelae compared to the non-COVID-19 control cohort

Un-vaccinated, Incomplete vaccinated, Fully Vaccinated and Received booster dose of COVID-19 vaccines refers to patients who have received 0 dose, 1 doses, 2 doses and ≥3 doses of BioNtech or CoronaVac COVID-19 vaccines, respectively

Supplementary Table 9. Incidence rate and hazard ratio of clinical sequelae in COVID-19 and non-COVID-19 cohort after weighting during 0-30, 31-90, 91-180, 181-270,271-365 days of SARS-CoV-2 infection (Excluding patients with incident SARS-CoV-2 infection over 6 months after the last dose of COVID-19 vaccination; continued)

91-180 days

	Controls (REF)		COVID-19											
			Unvaccinated			Incompletely vaccinated			Fully vaccinated			Received booster dose		
**Clinical sequelae**	**Event**	**Incidence per 1,000 person-years**	**Event**	**Incidence per 1,000 person-years**	**Hazard ratio**	**Event**	**Incidence per 1,000 person-years**	**Hazard ratio**	**Event**	**Incidence per 1,000 person-years**	**Hazard ratio**	**Event**	**Incidence per 1,000 person-years**	**Hazard ratio**
Major CVD	3,549	6.26 (6.05,6.47)	5,416	9.43 (9.19,9.69)	1.51 (1.32,1.74)	4,774	7.76 (7.55,7.99)	1.25 (1.07,1.45)	4,025	6.70 (6.49,6.91)	1.07 (0.95,1.21)	2,881	5.59 (5.39,5.80)	0.89 (0.79,1.00)
Stroke	2,067	3.47 (3.33,3.63)	2,728	4.53 (4.36,4.70)	1.31 (1.08,1.58)	2,657	4.12 (3.97,4.28)	1.19 (0.97,1.47)	2,238	3.55 (3.41,3.70)	1.02 (0.87,1.20)	1,422	2.64 (2.50,2.78)	0.76 (0.64,0.89)
Myocardial infarction	781	1.27 (1.18,1.36)	1,275	2.01 (1.90,2.12)	1.58 (1.19,2.11)	982	1.46 (1.37,1.55)	1.15 (0.83,1.59)	767	1.17 (1.09,1.26)	0.93 (0.70,1.22)	801	1.44 (1.34,1.54)	1.14 (0.89,1.46)
Heart Failure	744	1.21 (1.13,1.30)	1,865	2.96 (2.83,3.10)	2.45 (1.97,3.06)	1,332	1.99 (1.89,2.10)	1.66 (1.25,2.19)	1,165	1.79 (1.69,1.90)	1.49 (1.19,1.86)	694	1.25 (1.16,1.35)	1.03 (0.78,1.35)
Atrial fibrillation	614	1.01 (0.93,1.09)	927	1.48 (1.39,1.58)	1.47 (1.07,2.02)	876	1.32 (1.24,1.41)	1.31 (0.94,1.81)	870	1.35 (1.26,1.44)	1.33 (1.03,1.72)	723	1.31 (1.22,1.41)	1.31 (0.87,1.96)
Coronary Artery Disease	1,423	2.39 (2.27,2.52)	2,237	3.64 (3.49,3.79)	1.52 (1.23,1.88)	1,947	3.00 (2.87,3.13)	1.25 (1.00,1.57)	1,543	2.45 (2.33,2.57)	1.02 (0.85,1.24)	1,317	2.46 (2.33,2.59)	1.02 (0.85,1.23)
Deep vein thrombosis	133	0.22 (0.18,0.25)	231	0.36 (0.32,0.41)	1.68 (0.88,3.20)	279	0.41 (0.37,0.46)	1.91 (0.98,3.73)	93	0.14 (0.11,0.17)	0.65 (0.31,1.37)	120	0.21 (0.18,0.25)	1.00 (0.40,2.48)
Cardiovascular mortality	1,419	2.28 (2.17,2.40)	2,551	3.98 (3.82,4.13)	1.74 (1.44,2.10)	1,480	2.18 (2.07,2.30)	0.95 (0.74,1.23)	1,052	1.60 (1.50,1.70)	0.70 (0.55,0.88)	387	0.69 (0.63,0.76)	0.30 (0.21,0.45)
Chronic pulmonary disease	375	0.62 (0.56,0.68)	791	1.27 (1.18,1.36)	2.07 (1.43,3.00)	683	1.03 (0.96,1.11)	1.70 (1.11,2.59)	515	0.80 (0.73,0.87)	1.31 (0.94,1.82)	371	0.68 (0.61,0.75)	1.09 (0.72,1.65)
Acute respiratory distress syndrome	626	1.01 (0.94,1.09)	1,200	1.89 (1.78,2.00)	1.87 (1.39,2.50)	840	1.25 (1.17,1.33)	1.23 (0.86,1.77)	923	1.41 (1.32,1.50)	1.39 (1.08,1.79)	550	0.99 (0.91,1.07)	0.98 (0.68,1.40)
Seizure	319	0.52 (0.46,0.58)	844	1.34 (1.25,1.43)	2.61 (1.82,3.74)	738	1.10 (1.02,1.18)	2.16 (1.44,3.25)	341	0.52 (0.47,0.58)	1.02 (0.68,1.52)	279	0.50 (0.44,0.56)	0.95 (0.50,1.81)
End-stage renal disease	71	0.11 (0.09,0.14)	109	0.17 (0.14,0.21)	1.49 (0.56,3.99)	147	0.22 (0.18,0.26)	1.92 (0.75,4.93)	17	0.03 (0.02,0.04)	0.22 (0.05,0.93)	0	NA	NA
Acute kidney injury	314	0.51 (0.45,0.57)	567	0.89 (0.82,0.97)	1.76 (1.17,2.65)	448	0.66 (0.61,0.73)	1.32 (0.84,2.06)	390	0.60 (0.54,0.66)	1.18 (0.81,1.71)	110	0.20 (0.16,0.24)	0.39 (0.20,0.73)
Pancreatitis	150	0.24 (0.21,0.28)	173	0.27 (0.23,0.31)	1.12 (0.55,2.27)	154	0.23 (0.19,0.26)	0.94 (0.39,2.24)	174	0.26 (0.23,0.31)	1.09 (0.61,1.97)	86	0.15 (0.12,0.19)	0.63 (0.31,1.28)
All-cause mortality	9,122	14.68 (14.38,14.98)	19,759	30.81 (30.38,31.24)	2.10 (1.96,2.25)	11,570	17.06 (16.76,17.38)	1.16 (1.06,1.28)	7,633	11.59 (11.33,11.85)	0.79 (0.72,0.86)	5,185	9.26 (9.01,9.51)	0.63 (0.55,0.72)

Note: CI: confidence interval, REF: Reference group; Major CVD: Major Cardiovascular Disease; Composite outcome of stroke, heart failure and coronary artery disease
Hazard Ratio (HR) and 95% Confidence Interval (95% CI) were estimated by Cox regression, HR >1 (or <1) indicates patients with COVID-19 had higher (lower) risk of sequelae compared to the non-COVID-19 control cohort
Un-vaccinated, Incomplete vaccinated, Fully Vaccinated and Received booster dose of COVID-19 vaccines refers to patients who have received 0 dose, 1 doses, 2 doses and ≥3 doses of BioNtech or CoronaVac COVID-19 vaccines, respectively

Supplementary Table 9. Incidence rate and hazard ratio of clinical sequelae in COVID-19 and non-COVID-19 cohort after weighting during 0-30, 31-90, 91-180, 181-270,271-365 days of SARS-CoV-2 infection (Excluding patients with incident SARS-CoV-2 infection over 6 months after the last dose of COVID-19 vaccination; continued)

181-270 days

	Controls (REF)		COVID-19											
			Unvaccinated			Incompletely vaccinated			Fully vaccinated			Received booster dose		
**Clinical sequelae**	**Event**	**Incidence per 1,000 person-years**	**Event**	**Incidence per 1,000 person-years**	**Hazard ratio**	**Event**	**Incidence per 1,000 person-years**	**Hazard ratio**	**Event**	**Incidence per 1,000 person-years**	**Hazard ratio**	**Event**	**Incidence per 1,000 person-years**	**Hazard ratio**
Major CVD	2,959	6.69 (6.45,6.93)	4,413	9.85 (9.57,10.15)	1.47 (1.27,1.71)	4,113	8.76 (8.50,9.04)	1.31 (1.12,1.53)	3,193	7.01 (6.77,7.26)	1.05 (0.92,1.19)	2,138	5.23 (5.02,5.46)	0.78 (0.66,0.93)
Stroke	1,660	3.57 (3.40,3.74)	2,517	5.35 (5.14,5.56)	1.50 (1.23,1.82)	2,004	4.07 (3.89,4.25)	1.14 (0.92,1.42)	1,774	3.71 (3.54,3.89)	1.04 (0.88,1.23)	1,113	2.58 (2.43,2.73)	0.72 (0.57,0.91)
Myocardial infarction	695	1.44 (1.34,1.55)	815	1.64 (1.53,1.76)	1.13 (0.83,1.54)	1,067	2.08 (1.95,2.20)	1.43 (1.07,1.92)	584	1.18 (1.09,1.28)	0.82 (0.61,1.09)	1,379	3.08 (2.92,3.25)	2.16 (0.62,7.45)
Heart Failure	722	1.50 (1.40,1.62)	1,791	3.64 (3.47,3.81)	2.42 (1.94,3.02)	1,369	2.68 (2.54,2.82)	1.78 (1.38,2.30)	754	1.53 (1.42,1.64)	1.02 (0.78,1.32)	648	1.45 (1.34,1.56)	0.96 (0.46,2.00)
Atrial fibrillation	503	1.05 (0.96,1.15)	888	1.82 (1.70,1.94)	1.73 (1.27,2.36)	664	1.31 (1.21,1.41)	1.25 (0.86,1.81)	842	1.72 (1.60,1.84)	1.63 (1.27,2.11)	340	0.77 (0.69,0.85)	0.72 (0.46,1.14)
Coronary Artery Disease	1,222	2.63 (2.49,2.78)	1,501	3.12 (2.97,3.28)	1.19 (0.94,1.50)	1,732	3.49 (3.33,3.66)	1.33 (1.05,1.67)	1,307	2.74 (2.59,2.89)	1.04 (0.86,1.26)	926	2.16 (2.02,2.30)	0.82 (0.64,1.05)
Deep vein thrombosis	93	0.19 (0.16,0.23)	115	0.23 (0.19,0.28)	1.20 (0.59,2.43)	83	0.16 (0.13,0.20)	0.84 (0.34,2.04)	122	0.24 (0.21,0.29)	1.28 (0.67,2.43)	41	0.09 (0.07,0.12)	0.47 (0.16,1.33)
Cardiovascular mortality	907	1.87 (1.75,1.99)	1,660	3.31 (3.16,3.47)	1.78 (1.43,2.21)	1,069	2.06 (1.94,2.19)	1.11 (0.84,1.46)	526	1.05 (0.97,1.15)	0.57 (0.41,0.77)	444	0.98 (0.89,1.08)	0.52 (0.29,0.92)
Chronic pulmonary disease	299	0.63 (0.56,0.70)	728	1.49 (1.39,1.61)	2.38 (1.66,3.42)	561	1.11 (1.02,1.21)	1.77 (1.18,2.66)	400	0.82 (0.74,0.90)	1.31 (0.90,1.89)	160	0.36 (0.31,0.42)	0.58 (0.35,0.97)
Acute respiratory distress syndrome	481	0.99 (0.91,1.09)	753	1.51 (1.41,1.63)	1.52 (1.07,2.16)	468	0.91 (0.83,0.99)	0.91 (0.60,1.39)	503	1.01 (0.93,1.11)	1.02 (0.74,1.39)	405	0.90 (0.82,1.00)	0.92 (0.40,2.13)
Seizure	272	0.56 (0.50,0.63)	822	1.67 (1.55,1.78)	2.96 (2.08,4.21)	578	1.13 (1.04,1.22)	2.00 (1.32,3.05)	238	0.48 (0.42,0.54)	0.85 (0.54,1.35)	185	0.41 (0.35,0.47)	0.73 (0.23,2.35)
End-stage renal disease	46	0.09 (0.07,0.12)	85	0.17 (0.14,0.21)	1.80 (0.72,4.46)	98	0.19 (0.16,0.23)	2.00 (0.89,4.49)	101	0.20 (0.16,0.24)	2.13 (1.00,4.53)	0	NA	NA
Acute kidney injury	271	0.56 (0.50,0.63)	428	0.86 (0.78,0.94)	1.54 (1.02,2.33)	323	0.63 (0.56,0.70)	1.12 (0.67,1.87)	252	0.51 (0.45,0.57)	0.90 (0.56,1.45)	90	0.20 (0.16,0.24)	0.35 (0.16,0.78)
Pancreatitis	144	0.30 (0.25,0.35)	250	0.50 (0.44,0.57)	1.69 (0.93,3.07)	133	0.26 (0.22,0.30)	0.87 (0.36,2.06)	118	0.24 (0.20,0.28)	0.80 (0.42,1.52)	70	0.15 (0.12,0.19)	0.51 (0.23,1.17)
All-cause mortality	7,415	15.25 (14.90,15.60)	15,111	30.14 (29.66,30.62)	1.98 (1.83,2.13)	8,955	17.27 (16.92,17.63)	1.13 (1.03,1.25)	4,781	9.58 (9.31,9.85)	0.63 (0.57,0.70)	8,017	17.69 (17.31,18.08)	1.16 (0.77,1.73)

Note: CI: confidence interval, REF: Reference group; Major CVD: Major Cardiovascular Disease; Composite outcome of stroke, heart failure and coronary artery disease
Hazard Ratio (HR) and 95% Confidence Interval (95% CI) were estimated by Cox regression, HR >1 (or <1) indicates patients with COVID-19 had higher (lower) risk of sequelae compared to the non-COVID-19 control cohort
Un-vaccinated, Incomplete vaccinated, Fully Vaccinated and Received booster dose of COVID-19 vaccines refers to patients who have received 0 dose, 1 doses, 2 doses and ≥3 doses of BioNtech or CoronaVac COVID-19 vaccines, respectively

Supplementary Table 9. Incidence rate and hazard ratio of clinical sequelae in COVID-19 and non-COVID-19 cohort after weighting during 0-30, 31-90, 91-180, 181-270,271-365 days of SARS-CoV-2 infection (Excluding patients with incident SARS-CoV-2 infection over 6 months after the last dose of COVID-19 vaccination; continued)

271-365 days

	Controls (REF)		COVID-19												
			Unvaccinated			Incompletely vaccinated			Fully vaccinated			Received booster dose		
Clinical sequelae	Event	Incidence per 1,000 person-years	Event	Incidence per 1,000 person-years	Hazard ratio	Event	Incidence per 1,000 person-years	Hazard ratio	Event	Incidence per 1,000 person-years	Hazard ratio	Event	Incidence per 1,000 person-years	Hazard ratio
Major CVD	1,779	7.01 (6.69,7.34)	2,143	8.12 (7.78,8.47)	1.22 (1.00,1.50)	1,962	8.06 (7.71,8.42)	1.14 (0.92,1.40)	1,686	6.75 (6.44,7.08)	0.96 (0.81,1.14)	1,584	6.14 (5.84,6.44)	0.86 (0.51,1.45)
Stroke	930	3.49 (3.27,3.72)	1,064	3.86 (3.63,4.09)	1.15 (0.86,1.53)	772	3.02 (2.82,3.24)	0.86 (0.62,1.20)	918	3.51 (3.29,3.74)	1.00 (0.79,1.27)	1,089	3.93 (3.70,4.17)	1.11 (0.52,2.36)
Myocardial infarction	443	1.60 (1.46,1.76)	426	1.47 (1.34,1.62)	0.98 (0.65,1.47)	562	2.12 (1.95,2.30)	1.30 (0.88,1.92)	397	1.47 (1.33,1.62)	0.91 (0.64,1.31)	195	0.67 (0.58,0.77)	0.41 (0.26,0.66)
Heart Failure	530	1.93 (1.77,2.10)	1,024	3.56 (3.35,3.79)	1.98 (1.49,2.62)	838	3.17 (2.96,3.39)	1.62 (1.20,2.19)	628	2.33 (2.15,2.52)	1.20 (0.91,1.60)	355	1.23 (1.10,1.36)	0.62 (0.28,1.40)
Atrial fibrillation	275	1.01 (0.89,1.13)	441	1.55 (1.41,1.69)	1.62 (1.04,2.52)	369	1.41 (1.27,1.56)	1.39 (0.85,2.25)	291	1.09 (0.97,1.22)	1.08 (0.70,1.65)	815	2.84 (2.65,3.04)	2.78 (0.69,11.19)
Coronary Artery Disease	732	2.75 (2.56,2.96)	662	2.36 (2.18,2.54)	0.89 (0.63,1.25)	849	3.31 (3.09,3.53)	1.19 (0.86,1.65)	554	2.12 (1.95,2.31)	0.77 (0.57,1.03)	540	1.97 (1.81,2.14)	0.71 (0.46,1.09)
Deep vein thrombosis	54	0.19 (0.15,0.25)	127	0.44 (0.36,0.52)	2.42 (1.05,5.57)	31	0.12 (0.08,0.16)	0.58 (0.14,2.42)	22	0.08 (0.05,0.12)	0.41 (0.09,1.81)	21	0.07 (0.04,0.11)	0.35 (0.08,1.50)
Cardiovascular mortality	94	0.34 (0.28,0.41)	176	0.60 (0.52,0.70)	1.66 (0.81,3.40)	52	0.19 (0.14,0.25)	0.59 (0.18,1.92)	10	0.04 (0.02,0.07)	0.11 (0.02,0.82)	0	NA	NA
Chronic pulmonary disease	250	0.92 (0.81,1.04)	334	1.17 (1.05,1.30)	1.36 (0.82,2.27)	409	1.57 (1.42,1.72)	1.68 (1.04,2.73)	319	1.20 (1.07,1.33)	1.30 (0.85,1.97)	82	0.29 (0.23,0.35)	0.30 (0.15,0.61)
Acute respiratory distress syndrome	243	0.88 (0.77,0.99)	310	1.07 (0.95,1.19)	1.30 (0.76,2.22)	433	1.62 (1.48,1.78)	1.82 (1.18,2.80)	287	1.06 (0.94,1.18)	1.20 (0.77,1.85)	567	1.96 (1.81,2.13)	2.18 (0.59,7.98)
Seizure	136	0.49 (0.41,0.58)	218	0.76 (0.66,0.86)	1.62 (0.89,2.95)	240	0.90 (0.79,1.02)	1.82 (0.99,3.34)	85	0.32 (0.25,0.39)	0.64 (0.33,1.24)	114	0.39 (0.33,0.47)	0.79 (0.21,2.99)
End-stage renal disease	30	0.11 (0.08,0.15)	143	0.49 (0.41,0.57)	4.91 (2.05,11.76)	56	0.21 (0.16,0.27)	1.90 (0.56,6.43)	6	0.02 (0.01,0.04)	0.19 (0.02,1.38)	50	0.17 (0.13,0.23)	1.52 (0.20,11.35)
Acute kidney injury	136	0.49 (0.42,0.58)	177	0.61 (0.53,0.71)	1.33 (0.70,2.53)	86	0.32 (0.26,0.40)	0.65 (0.26,1.62)	93	0.34 (0.28,0.42)	0.69 (0.35,1.37)	454	1.55 (1.42,1.70)	3.03 (0.52,17.83)
Pancreatitis	69	0.25 (0.19,0.31)	161	0.55 (0.47,0.65)	2.39 (1.07,5.34)	94	0.35 (0.28,0.43)	1.39 (0.51,3.81)	22	0.08 (0.05,0.12)	0.32 (0.08,1.37)	16	0.05 (0.03,0.08)	0.21 (0.03,1.53)
All-cause mortality	4,226	15.20 (14.74,15.66)	9,154	31.36 (30.73,32.01)	2.18 (1.98,2.41)	4,048	15.11 (14.65,15.58)	0.98 (0.86,1.13)	3,142	11.52 (11.12,11.93)	0.75 (0.66,0.86)	1,263	4.29 (4.06,4.54)	0.28 (0.18,0.43)

Note: CI: confidence interval, REF: Reference group; Major CVD: Major Cardiovascular Disease; Composite outcome of stroke, heart failure and coronary artery disease
Hazard Ratio (HR) and 95% Confidence Interval (95% CI) were estimated by Cox regression, HR >1 (or <1) indicates patients with COVID-19 had higher (lower) risk of sequelae compared to the non-COVID-19 control cohort
Un-vaccinated, Incomplete vaccinated, Fully Vaccinated and Received booster dose of COVID-19 vaccines refers to patients who have received 0 dose, 1 doses, 2 doses and ≥3 doses of BioNtech or CoronaVac COVID-19 vaccines, respectively

Supplementary Table 10. Incidence rate and hazard ratio of clinical sequelae in COVID-19 and non-COVID-19 cohort after weighting during 0-30, 31-90, 91-180, 181-270,271-365 days of SARS-CoV-2 infection (Adjusting for the likely variant of SARS-COV-2 responsible for the infection)

0-30 days

	Controls (REF)		COVID-19											
			Unvaccinated			Received single dose			Received two doses			Received booster dose(s)		
**Clinical sequelae**	**Event**	**Incidence per 1,000 person-years**	**Event**	**Incidence per 1,000 person-years**	**Hazard ratio**	**Event**	**Incidence per 1,000 person-years**	**Hazard ratio**	**Event**	**Incidence per 1,000 person-years**	**Hazard ratio**	**Event**	**Incidence per 1,000 person-years**	**Hazard ratio**
Major CVD	1,317	4.38 (4.15,4.63)	6,016	20.92 (20.40,21.46)	4.75 (4.08,5.52)	4,012	13.58 (13.17,14.01)	3.09 (2.57,3.72)	3,278	10.97 (10.60,11.35)	2.50 (2.19,2.86)	2,594	8.69 (8.36,9.03)	1.98 (1.72,2.29)
Stroke	818	2.61 (2.44,2.80)	3,590	12.00 (11.61,12.40)	4.56 (3.76,5.54)	2,422	7.88 (7.57,8.20)	3.01 (2.38,3.80)	2,271	7.30 (7.00,7.60)	2.79 (2.37,3.29)	1,690	5.41 (5.16,5.68)	2.07 (1.71,2.51)
Myocardial infarction	299	0.93 (0.83,1.04)	1,960	6.28 (6.00,6.56)	6.71 (5.21,8.65)	1,550	4.87 (4.63,5.12)	5.23 (3.85,7.11)	974	3.04 (2.85,3.23)	3.27 (2.52,4.24)	919	2.86 (2.68,3.05)	3.08 (2.39,3.97)
Heart Failure	276	0.86 (0.76,0.97)	1,392	4.49 (4.25,4.73)	5.21 (3.87,7.01)	907	2.86 (2.68,3.05)	3.33 (2.30,4.82)	639	2.00 (1.85,2.16)	2.33 (1.70,3.18)	352	1.10 (0.99,1.22)	1.28 (0.86,1.89)
Atrial fibrillation	155	0.48 (0.41,0.57)	697	2.26 (2.10,2.44)	4.65 (3.09,7.01)	594	1.89 (1.74,2.04)	3.89 (2.48,6.08)	423	1.33 (1.21,1.46)	2.74 (1.87,4.02)	506	1.59 (1.45,1.73)	3.27 (2.33,4.59)
Coronary Artery Disease	483	1.54 (1.41,1.69)	2,181	7.18 (6.88,7.48)	4.62 (3.64,5.86)	1,658	5.36 (5.11,5.62)	3.46 (2.61,4.60)	1,258	4.04 (3.83,4.27)	2.62 (2.10,3.26)	1,091	3.51 (3.31,3.73)	2.27 (1.83,2.83)
Deep vein thrombosis	41	0.13 (0.09,0.17)	308	0.98 (0.87,1.09)	7.71 (3.78,15.74)	271	0.85 (0.75,0.95)	6.68 (3.15,14.17)	109	0.34 (0.28,0.41)	2.67 (1.24,5.75)	71	0.22 (0.17,0.28)	1.74 (0.77,3.94)
Cardiovascular mortality	1,103	3.39 (3.20,3.60)	6,406	20.34 (19.85,20.85)	5.97 (5.20,6.85)	3,418	10.66 (10.31,11.02)	3.14 (2.60,3.77)	2,104	6.51 (6.24,6.79)	1.92 (1.61,2.29)	1,051	3.24 (3.05,3.44)	0.96 (0.73,1.25)
Chronic pulmonary disease	115	0.36 (0.30,0.43)	379	1.23 (1.11,1.36)	3.42 (1.99,5.86)	682	2.18 (2.02,2.35)	6.05 (3.62,10.12)	397	1.25 (1.13,1.38)	3.49 (2.32,5.24)	232	0.73 (0.64,0.83)	2.03 (1.31,3.17)
Acute respiratory distress syndrome	189	0.58 (0.50,0.67)	1,150	3.68 (3.47,3.90)	6.28 (4.41,8.92)	710	2.23 (2.07,2.40)	3.81 (2.51,5.77)	446	1.39 (1.26,1.52)	2.37 (1.65,3.41)	428	1.33 (1.21,1.46)	2.28 (1.60,3.25)
Seizure	105	0.32 (0.27,0.39)	792	2.56 (2.38,2.74)	7.85 (4.95,12.45)	568	1.79 (1.65,1.94)	5.51 (3.11,9.79)	232	0.72 (0.63,0.82)	2.22 (1.39,3.56)	297	0.92 (0.82,1.03)	2.84 (1.49,5.42)
End-stage renal disease	40	0.12 (0.09,0.16)	246	0.78 (0.69,0.89)	6.38 (2.83,14.40)	55	0.17 (0.13,0.22)	1.40 (0.35,5.63)	93	0.29 (0.24,0.35)	2.36 (0.96,5.82)	86	0.27 (0.22,0.33)	2.18 (0.59,8.01)
Acute kidney injury	87	0.27 (0.22,0.33)	841	2.69 (2.51,2.88)	10.02 (6.63,15.14)	812	2.55 (2.38,2.73)	9.51 (5.99,15.10)	311	0.97 (0.86,1.08)	3.62 (2.24,5.85)	385	1.19 (1.08,1.31)	4.45 (2.79,7.12)
Pancreatitis	56	0.17 (0.13,0.22)	247	0.79 (0.69,0.89)	4.52 (2.18,9.35)	136	0.43 (0.36,0.50)	2.45 (0.80,7.56)	145	0.45 (0.38,0.53)	2.59 (1.37,4.88)	132	0.41 (0.34,0.48)	2.35 (1.31,4.23)
All-cause mortality	5,456	16.79 (16.35,17.24)	101,849	323.47 (321.49,325.46)	19.20 (18.34,20.09)	47,275	147.43 (146.10,148.76)	8.77 (8.27,9.29)	21,008	65.00 (64.13,65.89)	3.87 (3.64,4.12)	9,358	28.86 (28.28,29.45)	1.72 (1.48,1.99)

Note: CI: confidence interval, REF: Reference group; Major CVD: Major Cardiovascular Disease; Composite outcome of stroke, heart failure and coronary artery disease

Hazard Ratio (HR) and 95% Confidence Interval (95% CI) were estimated by Cox regression, HR >1 (or <1) indicates patients with COVID-19 had higher (lower) risk of sequelae compared to the non-COVID-19 control cohort

Un-vaccinated, Incomplete vaccinated, Fully Vaccinated and Received booster dose of COVID-19 vaccines refers to patients who have received 0 dose, 1 doses, 2 doses and ≥3 doses of BioNtech or CoronaVac COVID-19 vaccines, respectively

Supplementary Table 10. Incidence rate and hazard ratio of clinical sequelae in COVID-19 and non-COVID-19 cohort after weighting during 0-30, 31-90, 91-180, 181-270,271-365 days of SARS-CoV-2 infection (Adjusting for the likely variant of SARS-COV-2 responsible for the infection, continued)

31-90 days

	Controls (REF)		COVID-19											
			Unvaccinated			Incompletely vaccinated			Fully vaccinated			Received booster dose		
Clinical sequelae	Event	Incidence per 1,000 person-years	Event	Incidence per 1,000 person-years	Hazard ratio	Event	Incidence per 1,000 person-years	Hazard ratio	Event	Incidence per 1,000 person-years	Hazard ratio	Event	Incidence per 1,000 person-years	Hazard ratio
Major CVD	3,127	5.31 (5.13,5.50)	5,868	10.44 (10.18,10.71)	1.97 (1.70,2.27)	4,369	7.54 (7.32,7.76)	1.42 (1.20,1.68)	3,272	5.59 (5.40,5.78)	1.05 (0.93,1.19)	3,430	5.88 (5.68,6.08)	1.11 (0.98,1.25)
Stroke	1,779	2.90 (2.77,3.04)	2,901	4.95 (4.78,5.14)	1.71 (1.39,2.10)	2,385	3.95 (3.80,4.12)	1.36 (1.08,1.72)	1,748	2.87 (2.73,3.00)	0.99 (0.84,1.17)	1,585	2.60 (2.47,2.73)	0.90 (0.75,1.08)
Myocardial infarction	695	1.10 (1.02,1.18)	1,229	2.01 (1.90,2.12)	1.82 (1.36,2.44)	679	1.09 (1.01,1.17)	0.99 (0.64,1.53)	711	1.13 (1.05,1.22)	1.03 (0.80,1.33)	696	1.11 (1.03,1.19)	1.01 (0.78,1.31)
Heart Failure	727	1.15 (1.07,1.24)	2,348	3.86 (3.71,4.02)	3.35 (2.71,4.14)	1,216	1.96 (1.85,2.07)	1.69 (1.27,2.25)	1,022	1.63 (1.53,1.73)	1.41 (1.12,1.79)	977	1.56 (1.46,1.66)	1.35 (1.05,1.74)
Atrial fibrillation	474	0.76 (0.69,0.83)	1,132	1.87 (1.77,1.99)	2.48 (1.82,3.37)	702	1.14 (1.05,1.22)	1.50 (1.01,2.24)	629	1.01 (0.93,1.09)	1.34 (1.00,1.77)	504	0.81 (0.74,0.88)	1.07 (0.79,1.46)
Coronary Artery Disease	1,230	2.01 (1.90,2.12)	2,342	3.94 (3.78,4.10)	1.96 (1.57,2.44)	1,523	2.51 (2.39,2.64)	1.25 (0.93,1.67)	1,334	2.19 (2.07,2.31)	1.09 (0.90,1.31)	1,594	2.63 (2.50,2.76)	1.31 (1.11,1.55)
Deep vein thrombosis	118	0.19 (0.15,0.22)	457	0.74 (0.68,0.81)	4.00 (2.33,6.87)	220	0.35 (0.31,0.40)	1.88 (0.96,3.71)	236	0.37 (0.33,0.42)	2.01 (1.24,3.27)	105	0.17 (0.14,0.20)	0.90 (0.48,1.68)
Cardiovascular mortality	1,327	2.09 (1.97,2.20)	2,649	4.29 (4.13,4.46)	2.06 (1.70,2.49)	1,561	2.48 (2.36,2.61)	1.19 (0.92,1.53)	1,247	1.97 (1.86,2.08)	0.94 (0.76,1.17)	690	1.09 (1.01,1.17)	0.52 (0.38,0.72)
Chronic pulmonary disease	309	0.49 (0.44,0.55)	919	1.53 (1.43,1.63)	3.09 (2.12,4.52)	628	1.02 (0.94,1.10)	2.07 (1.25,3.42)	383	0.62 (0.56,0.68)	1.25 (0.87,1.79)	274	0.44 (0.39,0.50)	0.90 (0.60,1.33)
Acute respiratory distress syndrome	539	0.85 (0.78,0.93)	911	1.49 (1.39,1.59)	1.75 (1.21,2.52)	886	1.42 (1.33,1.51)	1.66 (1.13,2.46)	621	0.99 (0.91,1.07)	1.16 (0.87,1.54)	710	1.13 (1.05,1.21)	1.32 (0.97,1.81)
Seizure	290	0.46 (0.41,0.51)	751	1.24 (1.15,1.33)	2.70 (1.74,4.20)	533	0.86 (0.79,0.93)	1.87 (1.08,3.24)	348	0.55 (0.50,0.61)	1.21 (0.83,1.76)	196	0.31 (0.27,0.36)	0.68 (0.40,1.15)
End-stage renal disease	62	0.10 (0.08,0.13)	109	0.18 (0.15,0.21)	1.81 (0.61,5.36)	138	0.22 (0.19,0.26)	2.25 (0.86,5.91)	62	0.10 (0.08,0.13)	1.01 (0.42,2.46)	21	0.03 (0.02,0.05)	0.34 (0.10,1.10)
Acute kidney injury	203	0.32 (0.28,0.37)	778	1.27 (1.18,1.36)	3.95 (2.67,5.85)	509	0.81 (0.75,0.89)	2.54 (1.53,4.20)	250	0.40 (0.35,0.45)	1.24 (0.77,1.99)	257	0.41 (0.36,0.46)	1.27 (0.77,2.11)
Pancreatitis	144	0.23 (0.19,0.27)	486	0.79 (0.72,0.86)	3.48 (1.96,6.18)	263	0.42 (0.37,0.47)	1.85 (0.90,3.80)	153	0.24 (0.21,0.28)	1.07 (0.60,1.91)	175	0.28 (0.24,0.32)	1.22 (0.75,1.98)
All-cause mortality	7,772	12.21 (11.94,12.49)	28,976	46.97 (46.43,47.51)	3.84 (3.61,4.09)	13,386	21.28 (20.92,21.64)	1.74 (1.59,1.91)	8,493	13.42 (13.14,13.71)	1.10 (1.01,1.20)	6,698	10.59 (10.34,10.85)	0.87 (0.76,0.99)

Note: CI: confidence interval, REF: Reference group; Major CVD: Major Cardiovascular Disease; Composite outcome of stroke, heart failure and coronary artery disease

Hazard Ratio (HR) and 95% Confidence Interval (95% CI) were estimated by Cox regression, HR >1 (or <1) indicates patients with COVID-19 had higher (lower) risk of sequelae compared to the non-COVID-19 control cohort

Un-vaccinated, Incomplete vaccinated, Fully Vaccinated and Received booster dose of COVID-19 vaccines refers to patients who have received 0 dose, 1 doses, 2 doses and ≥3 doses of BioNtech or CoronaVac COVID-19 vaccines, respectively

Supplementary Table 10. Incidence rate and hazard ratio of clinical sequelae in COVID-19 and non-COVID-19 cohort after weighting during 0-30, 31-90, 91-180, 181-270,271-365 days of SARS-CoV-2 infection (Adjusting for the likely variant of SARS-COV-2 responsible for the infection, continued)

91-180 days

	Controls (REF)		COVID-19												
			Unvaccinated			Incompletely vaccinated			Fully vaccinated			Received booster dose		
**Clinical sequelae**	**Event**	**Incidence per 1,000 person-years**	**Event**	**Incidence per 1,000 person-years**	**Hazard ratio**	**Event**	**Incidence per 1,000 person-years**	**Hazard ratio**	**Event**	**Incidence per 1,000 person-years**	**Hazard ratio**	**Event**	**Incidence per 1,000 person-years**	**Hazard ratio**
Major CVD	4,244	5.59 (5.42,5.76)	6,730	8.67 (8.47,8.88)	1.56 (1.35,1.80)	5,782	6.85 (6.68,7.03)	1.23 (1.06,1.44)	4,678	5.59 (5.43,5.75)	1.01 (0.91,1.11)	3,201	5.01 (4.84,5.19)	0.89 (0.80,0.99)
Stroke	2,472	3.12 (3.00,3.25)	3,402	4.20 (4.06,4.34)	1.35 (1.11,1.65)	3,176	3.62 (3.49,3.74)	1.16 (0.95,1.43)	2,620	3.01 (2.89,3.12)	0.97 (0.84,1.11)	1,551	2.33 (2.22,2.45)	0.74 (0.63,0.87)
Myocardial infarction	914	1.12 (1.05,1.19)	1,505	1.77 (1.69,1.86)	1.58 (1.18,2.12)	1,148	1.26 (1.19,1.34)	1.13 (0.81,1.56)	931	1.04 (0.97,1.11)	0.93 (0.74,1.16)	893	1.31 (1.22,1.39)	1.17 (0.93,1.47)
Heart Failure	835	1.03 (0.96,1.10)	2,197	2.61 (2.50,2.72)	2.55 (2.04,3.20)	1,507	1.66 (1.58,1.75)	1.64 (1.23,2.17)	1,292	1.45 (1.37,1.53)	1.42 (1.16,1.74)	713	1.04 (0.97,1.12)	1.00 (0.77,1.31)
Atrial fibrillation	681	0.84 (0.78,0.91)	1,070	1.28 (1.20,1.36)	1.52 (1.10,2.10)	1,065	1.18 (1.11,1.26)	1.40 (1.02,1.94)	950	1.07 (1.00,1.14)	1.27 (1.01,1.59)	793	1.17 (1.09,1.25)	1.39 (0.96,2.03)
Coronary Artery Disease	1,700	2.15 (2.05,2.25)	2,757	3.34 (3.22,3.47)	1.56 (1.25,1.93)	2,387	2.70 (2.60,2.81)	1.26 (1.00,1.58)	1,844	2.12 (2.02,2.22)	0.99 (0.84,1.15)	1,503	2.27 (2.16,2.39)	1.05 (0.89,1.25)
Deep vein thrombosis	149	0.18 (0.15,0.21)	275	0.32 (0.29,0.36)	1.77 (0.93,3.36)	341	0.37 (0.33,0.41)	2.04 (1.04,4.00)	98	0.11 (0.09,0.13)	0.59 (0.30,1.16)	135	0.20 (0.16,0.23)	1.08 (0.47,2.50)
Cardiovascular mortality	1,575	1.92 (1.82,2.01)	3,011	3.52 (3.39,3.65)	1.83 (1.52,2.21)	1,663	1.82 (1.73,1.90)	0.95 (0.73,1.22)	1,207	1.34 (1.26,1.41)	0.70 (0.57,0.86)	396	0.57 (0.52,0.63)	0.30 (0.20,0.44)
Chronic pulmonary disease	432	0.54 (0.49,0.59)	973	1.17 (1.10,1.24)	2.19 (1.50,3.21)	857	0.96 (0.90,1.02)	1.81 (1.17,2.78)	555	0.63 (0.58,0.68)	1.18 (0.88,1.58)	422	0.63 (0.57,0.69)	1.15 (0.78,1.68)
Acute respiratory distress syndrome	714	0.87 (0.81,0.94)	1,436	1.69 (1.61,1.78)	1.94 (1.43,2.62)	1,010	1.11 (1.04,1.18)	1.27 (0.88,1.83)	1,016	1.13 (1.06,1.20)	1.29 (1.04,1.61)	592	0.86 (0.80,0.94)	0.99 (0.70,1.41)
Seizure	389	0.48 (0.43,0.52)	1,073	1.28 (1.20,1.35)	2.70 (1.88,3.89)	929	1.02 (0.96,1.09)	2.18 (1.44,3.31)	409	0.45 (0.41,0.50)	0.97 (0.69,1.35)	299	0.44 (0.39,0.49)	0.89 (0.48,1.67)
End-stage renal disease	77	0.09 (0.08,0.12)	141	0.17 (0.14,0.19)	1.77 (0.62,5.04)	181	0.20 (0.17,0.23)	2.13 (0.82,5.56)	39	0.04 (0.03,0.06)	0.46 (0.16,1.33)	0	NA	NA
Acute kidney injury	355	0.43 (0.39,0.48)	672	0.79 (0.73,0.85)	1.83 (1.22,2.76)	495	0.54 (0.50,0.59)	1.26 (0.80,1.97)	439	0.49 (0.44,0.53)	1.13 (0.80,1.61)	143	0.21 (0.18,0.25)	0.48 (0.26,0.88)
Pancreatitis	181	0.22 (0.19,0.25)	173	0.20 (0.17,0.24)	0.92 (0.44,1.94)	184	0.20 (0.17,0.23)	0.92 (0.37,2.27)	193	0.21 (0.18,0.25)	0.97 (0.58,1.63)	103	0.15 (0.12,0.18)	0.68 (0.36,1.28)
All-cause mortality	10,015	12.19 (11.96,12.43)	23,233	27.16 (26.81,27.51)	2.23 (2.08,2.39)	12,940	14.12 (13.88,14.37)	1.16 (1.06,1.27)	8,709	9.64 (9.44,9.85)	0.79 (0.73,0.86)	5,518	8.02 (7.81,8.23)	0.66 (0.57,0.75)

Note: CI: confidence interval, REF: Reference group; Major CVD: Major Cardiovascular Disease; Composite outcome of stroke, heart failure and coronary artery disease

Hazard Ratio (HR) and 95% Confidence Interval (95% CI) were estimated by Cox regression, HR >1 (or <1) indicates patients with COVID-19 had higher (lower) risk of sequelae compared to the non-COVID-19 control cohort

Un-vaccinated, Incomplete vaccinated, Fully Vaccinated and Received booster dose of COVID-19 vaccines refers to patients who have received 0 dose, 1 doses, 2 doses and ≥3 doses of BioNtech or CoronaVac COVID-19 vaccines, respectively

Supplementary Table 10. Incidence rate and hazard ratio of clinical sequelae in COVID-19 and non-COVID-19 cohort after weighting during 0-30, 31-90, 91-180, 181-270,271-365 days of SARS-CoV-2 infection (Adjusting for the likely variant of SARS-COV-2 responsible for the infection, continued)

181-270 days

	Controls (REF)		COVID-19											
			Unvaccinated			Incompletely vaccinated			Fully vaccinated			Received booster dose		
**Clinical sequelae**	**Event**	**Incidence per 1,000 person-years**	**Event**	**Incidence per 1,000 person-years**	**Hazard ratio**	**Event**	**Incidence per 1,000 person-years**	**Hazard ratio**	**Event**	**Incidence per 1,000 person-years**	**Hazard ratio**	**Event**	**Incidence per 1,000 person-years**	**Hazard ratio**
Major CVD	3,464	5.79 (5.60,5.99)	5,117	8.69 (8.46,8.93)	1.50 (1.29,1.74)	4,679	7.46 (7.24,7.67)	1.29 (1.10,1.50)	3,543	5.71 (5.53,5.90)	0.99 (0.89,1.10)	2,506	4.83 (4.64,5.02)	0.83 (0.71,0.98)
Stroke	1,928	3.09 (2.96,3.23)	2,960	4.82 (4.65,4.99)	1.56 (1.28,1.90)	2,259	3.46 (3.31,3.60)	1.12 (0.90,1.39)	1,918	2.97 (2.84,3.11)	0.96 (0.83,1.11)	1,299	2.39 (2.26,2.52)	0.77 (0.62,0.96)
Myocardial infarction	827	1.29 (1.20,1.38)	879	1.36 (1.28,1.46)	1.06 (0.77,1.45)	1,212	1.79 (1.69,1.89)	1.39 (1.03,1.87)	689	1.04 (0.96,1.12)	0.80 (0.63,1.03)	1,084	1.94 (1.82,2.05)	1.52 (0.60,3.82)
Heart Failure	772	1.21 (1.12,1.29)	1,987	3.10 (2.97,3.24)	2.58 (2.06,3.22)	1,489	2.21 (2.10,2.32)	1.83 (1.42,2.37)	812	1.23 (1.14,1.31)	1.02 (0.80,1.29)	677	1.21 (1.12,1.31)	1.00 (0.51,1.99)
Atrial fibrillation	545	0.86 (0.79,0.93)	971	1.53 (1.44,1.63)	1.79 (1.30,2.46)	742	1.11 (1.03,1.19)	1.30 (0.90,1.88)	782	1.19 (1.11,1.27)	1.39 (1.09,1.76)	381	0.68 (0.62,0.76)	0.79 (0.51,1.24)
Coronary Artery Disease	1,464	2.35 (2.23,2.47)	1,707	2.73 (2.60,2.86)	1.16 (0.91,1.48)	2,000	3.04 (2.91,3.18)	1.29 (1.03,1.63)	1,587	2.46 (2.34,2.58)	1.05 (0.89,1.22)	1,099	2.04 (1.92,2.16)	0.87 (0.68,1.10)
Deep vein thrombosis	118	0.18 (0.15,0.22)	121	0.19 (0.15,0.22)	1.02 (0.51,2.06)	86	0.13 (0.10,0.15)	0.69 (0.29,1.67)	136	0.20 (0.17,0.24)	1.11 (0.65,1.91)	48	0.09 (0.06,0.11)	0.47 (0.17,1.30)
Cardiovascular mortality	987	1.53 (1.43,1.62)	1,813	2.79 (2.66,2.92)	1.83 (1.46,2.29)	1,147	1.68 (1.58,1.78)	1.11 (0.84,1.45)	641	0.96 (0.88,1.03)	0.63 (0.48,0.82)	467	0.83 (0.75,0.90)	0.54 (0.31,0.92)
Chronic pulmonary disease	361	0.57 (0.51,0.63)	877	1.39 (1.30,1.48)	2.44 (1.69,3.52)	630	0.94 (0.87,1.02)	1.66 (1.10,2.51)	378	0.58 (0.52,0.64)	1.01 (0.74,1.40)	194	0.35 (0.30,0.40)	0.62 (0.38,1.02)
Acute respiratory distress syndrome	562	0.87 (0.80,0.95)	886	1.37 (1.29,1.47)	1.57 (1.10,2.26)	577	0.85 (0.78,0.92)	0.97 (0.64,1.47)	489	0.73 (0.67,0.80)	0.84 (0.63,1.11)	466	0.83 (0.76,0.91)	0.96 (0.45,2.07)
Seizure	321	0.50 (0.45,0.55)	1,096	1.72 (1.62,1.82)	3.45 (2.43,4.90)	773	1.14 (1.07,1.23)	2.30 (1.52,3.48)	265	0.40 (0.35,0.45)	0.80 (0.54,1.18)	211	0.38 (0.33,0.43)	0.75 (0.25,2.23)
End-stage renal disease	52	0.08 (0.06,0.11)	103	0.16 (0.13,0.19)	1.97 (0.80,4.86)	108	0.16 (0.13,0.19)	1.96 (0.88,4.39)	91	0.14 (0.11,0.17)	1.68 (0.83,3.37)	0	NA	NA
Acute kidney injury	302	0.47 (0.42,0.52)	436	0.68 (0.61,0.74)	1.44 (0.94,2.20)	349	0.51 (0.46,0.57)	1.09 (0.66,1.82)	270	0.40 (0.36,0.45)	0.86 (0.57,1.31)	98	0.17 (0.14,0.21)	0.37 (0.17,0.81)
Pancreatitis	173	0.27 (0.23,0.31)	248	0.38 (0.34,0.43)	1.43 (0.75,2.72)	161	0.24 (0.20,0.28)	0.89 (0.37,2.15)	147	0.22 (0.19,0.26)	0.82 (0.50,1.34)	82	0.14 (0.12,0.18)	0.54 (0.25,1.18)
All-cause mortality	8,094	12.50 (12.23,12.78)	16,767	25.80 (25.41,26.19)	2.06 (1.91,2.23)	9,604	14.07 (13.79,14.36)	1.13 (1.02,1.24)	5,561	8.31 (8.09,8.53)	0.66 (0.60,0.73)	7,917	14.01 (13.71,14.32)	1.12 (0.76,1.64)

Note: CI: confidence interval, REF: Reference group; Major CVD: Major Cardiovascular Disease; Composite outcome of stroke, heart failure and coronary artery disease
Hazard Ratio (HR) and 95% Confidence Interval (95% CI) were estimated by Cox regression, HR >1 (or <1) indicates patients with COVID-19 had higher (lower) risk of sequelae compared to the non-COVID-19 control cohort
Un-vaccinated, Incomplete vaccinated, Fully Vaccinated and Received booster dose of COVID-19 vaccines refers to patients who have received 0 dose, 1 doses, 2 doses and ≥3 doses of BioNtech or CoronaVac COVID-19 vaccines, respectively

Supplementary Table 10. Incidence rate and hazard ratio of clinical sequelae in COVID-19 and non-COVID-19 cohort after weighting during 0-30, 31-90, 91-180, 181-270,271-365 days of SARS-CoV-2 infection (Adjusting for the likely variant of SARS-COV-2 responsible for the infection, continued)

271-365 days

	Controls (REF)		COVID-19											
			Unvaccinated			Incompletely vaccinated			Fully vaccinated			Received booster dose		
**Clinical sequelae**	**Event**	**Incidence per 1,000 person-years**	**Event**	**Incidence per 1,000 person-years**	**Hazard ratio**	**Event**	**Incidence per 1,000 person-years**	**Hazard ratio**	**Event**	**Incidence per 1,000 person-years**	**Hazard ratio**	**Event**	**Incidence per 1,000 person-years**	**Hazard ratio**
Major CVD	2,091	6.03 (5.77,6.29)	2,469	7.53 (7.23,7.83)	1.26 (1.03,1.55)	2,346	6.96 (6.68,7.25)	1.15 (0.93,1.42)	1,815	5.25 (5.01,5.50)	0.87 (0.76,1.01)	1,623	4.88 (4.65,5.13)	0.80 (0.59,1.07)
Stroke	1,099	3.04 (2.86,3.22)	1,170	3.42 (3.23,3.62)	1.13 (0.84,1.53)	925	2.64 (2.47,2.81)	0.86 (0.62,1.21)	963	2.68 (2.52,2.86)	0.88 (0.73,1.08)	1,003	2.86 (2.69,3.04)	0.92 (0.59,1.44)
Myocardial infarction	510	1.37 (1.26,1.49)	512	1.43 (1.32,1.56)	1.06 (0.69,1.62)	649	1.79 (1.66,1.94)	1.30 (0.87,1.94)	459	1.24 (1.13,1.36)	0.91 (0.67,1.23)	242	0.67 (0.59,0.76)	0.48 (0.30,0.77)
Heart Failure	573	1.54 (1.42,1.68)	1,122	3.16 (2.98,3.35)	2.07 (1.56,2.74)	938	2.60 (2.44,2.77)	1.68 (1.24,2.27)	654	1.78 (1.64,1.91)	1.15 (0.88,1.51)	391	1.08 (0.98,1.19)	0.69 (0.33,1.45)
Atrial fibrillation	307	0.83 (0.74,0.93)	509	1.44 (1.32,1.57)	1.74 (1.09,2.79)	410	1.14 (1.04,1.26)	1.38 (0.84,2.26)	318	0.87 (0.77,0.97)	1.04 (0.73,1.49)	563	1.56 (1.44,1.69)	1.86 (0.64,5.40)
Coronary Artery Disease	868	2.41 (2.25,2.57)	831	2.39 (2.23,2.56)	1.00 (0.70,1.41)	994	2.83 (2.65,3.00)	1.17 (0.85,1.63)	678	1.89 (1.75,2.03)	0.79 (0.62,1.00)	644	1.85 (1.71,2.00)	0.76 (0.51,1.15)
Deep vein thrombosis	56	0.15 (0.12,0.19)	147	0.41 (0.35,0.48)	2.74 (1.20,6.26)	30	0.08 (0.06,0.12)	0.55 (0.13,2.29)	29	0.08 (0.05,0.11)	0.53 (0.19,1.51)	28	0.08 (0.05,0.11)	0.49 (0.12,2.05)
Cardiovascular mortality	110	0.29 (0.24,0.35)	109	0.30 (0.25,0.36)	1.01 (0.44,2.30)	52	0.14 (0.11,0.18)	0.48 (0.15,1.57)	8	0.02 (0.01,0.04)	0.07 (0.01,0.50)	0	NA	NA
Chronic pulmonary disease	287	0.78 (0.69,0.87)	371	1.06 (0.95,1.17)	1.37 (0.81,2.32)	465	1.30 (1.19,1.42)	1.67 (1.02,2.73)	341	0.94 (0.84,1.04)	1.20 (0.84,1.73)	108	0.30 (0.25,0.36)	0.38 (0.19,0.75)
Acute respiratory distress syndrome	286	0.77 (0.68,0.86)	363	1.02 (0.91,1.12)	1.33 (0.76,2.31)	495	1.36 (1.25,1.49)	1.76 (1.14,2.72)	301	0.81 (0.73,0.91)	1.06 (0.72,1.57)	594	1.64 (1.51,1.77)	2.08 (0.59,7.29)
Seizure	180	0.48 (0.42,0.56)	273	0.77 (0.68,0.87)	1.61 (0.86,3.01)	312	0.86 (0.77,0.97)	1.78 (0.97,3.25)	142	0.38 (0.32,0.45)	0.79 (0.50,1.25)	125	0.34 (0.29,0.41)	0.69 (0.19,2.56)
End-stage renal disease	42	0.11 (0.08,0.15)	173	0.48 (0.41,0.56)	4.36 (1.83,10.38)	56	0.15 (0.12,0.20)	1.36 (0.40,4.61)	4	0.01 (0.00,0.03)	0.10 (0.01,0.78)	57	0.16 (0.12,0.20)	1.34 (0.18,9.98)
Acute kidney injury	151	0.40 (0.34,0.47)	187	0.52 (0.45,0.60)	1.30 (0.69,2.46)	92	0.25 (0.21,0.31)	0.62 (0.25,1.58)	95	0.26 (0.21,0.31)	0.64 (0.34,1.21)	417	1.14 (1.04,1.25)	2.71 (0.49,14.88)
Pancreatitis	79	0.21 (0.17,0.26)	207	0.58 (0.50,0.66)	2.74 (1.24,6.06)	117	0.32 (0.27,0.38)	1.50 (0.54,4.18)	34	0.09 (0.06,0.12)	0.43 (0.17,1.10)	19	0.05 (0.03,0.08)	0.24 (0.03,1.75)
All-cause mortality	4,662	12.43 (12.08,12.79)	10,053	27.93 (27.39,28.48)	2.26 (2.05,2.49)	4,376	12.00 (11.65,12.36)	0.96 (0.84,1.10)	3,373	9.07 (8.77,9.38)	0.73 (0.65,0.82)	1,423	3.88 (3.68,4.08)	0.31 (0.20,0.47)

Note: CI: confidence interval, REF: Reference group; Major CVD: Major Cardiovascular Disease; Composite outcome of stroke, heart failure and coronary artery disease
Hazard Ratio (HR) and 95% Confidence Interval (95% CI) were estimated by Cox regression, HR >1 (or <1) indicates patients with COVID-19 had higher (lower) risk of sequelae compared to the non-COVID-19 control cohort
Un-vaccinated, Incomplete vaccinated, Fully Vaccinated and Received booster dose of COVID-19 vaccines refers to patients who have received 0 dose, 1 doses, 2 doses and ≥3 doses of BioNtech or CoronaVac COVID-19 vaccines, respectively

Supplementary Table 11. P-value, rank and critical values of clinical sequelae estimated by the Benjamini–Hochberg procedure

Day 0-30

Clinical sequelae	Unvaccinated			Incompletely vaccinated			Fully vaccinated			Received booster dose		
	P-value	Rank	Critical value	P-value	Rank	Critical value	P-value	Rank	Critical value	P-value	Rank	Critical value
Major CVD	<0.001	5	0.01	<0.001	12	0.01	<0.001	8	0.01	<0.001	18	0.02
Stroke	<0.001	6	0.01	<0.001	19	0.02	<0.001	10	0.01	<0.001	26	0.03
Myocardial infarction	<0.001	7	0.01	<0.001	16	0.02	<0.001	20	0.02	<0.001	21	0.02
Heart Failure	<0.001	14	0.01	<0.001	32	0.03	<0.001	38	0.04	0.194	57	0.06
Atrial fibrillation	<0.001	29	0.03	<0.001	36	0.04	<0.001	41	0.04	<0.001	31	0.03
Coronary Artery Disease	<0.001	9	0.01	<0.001	23	0.02	<0.001	22	0.02	<0.001	27	0.03
Deep vein thrombosis	<0.001	39	0.04	<0.001	42	0.04	0.011	53	0.06	0.182	56	0.06
Cardiovascular mortality	<0.001	4	0.00	<0.001	11	0.01	<0.001	25	0.03	0.776	60	0.06
Chronic pulmonary disease	<0.001	47	0.05	<0.001	30	0.03	<0.001	35	0.04	0.002	50	0.05
Acute respiratory distress syndrome	<0.001	13	0.01	<0.001	33	0.03	<0.001	43	0.04	<0.001	44	0.05
Seizure	<0.001	24	0.03	<0.001	37	0.04	0.001	48	0.05	0.001	49	0.05
End-stage renal disease	<0.001	45	0.05	0.572	59	0.06	0.044	54	0.06	0.206	58	0.06
Acute kidney injury	<0.001	15	0.02	<0.001	17	0.02	<0.001	40	0.04	<0.001	34	0.04
Pancreatitis	<0.001	46	0.05	0.115	55	0.06	0.003	51	0.05	0.004	52	0.05
All-cause mortality	<0.001	1	0.00	<0.001	2	0.00	<0.001	3	0.00	<0.001	28	0.03

Un-vaccinated, Incomplete vaccinated, Fully Vaccinated and Received booster dose of COVID-19 vaccines refers to patients who have received 0 dose, 1 doses, 2 doses and ≥3 doses of BioNtech or CoronaVac COVID-19 vaccines, respectively
All P-value reported were two-sided without adjustment for multiple comparison; the exact P-value were presented except for value <0.001
Benjamini-Hochberg critical value, using the formula (i/m)Q, where i = the individual p-value's rank, m = total number of tests, Q = the false discovery rate set at 0.05 (5%)

Supplementary Table 11. P-value, rank and critical values of clinical sequelae estimated by the Benjamini–Hochberg procedure (Continued)

Day 31-90

Clinical sequelae	Unvaccinated			Incompletely vaccinated			Fully vaccinated			Received booster dose		
	P-value	Rank	Critical value	P-value	Rank	Critical value	P-value	Rank	Critical value	P-value	Rank	Critical value
Major CVD	<0.001	4	0.00	<0.001	14	0.01	0.346	46	0.05	0.097	37	0.04
Stroke	<0.001	10	0.01	0.007	24	0.03	0.970	58	0.06	0.250	42	0.04
Myocardial infarction	<0.001	17	0.02	0.987	60	0.06	0.788	55	0.06	0.959	57	0.06
Heart Failure	<0.001	3	0.00	<0.001	16	0.02	0.003	21	0.02	0.020	27	0.03
Atrial fibrillation	<0.001	8	0.01	0.044	31	0.03	0.043	30	0.03	0.680	53	0.06
Coronary Artery Disease	<0.001	7	0.01	0.118	38	0.04	0.323	45	0.05	0.002	20	0.02
Deep vein thrombosis	<0.001	11	0.01	0.064	32	0.03	0.004	23	0.02	0.732	54	0.06
Cardiovascular mortality	<0.001	5	0.01	0.156	40	0.04	0.652	52	0.05	<0.001	15	0.02
Chronic pulmonary disease	<0.001	9	0.01	0.004	22	0.02	0.209	41	0.04	0.583	51	0.05
Acute respiratory distress syndrome	0.001	19	0.02	0.009	25	0.03	0.294	43	0.04	0.081	34	0.04
Seizure	<0.001	13	0.01	0.024	28	0.03	0.303	44	0.05	0.148	39	0.04
End-stage renal disease	0.348	47	0.05	0.097	36	0.04	0.971	59	0.06	0.071	33	0.03
Acute kidney injury	<0.001	6	0.01	<0.001	18	0.02	0.368	49	0.05	0.361	48	0.05
Pancreatitis	<0.001	12	0.01	0.091	35	0.04	0.816	56	0.06	0.431	50	0.05
All-cause mortality	<0.001	1	0.00	<0.001	2	0.00	0.012	26	0.03	0.035	29	0.03

Un-vaccinated, Incomplete vaccinated, Fully Vaccinated and Received booster dose of COVID-19 vaccines refers to patients who have received 0 dose, 1 doses, 2 doses and ≥3 doses of BioNtech or CoronaVac COVID-19 vaccines, respectively
All P-value reported were two-sided without adjustment for multiple comparison; the exact P-value were presented except for value <0.001
Benjamini-Hochberg critical value, using the formula (i/m)Q, where i = the individual p-value's rank, m = total number of tests, Q = the false discovery rate set at 0.05 (5%)

Supplementary Table 11. P-value, rank and critical values of clinical sequelae estimated by the Benjamini–Hochberg procedure (Continued)

Day 91-180												
Clinical sequelae	Unvaccinated			Incompletely vaccinated			Fully vaccinated			Received booster dose		
	P-value	Rank	Critical value	P-value	Rank	Critical value	P-value	Rank	Critical value	P-value	Rank	Critical value
Major CVD	<0.001	7	0.01	0.005	20	0.02	0.791	52	0.05	0.036	30	0.03
Stroke	0.014	23	0.02	0.121	34	0.04	0.758	50	0.05	<0.001	13	0.01
Myocardial infarction	0.003	19	0.02	0.448	44	0.05	0.558	48	0.05	0.197	39	0.04
Heart Failure	<0.001	3	0.00	0.001	15	0.02	0.001	16	0.02	0.987	60	0.06
Atrial fibrillation	0.018	25	0.03	0.032	28	0.03	0.031	27	0.03	0.077	32	0.03
Coronary Artery Disease	<0.001	14	0.01	0.040	31	0.03	0.931	58	0.06	0.549	47	0.05
Deep vein thrombosis	0.122	35	0.04	0.036	29	0.03	0.131	36	0.04	0.867	55	0.06
Cardiovascular mortality	<0.001	6	0.01	0.771	51	0.05	0.001	18	0.02	<0.001	4	0.00
Chronic pulmonary disease	<0.001	11	0.01	0.006	21	0.02	0.246	41	0.04	0.479	46	0.05
Acute respiratory distress syndrome	<0.001	10	0.01	0.187	38	0.04	0.020	26	0.03	0.947	59	0.06
Seizure	<0.001	9	0.01	<0.001	12	0.01	0.878	56	0.06	0.701	49	0.05
End-stage renal disease	0.353	43	0.04	0.117	33	0.03	0.157	37	0.04	<0.001	1	0.00
Acute kidney injury	0.010	22	0.02	0.299	42	0.04	0.477	45	0.05	0.017	24	0.03
Pancreatitis	0.855	53	0.06	0.856	54	0.06	0.921	57	0.06	0.244	40	0.04
All-cause mortality	<0.001	2	0.00	0.001	17	0.02	<0.001	8	0.01	<0.001	5	0.01

Un-vaccinated, Incomplete vaccinated, Fully Vaccinated and Received booster dose of COVID-19 vaccines refers to patients who have received 0 dose, 1 doses, 2 doses and ≥3 doses of BioNtech or CoronaVac COVID-19 vaccines, respectively
All P-value reported were two-sided without adjustment for multiple comparison; the exact P-value were presented except for value <0.001
Benjamini-Hochberg critical value, using the formula (i/m)Q, where i = the individual p-value's rank, m = total number of tests, Q = the false discovery rate set at 0.05 (5%)

Supplementary Table 11. P-value, rank and critical values of clinical sequelae estimated by the Benjamini–Hochberg procedure (Continued)

Day 181-270

Clinical sequelae	Unvaccinated			Incompletely vaccinated			Fully vaccinated			Received booster dose		
	P-value	Rank	Critical value	P-value	Rank	Critical value	P-value	Rank	Critical value	P-value	Rank	Critical value
Major CVD	<0.001	7	0.01	0.001	13	0.01	0.936	58	0.06	0.025	23	0.02
Stroke	<0.001	11	0.01	0.274	37	0.04	0.679	50	0.05	0.020	19	0.02
Myocardial infarction	0.717	52	0.05	0.027	24	0.03	0.093	27	0.03	0.369	41	0.04
Heart Failure	<0.001	4	0.00	<0.001	8	0.01	0.819	54	0.06	0.986	60	0.06
Atrial fibrillation	<0.001	12	0.01	0.146	32	0.03	0.005	15	0.02	0.300	40	0.04
Coronary Artery Disease	0.278	38	0.04	0.023	21	0.02	0.489	45	0.05	0.231	35	0.04
Deep vein thrombosis	0.961	59	0.06	0.460	44	0.05	0.592	48	0.05	0.163	33	0.03
Cardiovascular mortality	<0.001	6	0.01	0.417	42	0.04	0.001	14	0.01	0.022	20	0.02
Chronic pulmonary disease	<0.001	9	0.01	0.014	18	0.02	0.888	55	0.06	0.060	25	0.03
Acute respiratory distress syndrome	0.023	22	0.02	0.931	57	0.06	0.249	36	0.04	0.917	56	0.06
Seizure	<0.001	5	0.01	<0.001	10	0.01	0.285	39	0.04	0.610	49	0.05
End-stage renal disease	0.187	34	0.04	0.096	28	0.03	0.144	31	0.03	<0.001	1	0.00
Acute kidney injury	0.067	26	0.03	0.697	51	0.05	0.522	46	0.05	0.012	17	0.02
Pancreatitis	0.097	29	0.03	0.815	53	0.06	0.454	43	0.04	0.115	30	0.03
All-cause mortality	<0.001	2	0.00	0.007	16	0.02	<0.001	3	0.00	0.583	47	0.05

Un-vaccinated, Incomplete vaccinated, Fully Vaccinated and Received booster dose of COVID-19 vaccines refers to patients who have received 0 dose, 1 doses, 2 doses and ≥3 doses of BioNtech or CoronaVac COVID-19 vaccines, respectively
All P-value reported were two-sided without adjustment for multiple comparison; the exact P-value were presented except for value <0.001
Benjamini-Hochberg critical value, using the formula (i/m)Q, where i = the individual p-value's rank, m = total number of tests, Q = the false discovery rate set at 0.05 (5%)

Supplementary Table 11. P-value, rank and critical value of clinical sequelae estimated by the Benjamini–Hochberg procedure (Continued)

**Day 271-365**												
**Clinical sequelae**	**Unvaccinated**			**Incompletely vaccinated**			**Fully vaccinated**			**Received booster dose**		
	**P-value**	**Rank**	**Critical value**	**P-value**	**Rank**	**Critical value**	**P-value**	**Rank**	**Critical value**	**P-value**	**Rank**	**Critical value**
Major CVD	0.147	23	0.02	0.147	24	0.03	0.098	20	0.02	0.129	22	0.02
Stroke	0.657	55	0.06	0.460	48	0.05	0.284	39	0.04	0.735	57	0.06
Myocardial infarction	0.851	60	0.06	0.171	26	0.03	0.599	53	0.06	0.002	8	0.01
Heart Failure	<0.001	5	0.01	0.001	6	0.01	0.264	36	0.04	0.325	41	0.04
Atrial fibrillation	0.031	14	0.01	0.176	27	0.03	0.726	56	0.06	0.251	34	0.04
Coronary Artery Disease	0.502	49	0.05	0.278	37	0.04	0.066	18	0.02	0.191	30	0.03
Deep vein thrombosis	0.032	15	0.02	0.423	47	0.05	0.245	33	0.03	0.328	42	0.04
Cardiovascular mortality	0.125	21	0.02	0.373	45	0.05	0.013	11	0.01	<0.001	1	0.00
Chronic pulmonary disease	0.239	32	0.03	0.037	16	0.02	0.279	38	0.04	0.005	9	0.01
Acute respiratory distress syndrome	0.311	40	0.04	0.006	10	0.01	0.616	54	0.06	0.237	31	0.03
Seizure	0.181	29	0.03	0.054	17	0.02	0.346	44	0.05	0.579	51	0.05
End-stage renal disease	0.001	7	0.01	0.581	52	0.05	0.030	13	0.01	0.753	58	0.06
Acute kidney injury	0.553	50	0.05	0.336	43	0.04	0.179	28	0.03	0.252	35	0.04
Pancreatitis	0.025	12	0.01	0.417	46	0.05	0.083	19	0.02	0.159	25	0.03
All-cause mortality	<0.001	2	0.00	0.810	59	0.06	<0.001	4	0.00	<0.001	3	0.00

Un-vaccinated, Incomplete vaccinated, Fully Vaccinated and Received booster dose of COVID-19 vaccines refers to patients who have received 0 dose, 1 doses, 2 doses and ≥3 doses of BioNtech or CoronaVac COVID-19 vaccines, respectively
All P-value reported were two-sided without adjustment for multiple comparison; the exact P-value were presented except for value <0.001
Benjamini-Hochberg critical value, using the formula (i/m)Q, where i = the individual p-value's rank, m = total number of tests, Q = the false discovery rate set at 0.05 (5%)

Supplementary Table 12. Incidence rate and hazard ratio of lung cancer, brain cancer and lymphoma in COVID-19 and non-COVID-19 cohort after weighting during 0-30, 31-90, 91-180, 181-270, 271-365 days of SARS-CoV-2 infection

	Controls (REF)		COVID-19												
			Unvaccinated			Received single dose			Received two doses			Received booster dose(s)		
**Clinical sequelae**	**Event**	**Incidence per 1,000 person-years**	**Event**	**Incidence per 1,000 person-years**	**Hazard ratio**	**Event**	**Incidence per 1,000 person-years**	**Hazard ratio**	**Event**	**Incidence per 1,000 person-years**	**Hazard ratio**	**Event**	**Incidence per 1,000 person-years**	**Hazard ratio**
**0-30 days**														
Lung Cancer	221	0.68 (0.60,0.78)	384	1.21 (1.10,1.34)	1.77 (1.08,2.90)	312	0.98 (0.87,1.09)	1.43 (0.79,2.58)	297	0.92 (0.82,1.03)	1.35 (0.90,2.03)	247	0.76 (0.67,0.86)	1.12 (0.70,1.77)
Brain Cancer	12	0.04 (0.02,0.06)	30	0.09 (0.06,0.13)	2.44 (0.31,19.42)	42	0.13 (0.10,0.18)	3.46 (0.43,27.75)	9	0.03 (0.01,0.05)	0.74 (0.09,5.95)	19	0.06 (0.03,0.09)	1.52 (0.32,7.19)
Lymphoma	112	0.35 (0.29,0.42)	309	0.97 (0.87,1.09)	2.81 (1.46,5.41)	114	0.36 (0.30,0.43)	1.03 (0.40,2.68)	183	0.57 (0.49,0.65)	1.64 (0.98,2.73)	205	0.63 (0.55,0.72)	1.82 (1.06,3.11)
**31-90 days**														
Lung Cancer	505	0.80 (0.73,0.87)	1,102	1.78 (1.68,1.89)	2.23 (1.60,3.11)	609	0.97 (0.90,1.05)	1.22 (0.75,1.98)	716	1.14 (1.06,1.22)	1.43 (1.10,1.86)	483	0.76 (0.70,0.84)	0.96 (0.72,1.29)
Brain Cancer	17	0.03 (0.02,0.04)	62	0.10 (0.08,0.13)	3.67 (0.77,17.44)	0	NA	NA	0	NA	NA	7	0.01 (0.00,0.02)	0.38 (0.05,2.91)
Lymphoma	243	0.38 (0.34,0.43)	319	0.51 (0.46,0.57)	1.34 (0.68,2.66)	206	0.33 (0.29,0.38)	0.86 (0.36,2.06)	345	0.55 (0.49,0.61)	1.43 (0.99,2.06)	396	0.62 (0.56,0.69)	1.63 (1.11,2.38)
**91-180 days**														
Lung Cancer	844	1.03 (0.96,1.10)	1,236	1.43 (1.35,1.51)	1.38 (1.00,1.92)	1,258	1.38 (1.30,1.46)	1.34 (0.97,1.84)	1,021	1.14 (1.07,1.21)	1.10 (0.89,1.37)	562	0.82 (0.75,0.89)	0.79 (0.60,1.04)
Brain Cancer	20	0.02 (0.02,0.04)	0	NA	NA	0	NA	NA	10	0.01 (0.01,0.02)	0.47 (0.06,3.70)	22	0.03 (0.02,0.05)	1.27 (0.36,4.50)
Lymphoma	288	0.35 (0.31,0.39)	571	0.66 (0.61,0.71)	1.86 (1.10,3.15)	285	0.31 (0.28,0.35)	0.88 (0.46,1.68)	397	0.44 (0.40,0.48)	1.24 (0.88,1.74)	341	0.49 (0.44,0.55)	1.43 (0.96,2.15)
**181-270 days**														
Lung Cancer	619	0.96 (0.89,1.04)	876	1.33 (1.25,1.42)	1.39 (0.98,1.96)	917	1.35 (1.27,1.44)	1.41 (1.00,1.98)	654	0.98 (0.91,1.06)	1.02 (0.79,1.32)	961	1.72 (1.61,1.83)	1.79 (0.82,3.89)
Brain Cancer	14	0.02 (0.01,0.04)	36	0.06 (0.04,0.08)	2.47 (0.32,19.32)	49	0.07 (0.05,0.10)	3.25 (0.70,14.99)	20	0.03 (0.02,0.05)	1.35 (0.35,5.20)	0	NA	NA
Lymphoma	267	0.41 (0.37,0.46)	209	0.32 (0.28,0.36)	0.77 (0.38,1.55)	234	0.34 (0.30,0.39)	0.83 (0.43,1.60)	288	0.43 (0.38,0.48)	1.04 (0.72,1.50)	241	0.42 (0.37,0.48)	1.04 (0.64,1.69)
**271-365 days**														
Lung Cancer	261	0.70 (0.62,0.79)	306	0.77 (0.69,0.86)	1.20 (0.69,2.09)	334	0.93 (0.83,1.03)	1.31 (0.75,2.29)	366	1.00 (0.90,1.11)	1.42 (1.02,1.99)	162	0.45 (0.39,0.52)	0.63 (0.33,1.19)
Brain Cancer	6	0.02 (0.01,0.03)	0	NA	NA	0	NA	NA	0	NA	NA	0	NA	NA
Lymphoma	140	0.38 (0.32,0.44)	207	0.52 (0.46,0.60)	1.50 (0.70,3.20)	113	0.32 (0.26,0.38)	0.83 (0.33,2.08)	115	0.31 (0.26,0.37)	0.83 (0.46,1.47)	28	0.07 (0.05,0.11)	0.20 (0.05,0.81)

Note: CI: confidence interval, REF: Reference group; Major CVD: Major Cardiovascular Disease; Composite outcome of stroke, heart failure and coronary artery disease
Hazard Ratio (HR) and 95% Confidence Interval (95% CI) were estimated by Cox regression, HR >1 (or <1) indicates patients with COVID-19 had higher (lower) risk of sequelae compared to the non-COVID-19 control cohort
Un-vaccinated, Incomplete vaccinated, Fully Vaccinated and Received booster dose of COVID-19 vaccines refers to patients who have received 0 dose, 1 doses, 2 doses and ≥3 doses of BioNtech or CoronaVac COVID-19 vaccines, respectively

Supplementary Table 13. Incidence rate and hazard ratio of clinical sequelae in COVID-19 and non-COVID-19 cohort after weighting during 0-30, 31-90, 91-180, 181-270, 271-365 days of SARS-CoV-2 infection (Patients aged over 65)

0-30 days

	Controls (REF)		COVID-19												
			Unvaccinated			Received single dose			Received two doses			Received booster dose(s)		
**Clinical sequelae**	**Event**	**Incidence per 1,000 person-years**	**Event**	**Incidence per 1,000 person-years**	**Hazard ratio**	**Event**	**Incidence per 1,000 person-years**	**Hazard ratio**	**Event**	**Incidence per 1,000 person-years**	**Hazard ratio**	**Event**	**Incidence per 1,000 person-years**	**Hazard ratio**
Major CVD	830	10.92 (10.19,11.68)	3,233	45.87 (44.31,47.47)	4.15 (3.47,4.95)	2,655	36.37 (35.00,37.77)	3.31 (2.70,4.06)	2,456	32.73 (31.46,34.05)	2.99 (2.53,3.53)	2,006	26.21 (25.08,27.38)	2.40 (2.00,2.88)
Stroke	555	6.53 (6.00,7.09)	1,988	25.25 (24.15,26.37)	3.81 (3.02,4.81)	1,734	21.33 (20.35,22.35)	3.24 (2.49,4.21)	1,716	20.51 (19.55,21.49)	3.13 (2.53,3.86)	1,351	15.69 (14.86,16.54)	2.40 (1.89,3.04)
Myocardial infarction	190	2.06 (1.79,2.37)	1,249	14.19 (13.42,14.99)	6.77 (5.06,9.05)	1,057	11.79 (11.10,12.52)	5.66 (4.06,7.91)	770	8.44 (7.87,9.06)	4.08 (2.98,5.57)	704	7.58 (7.03,8.15)	3.67 (2.64,5.10)
Heart Failure	225	2.48 (2.17,2.82)	746	8.61 (8.01,9.25)	3.45 (2.49,4.79)	590	6.69 (6.16,7.24)	2.69 (1.82,3.97)	543	6.06 (5.56,6.58)	2.44 (1.72,3.46)	313	3.42 (3.06,3.81)	1.38 (0.86,2.20)
Atrial fibrillation	127	1.42 (1.19,1.69)	523	6.17 (5.65,6.71)	4.30 (2.74,6.76)	475	5.49 (5.02,6.01)	3.85 (2.41,6.14)	384	4.36 (3.94,4.81)	3.06 (2.00,4.67)	531	5.90 (5.41,6.41)	4.14 (2.82,6.08)
Coronary Artery Disease	285	3.35 (2.97,3.75)	1,252	15.27 (14.45,16.14)	4.50 (3.42,5.93)	1,105	13.29 (12.52,14.09)	3.94 (2.88,5.40)	974	11.54 (10.83,12.28)	3.44 (2.63,4.50)	854	10.02 (9.37,10.72)	2.99 (2.25,3.99)
Deep vein thrombosis	29	0.31 (0.21,0.43)	233	2.61 (2.29,2.96)	8.47 (3.69,19.44)	146	1.61 (1.37,1.89)	5.24 (2.20,12.50)	90	0.97 (0.78,1.19)	3.17 (1.27,7.91)	73	0.78 (0.62,0.97)	2.54 (0.91,7.08)
Cardiovascular mortality	967	10.31 (9.67,10.97)	4,043	45.01 (43.64,46.41)	4.33 (3.70,5.06)	2,600	28.52 (27.45,29.64)	2.75 (2.26,3.35)	1,857	20.04 (19.14,20.96)	1.94 (1.59,2.37)	1,055	11.15 (10.49,11.83)	1.08 (0.82,1.42)
Chronic pulmonary disease	74	0.83 (0.65,1.03)	226	2.66 (2.33,3.03)	3.20 (1.71,5.99)	345	3.99 (3.59,4.43)	4.82 (2.66,8.71)	293	3.32 (2.96,3.72)	4.01 (2.39,6.73)	135	1.49 (1.25,1.75)	1.80 (0.95,3.41)
Acute respiratory distress syndrome	132	1.42 (1.19,1.68)	1,113	12.63 (11.91,13.39)	8.76 (5.94,12.93)	643	7.14 (6.60,7.71)	4.98 (3.26,7.62)	370	4.05 (3.65,4.48)	2.83 (1.87,4.30)	371	3.97 (3.58,4.39)	2.78 (1.85,4.20)
Seizure	38	0.41 (0.30,0.56)	263	2.98 (2.64,3.36)	7.15 (3.79,13.48)	155	1.72 (1.47,2.01)	4.15 (1.82,9.45)	107	1.16 (0.95,1.39)	2.80 (1.28,6.14)	100	1.07 (0.88,1.30)	2.59 (1.20,5.61)
End-stage renal disease	38	0.41 (0.30,0.56)	186	2.08 (1.80,2.40)	5.07 (1.55,16.59)	46	0.50 (0.37,0.66)	1.22 (0.27,5.57)	59	0.64 (0.49,0.81)	1.56 (0.41,5.90)	15	0.16 (0.09,0.25)	0.39 (0.07,2.13)
Acute kidney injury	74	0.79 (0.63,0.99)	555	6.29 (5.78,6.82)	7.87 (5.09,12.16)	524	5.83 (5.35,6.35)	7.32 (4.46,12.01)	293	3.20 (2.85,3.58)	4.02 (2.41,6.72)	358	3.82 (3.43,4.22)	4.81 (2.82,8.19)
Pancreatitis	32	0.34 (0.23,0.47)	146	1.63 (1.39,1.92)	4.75 (1.93,11.71)	76	0.84 (0.67,1.05)	2.46 (0.64,9.42)	83	0.90 (0.72,1.10)	2.63 (1.05,6.62)	61	0.65 (0.50,0.83)	1.91 (0.67,5.44)
All-cause mortality	4,738	50.53 (49.10,51.98)	62,225	692.83 (687.41,698.29)	13.61 (12.84,14.43)	35,557	390.09 (386.05,394.16)	7.69 (7.18,8.23)	19,395	209.30 (206.36,212.25)	4.13 (3.84,4.45)	11,256	118.98 (116.80,121.19)	2.35 (1.58,3.50)

Note: CI: confidence interval, REF: Reference group; Major CVD: Major Cardiovascular Disease; Composite outcome of stroke, heart failure and coronary artery disease
Hazard Ratio (HR) and 95% Confidence Interval (95% CI) were estimated by Cox regression, HR >1 (or <1) indicates patients with COVID-19 had higher (lower) risk of sequelae compared to the non-COVID-19 control cohort
Un-vaccinated, Incomplete vaccinated, Fully Vaccinated and Received booster dose of COVID-19 vaccines refers to patients who have received 0 dose, 1 doses, 2 doses and ≥3 doses of BioNtech or CoronaVac COVID-19 vaccines, respectively

Supplementary Table 13. Incidence rate and hazard ratio of clinical sequelae in COVID-19 and non-COVID-19 cohort after weighting during 0-30, 31-90, 91-180, 181-270,271-365 days of SARS-CoV-2 infection (Patients aged over 65, continued)

31-90 days

	Controls (REF)		COVID-19											
			Unvaccinated			Incompletely vaccinated			Fully vaccinated			Received booster dose		
**Clinical sequelae**	**Event**	**Incidence per 1,000 person-years**	**Event**	**Incidence per 1,000 person-years**	**Hazard ratio**	**Event**	**Incidence per 1,000 person-years**	**Hazard ratio**	**Event**	**Incidence per 1,000 person-years**	**Hazard ratio**	**Event**	**Incidence per 1,000 person-years**	**Hazard ratio**
Major CVD	1,901	12.87 (12.30,13.45)	3,878	28.26 (27.37,29.15)	2.20 (1.85,2.61)	2,980	20.88 (20.14,21.64)	1.62 (1.34,1.96)	2,135	14.61 (14.00,15.24)	1.14 (0.97,1.34)	2,578	17.50 (16.83,18.18)	1.36 (1.17,1.58)
Stroke	1,105	6.70 (6.31,7.10)	1,821	11.87 (11.33,12.42)	1.77 (1.40,2.25)	1,626	10.23 (9.74,10.74)	1.53 (1.18,1.98)	1,158	7.12 (6.72,7.54)	1.06 (0.85,1.32)	1,143	6.92 (6.53,7.33)	1.03 (0.83,1.28)
Myocardial infarction	412	2.30 (2.08,2.53)	903	5.26 (4.93,5.61)	2.28 (1.64,3.19)	459	2.62 (2.39,2.87)	1.14 (0.71,1.83)	397	2.24 (2.03,2.47)	0.97 (0.67,1.41)	474	2.66 (2.42,2.90)	1.15 (0.81,1.65)
Heart Failure	619	3.51 (3.24,3.79)	1,661	9.84 (9.38,10.33)	2.81 (2.21,3.56)	980	5.68 (5.33,6.04)	1.62 (1.22,2.16)	947	5.43 (5.10,5.79)	1.55 (1.20,2.01)	1,046	5.94 (5.59,6.31)	1.69 (1.30,2.20)
Atrial fibrillation	368	2.12 (1.91,2.35)	787	4.76 (4.43,5.10)	2.24 (1.60,3.13)	525	3.10 (2.85,3.38)	1.46 (0.94,2.26)	470	2.74 (2.50,2.99)	1.29 (0.91,1.82)	415	2.40 (2.18,2.64)	1.13 (0.75,1.70)
Coronary Artery Disease	698	4.22 (3.92,4.54)	1,693	10.59 (10.09,11.10)	2.51 (1.94,3.24)	925	5.69 (5.33,6.07)	1.35 (0.96,1.90)	780	4.75 (4.43,5.10)	1.13 (0.87,1.46)	1,023	6.25 (5.88,6.64)	1.48 (1.18,1.86)
Deep vein thrombosis	71	0.39 (0.31,0.49)	250	1.44 (1.27,1.62)	3.66 (1.84,7.28)	160	0.90 (0.77,1.05)	2.30 (1.12,4.73)	167	0.93 (0.79,1.08)	2.37 (1.27,4.39)	59	0.32 (0.25,0.41)	0.83 (0.35,1.96)
Cardiovascular mortality	1,121	6.17 (5.82,6.54)	1,701	9.71 (9.26,10.18)	1.57 (1.29,1.91)	1,207	6.78 (6.40,7.17)	1.10 (0.84,1.43)	1,127	6.26 (5.91,6.64)	1.01 (0.80,1.29)	580	3.20 (2.95,3.47)	0.52 (0.37,0.73)
Chronic pulmonary disease	192	1.10 (0.95,1.27)	496	2.99 (2.73,3.26)	2.71 (1.76,4.18)	286	1.69 (1.50,1.89)	1.53 (0.84,2.80)	262	1.52 (1.34,1.71)	1.38 (0.86,2.22)	210	1.21 (1.05,1.38)	1.10 (0.65,1.84)
Acute respiratory distress syndrome	341	1.90 (1.70,2.10)	588	3.42 (3.15,3.70)	1.80 (1.17,2.76)	611	3.48 (3.21,3.76)	1.83 (1.18,2.85)	436	2.45 (2.23,2.69)	1.29 (0.91,1.84)	443	2.47 (2.24,2.70)	1.30 (0.94,1.80)
Seizure	114	0.63 (0.53,0.76)	306	1.78 (1.58,1.98)	2.81 (1.46,5.40)	203	1.15 (1.00,1.32)	1.82 (0.78,4.26)	195	1.09 (0.94,1.25)	1.72 (0.94,3.18)	66	0.36 (0.28,0.46)	0.58 (0.24,1.40)
End-stage renal disease	50	0.28 (0.21,0.36)	44	0.25 (0.19,0.34)	0.92 (0.35,2.41)	65	0.37 (0.28,0.46)	1.33 (0.44,3.98)	36	0.20 (0.14,0.27)	0.73 (0.22,2.44)	0	NA	NA
Acute kidney injury	159	0.88 (0.76,1.03)	417	2.42 (2.20,2.67)	2.73 (1.76,4.24)	385	2.19 (1.98,2.41)	2.47 (1.43,4.28)	193	1.08 (0.94,1.24)	1.22 (0.71,2.12)	184	1.02 (0.88,1.18)	1.15 (0.63,2.11)
Pancreatitis	74	0.41 (0.32,0.51)	243	1.39 (1.22,1.57)	3.42 (1.58,7.41)	128	0.72 (0.60,0.85)	1.77 (0.78,4.05)	115	0.64 (0.53,0.76)	1.57 (0.73,3.41)	94	0.52 (0.42,0.63)	1.28 (0.61,2.69)
All-cause mortality	6,270	34.49 (33.65,35.36)	17,849	101.91 (100.42,103.41)	2.94 (2.75,3.15)	10,316	57.93 (56.83,59.06)	1.68 (1.53,1.84)	7,535	41.86 (40.93,42.82)	1.21 (1.11,1.33)	5,551	30.64 (29.84,31.45)	0.89 (0.79,1.00)

Note: CI: confidence interval, REF: Reference group; Major CVD: Major Cardiovascular Disease; Composite outcome of stroke, heart failure and coronary artery disease
Hazard Ratio (HR) and 95% Confidence Interval (95% CI) were estimated by Cox regression, HR >1 (or <1) indicates patients with COVID-19 had higher (lower) risk of sequelae compared to the non-COVID-19 control cohort
Un-vaccinated, Incomplete vaccinated, Fully Vaccinated and Received booster dose of COVID-19 vaccines refers to patients who have received 0 dose, 1 doses, 2 doses and ≥3 doses of BioNtech or CoronaVac COVID-19 vaccines, respectively

Supplementary Table 13. Incidence rate and hazard ratio of clinical sequelae in COVID-19 and non-COVID-19 cohort after weighting during 0-30, 31-90, 91-180, 181-270,271-365 days of SARS-CoV-2 infection (Patients aged over 65, continued)

91-180 days

	Controls (REF)		COVID-19											
			Unvaccinated			Incompletely vaccinated			Fully vaccinated			Received booster dose		
**Clinical sequelae**	**Event**	**Incidence per 1,000 person-years**	**Event**	**Incidence per 1,000 person-years**	**Hazard ratio**	**Event**	**Incidence per 1,000 person-years**	**Hazard ratio**	**Event**	**Incidence per 1,000 person-years**	**Hazard ratio**	**Event**	**Incidence per 1,000 person-years**	**Hazard ratio**
Major CVD	2,531	13.24 (12.73,13.76)	3,760	19.34 (18.73,19.96)	1.47 (1.23,1.75)	3,207	15.40 (14.87,15.94)	1.17 (0.97,1.40)	3,046	14.78 (14.26,15.31)	1.12 (0.98,1.28)	2,333	14.87 (14.28,15.49)	1.11 (0.96,1.30)
Stroke	1,520	7.13 (6.77,7.49)	1,895	8.71 (8.32,9.10)	1.22 (0.97,1.55)	1,766	7.61 (7.26,7.97)	1.07 (0.83,1.37)	1,750	7.64 (7.29,8.00)	1.07 (0.90,1.28)	1,160	6.63 (6.26,7.03)	0.93 (0.75,1.14)
Myocardial infarction	535	2.31 (2.12,2.51)	911	3.73 (3.49,3.98)	1.62 (1.14,2.30)	661	2.58 (2.39,2.78)	1.12 (0.77,1.62)	608	2.44 (2.25,2.63)	1.06 (0.78,1.44)	592	3.15 (2.91,3.42)	1.36 (0.98,1.90)
Heart Failure	666	2.92 (2.70,3.14)	1,360	5.67 (5.38,5.98)	1.95 (1.52,2.50)	1,127	4.47 (4.22,4.74)	1.54 (1.15,2.07)	1,100	4.48 (4.23,4.76)	1.54 (1.22,1.95)	823	4.43 (4.13,4.74)	1.50 (1.13,2.01)
Atrial fibrillation	509	2.27 (2.08,2.47)	796	3.39 (3.16,3.63)	1.49 (1.04,2.13)	803	3.25 (3.03,3.48)	1.43 (1.02,2.01)	719	2.98 (2.77,3.20)	1.31 (1.00,1.72)	550	3.01 (2.77,3.27)	1.34 (0.96,1.87)
Coronary Artery Disease	961	4.50 (4.22,4.79)	1,659	7.30 (6.96,7.66)	1.63 (1.24,2.14)	1,306	5.51 (5.21,5.81)	1.23 (0.94,1.61)	1,101	4.77 (4.49,5.06)	1.06 (0.85,1.33)	925	5.36 (5.02,5.71)	1.18 (0.93,1.51)
Deep vein thrombosis	86	0.37 (0.30,0.45)	102	0.41 (0.34,0.50)	1.12 (0.52,2.39)	113	0.43 (0.36,0.52)	1.17 (0.47,2.93)	68	0.27 (0.21,0.34)	0.73 (0.31,1.73)	62	0.33 (0.25,0.42)	0.91 (0.35,2.38)
Cardiovascular mortality	1,288	5.48 (5.18,5.78)	1,674	6.72 (6.41,7.05)	1.22 (1.00,1.50)	1,130	4.35 (4.10,4.61)	0.79 (0.60,1.04)	994	3.92 (3.69,4.17)	0.72 (0.56,0.91)	359	1.88 (1.70,2.09)	0.35 (0.22,0.55)
Chronic pulmonary disease	259	1.14 (1.01,1.29)	553	2.35 (2.16,2.55)	2.07 (1.30,3.30)	376	1.52 (1.38,1.68)	1.35 (0.77,2.35)	324	1.34 (1.20,1.49)	1.18 (0.77,1.79)	268	1.47 (1.30,1.65)	1.26 (0.78,2.03)
Acute respiratory distress syndrome	484	2.08 (1.90,2.27)	1,063	4.34 (4.09,4.61)	2.09 (1.47,2.97)	657	2.56 (2.37,2.76)	1.23 (0.81,1.88)	705	2.82 (2.62,3.03)	1.35 (1.02,1.79)	293	1.55 (1.38,1.73)	0.74 (0.49,1.12)
Seizure	163	0.70 (0.60,0.81)	515	2.10 (1.93,2.29)	3.03 (1.91,4.81)	379	1.47 (1.33,1.62)	2.13 (1.25,3.60)	211	0.84 (0.73,0.96)	1.21 (0.70,2.06)	109	0.58 (0.48,0.69)	0.81 (0.37,1.78)
End-stage renal disease	55	0.24 (0.18,0.31)	57	0.23 (0.18,0.30)	0.97 (0.36,2.60)	89	0.34 (0.28,0.42)	1.44 (0.47,4.45)	22	0.09 (0.05,0.13)	0.36 (0.08,1.62)	0	NA	NA
Acute kidney injury	273	1.17 (1.04,1.32)	412	1.68 (1.52,1.85)	1.44 (0.93,2.22)	416	1.62 (1.47,1.78)	1.38 (0.88,2.18)	383	1.53 (1.38,1.69)	1.30 (0.88,1.93)	70	0.37 (0.29,0.46)	0.31 (0.12,0.78)
Pancreatitis	91	0.39 (0.32,0.48)	106	0.43 (0.35,0.51)	1.11 (0.48,2.54)	119	0.46 (0.38,0.55)	1.21 (0.49,2.98)	132	0.52 (0.44,0.62)	1.36 (0.69,2.70)	45	0.24 (0.18,0.32)	0.59 (0.21,1.63)
All-cause mortality	8,231	35.00 (34.25,35.77)	13,987	56.17 (55.24,57.11)	1.61 (1.49,1.73)	9,448	36.34 (35.61,37.07)	1.04 (0.94,1.14)	7,786	30.75 (30.08,31.44)	0.88 (0.80,0.96)	3,911	20.54 (19.91,21.20)	0.59 (0.51,0.67)

Note: CI: confidence interval, REF: Reference group; Major CVD: Major Cardiovascular Disease; Composite outcome of stroke, heart failure and coronary artery disease

Hazard Ratio (HR) and 95% Confidence Interval (95% CI) were estimated by Cox regression, HR >1 (or <1) indicates patients with COVID-19 had higher (lower) risk of sequelae compared to the non-COVID-19 control cohort

Un-vaccinated, Incomplete vaccinated, Fully Vaccinated and Received booster dose of COVID-19 vaccines refers to patients who have received 0 dose, 1 doses, 2 doses and ≥3 doses of BioNtech or CoronaVac COVID-19 vaccines, respectively

Supplementary Table 13. Incidence rate and hazard ratio of clinical sequelae in COVID-19 and non-COVID-19 cohort after weighting during 0-30, 31-90, 91-180, 181-270,271-365 days of SARS-CoV-2 infection (Patients aged over 65, continued)

181-270 days

	Controls (REF)		COVID-19											
			Unvaccinated			Incompletely vaccinated			Fully vaccinated			Received booster dose		
**Clinical sequelae**	**Event**	**Incidence per 1,000 person-years**	**Event**	**Incidence per 1,000 person-years**	**Hazard ratio**	**Event**	**Incidence per 1,000 person-years**	**Hazard ratio**	**Event**	**Incidence per 1,000 person-years**	**Hazard ratio**	**Event**	**Incidence per 1,000 person-years**	**Hazard ratio**
Major CVD	2,094	13.82 (13.24,14.42)	2,917	19.56 (18.86,20.28)	1.42 (1.19,1.69)	2,744	17.52 (16.87,18.18)	1.27 (1.06,1.51)	2,385	15.49 (14.88,16.12)	1.12 (0.97,1.29)	1,628	12.39 (11.81,13.01)	0.90 (0.70,1.16)
Stroke	1,199	7.09 (6.70,7.50)	1,749	10.49 (10.00,10.99)	1.48 (1.17,1.87)	1,438	8.24 (7.82,8.67)	1.16 (0.91,1.48)	1,356	7.92 (7.51,8.35)	1.12 (0.92,1.35)	947	6.28 (5.89,6.69)	0.89 (0.63,1.25)
Myocardial infarction	514	2.80 (2.56,3.05)	607	3.24 (2.99,3.50)	1.16 (0.82,1.63)	706	3.66 (3.40,3.94)	1.31 (0.94,1.83)	441	2.36 (2.15,2.59)	0.85 (0.61,1.18)	326	2.03 (1.82,2.26)	0.73 (0.38,1.41)
Heart Failure	637	3.51 (3.24,3.79)	1,187	6.45 (6.09,6.82)	1.84 (1.44,2.34)	1,047	5.52 (5.19,5.86)	1.57 (1.20,2.06)	710	3.87 (3.59,4.16)	1.10 (0.84,1.45)	470	2.91 (2.65,3.18)	0.83 (0.48,1.41)
Atrial fibrillation	415	2.33 (2.11,2.56)	627	3.48 (3.21,3.76)	1.50 (1.07,2.09)	574	3.09 (2.84,3.35)	1.33 (0.91,1.95)	666	3.68 (3.41,3.97)	1.58 (1.19,2.10)	294	1.86 (1.65,2.08)	0.79 (0.42,1.48)
Coronary Artery Disease	855	5.04 (4.71,5.38)	1,086	6.23 (5.86,6.60)	1.24 (0.94,1.63)	1,106	6.19 (5.84,6.57)	1.23 (0.93,1.63)	957	5.55 (5.20,5.91)	1.10 (0.89,1.37)	591	4.07 (3.75,4.41)	0.81 (0.55,1.19)
Deep vein thrombosis	68	0.36 (0.28,0.46)	91	0.48 (0.39,0.59)	1.32 (0.65,2.69)	79	0.40 (0.33,0.50)	1.11 (0.46,2.68)	90	0.48 (0.39,0.59)	1.31 (0.61,2.82)	23	0.14 (0.09,0.21)	0.39 (0.05,2.83)
Cardiovascular mortality	839	4.49 (4.20,4.81)	1,221	6.39 (6.04,6.76)	1.42 (1.12,1.81)	882	4.50 (4.21,4.80)	1.01 (0.76,1.34)	599	3.16 (2.92,3.42)	0.70 (0.52,0.95)	489	2.97 (2.71,3.24)	0.65 (0.33,1.28)
Chronic pulmonary disease	213	1.18 (1.03,1.35)	434	2.40 (2.19,2.64)	2.03 (1.32,3.11)	414	2.22 (2.02,2.44)	1.87 (1.19,2.95)	209	1.15 (1.00,1.31)	0.97 (0.59,1.60)	117	0.75 (0.62,0.89)	0.64 (0.30,1.37)
Acute respiratory distress syndrome	371	2.01 (1.81,2.22)	646	3.44 (3.18,3.71)	1.71 (1.15,2.56)	370	1.91 (1.72,2.11)	0.95 (0.60,1.50)	298	1.59 (1.42,1.78)	0.79 (0.53,1.18)	107	0.66 (0.54,0.79)	0.33 (0.16,0.68)
Seizure	140	0.76 (0.64,0.89)	356	1.90 (1.71,2.10)	2.52 (1.45,4.36)	208	1.07 (0.94,1.23)	1.43 (0.75,2.73)	114	0.60 (0.50,0.72)	0.80 (0.38,1.69)	42	0.26 (0.19,0.35)	0.34 (0.08,1.40)
End-stage renal disease	27	0.15 (0.10,0.21)	49	0.26 (0.19,0.33)	1.74 (0.57,5.29)	60	0.31 (0.24,0.40)	2.10 (0.77,5.69)	79	0.42 (0.34,0.52)	2.84 (1.23,6.61)	0	NA	NA
Acute kidney injury	222	1.20 (1.05,1.36)	335	1.78 (1.60,1.98)	1.49 (0.96,2.32)	257	1.33 (1.17,1.50)	1.11 (0.64,1.92)	245	1.30 (1.15,1.47)	1.09 (0.66,1.81)	90	0.55 (0.44,0.67)	0.46 (0.18,1.15)
Pancreatitis	83	0.45 (0.36,0.55)	110	0.58 (0.48,0.70)	1.30 (0.65,2.59)	80	0.41 (0.33,0.50)	0.92 (0.31,2.67)	72	0.38 (0.30,0.48)	0.86 (0.37,1.98)	90	0.55 (0.44,0.67)	1.22 (0.24,6.18)
All-cause mortality	6,696	35.84 (34.99,36.70)	10,208	53.41 (52.39,54.46)	1.49 (1.37,1.62)	7,348	37.51 (36.66,38.38)	1.05 (0.95,1.16)	4,674	24.68 (23.98,25.40)	0.69 (0.62,0.77)	2,967	18.01 (17.37,18.67)	0.50 (0.39,0.65)

Note: CI: confidence interval, REF: Reference group; Major CVD: Major Cardiovascular Disease; Composite outcome of stroke, heart failure and coronary artery disease
Hazard Ratio (HR) and 95% Confidence Interval (95% CI) were estimated by Cox regression, HR >1 (or <1) indicates patients with COVID-19 had higher (lower) risk of sequelae compared to the non-COVID-19 control cohort
Un-vaccinated, Incomplete vaccinated, Fully Vaccinated and Received booster dose of COVID-19 vaccines refers to patients who have received 0 dose, 1 doses, 2 doses and ≥3 doses of BioNtech or CoronaVac COVID-19 vaccines, respectively

Supplementary Table 13. Incidence rate and hazard ratio of clinical sequelae in COVID-19 and non-COVID-19 cohort after weighting during 0-30, 31-90, 91-180, 181-270,271-365 days of SARS-CoV-2 infection (Patients aged over 65, continued)

271-365 days

	Controls (REF)		COVID-19											
			Unvaccinated			Incompletely vaccinated			Fully vaccinated			Received booster dose		
**Clinical sequelae**	**Event**	**Incidence per 1,000 person-years**	**Event**	**Incidence per 1,000 person-years**	**Hazard ratio**	**Event**	**Incidence per 1,000 person-years**	**Hazard ratio**	**Event**	**Incidence per 1,000 person-years**	**Hazard ratio**	**Event**	**Incidence per 1,000 person-years**	**Hazard ratio**
Major CVD	1,314	15.65 (14.82,16.51)	1,549	18.49 (17.58,19.42)	1.20 (0.95,1.53)	1,302	16.05 (15.20,16.94)	1.02 (0.79,1.32)	1,279	15.55 (14.72,16.42)	0.99 (0.82,1.20)	827	9.97 (9.31,10.67)	0.63 (0.43,0.92)
Stroke	695	7.42 (6.89,7.99)	739	7.93 (7.38,8.52)	1.06 (0.76,1.48)	563	6.24 (5.75,6.78)	0.84 (0.57,1.24)	652	7.13 (6.60,7.69)	0.96 (0.73,1.26)	469	4.72 (4.31,5.17)	0.63 (0.38,1.06)
Myocardial infarction	342	3.36 (3.01,3.73)	333	3.21 (2.87,3.56)	0.99 (0.62,1.57)	383	3.87 (3.49,4.27)	1.14 (0.73,1.79)	375	3.77 (3.40,4.17)	1.12 (0.77,1.62)	165	1.57 (1.34,1.82)	0.46 (0.13,1.60)
Heart Failure	469	4.66 (4.26,5.10)	749	7.34 (6.82,7.87)	1.63 (1.21,2.21)	663	6.79 (6.29,7.33)	1.45 (1.06,1.97)	624	6.38 (5.89,6.90)	1.36 (1.02,1.82)	303	2.83 (2.53,3.17)	0.60 (0.27,1.35)
Atrial fibrillation	233	2.35 (2.06,2.67)	249	2.49 (2.20,2.82)	1.10 (0.67,1.81)	331	3.46 (3.10,3.84)	1.46 (0.88,2.41)	253	2.63 (2.32,2.97)	1.11 (0.71,1.74)	1,334	12.78 (12.10,13.48)	5.34 (1.14,25.01)
Coronary Artery Disease	521	5.55 (5.09,6.04)	564	5.83 (5.37,6.34)	1.07 (0.71,1.61)	514	5.60 (5.14,6.11)	1.01 (0.67,1.50)	453	4.93 (4.49,5.40)	0.89 (0.64,1.22)	362	3.92 (3.53,4.34)	0.70 (0.36,1.36)
Deep vein thrombosis	45	0.43 (0.32,0.57)	84	0.80 (0.64,0.98)	1.92 (0.72,5.07)	26	0.26 (0.17,0.37)	0.58 (0.14,2.43)	24	0.24 (0.16,0.36)	0.56 (0.17,1.84)	0	NA	NA
Cardiovascular mortality	80	0.78 (0.62,0.96)	75	0.71 (0.56,0.88)	0.84 (0.38,1.87)	45	0.45 (0.33,0.59)	0.59 (0.18,1.89)	8	0.08 (0.03,0.14)	0.10 (0.01,0.73)	0	NA	NA
Chronic pulmonary disease	166	1.67 (1.43,1.94)	219	2.18 (1.91,2.49)	1.36 (0.73,2.52)	241	2.52 (2.22,2.86)	1.49 (0.86,2.61)	231	2.39 (2.09,2.71)	1.43 (0.86,2.36)	26	0.25 (0.17,0.36)	0.15 (0.03,0.66)
Acute respiratory distress syndrome	196	1.92 (1.67,2.20)	230	2.22 (1.95,2.52)	1.20 (0.66,2.19)	317	3.19 (2.86,3.56)	1.65 (1.00,2.71)	245	2.45 (2.16,2.78)	1.27 (0.81,2.00)	156	1.44 (1.22,1.67)	0.74 (0.34,1.60)
Seizure	66	0.64 (0.51,0.82)	139	1.33 (1.12,1.57)	2.14 (0.92,4.97)	136	1.36 (1.14,1.60)	2.10 (1.00,4.43)	43	0.43 (0.31,0.57)	0.66 (0.26,1.69)	73	0.68 (0.53,0.85)	1.03 (0.31,3.50)
End-stage renal disease	22	0.21 (0.13,0.31)	58	0.55 (0.42,0.70)	2.73 (0.99,7.54)	45	0.44 (0.33,0.59)	2.07 (0.59,7.21)	0	NA	NA	0	NA	NA
Acute kidney injury	114	1.12 (0.93,1.34)	117	1.12 (0.93,1.34)	1.04 (0.56,1.93)	61	0.62 (0.48,0.79)	0.55 (0.19,1.56)	66	0.66 (0.52,0.84)	0.59 (0.23,1.50)	397	3.67 (3.32,4.04)	3.16 (0.44,22.55)
Pancreatitis	50	0.49 (0.37,0.64)	66	0.63 (0.49,0.79)	1.32 (0.53,3.32)	25	0.25 (0.17,0.37)	0.52 (0.12,2.16)	24	0.24 (0.15,0.34)	0.48 (0.15,1.57)	0	NA	NA
All-cause mortality	3,738	36.17 (35.02,37.33)	6,210	58.77 (57.33,60.25)	1.67 (1.50,1.85)	3,208	31.90 (30.81,33.02)	0.88 (0.76,1.01)	2,941	29.23 (28.19,30.30)	0.80 (0.70,0.93)	986	9.04 (8.48,9.61)	0.25 (0.16,0.38)

Note: CI: confidence interval, REF: Reference group; Major CVD: Major Cardiovascular Disease; Composite outcome of stroke, heart failure and coronary artery disease

Hazard Ratio (HR) and 95% Confidence Interval (95% CI) were estimated by Cox regression, HR >1 (or <1) indicates patients with COVID-19 had higher (lower) risk of sequelae compared to the non-COVID-19 control cohort

Un-vaccinated, Incomplete vaccinated, Fully Vaccinated and Received booster dose of COVID-19 vaccines refers to patients who have received 0 dose, 1 doses, 2 doses and ≥3 doses of BioNtech or CoronaVac COVID-19 vaccines, respectively

Supplementary Table 14. Incidence rate and hazard ratio of clinical sequelae in COVID-19 and non-COVID-19 cohort after weighting during 0-30, 31-90, 91-180, 181-270, 271-365 days of SARS-CoV-2 infection (Patients aged 65 or under)

0-30 days

	Controls (REF)		COVID-19											
			Unvaccinated			Incompletely vaccinated			Fully vaccinated			Received booster dose		
**Clinical sequelae**	**Event**	**Incidence per 1,000 person-years**	**Event**	**Incidence per 1,000 person-years**	**Hazard ratio**	**Event**	**Incidence per 1,000 person-years**	**Hazard ratio**	**Event**	**Incidence per 1,000 person-years**	**Hazard ratio**	**Event**	**Incidence per 1,000 person-years**	**Hazard ratio**
Major CVD	830	10.92 (10.19,11.68)	3,233	45.87 (44.31,47.47)	4.15 (3.47,4.95)	2,655	36.37 (35.00,37.77)	3.31 (2.70,4.06)	2,456	32.73 (31.46,34.05)	2.99 (2.53,3.53)	2,006	26.21 (25.08,27.38)	2.40 (2.00,2.88)
Stroke	555	6.53 (6.00,7.09)	1,988	25.25 (24.15,26.37)	3.81 (3.02,4.81)	1,734	21.33 (20.35,22.35)	3.24 (2.49,4.21)	1,716	20.51 (19.55,21.49)	3.13 (2.53,3.86)	1,351	15.69 (14.86,16.54)	2.40 (1.89,3.04)
Myocardial infarction	190	2.06 (1.79,2.37)	1,249	14.19 (13.42,14.99)	6.77 (5.06,9.05)	1,057	11.79 (11.10,12.52)	5.66 (4.06,7.91)	770	8.44 (7.87,9.06)	4.08 (2.98,5.57)	704	7.58 (7.03,8.15)	3.67 (2.64,5.10)
Heart Failure	225	2.48 (2.17,2.82)	746	8.61 (8.01,9.25)	3.45 (2.49,4.79)	590	6.69 (6.16,7.24)	2.69 (1.82,3.97)	543	6.06 (5.56,6.58)	2.44 (1.72,3.46)	313	3.42 (3.06,3.81)	1.38 (0.86,2.20)
Atrial fibrillation	127	1.42 (1.19,1.69)	523	6.17 (5.65,6.71)	4.30 (2.74,6.76)	475	5.49 (5.02,6.01)	3.85 (2.41,6.14)	384	4.36 (3.94,4.81)	3.06 (2.00,4.67)	531	5.90 (5.41,6.41)	4.14 (2.82,6.08)
Coronary Artery Disease	285	3.35 (2.97,3.75)	1,252	15.27 (14.45,16.14)	4.50 (3.42,5.93)	1,105	13.29 (12.52,14.09)	3.94 (2.88,5.40)	974	11.54 (10.83,12.28)	3.44 (2.63,4.50)	854	10.02 (9.37,10.72)	2.99 (2.25,3.99)
Deep vein thrombosis	29	0.31 (0.21,0.43)	233	2.61 (2.29,2.96)	8.47 (3.69,19.44)	146	1.61 (1.37,1.89)	5.24 (2.20,12.50)	90	0.97 (0.78,1.19)	3.17 (1.27,7.91)	73	0.78 (0.62,0.97)	2.54 (0.91,7.08)
Cardiovascular mortality	967	10.31 (9.67,10.97)	4,043	45.01 (43.64,46.41)	4.33 (3.70,5.06)	2,600	28.52 (27.45,29.64)	2.75 (2.26,3.35)	1,857	20.04 (19.14,20.96)	1.94 (1.59,2.37)	1,055	11.15 (10.49,11.83)	1.08 (0.82,1.42)
Chronic pulmonary disease	74	0.83 (0.65,1.03)	226	2.66 (2.33,3.03)	3.20 (1.71,5.99)	345	3.99 (3.59,4.43)	4.82 (2.66,8.71)	293	3.32 (2.96,3.72)	4.01 (2.39,6.73)	135	1.49 (1.25,1.75)	1.80 (0.95,3.41)
Acute respiratory distress syndrome	132	1.42 (1.19,1.68)	1,113	12.63 (11.91,13.39)	8.76 (5.94,12.93)	643	7.14 (6.60,7.71)	4.98 (3.26,7.62)	370	4.05 (3.65,4.48)	2.83 (1.87,4.30)	371	3.97 (3.58,4.39)	2.78 (1.85,4.20)
Seizure	38	0.41 (0.30,0.56)	263	2.98 (2.64,3.36)	7.15 (3.79,13.48)	155	1.72 (1.47,2.01)	4.15 (1.82,9.45)	107	1.16 (0.95,1.39)	2.80 (1.28,6.14)	100	1.07 (0.88,1.30)	2.59 (1.20,5.61)
End-stage renal disease	38	0.41 (0.30,0.56)	186	2.08 (1.80,2.40)	5.07 (1.55,16.59)	46	0.50 (0.37,0.66)	1.22 (0.27,5.57)	59	0.64 (0.49,0.81)	1.56 (0.41,5.90)	15	0.16 (0.09,0.25)	0.39 (0.07,2.13)
Acute kidney injury	74	0.79 (0.63,0.99)	555	6.29 (5.78,6.82)	7.87 (5.09,12.16)	524	5.83 (5.35,6.35)	7.32 (4.46,12.01)	293	3.20 (2.85,3.58)	4.02 (2.41,6.72)	358	3.82 (3.43,4.22)	4.81 (2.82,8.19)
Pancreatitis	32	0.34 (0.23,0.47)	146	1.63 (1.39,1.92)	4.75 (1.93,11.71)	76	0.84 (0.67,1.05)	2.46 (0.64,9.42)	83	0.90 (0.72,1.10)	2.63 (1.05,6.62)	61	0.65 (0.50,0.83)	1.91 (0.67,5.44)
All-cause mortality	4,738	50.53 (49.10,51.98)	62,225	692.83 (687.41,698.29)	13.61 (12.84,14.43)	35,557	390.09 (386.05,394.16)	7.69 (7.18,8.23)	19,395	209.30 (206.36,212.25)	4.13 (3.84,4.45)	11,256	118.98 (116.80,121.19)	2.35 (1.58,3.50)

Note: CI: confidence interval, REF: Reference group; Major CVD: Major Cardiovascular Disease; Composite outcome of stroke, heart failure and coronary artery disease
Hazard Ratio (HR) and 95% Confidence Interval (95% CI) were estimated by Cox regression, HR >1 (or <1) indicates patients with COVID-19 had higher (lower) risk of sequelae compared to the non-COVID-19 control cohort
Un-vaccinated, Incomplete vaccinated, Fully Vaccinated and Received booster dose of COVID-19 vaccines refers to patients who have received 0 dose, 1 doses, 2 doses and ≥3 doses of BioNtech or CoronaVac COVID-19 vaccines, respectively

Supplementary Table 14. Incidence rate and hazard ratio of clinical sequelae in COVID-19 and non-COVID-19 cohort after weighting during 0-30, 31-90, 91-180, 181-270, 271-365 days of SARS-CoV-2 infection (Patients aged 65 or under; continued)

31-90 days

	Controls (REF)		COVID-19												
			Unvaccinated			Incompletely vaccinated			Fully vaccinated			Received booster dose			
**Clinical sequelae**	**Event**	**Incidence per 1,000 person-years**	**Event**	**Incidence per 1,000 person-years**	**Hazard ratio**	**Event**	**Incidence per 1,000 person-years**	**Hazard ratio**	**Event**	**Incidence per 1,000 person-years**	**Hazard ratio**	**Event**	**Incidence per 1,000 person-years**	**Hazard ratio**
Major CVD	1,225	2.78 (2.63,2.94)	1,571	3.64 (3.46,3.82)	1.31 (0.93,1.84)	1,152	2.65 (2.50,2.81)	0.95 (0.63,1.44)	1,213	2.76 (2.60,2.92)	0.99 (0.83,1.18)	1,240	2.81 (2.66,2.97)	1.01 (0.82,1.25)
Stroke	670	1.49 (1.38,1.61)	836	1.90 (1.78,2.04)	1.27 (0.78,2.08)	646	1.46 (1.35,1.58)	0.98 (0.56,1.69)	626	1.40 (1.29,1.51)	0.94 (0.73,1.20)	598	1.33 (1.23,1.44)	0.89 (0.62,1.28)
Myocardial infarction	296	0.65 (0.58,0.73)	137	0.31 (0.26,0.36)	0.47 (0.16,1.35)	190	0.42 (0.37,0.49)	0.65 (0.21,2.03)	316	0.70 (0.63,0.78)	1.07 (0.75,1.53)	262	0.58 (0.51,0.65)	0.89 (0.61,1.29)
Heart Failure	115	0.25 (0.21,0.30)	248	0.56 (0.49,0.63)	2.20 (1.05,4.60)	56	0.12 (0.09,0.16)	0.49 (0.12,2.05)	119	0.26 (0.22,0.31)	1.04 (0.60,1.82)	101	0.22 (0.18,0.27)	0.88 (0.46,1.69)
Atrial fibrillation	105	0.23 (0.19,0.28)	269	0.60 (0.54,0.68)	2.59 (1.10,6.13)	123	0.27 (0.23,0.33)	1.18 (0.42,3.30)	170	0.38 (0.32,0.44)	1.61 (0.98,2.66)	147	0.32 (0.28,0.38)	1.39 (0.86,2.27)
Coronary Artery Disease	534	1.19 (1.10,1.30)	726	1.64 (1.53,1.77)	1.38 (0.86,2.21)	600	1.36 (1.25,1.47)	1.14 (0.64,2.01)	569	1.28 (1.18,1.39)	1.07 (0.82,1.40)	633	1.42 (1.31,1.53)	1.19 (0.93,1.52)
Deep vein thrombosis	47	0.10 (0.08,0.14)	165	0.37 (0.32,0.43)	3.59 (1.12,11.50)	33	0.07 (0.05,0.10)	0.72 (0.10,5.29)	73	0.16 (0.13,0.20)	1.56 (0.74,3.31)	41	0.09 (0.07,0.12)	0.88 (0.37,2.12)
Cardiovascular mortality	243	0.54 (0.47,0.61)	148	0.33 (0.28,0.39)	0.62 (0.22,1.76)	147	0.33 (0.28,0.38)	0.61 (0.24,1.56)	137	0.30 (0.26,0.36)	0.57 (0.34,0.95)	125	0.28 (0.23,0.33)	0.51 (0.31,0.86)
Chronic pulmonary disease	117	0.26 (0.21,0.31)	354	0.80 (0.72,0.88)	3.08 (1.40,6.74)	285	0.64 (0.57,0.72)	2.48 (1.06,5.79)	130	0.29 (0.24,0.34)	1.12 (0.65,1.93)	80	0.18 (0.14,0.22)	0.69 (0.37,1.26)
Acute respiratory distress syndrome	199	0.44 (0.38,0.50)	392	0.88 (0.80,0.97)	2.00 (0.97,4.15)	277	0.62 (0.55,0.70)	1.41 (0.64,3.13)	179	0.40 (0.34,0.46)	0.90 (0.57,1.43)	203	0.45 (0.39,0.51)	1.02 (0.63,1.66)
Seizure	183	0.40 (0.35,0.47)	375	0.85 (0.76,0.94)	2.10 (1.06,4.16)	342	0.77 (0.69,0.85)	1.90 (0.91,3.96)	157	0.35 (0.30,0.41)	0.86 (0.54,1.38)	120	0.27 (0.22,0.32)	0.66 (0.37,1.18)
End-stage renal disease	16	0.04 (0.02,0.06)	39	0.09 (0.06,0.12)	2.47 (0.32,19.17)	82	0.18 (0.15,0.23)	5.18 (0.88,30.38)	25	0.06 (0.04,0.08)	1.56 (0.43,5.67)	20	0.04 (0.03,0.07)	1.24 (0.35,4.46)
Acute kidney injury	46	0.10 (0.08,0.14)	127	0.28 (0.24,0.34)	2.80 (0.84,9.29)	67	0.15 (0.12,0.19)	1.46 (0.44,4.87)	55	0.12 (0.09,0.16)	1.19 (0.49,2.89)	76	0.17 (0.13,0.21)	1.64 (0.67,4.00)
Pancreatitis	73	0.16 (0.13,0.20)	234	0.52 (0.46,0.59)	3.26 (1.31,8.09)	118	0.26 (0.22,0.31)	1.63 (0.49,5.46)	47	0.10 (0.08,0.14)	0.65 (0.28,1.53)	84	0.18 (0.15,0.23)	1.15 (0.61,2.18)
All-cause mortality	1,599	3.52 (3.35,3.69)	3,165	7.06 (6.81,7.30)	2.01 (1.62,2.48)	1,312	2.92 (2.76,3.08)	0.83 (0.58,1.18)	988	2.18 (2.05,2.32)	0.62 (0.51,0.75)	972	2.14 (2.01,2.28)	0.61 (0.47,0.79)

Note: CI: confidence interval, REF: Reference group; Major CVD: Major Cardiovascular Disease; Composite outcome of stroke, heart failure and coronary artery disease
Hazard Ratio (HR) and 95% Confidence Interval (95% CI) were estimated by Cox regression, HR >1 (or <1) indicates patients with COVID-19 had higher (lower) risk of sequelae compared to the non-COVID-19 control cohort
Un-vaccinated, Incomplete vaccinated, Fully Vaccinated and Received booster dose of COVID-19 vaccines refers to patients who have received 0 dose, 1 doses, 2 doses and ≥3 doses of BioNtech or CoronaVac COVID-19 vaccines, respectively

Supplementary Table 14. Incidence rate and hazard ratio of clinical sequelae in COVID-19 and non-COVID-19 cohort after weighting during 0-30, 31-90, 91-180, 181-270, 271-365 days of SARS-CoV-2 infection (Patients aged 65 or under; continued)

91-180 days

	Controls (REF)		COVID-19											
			Unvaccinated			Incompletely vaccinated			Fully vaccinated			Received booster dose		
**Clinical sequelae**	**Event**	**Incidence per 1,000 person-years**	**Event**	**Incidence per 1,000 person-years**	**Hazard ratio**	**Event**	**Incidence per 1,000 person-years**	**Hazard ratio**	**Event**	**Incidence per 1,000 person-years**	**Hazard ratio**	**Event**	**Incidence per 1,000 person-years**	**Hazard ratio**
Major CVD	1,699	2.99 (2.85,3.13)	2,647	4.42 (4.26,4.59)	1.49 (1.13,1.95)	2,415	3.81 (3.66,3.97)	1.29 (0.97,1.70)	1,744	2.76 (2.64,2.90)	0.93 (0.80,1.08)	1,100	2.27 (2.14,2.41)	0.75 (0.64,0.89)
Stroke	938	1.62 (1.52,1.73)	1,180	1.94 (1.83,2.05)	1.20 (0.80,1.81)	1,337	2.08 (1.97,2.19)	1.30 (0.89,1.89)	926	1.44 (1.35,1.54)	0.90 (0.73,1.10)	497	1.01 (0.92,1.10)	0.61 (0.48,0.78)
Myocardial infarction	384	0.66 (0.59,0.72)	465	0.75 (0.68,0.82)	1.14 (0.59,2.21)	436	0.67 (0.61,0.73)	1.01 (0.52,1.96)	342	0.53 (0.47,0.59)	0.80 (0.57,1.12)	319	0.64 (0.58,0.72)	0.99 (0.72,1.35)
Heart Failure	171	0.29 (0.25,0.34)	505	0.81 (0.75,0.89)	2.82 (1.54,5.19)	251	0.38 (0.34,0.44)	1.35 (0.52,3.51)	240	0.37 (0.33,0.42)	1.29 (0.86,1.95)	58	0.12 (0.09,0.15)	0.39 (0.19,0.81)
Atrial fibrillation	163	0.28 (0.24,0.33)	98	0.16 (0.13,0.19)	0.57 (0.16,2.00)	172	0.26 (0.23,0.31)	0.95 (0.38,2.37)	254	0.39 (0.35,0.44)	1.40 (0.94,2.10)	266	0.54 (0.48,0.60)	1.91 (0.94,3.88)
Coronary Artery Disease	737	1.28 (1.19,1.37)	1,170	1.91 (1.80,2.02)	1.49 (1.00,2.22)	1,096	1.70 (1.60,1.81)	1.33 (0.90,1.97)	769	1.20 (1.12,1.29)	0.94 (0.75,1.17)	600	1.22 (1.13,1.32)	0.96 (0.76,1.21)
Deep vein thrombosis	63	0.11 (0.08,0.14)	86	0.14 (0.11,0.17)	1.30 (0.38,4.42)	217	0.33 (0.29,0.38)	3.14 (1.21,8.11)	30	0.05 (0.03,0.07)	0.44 (0.16,1.26)	42	0.08 (0.06,0.11)	0.77 (0.32,1.82)
Cardiovascular mortality	301	0.51 (0.46,0.57)	675	1.08 (1.01,1.17)	2.11 (1.31,3.38)	421	0.64 (0.58,0.71)	1.25 (0.64,2.42)	245	0.38 (0.33,0.43)	0.73 (0.49,1.10)	98	0.20 (0.16,0.24)	0.38 (0.19,0.79)
Chronic pulmonary disease	174	0.30 (0.26,0.35)	341	0.56 (0.50,0.62)	1.86 (0.87,3.96)	469	0.72 (0.66,0.79)	2.42 (1.27,4.63)	240	0.37 (0.33,0.42)	1.25 (0.84,1.86)	150	0.30 (0.26,0.36)	1.01 (0.63,1.61)
Acute respiratory distress syndrome	233	0.40 (0.35,0.45)	490	0.79 (0.72,0.86)	1.97 (1.05,3.70)	379	0.58 (0.53,0.64)	1.44 (0.72,2.88)	324	0.50 (0.45,0.56)	1.24 (0.87,1.77)	214	0.43 (0.37,0.49)	1.10 (0.74,1.64)
Seizure	226	0.39 (0.34,0.44)	495	0.81 (0.74,0.88)	2.11 (1.16,3.84)	520	0.80 (0.74,0.87)	2.12 (1.13,3.95)	206	0.32 (0.28,0.36)	0.83 (0.56,1.25)	135	0.27 (0.23,0.32)	0.68 (0.38,1.23)
End-stage renal disease	22	0.04 (0.02,0.06)	63	0.10 (0.08,0.13)	2.75 (0.38,19.97)	84	0.13 (0.10,0.16)	3.57 (0.76,16.79)	18	0.03 (0.02,0.04)	0.74 (0.17,3.30)	0	NA	NA
Acute kidney injury	85	0.15 (0.12,0.18)	166	0.27 (0.23,0.31)	1.84 (0.61,5.50)	38	0.06 (0.04,0.08)	0.40 (0.05,2.93)	64	0.10 (0.08,0.12)	0.68 (0.32,1.45)	70	0.14 (0.11,0.18)	0.95 (0.43,2.10)
Pancreatitis	90	0.15 (0.12,0.19)	64	0.10 (0.08,0.13)	0.66 (0.15,2.84)	66	0.10 (0.08,0.13)	0.63 (0.09,4.53)	66	0.10 (0.08,0.13)	0.64 (0.30,1.36)	57	0.11 (0.09,0.15)	0.80 (0.38,1.68)
All-cause mortality	1,907	3.25 (3.11,3.40)	4,137	6.65 (6.45,6.86)	2.05 (1.69,2.47)	2,058	3.14 (3.01,3.28)	0.97 (0.73,1.28)	1,111	1.71 (1.61,1.81)	0.53 (0.44,0.63)	1,160	2.33 (2.20,2.47)	0.72 (0.55,0.92)

Note: CI: confidence interval, REF: Reference group; Major CVD: Major Cardiovascular Disease; Composite outcome of stroke, heart failure and coronary artery disease
Hazard Ratio (HR) and 95% Confidence Interval (95% CI) were estimated by Cox regression, HR >1 (or <1) indicates patients with COVID-19 had higher (lower) risk of sequelae compared to the non-COVID-19 control cohort
Un-vaccinated, Incomplete vaccinated, Fully Vaccinated and Received booster dose of COVID-19 vaccines refers to patients who have received 0 dose, 1 doses, 2 doses and ≥3 doses of BioNtech or CoronaVac COVID-19 vaccines, respectively

Supplementary Table 14. Incidence rate and hazard ratio of clinical sequelae in COVID-19 and non-COVID-19 cohort after weighting during 0-30, 31-90, 91-180, 181-270, 271-365 days of SARS-CoV-2 infection (Patients aged 65 or under; continued)

181-270 days

	Controls (REF)		COVID-19											
			Unvaccinated			Incompletely vaccinated			Fully vaccinated			Received booster dose		
**Clinical sequelae**	**Event**	**Incidence per 1,000 person-years**	**Event**	**Incidence per 1,000 person-years**	**Hazard ratio**	**Event**	**Incidence per 1,000 person-years**	**Hazard ratio**	**Event**	**Incidence per 1,000 person-years**	**Hazard ratio**	**Event**	**Incidence per 1,000 person-years**	**Hazard ratio**
Major CVD	1,365	3.06 (2.90,3.23)	1,778	3.92 (3.74,4.11)	1.28 (0.94,1.75)	1,821	3.88 (3.70,4.06)	1.27 (0.94,1.71)	1,304	2.80 (2.65,2.95)	0.91 (0.78,1.07)	1,066	2.78 (2.62,2.95)	0.91 (0.73,1.14)
Stroke	729	1.61 (1.49,1.72)	972	2.11 (1.98,2.24)	1.31 (0.86,1.99)	731	1.53 (1.42,1.65)	0.95 (0.60,1.52)	651	1.37 (1.27,1.48)	0.86 (0.69,1.07)	503	1.29 (1.18,1.40)	0.80 (0.60,1.08)
Myocardial infarction	316	0.69 (0.62,0.77)	246	0.52 (0.46,0.59)	0.76 (0.34,1.72)	499	1.03 (0.95,1.13)	1.49 (0.82,2.70)	263	0.55 (0.49,0.62)	0.80 (0.56,1.14)	666	1.69 (1.56,1.82)	2.47 (0.72,8.52)
Heart Failure	135	0.29 (0.25,0.35)	320	0.68 (0.61,0.76)	2.31 (1.12,4.76)	327	0.67 (0.60,0.75)	2.28 (1.13,4.63)	148	0.31 (0.26,0.36)	1.04 (0.65,1.67)	128	0.32 (0.27,0.39)	1.10 (0.42,2.91)
Atrial fibrillation	128	0.28 (0.23,0.33)	280	0.60 (0.53,0.67)	2.15 (0.96,4.79)	84	0.17 (0.14,0.22)	0.63 (0.19,2.11)	166	0.35 (0.30,0.40)	1.25 (0.80,1.95)	130	0.33 (0.28,0.39)	1.16 (0.65,2.10)
Coronary Artery Disease	608	1.34 (1.24,1.45)	717	1.55 (1.44,1.66)	1.15 (0.72,1.85)	913	1.91 (1.79,2.04)	1.42 (0.94,2.14)	663	1.40 (1.30,1.51)	1.04 (0.83,1.31)	496	1.28 (1.17,1.39)	0.95 (0.70,1.29)
Deep vein thrombosis	46	0.10 (0.08,0.13)	0	NA	NA	0	NA	NA	55	0.12 (0.09,0.15)	1.15 (0.54,2.44)	28	0.07 (0.05,0.10)	0.71 (0.22,2.35)
Cardiovascular mortality	168	0.36 (0.31,0.42)	138	0.29 (0.25,0.35)	0.81 (0.28,2.35)	176	0.36 (0.31,0.42)	1.00 (0.42,2.35)	76	0.16 (0.13,0.20)	0.44 (0.23,0.81)	89	0.22 (0.18,0.27)	0.61 (0.30,1.22)
Chronic pulmonary disease	148	0.32 (0.28,0.38)	260	0.56 (0.49,0.63)	1.72 (0.80,3.71)	171	0.36 (0.31,0.41)	1.10 (0.43,2.83)	184	0.39 (0.33,0.44)	1.19 (0.78,1.81)	91	0.23 (0.19,0.28)	0.71 (0.35,1.44)
Acute respiratory distress syndrome	192	0.42 (0.36,0.48)	261	0.56 (0.49,0.63)	1.33 (0.61,2.92)	223	0.46 (0.40,0.52)	1.10 (0.47,2.56)	200	0.42 (0.36,0.48)	1.00 (0.66,1.50)	223	0.56 (0.49,0.64)	1.36 (0.62,2.96)
Seizure	186	0.41 (0.35,0.47)	701	1.51 (1.40,1.63)	3.72 (2.31,5.97)	544	1.13 (1.04,1.23)	2.78 (1.64,4.71)	158	0.33 (0.28,0.39)	0.81 (0.53,1.24)	138	0.35 (0.30,0.41)	0.87 (0.30,2.51)
End-stage renal disease	25	0.05 (0.04,0.08)	36	0.08 (0.06,0.11)	1.40 (0.32,6.12)	51	0.10 (0.08,0.14)	1.89 (0.50,7.21)	17	0.03 (0.02,0.05)	0.63 (0.18,2.18)	0	NA	NA
Acute kidney injury	81	0.18 (0.14,0.22)	33	0.07 (0.05,0.10)	0.39 (0.09,1.70)	66	0.14 (0.11,0.17)	0.77 (0.23,2.62)	54	0.11 (0.09,0.15)	0.64 (0.31,1.31)	22	0.06 (0.03,0.08)	0.31 (0.07,1.30)
Pancreatitis	91	0.20 (0.16,0.24)	167	0.35 (0.30,0.41)	1.80 (0.63,5.12)	89	0.18 (0.15,0.23)	0.94 (0.23,3.84)	81	0.17 (0.14,0.21)	0.86 (0.47,1.58)	38	0.10 (0.07,0.13)	0.48 (0.18,1.33)
All-cause mortality	1,478	3.21 (3.05,3.37)	2,702	5.73 (5.52,5.95)	1.79 (1.43,2.23)	1,353	2.79 (2.64,2.94)	0.87 (0.65,1.17)	1,045	2.18 (2.05,2.31)	0.68 (0.57,0.81)	1,325	3.35 (3.17,3.53)	1.04 (0.67,1.63)

Note: CI: confidence interval, REF: Reference group; Major CVD: Major Cardiovascular Disease; Composite outcome of stroke, heart failure and coronary artery disease
Hazard Ratio (HR) and 95% Confidence Interval (95% CI) were estimated by Cox regression, HR >1 (or <1) indicates patients with COVID-19 had higher (lower) risk of sequelae compared to the non-COVID-19 control cohort
Un-vaccinated, Incomplete vaccinated, Fully Vaccinated and Received booster dose of COVID-19 vaccines refers to patients who have received 0 dose, 1 doses, 2 doses and ≥3 doses of BioNtech or CoronaVac COVID-19 vaccines, respectively

Supplementary Table 14. Incidence rate and hazard ratio of clinical sequelae in COVID-19 and non-COVID-19 cohort after weighting during 0-30, 31-90, 91-180, 181-270, 271-365 days of SARS-CoV-2 infection (Patients aged 65 or under; continued)

271-365 days

	Controls (REF)		COVID-19											
			Unvaccinated			Incompletely vaccinated			Fully vaccinated			Received booster dose		
**Clinical sequelae**	**Event**	**Incidence per 1,000 person-years**	**Event**	**Incidence per 1,000 person-years**	**Hazard ratio**	**Event**	**Incidence per 1,000 person-years**	**Hazard ratio**	**Event**	**Incidence per 1,000 person-years**	**Hazard ratio**	**Event**	**Incidence per 1,000 person-years**	**Hazard ratio**
Major CVD	774	2.94 (2.74,3.15)	634	2.23 (2.06,2.40)	0.82 (0.51,1.33)	1,065	4.23 (3.98,4.49)	1.42 (0.97,2.08)	634	2.44 (2.26,2.64)	0.83 (0.67,1.02)	877	3.60 (3.37,3.85)	1.20 (0.69,2.11)
Stroke	402	1.50 (1.36,1.66)	353	1.22 (1.09,1.35)	0.88 (0.44,1.77)	340	1.33 (1.19,1.47)	0.87 (0.46,1.66)	363	1.37 (1.24,1.52)	0.91 (0.68,1.21)	554	2.23 (2.05,2.43)	1.46 (0.62,3.42)
Myocardial infarction	168	0.62 (0.53,0.72)	83	0.28 (0.22,0.35)	0.50 (0.15,1.59)	282	1.09 (0.96,1.22)	1.73 (0.80,3.73)	106	0.40 (0.33,0.48)	0.64 (0.38,1.07)	151	0.60 (0.51,0.71)	0.95 (0.54,1.68)
Heart Failure	109	0.40 (0.33,0.49)	69	0.24 (0.19,0.30)	0.63 (0.16,2.52)	259	1.00 (0.88,1.13)	2.45 (1.07,5.59)	69	0.26 (0.20,0.32)	0.63 (0.34,1.18)	91	0.36 (0.30,0.45)	0.90 (0.37,2.19)
Atrial fibrillation	72	0.27 (0.21,0.34)	165	0.56 (0.48,0.65)	2.12 (0.86,5.22)	49	0.19 (0.14,0.25)	0.71 (0.10,5.14)	88	0.33 (0.27,0.41)	1.23 (0.67,2.28)	54	0.22 (0.17,0.28)	0.82 (0.32,2.10)
Coronary Artery Disease	342	1.28 (1.15,1.42)	215	0.74 (0.64,0.84)	0.63 (0.32,1.26)	513	2.00 (1.84,2.18)	1.54 (0.89,2.67)	245	0.93 (0.82,1.05)	0.73 (0.52,1.02)	279	1.13 (1.00,1.27)	0.86 (0.54,1.37)
Deep vein thrombosis	11	0.04 (0.02,0.07)	40	0.13 (0.10,0.18)	3.29 (0.60,17.90)	0	NA	NA	5	0.02 (0.01,0.04)	0.49 (0.06,3.98)	24	0.09 (0.06,0.14)	2.23 (0.47,10.58)
Cardiovascular mortality	16	0.06 (0.04,0.10)	95	0.32 (0.26,0.39)	4.29 (0.97,19.00)	0	NA	NA	0	NA	NA	0	NA	NA
Chronic pulmonary disease	123	0.46 (0.38,0.54)	146	0.50 (0.43,0.59)	1.18 (0.39,3.57)	217	0.84 (0.73,0.96)	1.83 (0.73,4.57)	126	0.48 (0.40,0.57)	1.04 (0.64,1.70)	79	0.32 (0.25,0.40)	0.69 (0.32,1.51)
Acute respiratory distress syndrome	82	0.30 (0.24,0.38)	127	0.43 (0.36,0.51)	1.56 (0.54,4.48)	182	0.70 (0.61,0.81)	2.27 (0.96,5.35)	63	0.24 (0.18,0.30)	0.78 (0.39,1.53)	171	0.68 (0.58,0.79)	2.17 (0.41,11.50)
Seizure	115	0.42 (0.35,0.51)	140	0.48 (0.41,0.57)	1.22 (0.52,2.89)	166	0.64 (0.55,0.75)	1.51 (0.60,3.81)	100	0.37 (0.31,0.45)	0.88 (0.52,1.47)	8	0.03 (0.02,0.06)	0.08 (0.01,0.56)
End-stage renal disease	17	0.06 (0.04,0.10)	84	0.28 (0.23,0.35)	4.97 (1.21,20.42)	0	NA	NA	5	0.02 (0.01,0.04)	0.27 (0.03,2.11)	33	0.13 (0.09,0.18)	1.98 (0.25,15.55)
Acute kidney injury	36	0.13 (0.10,0.18)	0	NA	NA	22	0.08 (0.05,0.12)	0.61 (0.08,4.50)	37	0.14 (0.10,0.19)	1.03 (0.42,2.52)	43	0.17 (0.13,0.23)	1.24 (0.43,3.56)
Pancreatitis	27	0.10 (0.07,0.14)	90	0.30 (0.25,0.37)	3.42 (0.93,12.58)	103	0.39 (0.32,0.48)	3.91 (1.06,14.47)	11	0.04 (0.02,0.07)	0.41 (0.09,1.78)	14	0.06 (0.03,0.09)	0.54 (0.07,4.08)
All-cause mortality	926	3.41 (3.20,3.64)	1,461	4.95 (4.70,5.21)	1.55 (1.14,2.11)	933	3.58 (3.36,3.82)	1.04 (0.72,1.51)	673	2.51 (2.33,2.71)	0.73 (0.59,0.91)	416	1.65 (1.50,1.81)	0.48 (0.27,0.84)

Note: CI: confidence interval, REF: Reference group; Major CVD: Major Cardiovascular Disease; Composite outcome of stroke, heart failure and coronary artery disease

Hazard Ratio (HR) and 95% Confidence Interval (95% CI) were estimated by Cox regression, HR >1 (or <1) indicates patients with COVID-19 had higher (lower) risk of sequelae compared to the non-COVID-19 control cohort

Un-vaccinated, Incomplete vaccinated, Fully Vaccinated and Received booster dose of COVID-19 vaccines refers to patients who have received 0 dose, 1 doses, 2 doses and ≥3 doses of BioNtech or CoronaVac COVID-19 vaccines, respectively

Supplementary Table 15. Incidence rate and hazard ratio of clinical sequelae in COVID-19 and non-COVID-19 cohort after weighting during 0-30, 31-90, 91-180, 181-270, 271-365 days of SARS-CoV-2 infection (Patients with Charlson Co-morbidity Index <4)

0-30days

	Controls (REF)		COVID-19											
			Unvaccinated			Incompletely vaccinated			Fully vaccinated			Received booster dose		
**Clinical sequelae**	**Event**	**Incidence per 1,000 person-years**	**Event**	**Incidence per 1,000 person-years**	**Hazard ratio**	**Event**	**Incidence per 1,000 person-years**	**Hazard ratio**	**Event**	**Incidence per 1,000 person-years**	**Hazard ratio**	**Event**	**Incidence per 1,000 person-years**	**Hazard ratio**
Major CVD	790	2.97 (2.76,3.18)	3,153	12.01 (11.60,12.44)	4.04 (3.12,5.23)	2,039	7.75 (7.42,8.09)	2.61 (1.93,3.53)	1,701	6.41 (6.11,6.72)	2.16 (1.82,2.57)	1,439	5.13 (4.87,5.40)	1.73 (1.46,2.05)
Stroke	474	1.74 (1.59,1.90)	2,006	7.48 (7.16,7.81)	4.29 (3.10,5.93)	1,297	4.82 (4.56,5.09)	2.77 (1.92,3.99)	1,080	3.98 (3.75,4.22)	2.29 (1.84,2.84)	852	2.95 (2.75,3.15)	1.69 (1.36,2.11)
Myocardial infarction	162	0.59 (0.50,0.68)	744	2.74 (2.55,2.94)	4.64 (2.74,7.84)	536	1.97 (1.81,2.15)	3.34 (1.76,6.34)	388	1.42 (1.28,1.56)	2.41 (1.67,3.47)	365	1.25 (1.13,1.39)	2.13 (1.52,2.98)
Heart Failure	71	0.26 (0.20,0.33)	284	1.04 (0.93,1.17)	4.03 (1.79,9.08)	281	1.03 (0.92,1.16)	3.99 (1.80,8.87)	193	0.71 (0.61,0.81)	2.73 (1.59,4.69)	126	0.43 (0.36,0.51)	1.67 (0.95,2.95)
Atrial fibrillation	60	0.22 (0.17,0.28)	298	1.10 (0.98,1.23)	5.04 (2.06,12.32)	181	0.67 (0.58,0.77)	3.06 (1.24,7.59)	130	0.48 (0.40,0.56)	2.18 (1.18,4.02)	199	0.69 (0.60,0.79)	3.15 (1.90,5.23)
Coronary Artery Disease	303	1.12 (1.00,1.26)	1,020	3.81 (3.59,4.05)	3.39 (2.18,5.26)	665	2.49 (2.31,2.69)	2.21 (1.29,3.80)	544	2.02 (1.86,2.20)	1.80 (1.34,2.42)	534	1.88 (1.73,2.05)	1.67 (1.27,2.20)
Deep vein thrombosis	14	0.05 (0.03,0.09)	247	0.91 (0.80,1.02)	17.46 (6.19,49.23)	125	0.46 (0.39,0.55)	8.87 (2.36,33.34)	8	0.03 (0.01,0.05)	0.53 (0.07,4.14)	28	0.10 (0.06,0.13)	1.85 (0.58,5.94)
Cardiovascular mortality	242	0.88 (0.77,1.00)	2,036	7.47 (7.15,7.80)	8.49 (6.07,11.88)	899	3.30 (3.09,3.52)	3.75 (2.40,5.87)	430	1.57 (1.42,1.72)	1.78 (1.27,2.49)	191	0.66 (0.57,0.75)	0.75 (0.48,1.15)
Chronic pulmonary disease	65	0.24 (0.18,0.30)	208	0.77 (0.67,0.88)	3.26 (1.17,9.06)	399	1.48 (1.34,1.63)	6.25 (2.89,13.52)	202	0.74 (0.64,0.85)	3.14 (1.87,5.26)	147	0.51 (0.43,0.60)	2.17 (1.28,3.69)
Acute respiratory distress syndrome	91	0.33 (0.27,0.40)	1,421	5.23 (4.97,5.51)	15.86 (10.18,24.69)	198	0.73 (0.63,0.84)	2.21 (0.91,5.38)	171	0.62 (0.53,0.72)	1.89 (1.11,3.22)	155	0.53 (0.45,0.62)	1.62 (0.98,2.66)
Seizure	63	0.23 (0.18,0.29)	419	1.56 (1.41,1.71)	6.81 (3.49,13.28)	418	1.55 (1.41,1.70)	6.78 (3.28,14.05)	145	0.53 (0.45,0.62)	2.32 (1.32,4.08)	109	0.38 (0.31,0.45)	1.65 (0.91,2.99)
End-stage renal disease	7	0.03 (0.01,0.05)	7	0.03 (0.01,0.05)	0.95 (0.11,8.13)	10	0.04 (0.02,0.07)	1.37 (0.16,11.69)	20	0.07 (0.04,0.11)	2.69 (0.64,11.28)	19	0.07 (0.04,0.10)	2.46 (0.58,10.36)
Acute kidney injury	24	0.09 (0.06,0.13)	164	0.60 (0.52,0.70)	6.98 (2.17,22.45)	406	1.49 (1.35,1.64)	17.24 (7.49,39.71)	30	0.11 (0.07,0.15)	1.25 (0.42,3.78)	101	0.35 (0.28,0.42)	4.01 (1.85,8.68)
Pancreatitis	33	0.12 (0.09,0.17)	187	0.69 (0.59,0.79)	5.69 (1.91,16.94)	139	0.51 (0.43,0.60)	4.25 (1.14,15.76)	82	0.30 (0.24,0.37)	2.48 (1.19,5.16)	108	0.37 (0.31,0.45)	3.08 (1.59,5.96)
All-cause mortality	721	2.62 (2.43,2.81)	19,352	70.97 (69.98,71.98)	27.10 (23.83,30.82)	8,494	31.16 (30.50,31.83)	11.90 (10.11,14.01)	2,239	8.16 (7.83,8.50)	3.12 (2.66,3.65)	744	2.55 (2.37,2.74)	0.97 (0.78,1.22)

Note: CI: confidence interval, REF: Reference group; Major CVD: Major Cardiovascular Disease; Composite outcome of stroke, heart failure and coronary artery disease
Hazard Ratio (HR) and 95% Confidence Interval (95% CI) were estimated by Cox regression, HR >1 (or <1) indicates patients with COVID-19 had higher (lower) risk of sequelae compared to the non-COVID-19 control cohort
Un-vaccinated, Incomplete vaccinated, Fully Vaccinated and Received booster dose of COVID-19 vaccines refers to patients who have received 0 dose, 1 doses, 2 doses and ≥3 doses of BioNtech or CoronaVac COVID-19 vaccines, respectively

Supplementary Table 15. Incidence rate and hazard ratio of clinical sequelae in COVID-19 and non-COVID-19 cohort after weighting during 0-30, 31-90, 91-180, 181-270, 271-365 days of SARS-CoV-2 infection (Patients with Charlson Co-morbidity Index <4; continued)

31-90 days

	Controls (REF)		COVID-19											
			Unvaccinated			Incompletely vaccinated			Fully vaccinated			Received booster dose		
**Clinical sequelae**	**Event**	**Incidence per 1,000 person-years**	**Event**	**Incidence per 1,000 person-years**	**Hazard ratio**	**Event**	**Incidence per 1,000 person-years**	**Hazard ratio**	**Event**	**Incidence per 1,000 person-years**	**Hazard ratio**	**Event**	**Incidence per 1,000 person-years**	**Hazard ratio**
Major CVD	1,991	3.81 (3.64,3.98)	3,606	7.01 (6.78,7.24)	1.84 (1.47,2.30)	2,485	4.80 (4.62,4.99)	1.26 (0.97,1.64)	1,944	3.73 (3.57,3.90)	0.98 (0.85,1.13)	1,966	3.62 (3.46,3.79)	0.95 (0.83,1.09)
Stroke	1,094	2.04 (1.93,2.17)	1,526	2.90 (2.76,3.05)	1.42 (0.99,2.03)	1,364	2.58 (2.44,2.72)	1.26 (0.88,1.81)	985	1.85 (1.73,1.96)	0.90 (0.73,1.11)	888	1.59 (1.49,1.70)	0.78 (0.64,0.95)
Myocardial infarction	402	0.74 (0.68,0.82)	488	0.92 (0.84,1.00)	1.23 (0.67,2.26)	331	0.62 (0.56,0.69)	0.83 (0.38,1.82)	426	0.79 (0.72,0.87)	1.06 (0.77,1.46)	357	0.63 (0.57,0.70)	0.85 (0.63,1.16)
Heart Failure	201	0.37 (0.32,0.43)	738	1.38 (1.29,1.49)	3.72 (2.29,6.05)	192	0.36 (0.31,0.41)	0.97 (0.42,2.22)	278	0.52 (0.46,0.58)	1.39 (0.93,2.07)	164	0.29 (0.25,0.34)	0.78 (0.49,1.25)
Atrial fibrillation	194	0.36 (0.31,0.41)	397	0.75 (0.68,0.82)	2.08 (1.05,4.09)	291	0.55 (0.49,0.61)	1.52 (0.72,3.17)	253	0.47 (0.42,0.53)	1.31 (0.86,1.99)	275	0.49 (0.44,0.55)	1.37 (0.94,1.99)
Coronary Artery Disease	808	1.53 (1.42,1.64)	1,606	3.06 (2.92,3.22)	2.01 (1.45,2.77)	1,010	1.93 (1.81,2.05)	1.26 (0.83,1.91)	827	1.57 (1.46,1.68)	1.03 (0.82,1.28)	1,057	1.92 (1.81,2.04)	1.26 (1.04,1.52)
Deep vein thrombosis	61	0.11 (0.09,0.14)	261	0.49 (0.43,0.55)	4.35 (1.82,10.42)	32	0.06 (0.04,0.08)	0.54 (0.07,3.89)	53	0.10 (0.08,0.13)	0.88 (0.39,1.97)	59	0.10 (0.08,0.13)	0.93 (0.43,2.00)
Cardiovascular mortality	343	0.63 (0.57,0.70)	262	0.49 (0.43,0.55)	0.77 (0.33,1.81)	162	0.30 (0.26,0.35)	0.48 (0.19,1.19)	232	0.43 (0.38,0.49)	0.68 (0.44,1.04)	176	0.31 (0.27,0.36)	0.49 (0.32,0.77)
Chronic pulmonary disease	183	0.34 (0.30,0.39)	459	0.87 (0.79,0.95)	2.54 (1.35,4.79)	389	0.73 (0.66,0.81)	2.15 (1.05,4.38)	161	0.30 (0.26,0.35)	0.88 (0.54,1.44)	171	0.31 (0.27,0.36)	0.91 (0.58,1.42)
Acute respiratory distress syndrome	321	0.59 (0.53,0.66)	586	1.10 (1.02,1.19)	1.86 (1.04,3.32)	466	0.87 (0.80,0.95)	1.47 (0.80,2.69)	321	0.60 (0.53,0.66)	1.00 (0.70,1.44)	353	0.63 (0.56,0.70)	1.06 (0.77,1.45)
Seizure	188	0.35 (0.30,0.40)	432	0.82 (0.74,0.90)	2.35 (1.23,4.49)	445	0.84 (0.76,0.92)	2.40 (1.28,4.52)	186	0.35 (0.30,0.40)	1.00 (0.63,1.57)	111	0.20 (0.16,0.24)	0.57 (0.34,0.96)
End-stage renal disease	16	0.03 (0.02,0.05)	40	0.07 (0.05,0.10)	2.57 (0.33,19.90)	57	0.11 (0.08,0.14)	3.68 (0.48,28.54)	29	0.05 (0.04,0.08)	1.87 (0.51,6.87)	22	0.04 (0.02,0.06)	1.34 (0.37,4.86)
Acute kidney injury	62	0.12 (0.09,0.15)	238	0.45 (0.39,0.51)	3.88 (1.59,9.45)	257	0.48 (0.43,0.54)	4.18 (1.84,9.48)	76	0.14 (0.11,0.17)	1.22 (0.57,2.64)	51	0.09 (0.07,0.12)	0.79 (0.33,1.87)
Pancreatitis	97	0.18 (0.15,0.22)	440	0.82 (0.75,0.90)	4.61 (2.25,9.48)	161	0.30 (0.26,0.35)	1.68 (0.60,4.76)	69	0.13 (0.10,0.16)	0.72 (0.34,1.51)	100	0.18 (0.14,0.21)	0.99 (0.55,1.78)
All-cause mortality	1,475	2.72 (2.59,2.86)	5,047	9.44 (9.18,9.70)	3.46 (2.83,4.23)	2,068	3.86 (3.69,4.03)	1.42 (1.06,1.89)	1,023	1.90 (1.78,2.02)	0.70 (0.57,0.85)	1,586	2.81 (2.68,2.95)	1.03 (0.45,2.36)

Note: CI: confidence interval, REF: Reference group; Major CVD: Major Cardiovascular Disease; Composite outcome of stroke, heart failure and coronary artery disease
Hazard Ratio (HR) and 95% Confidence Interval (95% CI) were estimated by Cox regression, HR >1 (or <1) indicates patients with COVID-19 had higher (lower) risk of sequelae compared to the non-COVID-19 control cohort
Un-vaccinated, Incomplete vaccinated, Fully Vaccinated and Received booster dose of COVID-19 vaccines refers to patients who have received 0 dose, 1 doses, 2 doses and ≥3 doses of BioNtech or CoronaVac COVID-19 vaccines, respectively

Supplementary Table 15. Incidence rate and hazard ratio of clinical sequelae in COVID-19 and non-COVID-19 cohort after weighting during 0-30, 31-90, 91-180, 181-270, 271-365 days of SARS-CoV-2 infection (Patients with Charlson Co-morbidity Index <4; continued)

91-180 days

	Controls (REF)		COVID-19											
			Unvaccinated			Incompletely vaccinated			Fully vaccinated			Received booster dose		
**Clinical sequelae**	**Event**	**Incidence per 1,000 person-years**	**Event**	**Incidence per 1,000 person-years**	**Hazard ratio**	**Event**	**Incidence per 1,000 person-years**	**Hazard ratio**	**Event**	**Incidence per 1,000 person-years**	**Hazard ratio**	**Event**	**Incidence per 1,000 person-years**	**Hazard ratio**
Major CVD	2,777	4.11 (3.96,4.27)	4,576	6.41 (6.22,6.59)	1.56 (1.28,1.92)	3,684	4.89 (4.73,5.05)	1.20 (0.96,1.49)	2,883	3.86 (3.72,4.00)	0.94 (0.84,1.07)	2,059	3.48 (3.34,3.64)	0.84 (0.74,0.95)
Stroke	1,507	2.18 (2.07,2.29)	1,920	2.63 (2.51,2.75)	1.21 (0.88,1.67)	1,836	2.38 (2.27,2.49)	1.10 (0.80,1.50)	1,570	2.05 (1.95,2.16)	0.95 (0.80,1.12)	1,019	1.68 (1.58,1.78)	0.76 (0.63,0.91)
Myocardial infarction	535	0.77 (0.70,0.83)	853	1.15 (1.08,1.23)	1.50 (0.92,2.46)	545	0.70 (0.64,0.76)	0.91 (0.52,1.60)	487	0.63 (0.58,0.69)	0.83 (0.61,1.12)	506	0.83 (0.76,0.90)	1.07 (0.82,1.41)
Heart Failure	306	0.44 (0.39,0.49)	809	1.09 (1.02,1.17)	2.52 (1.58,4.03)	641	0.82 (0.76,0.89)	1.92 (1.10,3.34)	382	0.49 (0.45,0.55)	1.15 (0.82,1.61)	187	0.31 (0.26,0.35)	0.67 (0.43,1.05)
Atrial fibrillation	319	0.46 (0.41,0.51)	454	0.62 (0.56,0.67)	1.35 (0.71,2.57)	354	0.46 (0.41,0.51)	1.00 (0.52,1.92)	480	0.62 (0.57,0.68)	1.37 (1.01,1.87)	361	0.59 (0.53,0.66)	1.28 (0.92,1.77)
Coronary Artery Disease	1,131	1.65 (1.56,1.75)	2,122	2.92 (2.79,3.04)	1.76 (1.31,2.37)	1,527	2.00 (1.90,2.10)	1.21 (0.88,1.67)	1,122	1.48 (1.40,1.57)	0.90 (0.74,1.09)	988	1.65 (1.55,1.76)	1.00 (0.82,1.21)
Deep vein thrombosis	82	0.12 (0.09,0.14)	75	0.10 (0.08,0.13)	0.87 (0.26,2.88)	227	0.29 (0.25,0.33)	2.51 (1.05,6.00)	62	0.08 (0.06,0.10)	0.69 (0.31,1.54)	63	0.10 (0.08,0.13)	0.86 (0.40,1.82)
Cardiovascular mortality	444	0.63 (0.58,0.70)	697	0.94 (0.87,1.01)	1.48 (0.93,2.35)	491	0.63 (0.58,0.69)	0.99 (0.54,1.83)	369	0.48 (0.43,0.53)	0.75 (0.53,1.06)	93	0.15 (0.12,0.18)	0.24 (0.13,0.43)
Chronic pulmonary disease	259	0.37 (0.33,0.42)	675	0.92 (0.85,0.99)	2.46 (1.42,4.26)	679	0.88 (0.81,0.95)	2.36 (1.39,3.99)	342	0.45 (0.40,0.50)	1.20 (0.85,1.70)	241	0.40 (0.35,0.45)	1.06 (0.69,1.63)
Acute respiratory distress syndrome	380	0.55 (0.49,0.60)	954	1.29 (1.21,1.37)	2.35 (1.50,3.68)	647	0.83 (0.77,0.90)	1.51 (0.90,2.53)	613	0.79 (0.73,0.86)	1.44 (1.09,1.91)	276	0.45 (0.40,0.51)	0.84 (0.59,1.20)
Seizure	249	0.36 (0.31,0.40)	610	0.83 (0.77,0.90)	2.34 (1.38,4.00)	684	0.88 (0.82,0.95)	2.50 (1.48,4.22)	239	0.31 (0.27,0.35)	0.88 (0.59,1.30)	169	0.28 (0.24,0.32)	0.76 (0.46,1.25)
End-stage renal disease	33	0.05 (0.03,0.06)	61	0.08 (0.06,0.11)	1.81 (0.21,15.66)	141	0.18 (0.15,0.21)	4.01 (0.89,18.01)	25	0.03 (0.02,0.05)	0.73 (0.16,3.25)	0	NA	NA
Acute kidney injury	116	0.17 (0.14,0.20)	239	0.32 (0.28,0.36)	1.93 (0.74,5.06)	22	0.03 (0.02,0.04)	0.17 (0.02,1.22)	107	0.14 (0.11,0.17)	0.83 (0.44,1.58)	48	0.08 (0.06,0.10)	0.47 (0.20,1.11)
Pancreatitis	117	0.17 (0.14,0.20)	102	0.14 (0.11,0.17)	0.81 (0.20,3.27)	125	0.16 (0.13,0.19)	0.93 (0.23,3.83)	102	0.13 (0.11,0.16)	0.77 (0.40,1.47)	74	0.12 (0.10,0.15)	0.76 (0.39,1.48)
All-cause mortality	2,058	2.94 (2.82,3.07)	5,268	7.09 (6.90,7.28)	2.40 (1.99,2.89)	2,540	3.25 (3.13,3.38)	1.09 (0.84,1.43)	1,397	1.81 (1.71,1.90)	0.61 (0.51,0.72)	7,231	11.78 (11.51,12.06)	4.08 (0.74,22.49)

Note: CI: confidence interval, REF: Reference group; Major CVD: Major Cardiovascular Disease; Composite outcome of stroke, heart failure and coronary artery disease
Hazard Ratio (HR) and 95% Confidence Interval (95% CI) were estimated by Cox regression, HR >1 (or <1) indicates patients with COVID-19 had higher (lower) risk of sequelae compared to the non-COVID-19 control cohort
Un-vaccinated, Incomplete vaccinated, Fully Vaccinated and Received booster dose of COVID-19 vaccines refers to patients who have received 0 dose, 1 doses, 2 doses and ≥3 doses of BioNtech or CoronaVac COVID-19 vaccines, respectively

Supplementary Table 15. Incidence rate and hazard ratio of clinical sequelae in COVID-19 and non-COVID-19 cohort after weighting during 0-30, 31-90, 91-180, 181-270, 271-365 days of SARS-CoV-2 infection (Patients with Charlson Co-morbidity Index <4; continued)

181-270 days

	Controls (REF)		COVID-19											
			Unvaccinated			Incompletely vaccinated			Fully vaccinated			Received booster dose		
**Clinical sequelae**	**Event**	**Incidence per 1,000 person-years**	**Event**	**Incidence per 1,000 person-years**	**Hazard ratio**	**Event**	**Incidence per 1,000 person-years**	**Hazard ratio**	**Event**	**Incidence per 1,000 person-years**	**Hazard ratio**	**Event**	**Incidence per 1,000 person-years**	**Hazard ratio**
Major CVD	2,254	4.25 (4.08,4.43)	3,070	5.67 (5.47,5.87)	1.33 (1.06,1.68)	2,991	5.35 (5.16,5.54)	1.26 (1.02,1.56)	2,108	3.82 (3.66,3.98)	0.90 (0.79,1.02)	1,756	2.76 (2.64,2.89)	0.65 (0.48,0.89)
Stroke	1,227	2.26 (2.14,2.39)	1,879	3.39 (3.24,3.55)	1.50 (1.11,2.02)	1,310	2.29 (2.17,2.41)	1.01 (0.73,1.40)	1,114	1.97 (1.86,2.09)	0.87 (0.73,1.04)	851	1.32 (1.23,1.41)	0.58 (0.41,0.82)
Myocardial infarction	460	0.84 (0.76,0.92)	395	0.70 (0.63,0.77)	0.84 (0.46,1.53)	732	1.26 (1.17,1.36)	1.51 (0.96,2.36)	339	0.59 (0.53,0.66)	0.71 (0.52,0.97)	368	0.57 (0.51,0.62)	0.68 (0.44,1.05)
Heart Failure	244	0.44 (0.39,0.50)	479	0.85 (0.78,0.93)	1.92 (1.11,3.31)	555	0.96 (0.88,1.04)	2.15 (1.31,3.55)	202	0.35 (0.31,0.40)	0.80 (0.53,1.19)	210	0.32 (0.28,0.37)	0.73 (0.41,1.32)
Atrial fibrillation	265	0.49 (0.43,0.55)	515	0.92 (0.84,1.00)	1.90 (1.13,3.18)	380	0.66 (0.60,0.73)	1.36 (0.76,2.45)	393	0.69 (0.62,0.76)	1.43 (1.04,1.95)	207	0.32 (0.28,0.36)	0.65 (0.37,1.12)
Coronary Artery Disease	939	1.75 (1.64,1.86)	1,123	2.03 (1.92,2.15)	1.16 (0.81,1.66)	1,379	2.43 (2.30,2.56)	1.39 (1.02,1.90)	998	1.78 (1.67,1.90)	1.02 (0.85,1.23)	850	1.33 (1.24,1.42)	0.77 (0.54,1.09)
Deep vein thrombosis	72	0.13 (0.10,0.17)	0	NA	NA	0	NA	NA	62	0.11 (0.08,0.14)	0.82 (0.39,1.69)	33	0.05 (0.03,0.07)	0.37 (0.11,1.24)
Cardiovascular mortality	254	0.46 (0.41,0.52)	448	0.79 (0.72,0.87)	1.72 (0.94,3.17)	205	0.35 (0.31,0.40)	0.77 (0.33,1.77)	129	0.23 (0.19,0.27)	0.49 (0.29,0.82)	142	0.22 (0.18,0.26)	0.44 (0.22,0.90)
Chronic pulmonary disease	242	0.44 (0.39,0.50)	327	0.59 (0.52,0.65)	1.32 (0.66,2.63)	296	0.51 (0.46,0.58)	1.16 (0.59,2.29)	295	0.52 (0.46,0.58)	1.17 (0.83,1.66)	158	0.24 (0.21,0.29)	0.57 (0.32,1.02)
Acute respiratory distress syndrome	294	0.54 (0.48,0.60)	608	1.08 (1.00,1.17)	2.01 (1.21,3.36)	326	0.56 (0.50,0.63)	1.04 (0.54,2.01)	262	0.46 (0.40,0.52)	0.85 (0.59,1.23)	222	0.34 (0.30,0.39)	0.66 (0.39,1.12)
Seizure	196	0.36 (0.31,0.41)	811	1.46 (1.36,1.56)	4.06 (2.58,6.39)	586	1.02 (0.94,1.10)	2.84 (1.72,4.67)	160	0.28 (0.24,0.33)	0.78 (0.51,1.19)	67	0.10 (0.08,0.13)	0.29 (0.13,0.66)
End-stage renal disease	26	0.05 (0.03,0.07)	32	0.06 (0.04,0.08)	1.20 (0.28,5.21)	20	0.04 (0.02,0.05)	0.73 (0.10,5.51)	45	0.08 (0.06,0.10)	1.65 (0.63,4.29)	0	NA	NA
Acute kidney injury	97	0.18 (0.14,0.21)	46	0.08 (0.06,0.11)	0.46 (0.12,1.72)	99	0.17 (0.14,0.21)	0.97 (0.34,2.78)	87	0.15 (0.12,0.19)	0.86 (0.47,1.58)	37	0.06 (0.04,0.08)	0.32 (0.09,1.12)
Pancreatitis	109	0.20 (0.16,0.24)	157	0.28 (0.24,0.32)	1.41 (0.51,3.91)	122	0.21 (0.17,0.25)	1.07 (0.33,3.42)	96	0.17 (0.14,0.20)	0.85 (0.49,1.49)	61	0.09 (0.07,0.12)	0.45 (0.19,1.08)
All-cause mortality	1,801	3.27 (3.13,3.43)	3,698	6.55 (6.34,6.77)	2.00 (1.60,2.50)	1,651	2.84 (2.71,2.98)	0.87 (0.65,1.16)	1,208	2.11 (1.99,2.23)	0.64 (0.54,0.77)	743	1.14 (1.06,1.22)	0.35 (0.24,0.51)

Note: CI: confidence interval, REF: Reference group; Major CVD: Major Cardiovascular Disease; Composite outcome of stroke, heart failure and coronary artery disease

Hazard Ratio (HR) and 95% Confidence Interval (95% CI) were estimated by Cox regression, HR >1 (or <1) indicates patients with COVID-19 had higher (lower) risk of sequelae compared to the non-COVID-19 control cohort

Un-vaccinated, Incomplete vaccinated, Fully Vaccinated and Received booster dose of COVID-19 vaccines refers to patients who have received 0 dose, 1 doses, 2 doses and ≥3 doses of BioNtech or CoronaVac COVID-19 vaccines, respectively

Supplementary Table 15. Incidence rate and hazard ratio of clinical sequelae in COVID-19 and non-COVID-19 cohort after weighting during 0-30, 31-90, 91-180, 181-270, 271-365 days of SARS-CoV-2 infection (Patients with Charlson Co-morbidity Index <4; continued)

271-365 days

	Controls (REF)		COVID-19											
			Unvaccinated			Incompletely vaccinated			Fully vaccinated			Received booster dose		
**Clinical sequelae**	**Event**	**Incidence per 1,000 person-years**	**Event**	**Incidence per 1,000 person-years**	**Hazard ratio**	**Event**	**Incidence per 1,000 person-years**	**Hazard ratio**	**Event**	**Incidence per 1,000 person-years**	**Hazard ratio**	**Event**	**Incidence per 1,000 person-years**	**Hazard ratio**
Major CVD	1,270	4.09 (3.87,4.32)	1,301	3.93 (3.72,4.15)	1.01 (0.72,1.42)	1,626	5.44 (5.18,5.71)	1.32 (0.99,1.76)	1,169	3.81 (3.60,4.03)	0.93 (0.79,1.10)	1,178	4.14 (3.91,4.38)	1.00 (0.79,1.26)
Stroke	683	2.15 (1.99,2.32)	570	1.69 (1.55,1.83)	0.80 (0.47,1.35)	572	1.87 (1.72,2.03)	0.87 (0.53,1.41)	662	2.11 (1.95,2.27)	0.98 (0.78,1.23)	600	2.06 (1.90,2.23)	0.95 (0.68,1.33)
Myocardial infarction	256	0.80 (0.71,0.90)	208	0.61 (0.53,0.69)	0.82 (0.35,1.91)	435	1.41 (1.28,1.55)	1.74 (0.99,3.06)	180	0.57 (0.49,0.66)	0.71 (0.46,1.09)	198	0.67 (0.59,0.77)	0.83 (0.49,1.40)
Heart Failure	161	0.50 (0.43,0.58)	242	0.71 (0.62,0.80)	1.53 (0.72,3.24)	333	1.08 (0.97,1.20)	2.12 (1.11,4.06)	184	0.58 (0.50,0.67)	1.15 (0.75,1.77)	189	0.64 (0.55,0.74)	1.26 (0.65,2.45)
Atrial fibrillation	139	0.43 (0.37,0.51)	198	0.58 (0.50,0.66)	1.36 (0.60,3.06)	205	0.67 (0.58,0.76)	1.54 (0.66,3.59)	189	0.60 (0.52,0.69)	1.38 (0.88,2.14)	119	0.40 (0.34,0.48)	0.93 (0.46,1.87)
Coronary Artery Disease	533	1.69 (1.55,1.84)	525	1.56 (1.43,1.70)	0.99 (0.60,1.65)	759	2.50 (2.33,2.68)	1.46 (0.96,2.22)	376	1.21 (1.09,1.33)	0.71 (0.53,0.95)	426	1.48 (1.34,1.62)	0.86 (0.60,1.23)
Deep vein thrombosis	22	0.07 (0.04,0.10)	58	0.17 (0.13,0.22)	2.66 (0.47,14.92)	0	NA	NA	15	0.05 (0.03,0.07)	0.70 (0.15,3.18)	26	0.09 (0.06,0.13)	1.31 (0.30,5.72)
Cardiovascular mortality	26	0.08 (0.05,0.11)	81	0.24 (0.19,0.29)	2.25 (0.50,10.17)	0	NA	NA	7	0.02 (0.01,0.05)	0.29 (0.04,2.20)	0	NA	NA
Chronic pulmonary disease	169	0.53 (0.45,0.61)	201	0.59 (0.51,0.68)	1.20 (0.51,2.87)	234	0.76 (0.67,0.87)	1.43 (0.64,3.19)	180	0.57 (0.49,0.66)	1.08 (0.70,1.65)	92	0.32 (0.26,0.39)	0.59 (0.28,1.22)
Acute respiratory distress syndrome	135	0.42 (0.36,0.50)	163	0.48 (0.41,0.55)	1.23 (0.48,3.16)	277	0.90 (0.79,1.00)	2.09 (1.07,4.09)	161	0.51 (0.43,0.59)	1.20 (0.74,1.94)	90	0.31 (0.25,0.38)	0.71 (0.29,1.73)
Seizure	127	0.40 (0.33,0.47)	159	0.47 (0.40,0.54)	1.28 (0.53,3.07)	220	0.72 (0.63,0.82)	1.80 (0.82,3.94)	109	0.34 (0.29,0.42)	0.87 (0.52,1.44)	21	0.07 (0.05,0.11)	0.18 (0.04,0.75)
End-stage renal disease	12	0.04 (0.02,0.07)	42	0.12 (0.09,0.17)	3.60 (0.64,20.20)	0	NA	NA	4	0.01 (0.01,0.03)	0.37 (0.05,2.93)	0	NA	NA
Acute kidney injury	43	0.13 (0.10,0.18)	0	NA	NA	51	0.17 (0.13,0.22)	1.23 (0.28,5.29)	46	0.14 (0.11,0.19)	1.08 (0.47,2.49)	53	0.18 (0.14,0.23)	1.31 (0.46,3.71)
Pancreatitis	31	0.10 (0.07,0.14)	72	0.21 (0.16,0.26)	2.38 (0.55,10.21)	93	0.30 (0.25,0.37)	3.04 (0.84,11.02)	25	0.08 (0.05,0.11)	0.81 (0.27,2.37)	18	0.06 (0.04,0.09)	0.60 (0.08,4.45)
All-cause mortality	1,125	3.49 (3.29,3.70)	2,053	5.98 (5.72,6.24)	1.81 (1.39,2.36)	1,103	3.56 (3.35,3.77)	1.01 (0.72,1.42)	882	2.78 (2.60,2.96)	0.79 (0.65,0.96)	559	1.89 (1.74,2.06)	0.54 (0.37,0.77)

Note: CI: confidence interval, REF: Reference group; Major CVD: Major Cardiovascular Disease; Composite outcome of stroke, heart failure and coronary artery disease

Hazard Ratio (HR) and 95% Confidence Interval (95% CI) were estimated by Cox regression, HR >1 (or <1) indicates patients with COVID-19 had higher (lower) risk of sequelae compared to the non-COVID-19 control cohort

Un-vaccinated, Incomplete vaccinated, Fully Vaccinated and Received booster dose of COVID-19 vaccines refers to patients who have received 0 dose, 1 doses, 2 doses and ≥3 doses of BioNtech or CoronaVac COVID-19 vaccines, respectively

Supplementary Table 16. Incidence rate and hazard ratio of clinical sequelae in COVID-19 and non-COVID-19 cohort after weighting during 0-30, 31-90, 91-180, 181-270, 271-365 days of SARS-CoV-2 infection (Patients with Charlson Co-morbidity Index >=4)

0-30 days

	Controls (REF)		COVID-19												
			Unvaccinated			Incompletely vaccinated			Fully vaccinated			Received booster dose		
**Clinical sequelae**	**Event**	**Incidence per 1,000 person-years**	**Event**	**Incidence per 1,000 person-years**	**Hazard ratio**	**Event**	**Incidence per 1,000 person-years**	**Hazard ratio**	**Event**	**Incidence per 1,000 person-years**	**Hazard ratio**	**Event**	**Incidence per 1,000 person-years**	**Hazard ratio**
Major CVD	520	15.22 (13.94,16.56)	2,279	76.30 (73.20,79.47)	4.90 (4.06,5.91)	1,765	54.81 (52.30,57.41)	3.56 (2.82,4.49)	1,628	48.57 (46.25,50.97)	3.17 (2.58,3.90)	1,349	39.27 (37.21,41.40)	2.58 (2.04,3.26)
Stroke	345	8.49 (7.63,9.42)	1,338	37.34 (35.38,39.38)	4.29 (3.37,5.46)	1,042	27.21 (25.58,28.88)	3.17 (2.35,4.26)	1,214	30.45 (28.79,32.22)	3.57 (2.79,4.56)	925	22.57 (21.16,24.07)	2.66 (1.97,3.57)
Myocardial infarction	141	2.93 (2.47,3.44)	968	21.74 (20.40,23.14)	7.21 (5.34,9.72)	883	19.12 (17.90,20.43)	6.43 (4.51,9.15)	572	12.13 (11.17,13.16)	4.11 (2.86,5.90)	595	12.44 (11.47,13.47)	4.24 (2.93,6.13)
Heart Failure	202	4.33 (3.76,4.95)	744	17.30 (16.09,18.58)	3.95 (2.86,5.46)	537	12.03 (11.05,13.08)	2.76 (1.85,4.12)	452	9.92 (9.04,10.87)	2.28 (1.57,3.33)	247	5.30 (4.67,5.99)	1.22 (0.72,2.07)
Atrial fibrillation	96	2.09 (1.71,2.55)	357	8.43 (7.60,9.35)	3.97 (2.50,6.32)	352	7.99 (7.18,8.85)	3.79 (2.28,6.30)	295	6.54 (5.83,7.32)	3.12 (1.92,5.05)	381	8.32 (7.51,9.18)	3.97 (2.51,6.29)
Coronary Artery Disease	179	4.13 (3.56,4.77)	955	23.51 (22.07,25.05)	5.54 (4.15,7.41)	884	21.08 (19.72,22.50)	5.03 (3.58,7.07)	706	16.56 (15.36,17.81)	3.98 (2.89,5.49)	633	14.76 (13.66,15.96)	3.57 (2.51,5.06)
Deep vein thrombosis	26	0.52 (0.34,0.75)	90	1.95 (1.57,2.38)	3.69 (1.50,9.10)	138	2.92 (2.46,3.44)	5.56 (2.33,13.27)	103	2.13 (1.74,2.56)	4.06 (1.73,9.53)	63	1.27 (0.99,1.62)	2.44 (0.80,7.43)
Cardiovascular mortality	889	17.94 (16.79,19.15)	3,391	73.18 (70.73,75.66)	4.02 (3.47,4.64)	2,167	45.39 (43.50,47.32)	2.51 (2.06,3.06)	1,629	33.50 (31.91,35.16)	1.86 (1.52,2.28)	898	18.21 (17.05,19.43)	1.01 (0.76,1.36)
Chronic pulmonary disease	51	1.11 (0.85,1.46)	139	3.31 (2.79,3.89)	2.92 (1.49,5.71)	260	5.98 (5.28,6.73)	5.32 (2.82,10.05)	201	4.51 (3.91,5.16)	4.03 (2.13,7.63)	100	2.21 (1.82,2.69)	1.99 (0.90,4.39)
Acute respiratory distress syndrome	99	2.05 (1.68,2.49)	368	8.21 (7.40,9.08)	3.93 (2.43,6.35)	460	9.89 (9.01,10.82)	4.78 (2.97,7.67)	278	5.85 (5.20,6.58)	2.84 (1.75,4.61)	286	5.93 (5.27,6.64)	2.89 (1.78,4.70)
Seizure	40	0.81 (0.58,1.09)	289	6.38 (5.67,7.15)	7.78 (4.21,14.37)	142	3.02 (2.56,3.55)	3.72 (1.66,8.32)	90	1.86 (1.50,2.27)	2.30 (1.01,5.25)	92	1.88 (1.52,2.29)	2.33 (1.03,5.25)
End-stage renal disease	29	0.58 (0.39,0.82)	180	3.91 (3.36,4.50)	6.61 (2.72,16.03)	35	0.73 (0.51,1.00)	1.25 (0.27,5.76)	68	1.40 (1.09,1.76)	2.39 (0.85,6.75)	24	0.49 (0.31,0.70)	0.83 (0.17,3.98)
Acute kidney injury	64	1.33 (1.03,1.68)	489	10.92 (9.99,11.93)	8.14 (5.23,12.65)	386	8.29 (7.48,9.14)	6.21 (3.67,10.51)	269	5.64 (4.99,6.34)	4.24 (2.50,7.19)	307	6.34 (5.66,7.08)	4.77 (2.72,8.37)
Pancreatitis	24	0.48 (0.31,0.70)	77	1.67 (1.32,2.07)	3.46 (1.40,8.51)	15	0.31 (0.18,0.50)	0.64 (0.08,4.87)	56	1.15 (0.87,1.48)	2.41 (0.79,7.32)	35	0.71 (0.50,0.97)	1.48 (0.33,6.63)
All-cause mortality	4,802	96.95 (94.24,99.72)	55,938	1,207.12 (1,197.14,1,217.15)	12.30 (11.74,12.90)	31,336	656.38 (649.14,663.68)	6.73 (6.34,7.15)	17,987	369.86 (364.50,375.31)	3.80 (3.56,4.06)	8,035	163.02 (159.47,166.60)	1.68 (1.50,1.88)

Note: CI: confidence interval, REF: Reference group; Major CVD: Major Cardiovascular Disease; Composite outcome of stroke, heart failure and coronary artery disease
Hazard Ratio (HR) and 95% Confidence Interval (95% CI) were estimated by Cox regression, HR >1 (or <1) indicates patients with COVID-19 had higher (lower) risk of sequelae compared to the non-COVID-19 control cohort
Un-vaccinated, Incomplete vaccinated, Fully Vaccinated and Received booster dose of COVID-19 vaccines refers to patients who have received 0 dose, 1 doses, 2 doses and ≥3 doses of BioNtech or CoronaVac COVID-19 vaccines, respectively

Supplementary Table 16. Incidence rate and hazard ratio of clinical sequelae in COVID-19 and non-COVID-19 cohort after weighting during 0-30, 31-90, 91-180, 181-270, 271-365 days of SARS-CoV-2 infection (Patients with Charlson Co-morbidity Index >=4; continued)

31-90 days

	Controls (REF)		COVID-19											
			Unvaccinated			Incompletely vaccinated			Fully vaccinated			Received booster dose		
**Clinical sequelae**	**Event**	**Incidence per 1,000 person-years**	**Event**	**Incidence per 1,000 person-years**	**Hazard ratio**	**Event**	**Incidence per 1,000 person-years**	**Hazard ratio**	**Event**	**Incidence per 1,000 person-years**	**Hazard ratio**	**Event**	**Incidence per 1,000 person-years**	**Hazard ratio**
Major CVD	1,135	17.26 (16.27,18.28)	2,068	35.66 (34.14,37.21)	2.07 (1.71,2.49)	1,767	28.30 (26.99,29.63)	1.64 (1.32,2.03)	1,387	21.45 (20.34,22.59)	1.24 (1.01,1.52)	1,724	26.24 (25.02,27.50)	1.52 (1.25,1.85)
Stroke	685	8.76 (8.12,9.43)	1,211	17.39 (16.42,18.38)	1.99 (1.56,2.52)	977	13.16 (12.35,14.00)	1.50 (1.12,2.02)	784	10.22 (9.52,10.96)	1.17 (0.89,1.53)	785	10.04 (9.35,10.76)	1.15 (0.86,1.52)
Myocardial infarction	296	3.21 (2.87,3.60)	604	6.98 (6.44,7.56)	2.16 (1.58,2.97)	317	3.54 (3.17,3.95)	1.10 (0.66,1.82)	289	3.19 (2.84,3.57)	0.99 (0.65,1.52)	360	3.95 (3.56,4.38)	1.23 (0.80,1.88)
Heart Failure	530	5.92 (5.43,6.44)	1,250	14.95 (14.15,15.80)	2.53 (2.00,3.19)	879	10.14 (9.49,10.83)	1.71 (1.27,2.31)	755	8.59 (7.99,9.22)	1.45 (1.09,1.93)	938	10.56 (9.90,11.25)	1.78 (1.35,2.35)
Atrial fibrillation	281	3.18 (2.83,3.57)	652	7.91 (7.31,8.53)	2.48 (1.77,3.47)	391	4.58 (4.15,5.05)	1.44 (0.90,2.30)	385	4.44 (4.01,4.89)	1.39 (0.95,2.04)	274	3.14 (2.79,3.53)	0.99 (0.58,1.67)
Coronary Artery Disease	418	5.03 (4.57,5.54)	856	10.84 (10.13,11.58)	2.15 (1.61,2.86)	531	6.53 (5.99,7.10)	1.30 (0.87,1.93)	520	6.34 (5.82,6.91)	1.26 (0.91,1.75)	555	6.78 (6.23,7.36)	1.35 (0.96,1.88)
Deep vein thrombosis	57	0.60 (0.46,0.77)	151	1.69 (1.44,1.98)	2.81 (1.46,5.40)	157	1.70 (1.45,1.98)	2.83 (1.36,5.88)	184	1.98 (1.70,2.27)	3.29 (1.81,6.00)	43	0.46 (0.34,0.62)	0.77 (0.27,2.18)
Cardiovascular mortality	1,008	10.60 (9.96,11.27)	1,630	18.09 (17.22,18.98)	1.70 (1.41,2.05)	1,185	12.81 (12.10,13.56)	1.21 (0.93,1.56)	992	10.61 (9.97,11.29)	1.00 (0.78,1.28)	504	5.37 (4.92,5.86)	0.51 (0.35,0.73)
Chronic pulmonary disease	127	1.44 (1.20,1.70)	362	4.41 (3.97,4.88)	3.07 (1.95,4.81)	184	2.18 (1.88,2.51)	1.52 (0.78,2.96)	229	2.67 (2.34,3.03)	1.86 (1.11,3.12)	110	1.28 (1.06,1.54)	0.89 (0.42,1.90)
Acute respiratory distress syndrome	219	2.35 (2.06,2.69)	420	4.82 (4.37,5.29)	2.04 (1.32,3.16)	407	4.50 (4.09,4.96)	1.91 (1.17,3.13)	284	3.11 (2.76,3.49)	1.32 (0.85,2.05)	259	2.81 (2.48,3.16)	1.19 (0.76,1.86)
Seizure	100	1.06 (0.86,1.28)	235	2.67 (2.34,3.02)	2.51 (1.37,4.61)	87	0.95 (0.76,1.16)	0.90 (0.32,2.53)	164	1.77 (1.51,2.05)	1.67 (0.90,3.11)	64	0.69 (0.54,0.88)	0.65 (0.26,1.65)
End-stage renal disease	47	0.50 (0.37,0.66)	44	0.49 (0.36,0.65)	0.99 (0.40,2.47)	86	0.93 (0.76,1.15)	1.88 (0.70,5.00)	31	0.33 (0.23,0.46)	0.66 (0.19,2.24)	0	NA	NA
Acute kidney injury	143	1.53 (1.29,1.79)	337	3.86 (3.46,4.29)	2.51 (1.66,3.80)	221	2.45 (2.14,2.79)	1.60 (0.84,3.02)	166	1.81 (1.56,2.11)	1.18 (0.66,2.11)	189	2.05 (1.77,2.36)	1.34 (0.74,2.43)
Pancreatitis	49	0.51 (0.38,0.67)	112	1.26 (1.04,1.51)	2.45 (1.07,5.60)	99	1.07 (0.87,1.30)	2.09 (0.86,5.10)	90	0.97 (0.78,1.18)	1.88 (0.77,4.59)	84	0.90 (0.73,1.11)	1.76 (0.77,4.02)
All-cause mortality	6,352	66.82 (65.19,68.48)	16,748	185.88 (183.08,188.71)	2.77 (2.60,2.95)	9,559	103.30 (101.25,105.39)	1.54 (1.41,1.69)	7,152	76.51 (74.75,78.30)	1.14 (1.04,1.25)	5,052	53.84 (52.37,55.34)	0.80 (0.71,0.91)

Note: CI: confidence interval, REF: Reference group; Major CVD: Major Cardiovascular Disease; Composite outcome of stroke, heart failure and coronary artery disease
Hazard Ratio (HR) and 95% Confidence Interval (95% CI) were estimated by Cox regression, HR >1 (or <1) indicates patients with COVID-19 had higher (lower) risk of sequelae compared to the non-COVID-19 control cohort
Un-vaccinated, Incomplete vaccinated, Fully Vaccinated and Received booster dose of COVID-19 vaccines refers to patients who have received 0 dose, 1 doses, 2 doses and ≥3 doses of BioNtech or CoronaVac COVID-19 vaccines, respectively

Supplementary Table 16. Incidence rate and hazard ratio of clinical sequelae in COVID-19 and non-COVID-19 cohort after weighting during 0-30, 31-90, 91-180, 181-270, 271-365 days of SARS-CoV-2 infection (Patients with Charlson Co-morbidity Index >=4; continued)

91-180 days

	Controls (REF)		COVID-19											
			Unvaccinated			Incompletely vaccinated			Fully vaccinated			Received booster dose		
**Clinical sequelae**	**Event**	**Incidence per 1,000 person-years**	**Event**	**Incidence per 1,000 person-years**	**Hazard ratio**	**Event**	**Incidence per 1,000 person-years**	**Hazard ratio**	**Event**	**Incidence per 1,000 person-years**	**Hazard ratio**	**Event**	**Incidence per 1,000 person-years**	**Hazard ratio**
Major CVD	1,471	17.37 (16.50,18.28)	1,997	23.99 (22.96,25.06)	1.38 (1.14,1.67)	1,947	21.44 (20.51,22.41)	1.24 (1.00,1.53)	1,866	20.78 (19.86,21.75)	1.20 (1.01,1.43)	1,321	20.03 (18.97,21.13)	1.14 (0.92,1.42)
Stroke	951	9.45 (8.86,10.07)	1,252	12.54 (11.86,13.25)	1.33 (1.05,1.68)	1,238	11.47 (10.84,12.12)	1.22 (0.93,1.59)	1,076	10.12 (9.52,10.73)	1.07 (0.85,1.35)	619	7.95 (7.34,8.60)	0.84 (0.61,1.14)
Myocardial infarction	401	3.38 (3.06,3.73)	547	4.40 (4.05,4.79)	1.30 (0.92,1.83)	531	4.08 (3.75,4.44)	1.20 (0.81,1.76)	451	3.58 (3.27,3.93)	1.06 (0.74,1.51)	399	4.41 (3.99,4.86)	1.33 (0.87,2.03)
Heart Failure	532	4.61 (4.23,5.02)	1,090	9.09 (8.57,9.65)	1.97 (1.54,2.52)	799	6.33 (5.91,6.79)	1.37 (1.01,1.87)	936	7.68 (7.19,8.18)	1.67 (1.29,2.15)	666	7.53 (6.97,8.11)	1.63 (1.17,2.25)
Atrial fibrillation	356	3.13 (2.81,3.46)	507	4.29 (3.93,4.68)	1.37 (0.95,1.96)	634	5.10 (4.72,5.51)	1.62 (1.13,2.33)	492	4.09 (3.74,4.46)	1.30 (0.93,1.82)	398	4.57 (4.13,5.03)	1.50 (0.96,2.33)
Coronary Artery Disease	585	5.47 (5.04,5.92)	742	6.54 (6.09,7.03)	1.20 (0.89,1.60)	862	7.30 (6.83,7.80)	1.33 (0.98,1.81)	733	6.45 (6.00,6.93)	1.18 (0.90,1.55)	517	6.35 (5.83,6.92)	1.17 (0.82,1.65)
Deep vein thrombosis	67	0.55 (0.43,0.69)	103	0.80 (0.66,0.97)	1.46 (0.68,3.16)	83	0.62 (0.50,0.77)	1.13 (0.40,3.17)	35	0.27 (0.19,0.37)	0.49 (0.15,1.62)	45	0.49 (0.36,0.65)	0.91 (0.28,3.00)
Cardiovascular mortality	1,130	9.24 (8.71,9.79)	1,639	12.68 (12.07,13.30)	1.37 (1.13,1.66)	1,073	7.97 (7.51,8.46)	0.86 (0.66,1.12)	837	6.46 (6.03,6.90)	0.70 (0.54,0.90)	327	3.52 (3.15,3.91)	0.38 (0.24,0.61)
Chronic pulmonary disease	174	1.53 (1.32,1.78)	273	2.32 (2.06,2.61)	1.53 (0.94,2.47)	183	1.50 (1.29,1.73)	0.99 (0.50,1.96)	211	1.77 (1.55,2.03)	1.16 (0.69,1.95)	188	2.20 (1.91,2.54)	1.39 (0.76,2.52)
Acute respiratory distress syndrome	337	2.81 (2.52,3.12)	595	4.75 (4.37,5.14)	1.70 (1.16,2.48)	394	3.00 (2.72,3.31)	1.07 (0.66,1.75)	408	3.21 (2.91,3.53)	1.15 (0.80,1.64)	233	2.55 (2.24,2.90)	0.90 (0.56,1.44)
Seizure	145	1.20 (1.02,1.41)	411	3.26 (2.96,3.59)	2.75 (1.65,4.57)	213	1.61 (1.40,1.83)	1.36 (0.72,2.56)	178	1.39 (1.20,1.61)	1.16 (0.65,2.09)	80	0.86 (0.69,1.07)	0.68 (0.25,1.84)
End-stage renal disease	56	0.46 (0.35,0.59)	57	0.44 (0.34,0.57)	0.97 (0.37,2.57)	52	0.39 (0.29,0.50)	0.84 (0.24,3.00)	14	0.11 (0.07,0.18)	0.24 (0.03,1.75)	0	NA	NA
Acute kidney injury	243	2.02 (1.78,2.29)	371	2.96 (2.68,3.28)	1.47 (0.94,2.29)	411	3.12 (2.83,3.43)	1.55 (0.98,2.46)	322	2.53 (2.27,2.82)	1.25 (0.83,1.90)	82	0.90 (0.72,1.11)	0.44 (0.20,0.96)
Pancreatitis	64	0.52 (0.40,0.66)	109	0.85 (0.70,1.02)	1.65 (0.69,3.95)	86	0.64 (0.51,0.79)	1.25 (0.48,3.25)	91	0.70 (0.57,0.86)	1.36 (0.60,3.06)	25	0.27 (0.17,0.39)	0.49 (0.12,1.99)
All-cause mortality	8,012	65.51 (64.09,66.96)	13,116	101.46 (99.74,103.21)	1.55 (1.44,1.66)	9,167	68.14 (66.76,69.55)	1.04 (0.95,1.14)	7,184	55.43 (54.16,56.73)	0.85 (0.77,0.93)	3,614	38.87 (37.61,40.15)	0.59 (0.52,0.68)

Note: CI: confidence interval, REF: Reference group; Major CVD: Major Cardiovascular Disease; Composite outcome of stroke, heart failure and coronary artery disease
Hazard Ratio (HR) and 95% Confidence Interval (95% CI) were estimated by Cox regression, HR >1 (or <1) indicates patients with COVID-19 had higher (lower) risk of sequelae compared to the non-COVID-19 control cohort
Un-vaccinated, Incomplete vaccinated, Fully Vaccinated and Received booster dose of COVID-19 vaccines refers to patients who have received 0 dose, 1 doses, 2 doses and ≥3 doses of BioNtech or CoronaVac COVID-19 vaccines, respectively

Supplementary Table 16. Incidence rate and hazard ratio of clinical sequelae in COVID-19 and non-COVID-19 cohort after weighting during 0-30, 31-90, 91-180, 181-270, 271-365 days of SARS-CoV-2 infection (Patients with Charlson Co-morbidity Index >=4; continued)

181-270 days

	Controls (REF)		COVID-19											
			Unvaccinated			Incompletely vaccinated			Fully vaccinated			Received booster dose		
Clinical sequelae	Event	Incidence per 1,000 person-years	Event	Incidence per 1,000 person-years	Hazard ratio	Event	Incidence per 1,000 person-years	Hazard ratio	Event	Incidence per 1,000 person-years	Hazard ratio	Event	Incidence per 1,000 person-years	Hazard ratio
Major CVD	1,207	17.88 (16.89,18.91)	1,717	26.81 (25.55,28.09)	1.50 (1.24,1.81)	1,611	23.37 (22.25,24.54)	1.31 (1.05,1.62)	1,544	22.84 (21.72,24.00)	1.28 (1.06,1.53)	867	16.24 (15.20,17.36)	0.91 (0.62,1.35)
Stroke	705	8.79 (8.16,9.46)	957	12.46 (11.69,13.27)	1.42 (1.11,1.81)	884	10.80 (10.11,11.53)	1.23 (0.92,1.63)	854	10.66 (9.96,11.39)	1.21 (0.95,1.55)	579	9.21 (8.48,9.98)	1.06 (0.65,1.73)
Myocardial infarction	368	3.89 (3.51,4.30)	472	4.93 (4.50,5.39)	1.27 (0.90,1.78)	470	4.76 (4.35,5.21)	1.22 (0.84,1.78)	355	3.75 (3.38,4.16)	0.96 (0.66,1.40)	268	3.68 (3.25,4.13)	0.95 (0.43,2.12)
Heart Failure	527	5.72 (5.25,6.22)	998	10.81 (10.16,11.50)	1.89 (1.49,2.39)	818	8.55 (7.97,9.14)	1.49 (1.12,1.99)	644	7.03 (6.50,7.59)	1.23 (0.92,1.64)	335	4.68 (4.21,5.21)	0.82 (0.41,1.65)
Atrial fibrillation	278	3.06 (2.71,3.43)	410	4.50 (4.09,4.96)	1.47 (1.00,2.18)	333	3.53 (3.17,3.92)	1.16 (0.74,1.82)	421	4.65 (4.22,5.11)	1.52 (1.06,2.18)	212	3.02 (2.63,3.45)	0.98 (0.44,2.17)
Coronary Artery Disease	521	6.11 (5.59,6.64)	673	7.71 (7.15,8.31)	1.26 (0.94,1.70)	664	7.41 (6.87,8.00)	1.21 (0.87,1.69)	606	7.09 (6.54,7.67)	1.16 (0.88,1.54)	247	3.78 (3.33,4.28)	0.62 (0.32,1.21)
Deep vein thrombosis	42	0.43 (0.31,0.57)	93	0.94 (0.76,1.14)	2.19 (1.05,4.60)	80	0.79 (0.63,0.97)	1.83 (0.74,4.52)	84	0.86 (0.69,1.06)	2.02 (0.89,4.55)	25	0.33 (0.22,0.48)	0.78 (0.11,5.81)
Cardiovascular mortality	737	7.56 (7.03,8.12)	995	10.00 (9.39,10.63)	1.32 (1.06,1.66)	857	8.40 (7.85,8.97)	1.11 (0.85,1.47)	535	5.49 (5.03,5.96)	0.73 (0.53,0.99)	461	6.15 (5.61,6.73)	0.80 (0.39,1.67)
Chronic pulmonary disease	120	1.32 (1.11,1.58)	384	4.24 (3.83,4.68)	3.20 (2.04,5.02)	292	3.15 (2.80,3.52)	2.37 (1.41,3.98)	90	1.01 (0.82,1.24)	0.76 (0.34,1.70)	53	0.78 (0.59,1.01)	0.59 (0.14,2.48)
Acute respiratory distress syndrome	268	2.81 (2.49,3.16)	302	3.12 (2.79,3.49)	1.11 (0.70,1.77)	261	2.62 (2.32,2.96)	0.93 (0.57,1.53)	242	2.54 (2.23,2.87)	0.91 (0.58,1.41)	61	0.83 (0.65,1.07)	0.30 (0.09,0.95)
Seizure	122	1.26 (1.06,1.51)	317	3.26 (2.91,3.63)	2.59 (1.47,4.58)	155	1.54 (1.32,1.80)	1.23 (0.63,2.42)	107	1.11 (0.92,1.34)	0.88 (0.42,1.82)	65	0.87 (0.68,1.10)	0.66 (0.20,2.12)
End-stage renal disease	26	0.27 (0.18,0.39)	46	0.46 (0.34,0.61)	1.70 (0.56,5.17)	92	0.91 (0.74,1.11)	3.34 (1.32,8.40)	51	0.52 (0.39,0.68)	1.91 (0.68,5.36)	0	NA	NA
Acute kidney injury	206	2.15 (1.88,2.46)	329	3.41 (3.05,3.79)	1.59 (1.01,2.49)	222	2.23 (1.94,2.53)	1.04 (0.59,1.81)	199	2.08 (1.80,2.38)	0.96 (0.55,1.68)	76	1.03 (0.81,1.27)	0.47 (0.17,1.29)
Pancreatitis	65	0.67 (0.53,0.85)	109	1.10 (0.91,1.32)	1.64 (0.82,3.28)	47	0.47 (0.35,0.62)	0.69 (0.21,2.25)	58	0.59 (0.45,0.76)	0.89 (0.34,2.31)	66	0.88 (0.68,1.11)	1.35 (0.18,9.92)
All-cause mortality	6,356	65.13 (63.54,66.74)	9,496	95.43 (93.52,97.36)	1.47 (1.36,1.58)	7,038	69.01 (67.40,70.63)	1.06 (0.96,1.17)	4,314	44.27 (42.96,45.60)	0.68 (0.61,0.76)	2,798	37.38 (36.02,38.79)	0.57 (0.44,0.74)

Note: CI: confidence interval, REF: Reference group; Major CVD: Major Cardiovascular Disease; Composite outcome of stroke, heart failure and coronary artery disease
Hazard Ratio (HR) and 95% Confidence Interval (95% CI) were estimated by Cox regression, HR >1 (or <1) indicates patients with COVID-19 had higher (lower) risk of sequelae compared to the non-COVID-19 control cohort
Un-vaccinated, Incomplete vaccinated, Fully Vaccinated and Received booster dose of COVID-19 vaccines refers to patients who have received 0 dose, 1 doses, 2 doses and ≥3 doses of BioNtech or CoronaVac COVID-19 vaccines, respectively

Supplementary Table 16. Incidence rate and hazard ratio of clinical sequelae in COVID-19 and non-COVID-19 cohort after weighting during 0-30, 31-90, 91-180, 181-270, 271-365 days of SARS-CoV-2 infection (Patients with Charlson Co-morbidity Index >=4; continued)

271-365 days

	Controls (REF)		COVID-19											
			Unvaccinated			Incompletely vaccinated			Fully vaccinated			Received booster dose		
**Clinical sequelae**	**Event**	**Incidence per 1,000 person-years**	**Event**	**Incidence per 1,000 person-years**	**Hazard ratio**	**Event**	**Incidence per 1,000 person-years**	**Hazard ratio**	**Event**	**Incidence per 1,000 person-years**	**Hazard ratio**	**Event**	**Incidence per 1,000 person-years**	**Hazard ratio**
Major CVD	815	22.25 (20.76,23.81)	976	28.84 (27.08,30.70)	1.32 (1.03,1.69)	751	21.73 (20.22,23.33)	0.97 (0.72,1.30)	721	20.57 (19.10,22.10)	0.92 (0.70,1.20)	318	9.57 (8.57,10.68)	0.42 (0.21,0.84)
Stroke	412	9.48 (8.60,10.43)	525	12.98 (11.92,14.14)	1.38 (0.98,1.95)	348	8.51 (7.66,9.45)	0.89 (0.58,1.37)	336	8.09 (7.26,8.99)	0.85 (0.57,1.26)	245	6.22 (5.48,7.04)	0.65 (0.27,1.54)
Myocardial infarction	252	4.91 (4.33,5.55)	235	4.69 (4.13,5.33)	0.97 (0.63,1.51)	219	4.46 (3.89,5.07)	0.90 (0.53,1.51)	296	6.06 (5.40,6.78)	1.22 (0.80,1.87)	110	2.41 (1.99,2.89)	0.48 (0.12,1.87)
Heart Failure	414	8.27 (7.50,9.09)	591	12.20 (11.25,13.22)	1.50 (1.12,2.01)	585	12.28 (11.32,13.31)	1.48 (1.07,2.04)	492	10.38 (9.49,11.33)	1.25 (0.90,1.74)	158	3.51 (2.98,4.08)	0.42 (0.13,1.31)
Atrial fibrillation	166	3.37 (2.89,3.91)	219	4.58 (4.00,5.21)	1.39 (0.84,2.30)	197	4.21 (3.65,4.83)	1.24 (0.71,2.17)	142	3.01 (2.55,3.54)	0.89 (0.49,1.64)	299	6.76 (6.01,7.54)	1.99 (0.79,4.97)
Coronary Artery Disease	330	7.12 (6.38,7.91)	316	6.93 (6.20,7.73)	0.98 (0.64,1.50)	265	5.94 (5.26,6.69)	0.83 (0.51,1.36)	315	7.14 (6.39,7.96)	1.00 (0.67,1.49)	170	4.15 (3.57,4.82)	0.58 (0.22,1.53)
Deep vein thrombosis	35	0.65 (0.46,0.90)	72	1.39 (1.09,1.73)	2.16 (0.85,5.46)	26	0.52 (0.35,0.76)	0.79 (0.19,3.35)	15	0.29 (0.17,0.47)	0.44 (0.10,1.87)	0	0.00 (0.00,0.08)	NA
Cardiovascular mortality	71	1.33 (1.05,1.67)	82	1.58 (1.27,1.96)	1.13 (0.55,2.34)	44	0.87 (0.63,1.14)	0.66 (0.21,2.13)	0	0.00 (0.00,0.07)	NA	0	0.00 (0.00,0.08)	NA
Chronic pulmonary disease	119	2.42 (2.02,2.89)	145	3.05 (2.58,3.58)	1.28 (0.70,2.35)	201	4.34 (3.77,4.97)	1.77 (0.98,3.20)	175	3.78 (3.24,4.36)	1.55 (0.85,2.82)	20	0.47 (0.30,0.71)	0.19 (0.03,1.37)
Acute respiratory distress syndrome	143	2.75 (2.34,3.24)	177	3.51 (3.02,4.06)	1.29 (0.71,2.34)	223	4.51 (3.95,5.13)	1.62 (0.93,2.81)	141	2.86 (2.41,3.35)	1.03 (0.56,1.90)	125	2.70 (2.26,3.21)	0.97 (0.39,2.44)
Seizure	54	1.03 (0.79,1.34)	104	2.05 (1.69,2.48)	2.00 (0.91,4.39)	90	1.79 (1.45,2.19)	1.73 (0.76,3.90)	34	0.67 (0.47,0.93)	0.64 (0.22,1.85)	61	1.30 (1.01,1.67)	1.24 (0.28,5.48)
End-stage renal disease	28	0.52 (0.35,0.74)	102	1.96 (1.61,2.37)	3.85 (1.27,11.69)	44	0.87 (0.63,1.15)	1.63 (0.47,5.67)	0	0.00 (0.00,0.07)	NA	20	0.42 (0.26,0.63)	0.79 (0.10,5.96)
Acute kidney injury	108	2.07 (1.70,2.48)	117	2.31 (1.91,2.75)	1.13 (0.61,2.08)	38	0.77 (0.56,1.05)	0.37 (0.12,1.16)	53	1.08 (0.82,1.40)	0.51 (0.18,1.41)	344	7.36 (6.61,8.17)	3.37 (0.48,23.76)
Pancreatitis	46	0.88 (0.65,1.16)	88	1.71 (1.38,2.10)	1.97 (0.81,4.78)	24	0.48 (0.32,0.71)	0.54 (0.13,2.29)	9	0.18 (0.08,0.31)	0.20 (0.03,1.47)	0	0.00 (0.00,0.08)	NA
All-cause mortality	3,506	66.16 (64.01,68.39)	5,671	108.99 (106.19,111.86)	1.67 (1.51,1.84)	3,015	59.54 (57.45,61.70)	0.89 (0.78,1.03)	2,596	51.59 (49.63,53.60)	0.78 (0.67,0.90)	814	17.24 (16.08,18.45)	0.26 (0.16,0.42)

Note: CI: confidence interval, REF: Reference group; Major CVD: Major Cardiovascular Disease; Composite outcome of stroke, heart failure and coronary artery disease
Hazard Ratio (HR) and 95% Confidence Interval (95% CI) were estimated by Cox regression, HR >1 (or <1) indicates patients with COVID-19 had higher (lower) risk of sequelae compared to the non-COVID-19 control cohort
Un-vaccinated, Incomplete vaccinated, Fully Vaccinated and Received booster dose of COVID-19 vaccines refers to patients who have received 0 dose, 1 doses, 2 doses and ≥3 doses of BioNtech or CoronaVac COVID-19 vaccines, respectively

Supplementary Table 17. Incidence rate and hazard ratio of clinical sequelae in COVID-19 and non-COVID-19 cohort after weighting during 0-30, 31-90, 91-180, 181-270, 271-365 days of SARS-CoV-2 infection (Male patients)

0-30 days

	Controls (REF)		COVID-19												
			Unvaccinated			Incompletely vaccinated			Fully vaccinated			Received booster dose		
**Clinical sequelae**	**Event**	**Incidence per 1,000 person-years**	**Event**	**Incidence per 1,000 person-years**	**Hazard ratio**	**Event**	**Incidence per 1,000 person-years**	**Hazard ratio**	**Event**	**Incidence per 1,000 person-years**	**Hazard ratio**	**Event**	**Incidence per 1,000 person-years**	**Hazard ratio**
Major CVD	739	5.72 (5.31,6.14)	3,252	26.08 (25.19,26.98)	4.54 (3.69,5.57)	1,983	15.59 (14.92,16.29)	2.72 (2.07,3.57)	1,849	14.37 (13.72,15.03)	2.51 (2.11,2.99)	1,486	11.45 (10.88,12.05)	2.00 (1.69,2.38)
Stroke	431	3.15 (2.86,3.45)	1,776	13.52 (12.90,14.16)	4.26 (3.22,5.64)	1,317	9.82 (9.30,10.36)	3.11 (2.25,4.30)	1,304	9.57 (9.07,10.11)	3.04 (2.45,3.77)	864	6.26 (5.85,6.68)	1.99 (1.57,2.51)
Myocardial infarction	202	1.42 (1.24,1.63)	1,083	7.84 (7.39,8.32)	5.47 (3.92,7.65)	782	5.60 (5.22,6.00)	3.92 (2.52,6.10)	607	4.31 (3.98,4.66)	3.02 (2.18,4.19)	572	4.02 (3.70,4.36)	2.83 (2.09,3.82)
Heart Failure	122	0.86 (0.71,1.02)	694	5.05 (4.68,5.43)	5.85 (3.82,8.96)	358	2.56 (2.31,2.84)	2.98 (1.68,5.27)	281	1.99 (1.77,2.24)	2.32 (1.46,3.68)	239	1.68 (1.47,1.90)	1.95 (1.22,3.11)
Atrial fibrillation	86	0.61 (0.50,0.75)	288	2.11 (1.88,2.36)	3.43 (1.84,6.39)	285	2.06 (1.83,2.31)	3.36 (1.73,6.52)	218	1.56 (1.36,1.77)	2.54 (1.50,4.31)	230	1.63 (1.43,1.85)	2.66 (1.69,4.20)
Coronary Artery Disease	329	2.43 (2.18,2.71)	1,278	9.60 (9.09,10.14)	3.93 (2.87,5.37)	817	6.08 (5.67,6.51)	2.49 (1.64,3.80)	838	6.21 (5.80,6.64)	2.55 (1.95,3.33)	700	5.16 (4.79,5.56)	2.12 (1.64,2.74)
Deep vein thrombosis	12	0.08 (0.04,0.14)	121	0.87 (0.72,1.03)	10.71 (3.39,33.81)	87	0.62 (0.49,0.75)	7.61 (2.24,25.89)	34	0.24 (0.17,0.33)	2.99 (0.76,11.73)	33	0.23 (0.16,0.31)	2.82 (0.83,9.59)
Cardiovascular mortality	591	4.12 (3.80,4.46)	3,671	26.29 (25.45,27.15)	6.34 (5.27,7.62)	1,683	11.91 (11.35,12.49)	2.88 (2.21,3.76)	1,228	8.62 (8.15,9.11)	2.09 (1.67,2.61)	555	3.85 (3.54,4.18)	0.93 (0.69,1.25)
Chronic pulmonary disease	77	0.55 (0.44,0.69)	183	1.36 (1.17,1.56)	2.46 (1.17,5.14)	442	3.22 (2.93,3.53)	5.86 (3.07,11.15)	234	1.69 (1.48,1.91)	3.06 (1.83,5.12)	125	0.89 (0.75,1.06)	1.62 (0.92,2.86)
Acute respiratory distress syndrome	126	0.89 (0.74,1.05)	899	6.51 (6.09,6.95)	7.28 (4.84,10.95)	469	3.35 (3.06,3.66)	3.76 (2.25,6.27)	288	2.04 (1.81,2.28)	2.29 (1.47,3.59)	250	1.75 (1.54,1.98)	1.97 (1.28,3.05)
Seizure	59	0.41 (0.32,0.53)	290	2.12 (1.88,2.37)	5.11 (2.56,10.18)	417	2.99 (2.71,3.29)	7.24 (3.63,14.43)	139	0.98 (0.83,1.16)	2.39 (1.30,4.37)	181	1.26 (1.09,1.45)	3.06 (1.34,6.96)
End-stage renal disease	17	0.12 (0.07,0.18)	84	0.60 (0.48,0.74)	5.09 (1.98,13.09)	12	0.08 (0.04,0.14)	0.70 (0.09,5.45)	56	0.39 (0.30,0.50)	3.33 (1.21,9.22)	13	0.09 (0.05,0.15)	0.78 (0.17,3.53)
Acute kidney injury	42	0.30 (0.22,0.40)	411	2.97 (2.69,3.27)	9.99 (5.55,17.98)	569	4.06 (3.74,4.40)	13.70 (7.44,25.21)	190	1.34 (1.16,1.54)	4.54 (2.44,8.45)	154	1.07 (0.92,1.26)	3.63 (1.97,6.71)
Pancreatitis	22	0.15 (0.10,0.23)	158	1.13 (0.96,1.32)	7.33 (2.66,20.14)	116	0.82 (0.68,0.98)	5.32 (1.44,19.68)	67	0.47 (0.37,0.60)	3.06 (1.15,8.10)	100	0.70 (0.57,0.85)	4.53 (2.13,9.63)
All-cause mortality	2,939	20.50 (19.77,21.25)	56,318	403.37 (400.04,406.71)	19.59 (18.45,20.81)	27,226	192.67 (190.39,194.97)	9.38 (8.69,10.13)	12,213	85.67 (84.16,87.20)	4.18 (3.86,4.52)	4,792	33.25 (32.32,34.20)	1.62 (1.41,1.86)

Note: CI: confidence interval, REF: Reference group; Major CVD: Major Cardiovascular Disease; Composite outcome of stroke, heart failure and coronary artery disease

Hazard Ratio (HR) and 95% Confidence Interval (95% CI) were estimated by Cox regression, HR >1 (or <1) indicates patients with COVID-19 had higher (lower) risk of sequelae compared to the non-COVID-19 control cohort

Un-vaccinated, Incomplete vaccinated, Fully Vaccinated and Received booster dose of COVID-19 vaccines refers to patients who have received 0 dose, 1 doses, 2 doses and ≥3 doses of BioNtech or CoronaVac COVID-19 vaccines, respectively

Supplementary Table 17. Incidence rate and hazard ratio of clinical sequelae in COVID-19 and non-COVID-19 cohort after weighting during 0-30, 31-90, 91-180, 181-270, 271-365 days of SARS-CoV-2 infection (Male patients; continued)

30-90 days

	Controls (REF)		COVID-19											
			Unvaccinated			Incompletely vaccinated			Fully vaccinated			Received booster dose		
**Clinical sequelae**	**Event**	**Incidence per 1,000 person-years**	**Event**	**Incidence per 1,000 person-years**	**Hazard ratio**	**Event**	**Incidence per 1,000 person-years**	**Hazard ratio**	**Event**	**Incidence per 1,000 person-years**	**Hazard ratio**	**Event**	**Incidence per 1,000 person-years**	**Hazard ratio**
Major CVD	1,875	7.41 (7.08,7.75)	2,798	11.54 (11.11,11.97)	1.56 (1.27,1.92)	2,505	10.06 (9.67,10.46)	1.36 (1.08,1.71)	1,868	7.41 (7.08,7.76)	1.00 (0.86,1.17)	2,012	7.94 (7.61,8.30)	1.07 (0.93,1.23)
Stroke	959	3.57 (3.35,3.80)	1,355	5.29 (5.02,5.58)	1.48 (1.11,1.98)	1,473	5.60 (5.32,5.89)	1.57 (1.16,2.12)	1,002	3.76 (3.53,3.99)	1.05 (0.84,1.31)	818	3.04 (2.84,3.25)	0.85 (0.69,1.05)
Myocardial infarction	490	1.77 (1.61,1.93)	537	1.99 (1.83,2.17)	1.13 (0.73,1.76)	497	1.82 (1.66,1.98)	1.03 (0.61,1.74)	489	1.77 (1.62,1.94)	1.00 (0.74,1.36)	530	1.91 (1.75,2.08)	1.08 (0.81,1.43)
Heart Failure	348	1.26 (1.13,1.39)	1,027	3.83 (3.60,4.07)	3.05 (2.24,4.15)	523	1.91 (1.75,2.08)	1.52 (0.98,2.37)	471	1.71 (1.56,1.87)	1.36 (0.99,1.88)	451	1.62 (1.48,1.78)	1.29 (0.93,1.79)
Atrial fibrillation	233	0.85 (0.74,0.96)	600	2.26 (2.08,2.44)	2.66 (1.72,4.13)	277	1.02 (0.91,1.15)	1.21 (0.64,2.29)	354	1.29 (1.16,1.43)	1.53 (1.06,2.20)	261	0.95 (0.84,1.07)	1.12 (0.75,1.67)
Coronary Artery Disease	893	3.37 (3.16,3.60)	1,334	5.15 (4.88,5.43)	1.53 (1.14,2.05)	1,027	3.90 (3.67,4.15)	1.16 (0.81,1.66)	844	3.19 (2.98,3.41)	0.95 (0.75,1.19)	1,134	4.29 (4.04,4.54)	1.27 (1.05,1.54)
Deep vein thrombosis	41	0.15 (0.11,0.19)	274	1.01 (0.90,1.13)	6.92 (3.26,14.69)	40	0.14 (0.10,0.19)	0.99 (0.23,4.29)	115	0.41 (0.34,0.49)	2.83 (1.41,5.69)	58	0.21 (0.16,0.27)	1.42 (0.61,3.32)
Cardiovascular mortality	742	2.65 (2.46,2.84)	1,004	3.68 (3.46,3.92)	1.39 (1.03,1.88)	880	3.18 (2.97,3.39)	1.20 (0.86,1.68)	702	2.52 (2.33,2.71)	0.95 (0.72,1.25)	385	1.37 (1.24,1.51)	0.52 (0.35,0.77)
Chronic pulmonary disease	192	0.70 (0.61,0.81)	738	2.80 (2.60,3.01)	3.98 (2.61,6.08)	412	1.54 (1.39,1.69)	2.19 (1.17,4.10)	216	0.79 (0.69,0.90)	1.13 (0.71,1.80)	194	0.71 (0.62,0.82)	1.01 (0.64,1.59)
Acute respiratory distress syndrome	320	1.15 (1.03,1.28)	510	1.89 (1.73,2.06)	1.64 (1.00,2.70)	497	1.81 (1.65,1.97)	1.57 (0.96,2.58)	389	1.40 (1.27,1.55)	1.22 (0.85,1.76)	418	1.50 (1.36,1.65)	1.30 (0.89,1.91)
Seizure	161	0.58 (0.49,0.67)	304	1.14 (1.02,1.27)	1.98 (1.00,3.90)	237	0.87 (0.76,0.98)	1.50 (0.68,3.32)	224	0.81 (0.71,0.92)	1.40 (0.89,2.22)	100	0.36 (0.29,0.44)	0.62 (0.32,1.21)
End-stage renal disease	28	0.10 (0.07,0.14)	70	0.26 (0.20,0.33)	2.58 (0.63,10.54)	141	0.51 (0.43,0.60)	5.08 (1.80,14.34)	22	0.08 (0.05,0.12)	0.78 (0.16,3.70)	14	0.05 (0.03,0.08)	0.48 (0.11,2.13)
Acute kidney injury	102	0.37 (0.30,0.44)	385	1.43 (1.29,1.57)	3.87 (2.18,6.90)	353	1.28 (1.15,1.42)	3.50 (1.84,6.64)	114	0.41 (0.34,0.49)	1.12 (0.60,2.11)	118	0.42 (0.35,0.51)	1.15 (0.61,2.18)
Pancreatitis	71	0.25 (0.20,0.32)	240	0.88 (0.77,1.00)	3.49 (1.55,7.84)	149	0.54 (0.46,0.63)	2.14 (0.82,5.59)	60	0.22 (0.17,0.28)	0.85 (0.35,2.06)	97	0.35 (0.28,0.42)	1.36 (0.73,2.55)
All-cause mortality	4,237	15.11 (14.66,15.57)	15,906	58.38 (57.48,59.29)	3.85 (3.54,4.19)	7,648	27.62 (27.01,28.25)	1.83 (1.62,2.06)	4,642	16.65 (16.17,17.13)	1.10 (0.99,1.23)	3,532	12.58 (12.17,13.00)	0.83 (0.71,0.97)

Note: CI: confidence interval, REF: Reference group; Major CVD: Major Cardiovascular Disease; Composite outcome of stroke, heart failure and coronary artery disease
Hazard Ratio (HR) and 95% Confidence Interval (95% CI) were estimated by Cox regression, HR >1 (or <1) indicates patients with COVID-19 had higher (lower) risk of sequelae compared to the non-COVID-19 control cohort
Un-vaccinated, Incomplete vaccinated, Fully Vaccinated and Received booster dose of COVID-19 vaccines refers to patients who have received 0 dose, 1 doses, 2 doses and ≥3 doses of BioNtech or CoronaVac COVID-19 vaccines, respectively

Supplementary Table 17. Incidence rate and hazard ratio of clinical sequelae in COVID-19 and non-COVID-19 cohort after weighting during 0-30, 31-90, 91-180, 181-270, 271-365 days of SARS-CoV-2 infection (Male patients; continued)

91-180 days

	Controls (REF)		COVID-19											
			Unvaccinated			Incompletely vaccinated			Fully vaccinated			Received booster dose		
**Clinical sequelae**	**Event**	**Incidence per 1,000 person-years**	**Event**	**Incidence per 1,000 person-years**	**Hazard ratio**	**Event**	**Incidence per 1,000 person-years**	**Hazard ratio**	**Event**	**Incidence per 1,000 person-years**	**Hazard ratio**	**Event**	**Incidence per 1,000 person-years**	**Hazard ratio**
Major CVD	2,475	7.54 (7.25,7.84)	3,615	10.63 (10.29,10.98)	1.42 (1.17,1.72)	3,296	9.08 (8.77,9.39)	1.21 (0.99,1.49)	2,908	8.04 (7.76,8.34)	1.07 (0.95,1.22)	1,924	6.81 (6.51,7.11)	0.89 (0.78,1.02)
Stroke	1,328	3.82 (3.62,4.03)	1,856	5.16 (4.93,5.40)	1.36 (1.04,1.78)	1,714	4.47 (4.26,4.68)	1.18 (0.89,1.57)	1,576	4.12 (3.92,4.33)	1.09 (0.91,1.29)	956	3.19 (3.00,3.40)	0.83 (0.68,1.00)
Myocardial infarction	653	1.81 (1.68,1.96)	689	1.82 (1.68,1.96)	1.00 (0.66,1.53)	580	1.45 (1.34,1.57)	0.80 (0.50,1.28)	671	1.70 (1.57,1.83)	0.94 (0.72,1.22)	611	1.98 (1.83,2.15)	1.09 (0.84,1.42)
Heart Failure	392	1.09 (0.99,1.20)	1,299	3.44 (3.26,3.63)	3.18 (2.35,4.31)	839	2.10 (1.96,2.25)	1.95 (1.31,2.91)	624	1.58 (1.46,1.71)	1.47 (1.10,1.95)	328	1.06 (0.95,1.18)	0.96 (0.67,1.36)
Atrial fibrillation	340	0.95 (0.85,1.06)	458	1.22 (1.11,1.34)	1.28 (0.79,2.08)	618	1.56 (1.44,1.69)	1.63 (1.05,2.53)	543	1.38 (1.27,1.50)	1.45 (1.07,1.96)	383	1.25 (1.13,1.38)	1.33 (0.90,1.96)
Coronary Artery Disease	1,181	3.43 (3.24,3.63)	1,455	3.99 (3.79,4.20)	1.16 (0.87,1.56)	1,589	4.14 (3.94,4.35)	1.21 (0.91,1.61)	1,326	3.50 (3.31,3.69)	1.02 (0.85,1.23)	1,009	3.43 (3.22,3.64)	0.99 (0.82,1.21)
Deep vein thrombosis	66	0.18 (0.14,0.23)	42	0.11 (0.08,0.15)	0.60 (0.14,2.59)	78	0.19 (0.16,0.24)	1.06 (0.32,3.45)	43	0.11 (0.08,0.14)	0.58 (0.20,1.67)	107	0.34 (0.28,0.42)	1.95 (0.79,4.82)
Cardiovascular mortality	885	2.43 (2.28,2.60)	1,249	3.26 (3.08,3.44)	1.33 (1.01,1.77)	722	1.79 (1.66,1.92)	0.73 (0.49,1.09)	706	1.77 (1.64,1.90)	0.72 (0.56,0.94)	208	0.67 (0.58,0.76)	0.28 (0.17,0.45)
Chronic pulmonary disease	268	0.75 (0.67,0.85)	577	1.56 (1.43,1.69)	2.08 (1.30,3.34)	430	1.10 (1.00,1.21)	1.47 (0.83,2.61)	347	0.89 (0.80,0.99)	1.19 (0.82,1.73)	286	0.94 (0.84,1.05)	1.22 (0.79,1.89)
Acute respiratory distress syndrome	426	1.18 (1.07,1.30)	814	2.15 (2.00,2.30)	1.81 (1.21,2.72)	490	1.22 (1.12,1.33)	1.03 (0.61,1.73)	536	1.35 (1.24,1.47)	1.14 (0.84,1.54)	389	1.25 (1.13,1.38)	1.07 (0.75,1.54)
Seizure	209	0.58 (0.50,0.66)	649	1.73 (1.60,1.86)	3.02 (1.90,4.82)	448	1.12 (1.02,1.23)	1.98 (1.13,3.48)	199	0.50 (0.44,0.58)	0.88 (0.56,1.40)	198	0.64 (0.55,0.73)	1.07 (0.50,2.30)
End-stage renal disease	26	0.07 (0.05,0.10)	77	0.20 (0.16,0.25)	2.89 (0.66,12.73)	167	0.41 (0.36,0.48)	5.99 (2.02,17.82)	17	0.04 (0.03,0.07)	0.62 (0.14,2.77)	0	NA	NA
Acute kidney injury	190	0.52 (0.45,0.60)	344	0.91 (0.81,1.01)	1.73 (0.99,3.03)	376	0.94 (0.84,1.03)	1.79 (1.05,3.03)	251	0.63 (0.56,0.71)	1.20 (0.76,1.90)	73	0.24 (0.19,0.30)	0.45 (0.22,0.91)
Pancreatitis	95	0.26 (0.21,0.32)	102	0.27 (0.22,0.32)	1.02 (0.37,2.81)	146	0.36 (0.31,0.42)	1.37 (0.46,4.11)	104	0.26 (0.22,0.32)	0.99 (0.50,1.97)	69	0.22 (0.18,0.28)	0.86 (0.39,1.88)
All-cause mortality	5,495	15.10 (14.71,15.51)	12,004	31.30 (30.74,31.86)	2.07 (1.88,2.28)	7,195	17.80 (17.39,18.21)	1.18 (1.04,1.34)	4,760	11.91 (11.57,12.25)	0.79 (0.71,0.88)	3,271	10.50 (10.14,10.86)	0.69 (0.59,0.82)

Note: CI: confidence interval, REF: Reference group; Major CVD: Major Cardiovascular Disease; Composite outcome of stroke, heart failure and coronary artery disease
Hazard Ratio (HR) and 95% Confidence Interval (95% CI) were estimated by Cox regression, HR >1 (or <1) indicates patients with COVID-19 had higher (lower) risk of sequelae compared to the non-COVID-19 control cohort
Un-vaccinated, Incomplete vaccinated, Fully Vaccinated and Received booster dose of COVID-19 vaccines refers to patients who have received 0 dose, 1 doses, 2 doses and ≥3 doses of BioNtech or CoronaVac COVID-19 vaccines, respectively

Supplementary Table 17. Incidence rate and hazard ratio of clinical sequelae in COVID-19 and non-COVID-19 cohort after weighting during 0-30, 31-90, 91-180, 181-270, 271-365 days of SARS-CoV-2 infection (Male patients; continued)

181-270 days

	Controls (REF)		COVID-19											
			Unvaccinated			Incompletely vaccinated			Fully vaccinated			Received booster dose		
Clinical sequelae	Event	Incidence per 1,000 person-years	Event	Incidence per 1,000 person-years	Hazard ratio	Event	Incidence per 1,000 person-years	Hazard ratio	Event	Incidence per 1,000 person-years	Hazard ratio	Event	Incidence per 1,000 person-years	Hazard ratio
Major CVD	2,056	7.92 (7.58,8.27)	2,804	10.80 (10.40,11.20)	1.36 (1.11,1.67)	2,578	9.46 (9.10,9.83)	1.19 (0.97,1.48)	2,131	7.88 (7.55,8.22)	0.99 (0.87,1.14)	1,545	6.76 (6.43,7.11)	0.85 (0.71,1.03)
Stroke	1,040	3.78 (3.55,4.01)	1,565	5.70 (5.42,5.99)	1.51 (1.15,1.98)	1,096	3.81 (3.59,4.04)	1.01 (0.73,1.40)	1,070	3.74 (3.52,3.97)	0.99 (0.82,1.20)	661	2.72 (2.52,2.93)	0.72 (0.54,0.95)
Myocardial infarction	583	2.05 (1.89,2.22)	571	1.97 (1.81,2.14)	0.96 (0.64,1.45)	744	2.48 (2.31,2.66)	1.21 (0.82,1.78)	432	1.46 (1.33,1.61)	0.71 (0.53,0.95)	537	2.14 (1.97,2.33)	1.05 (0.62,1.78)
Heart Failure	388	1.36 (1.23,1.51)	796	2.76 (2.57,2.96)	2.02 (1.41,2.89)	755	2.52 (2.34,2.70)	1.85 (1.27,2.69)	457	1.55 (1.41,1.69)	1.13 (0.84,1.54)	257	1.02 (0.91,1.16)	0.75 (0.46,1.23)
Atrial fibrillation	277	0.98 (0.87,1.10)	351	1.23 (1.11,1.36)	1.26 (0.72,2.19)	299	1.01 (0.90,1.12)	1.03 (0.57,1.85)	375	1.28 (1.15,1.41)	1.31 (0.94,1.82)	160	0.64 (0.55,0.75)	0.65 (0.36,1.15)
Coronary Artery Disease	1,030	3.79 (3.56,4.02)	1,147	4.11 (3.88,4.36)	1.09 (0.80,1.47)	1,325	4.60 (4.36,4.85)	1.21 (0.91,1.62)	1,030	3.63 (3.42,3.86)	0.96 (0.79,1.16)	869	3.64 (3.40,3.88)	0.96 (0.75,1.23)
Deep vein thrombosis	44	0.15 (0.11,0.21)	46	0.16 (0.12,0.21)	1.02 (0.33,3.09)	46	0.15 (0.11,0.20)	0.97 (0.33,2.88)	69	0.23 (0.18,0.29)	1.48 (0.69,3.15)	48	0.19 (0.14,0.25)	1.24 (0.43,3.56)
Cardiovascular mortality	535	1.86 (1.71,2.02)	898	3.06 (2.87,3.27)	1.65 (1.20,2.26)	588	1.94 (1.78,2.10)	1.04 (0.70,1.55)	311	1.04 (0.93,1.16)	0.56 (0.39,0.81)	207	0.82 (0.71,0.93)	0.43 (0.25,0.76)
Chronic pulmonary disease	227	0.81 (0.71,0.92)	489	1.72 (1.58,1.88)	2.14 (1.32,3.47)	345	1.17 (1.05,1.30)	1.45 (0.83,2.55)	208	0.71 (0.62,0.81)	0.88 (0.57,1.38)	84	0.34 (0.27,0.42)	0.42 (0.21,0.86)
Acute respiratory distress syndrome	346	1.21 (1.09,1.34)	389	1.34 (1.21,1.48)	1.11 (0.66,1.87)	420	1.39 (1.26,1.53)	1.15 (0.69,1.91)	279	0.94 (0.83,1.05)	0.78 (0.54,1.12)	200	0.80 (0.69,0.91)	0.66 (0.22,2.02)
Seizure	180	0.63 (0.54,0.72)	629	2.19 (2.02,2.37)	3.49 (2.22,5.47)	484	1.62 (1.48,1.77)	2.57 (1.48,4.46)	185	0.62 (0.54,0.72)	0.99 (0.63,1.55)	160	0.63 (0.54,0.73)	1.00 (0.34,2.97)
End-stage renal disease	31	0.11 (0.07,0.15)	36	0.12 (0.09,0.17)	1.12 (0.24,5.25)	50	0.16 (0.12,0.21)	1.52 (0.48,4.79)	50	0.17 (0.12,0.22)	1.53 (0.61,3.88)	0	NA	NA
Acute kidney injury	157	0.55 (0.47,0.64)	240	0.83 (0.73,0.94)	1.51 (0.84,2.71)	167	0.55 (0.47,0.64)	1.01 (0.49,2.07)	156	0.52 (0.45,0.61)	0.95 (0.57,1.60)	73	0.29 (0.23,0.36)	0.52 (0.22,1.21)
Pancreatitis	83	0.29 (0.23,0.36)	113	0.39 (0.32,0.46)	1.34 (0.50,3.56)	115	0.38 (0.32,0.46)	1.32 (0.45,3.88)	58	0.20 (0.15,0.25)	0.68 (0.31,1.48)	39	0.15 (0.11,0.21)	0.52 (0.19,1.45)
All-cause mortality	4,316	14.99 (14.55,15.45)	8,882	30.29 (29.67,30.93)	2.02 (1.82,2.24)	5,229	17.22 (16.76,17.69)	1.15 (1.01,1.31)	3,043	10.18 (9.82,10.54)	0.68 (0.60,0.77)	3,845	15.14 (14.66,15.62)	1.01 (0.65,1.56)

Note: CI: confidence interval, REF: Reference group; Major CVD: Major Cardiovascular Disease; Composite outcome of stroke, heart failure and coronary artery disease

Hazard Ratio (HR) and 95% Confidence Interval (95% CI) were estimated by Cox regression, HR >1 (or <1) indicates patients with COVID-19 had higher (lower) risk of sequelae compared to the non-COVID-19 control cohort

Un-vaccinated, Incomplete vaccinated, Fully Vaccinated and Received booster dose of COVID-19 vaccines refers to patients who have received 0 dose, 1 doses, 2 doses and ≥3 doses of BioNtech or CoronaVac COVID-19 vaccines, respectively

Supplementary Table 17. Incidence rate and hazard ratio of clinical sequelae in COVID-19 and non-COVID-19 cohort after weighting during 0-30, 31-90, 91-180, 181-270, 271-365 days of SARS-CoV-2 infection (Male patients; continued)

271-365 days

	Controls (REF)		COVID-19											
			Unvaccinated			Incompletely vaccinated			Fully vaccinated			Received booster dose		
**Clinical sequelae**	**Event**	**Incidence per 1,000 person-years**	**Event**	**Incidence per 1,000 person-years**	**Hazard ratio**	**Event**	**Incidence per 1,000 person-years**	**Hazard ratio**	**Event**	**Incidence per 1,000 person-years**	**Hazard ratio**	**Event**	**Incidence per 1,000 person-years**	**Hazard ratio**
Major CVD	1,230	8.15 (7.70,8.61)	1,260	7.93 (7.51,8.38)	1.05 (0.79,1.39)	1,406	9.69 (9.19,10.21)	1.18 (0.88,1.56)	1,094	7.32 (6.89,7.76)	0.89 (0.75,1.07)	962	6.65 (6.24,7.08)	0.80 (0.62,1.04)
Stroke	590	3.68 (3.39,3.99)	567	3.40 (3.13,3.69)	0.98 (0.65,1.49)	488	3.18 (2.91,3.47)	0.85 (0.53,1.37)	578	3.66 (3.37,3.96)	0.99 (0.76,1.28)	474	3.06 (2.80,3.35)	0.82 (0.56,1.19)
Myocardial infarction	338	2.04 (1.83,2.27)	285	1.63 (1.45,1.83)	0.86 (0.49,1.51)	445	2.80 (2.55,3.07)	1.35 (0.82,2.23)	304	1.87 (1.67,2.09)	0.91 (0.63,1.31)	201	1.26 (1.09,1.44)	0.60 (0.36,1.01)
Heart Failure	300	1.81 (1.61,2.02)	453	2.60 (2.36,2.84)	1.54 (0.99,2.39)	478	3.00 (2.74,3.28)	1.64 (1.02,2.62)	332	2.04 (1.82,2.26)	1.12 (0.77,1.62)	208	1.30 (1.13,1.48)	0.71 (0.30,1.68)
Atrial fibrillation	153	0.93 (0.79,1.08)	225	1.30 (1.14,1.48)	1.45 (0.74,2.84)	201	1.27 (1.10,1.46)	1.37 (0.63,2.95)	166	1.03 (0.88,1.19)	1.10 (0.67,1.79)	127	0.80 (0.67,0.94)	0.85 (0.36,2.03)
Coronary Artery Disease	592	3.75 (3.45,4.06)	512	3.03 (2.78,3.30)	0.85 (0.55,1.31)	628	4.11 (3.79,4.44)	1.09 (0.71,1.67)	478	3.06 (2.79,3.34)	0.81 (0.62,1.07)	493	3.24 (2.97,3.54)	0.86 (0.56,1.30)
Deep vein thrombosis	27	0.16 (0.11,0.24)	58	0.33 (0.25,0.42)	2.20 (0.58,8.34)	0	NA	NA	24	0.15 (0.10,0.22)	0.91 (0.27,3.11)	13	0.08 (0.05,0.14)	0.50 (0.07,3.72)
Cardiovascular mortality	55	0.33 (0.25,0.43)	76	0.43 (0.34,0.53)	1.30 (0.38,4.47)	29	0.18 (0.12,0.25)	0.54 (0.13,2.24)	8	0.05 (0.02,0.09)	0.14 (0.02,1.03)	0	NA	NA
Chronic pulmonary disease	184	1.12 (0.97,1.29)	207	1.21 (1.05,1.38)	1.14 (0.56,2.35)	279	1.78 (1.58,2.00)	1.57 (0.80,3.10)	241	1.50 (1.31,1.69)	1.33 (0.86,2.04)	94	0.60 (0.49,0.73)	0.52 (0.25,1.07)
Acute respiratory distress syndrome	161	0.97 (0.82,1.12)	310	1.77 (1.58,1.97)	1.97 (1.07,3.63)	259	1.62 (1.43,1.83)	1.65 (0.90,3.04)	192	1.17 (1.02,1.35)	1.20 (0.75,1.92)	348	2.17 (1.95,2.41)	2.20 (0.66,7.32)
Seizure	103	0.62 (0.51,0.75)	163	0.94 (0.81,1.09)	1.64 (0.74,3.63)	163	1.03 (0.88,1.20)	1.63 (0.69,3.89)	90	0.55 (0.45,0.68)	0.88 (0.49,1.58)	77	0.47 (0.38,0.59)	0.75 (0.18,3.18)
End-stage renal disease	26	0.16 (0.11,0.23)	114	0.64 (0.53,0.77)	4.52 (1.48,13.83)	22	0.13 (0.09,0.20)	0.84 (0.11,6.47)	4	0.03 (0.01,0.06)	0.17 (0.02,1.33)	42	0.26 (0.19,0.34)	1.60 (0.21,12.34)
Acute kidney injury	91	0.55 (0.44,0.66)	145	0.83 (0.70,0.97)	1.63 (0.76,3.46)	43	0.27 (0.19,0.35)	0.48 (0.11,2.08)	42	0.25 (0.18,0.34)	0.46 (0.20,1.08)	50	0.31 (0.24,0.41)	0.56 (0.20,1.52)
Pancreatitis	27	0.16 (0.11,0.24)	146	0.83 (0.70,0.97)	5.56 (2.20,14.04)	52	0.32 (0.25,0.42)	1.96 (0.37,10.42)	21	0.13 (0.08,0.20)	0.78 (0.23,2.69)	0	NA	NA
All-cause mortality	2,580	15.43 (14.85,16.04)	5,274	29.83 (29.03,30.64)	2.06 (1.80,2.35)	2,737	17.00 (16.37,17.64)	1.09 (0.91,1.30)	1,948	11.82 (11.30,12.35)	0.76 (0.66,0.88)	843	5.20 (4.85,5.56)	0.33 (0.21,0.54)

Note: CI: confidence interval, REF: Reference group; Major CVD: Major Cardiovascular Disease; Composite outcome of stroke, heart failure and coronary artery disease
Hazard Ratio (HR) and 95% Confidence Interval (95% CI) were estimated by Cox regression, HR >1 (or <1) indicates patients with COVID-19 had higher (lower) risk of sequelae compared to the non-COVID-19 control cohort
Un-vaccinated, Incomplete vaccinated, Fully Vaccinated and Received booster dose of COVID-19 vaccines refers to patients who have received 0 dose, 1 doses, 2 doses and ≥3 doses of BioNtech or CoronaVac COVID-19 vaccines, respectively

Supplementary Table 18. Incidence rate and hazard ratio of clinical sequelae in COVID-19 and non-COVID-19 cohort after weighting during 0-30, 31-90, 91-180, 181-270, 271-365 days of SARS-CoV-2 infection (Female patients)

0-30 days

	Controls (REF)		COVID-19												
			Unvaccinated			Incompletely vaccinated			Fully vaccinated			Received booster dose		
**Clinical sequelae**	**Event**	**Incidence per 1,000 person-years**	**Event**	**Incidence per 1,000 person-years**	**Hazard ratio**	**Event**	**Incidence per 1,000 person-years**	**Hazard ratio**	**Event**	**Incidence per 1,000 person-years**	**Hazard ratio**	**Event**	**Incidence per 1,000 person-years**	**Hazard ratio**
Major CVD	572	3.35 (3.08,3.63)	2,710	16.28 (15.68,16.90)	4.84 (3.91,5.99)	2,064	12.32 (11.80,12.86)	3.67 (2.85,4.73)	1,445	8.50 (8.07,8.94)	2.54 (2.07,3.11)	1,143	6.64 (6.26,7.03)	1.98 (1.54,2.55)
Stroke	386	2.20 (1.99,2.42)	1,831	10.68 (10.20,11.18)	4.84 (3.73,6.28)	1,141	6.62 (6.24,7.01)	3.00 (2.16,4.18)	970	5.54 (5.20,5.90)	2.52 (1.96,3.24)	857	4.80 (4.49,5.13)	2.18 (1.60,2.98)
Myocardial infarction	98	0.54 (0.44,0.66)	826	4.65 (4.34,4.98)	8.50 (5.75,12.57)	779	4.38 (4.08,4.69)	8.02 (5.19,12.41)	366	2.03 (1.83,2.25)	3.74 (2.42,5.77)	352	1.92 (1.73,2.13)	3.53 (2.23,5.61)
Heart Failure	150	0.83 (0.71,0.98)	630	3.58 (3.31,3.86)	4.28 (2.88,6.37)	560	3.17 (2.91,3.43)	3.79 (2.35,6.13)	361	2.02 (1.82,2.24)	2.42 (1.59,3.69)	106	0.58 (0.48,0.70)	0.70 (0.33,1.50)
Atrial fibrillation	69	0.38 (0.30,0.48)	381	2.17 (1.96,2.40)	5.67 (3.29,9.78)	308	1.75 (1.57,1.96)	4.57 (2.50,8.38)	210	1.18 (1.02,1.34)	3.08 (1.76,5.37)	285	1.57 (1.40,1.76)	4.10 (2.48,6.78)
Coronary Artery Disease	154	0.87 (0.74,1.01)	869	4.99 (4.66,5.33)	5.72 (3.99,8.19)	850	4.88 (4.56,5.21)	5.60 (3.79,8.29)	421	2.39 (2.17,2.63)	2.75 (1.88,4.04)	399	2.23 (2.02,2.46)	2.57 (1.71,3.86)
Deep vein thrombosis	30	0.16 (0.11,0.23)	167	0.94 (0.81,1.09)	5.75 (2.31,14.34)	189	1.06 (0.92,1.22)	6.50 (2.52,16.78)	74	0.41 (0.33,0.52)	2.53 (1.00,6.40)	41	0.22 (0.16,0.30)	1.37 (0.45,4.23)
Cardiovascular mortality	503	2.77 (2.54,3.02)	2,682	15.04 (14.48,15.62)	5.41 (4.42,6.63)	1,756	9.82 (9.37,10.29)	3.54 (2.74,4.57)	854	4.73 (4.42,5.06)	1.71 (1.28,2.27)	520	2.82 (2.58,3.06)	1.02 (0.63,1.64)
Chronic pulmonary disease	38	0.21 (0.16,0.29)	185	1.06 (0.92,1.22)	4.95 (2.23,11.00)	241	1.37 (1.20,1.55)	6.43 (2.75,15.03)	166	0.93 (0.80,1.08)	4.38 (2.23,8.59)	100	0.55 (0.45,0.67)	2.59 (1.26,5.32)
Acute respiratory distress syndrome	63	0.35 (0.27,0.45)	506	2.86 (2.62,3.11)	8.13 (4.63,14.27)	238	1.34 (1.17,1.51)	3.82 (1.89,7.70)	157	0.87 (0.74,1.01)	2.49 (1.35,4.62)	180	0.98 (0.85,1.13)	2.81 (1.52,5.19)
Seizure	44	0.24 (0.18,0.33)	454	2.58 (2.35,2.82)	10.49 (5.63,19.54)	150	0.84 (0.72,0.99)	3.44 (1.18,10.04)	96	0.53 (0.43,0.65)	2.18 (1.03,4.61)	94	0.51 (0.41,0.62)	2.08 (0.97,4.48)
End-stage renal disease	22	0.12 (0.08,0.18)	148	0.83 (0.71,0.98)	6.92 (1.95,24.58)	45	0.25 (0.18,0.33)	2.08 (0.34,12.72)	38	0.21 (0.15,0.29)	1.76 (0.37,8.38)	86	0.47 (0.38,0.58)	3.90 (0.75,20.30)
Acute kidney injury	44	0.25 (0.18,0.33)	394	2.22 (2.01,2.45)	9.03 (5.05,16.15)	246	1.38 (1.22,1.57)	5.64 (2.83,11.23)	126	0.70 (0.59,0.83)	2.85 (1.30,6.25)	244	1.32 (1.16,1.50)	5.39 (2.70,10.77)
Pancreatitis	34	0.19 (0.13,0.26)	117	0.66 (0.55,0.79)	3.51 (1.34,9.21)	21	0.12 (0.08,0.18)	0.64 (0.09,4.72)	77	0.43 (0.34,0.53)	2.28 (0.99,5.26)	32	0.17 (0.12,0.24)	0.92 (0.31,2.69)
All-cause mortality	2,447	13.48 (12.96,14.02)	43,370	243.23 (240.95,245.53)	17.99 (16.86,19.20)	20,276	113.42 (111.87,114.99)	8.40 (7.71,9.16)	8,821	48.83 (47.82,49.85)	3.62 (3.29,3.99)	8,586	46.52 (45.55,47.51)	3.45 (1.34,8.85)

Note: CI: confidence interval, REF: Reference group; Major CVD: Major Cardiovascular Disease; Composite outcome of stroke, heart failure and coronary artery disease
Hazard Ratio (HR) and 95% Confidence Interval (95% CI) were estimated by Cox regression, HR >1 (or <1) indicates patients with COVID-19 had higher (lower) risk of sequelae compared to the non-COVID-19 control cohort
Un-vaccinated, Incomplete vaccinated, Fully Vaccinated and Received booster dose of COVID-19 vaccines refers to patients who have received 0 dose, 1 doses, 2 doses and ≥3 doses of BioNtech or CoronaVac COVID-19 vaccines, respectively

Supplementary Table 18. Incidence rate and hazard ratio of clinical sequelae in COVID-19 and non-COVID-19 cohort after weighting during 0-30, 31-90, 91-180, 181-270, 271-365 days of SARS-CoV-2 infection (Female patients; continued)

31-90 days

	Controls (REF)		COVID-19											
			Unvaccinated			Incompletely vaccinated			Fully vaccinated			Received booster dose		
**Clinical sequelae**	**Event**	**Incidence per 1,000 person-years**	**Event**	**Incidence per 1,000 person-years**	**Hazard ratio**	**Event**	**Incidence per 1,000 person-years**	**Hazard ratio**	**Event**	**Incidence per 1,000 person-years**	**Hazard ratio**	**Event**	**Incidence per 1,000 person-years**	**Hazard ratio**
Major CVD	1,248	3.72 (3.52,3.93)	2,983	9.17 (8.85,9.50)	2.46 (2.02,3.00)	1,906	5.79 (5.54,6.06)	1.56 (1.21,2.00)	1,431	4.29 (4.07,4.52)	1.15 (0.95,1.39)	1,850	5.50 (5.25,5.76)	1.48 (0.86,2.55)
Stroke	816	2.37 (2.21,2.53)	1,474	4.40 (4.18,4.63)	1.86 (1.41,2.46)	933	2.76 (2.58,2.94)	1.17 (0.81,1.68)	751	2.19 (2.04,2.35)	0.93 (0.72,1.19)	1,194	3.43 (3.24,3.63)	1.45 (0.63,3.33)
Myocardial infarction	206	0.58 (0.50,0.66)	630	1.81 (1.68,1.96)	3.13 (2.12,4.60)	181	0.52 (0.45,0.60)	0.89 (0.40,1.97)	227	0.64 (0.56,0.73)	1.11 (0.68,1.80)	163	0.46 (0.39,0.53)	0.79 (0.41,1.51)
Heart Failure	376	1.07 (0.96,1.18)	1,247	3.62 (3.43,3.83)	3.39 (2.55,4.52)	702	2.02 (1.87,2.17)	1.89 (1.30,2.75)	565	1.61 (1.48,1.75)	1.51 (1.08,2.12)	557	1.57 (1.44,1.70)	1.47 (0.97,2.21)
Atrial fibrillation	241	0.69 (0.60,0.78)	514	1.50 (1.38,1.64)	2.19 (1.46,3.28)	425	1.23 (1.12,1.35)	1.80 (1.07,3.01)	283	0.81 (0.72,0.91)	1.18 (0.75,1.85)	247	0.70 (0.62,0.79)	1.02 (0.63,1.65)
Coronary Artery Disease	335	0.96 (0.86,1.07)	1,019	2.99 (2.81,3.18)	3.10 (2.25,4.28)	509	1.49 (1.36,1.62)	1.54 (0.93,2.55)	493	1.43 (1.30,1.56)	1.48 (1.07,2.04)	452	1.30 (1.18,1.42)	1.34 (0.94,1.91)
Deep vein thrombosis	77	0.22 (0.17,0.27)	160	0.46 (0.39,0.54)	2.12 (0.97,4.60)	181	0.52 (0.44,0.59)	2.37 (1.10,5.09)	123	0.35 (0.29,0.41)	1.60 (0.81,3.17)	46	0.13 (0.10,0.17)	0.60 (0.23,1.55)
Cardiovascular mortality	582	1.63 (1.51,1.77)	1,568	4.49 (4.27,4.72)	2.75 (2.14,3.52)	700	1.99 (1.85,2.14)	1.22 (0.83,1.78)	547	1.55 (1.42,1.68)	0.95 (0.67,1.33)	345	0.96 (0.86,1.07)	0.59 (0.35,1.00)
Chronic pulmonary disease	116	0.33 (0.28,0.40)	154	0.45 (0.38,0.53)	1.36 (0.63,2.93)	220	0.64 (0.56,0.73)	1.92 (0.84,4.43)	171	0.49 (0.42,0.57)	1.49 (0.84,2.64)	74	0.21 (0.16,0.26)	0.63 (0.28,1.42)
Acute respiratory distress syndrome	220	0.62 (0.54,0.71)	425	1.23 (1.11,1.35)	1.97 (1.18,3.31)	403	1.15 (1.04,1.27)	1.86 (1.00,3.46)	234	0.66 (0.58,0.75)	1.07 (0.68,1.69)	284	0.79 (0.71,0.89)	1.28 (0.76,2.15)
Seizure	129	0.37 (0.31,0.43)	394	1.14 (1.03,1.26)	3.12 (1.75,5.57)	300	0.86 (0.77,0.96)	2.36 (1.10,5.07)	130	0.37 (0.31,0.43)	1.01 (0.52,1.94)	99	0.28 (0.23,0.34)	0.76 (0.33,1.73)
End-stage renal disease	34	0.10 (0.07,0.13)	26	0.08 (0.05,0.11)	0.79 (0.24,2.63)	0	NA	NA	41	0.12 (0.08,0.15)	1.20 (0.41,3.53)	7	0.02 (0.01,0.04)	0.20 (0.03,1.50)
Acute kidney injury	101	0.28 (0.23,0.34)	352	1.01 (0.91,1.12)	3.57 (2.11,6.02)	167	0.48 (0.41,0.55)	1.68 (0.74,3.84)	144	0.41 (0.35,0.48)	1.44 (0.72,2.88)	134	0.37 (0.31,0.44)	1.31 (0.59,2.91)
Pancreatitis	74	0.21 (0.16,0.26)	225	0.65 (0.57,0.74)	3.12 (1.46,6.70)	119	0.34 (0.28,0.41)	1.65 (0.55,4.92)	95	0.27 (0.22,0.33)	1.30 (0.60,2.83)	78	0.22 (0.17,0.27)	1.05 (0.48,2.29)
All-cause mortality	3,479	9.78 (9.46,10.11)	12,749	36.53 (35.90,37.17)	3.73 (3.40,4.09)	5,808	16.55 (16.13,16.98)	1.69 (1.48,1.94)	3,900	11.02 (10.68,11.37)	1.13 (0.99,1.28)	3,201	8.91 (8.60,9.22)	0.91 (0.74,1.12)

Note: CI: confidence interval, REF: Reference group; Major CVD: Major Cardiovascular Disease; Composite outcome of stroke, heart failure and coronary artery disease
Hazard Ratio (HR) and 95% Confidence Interval (95% CI) were estimated by Cox regression, HR >1 (or <1) indicates patients with COVID-19 had higher (lower) risk of sequelae compared to the non-COVID-19 control cohort
Un-vaccinated, Incomplete vaccinated, Fully Vaccinated and Received booster dose of COVID-19 vaccines refers to patients who have received 0 dose, 1 doses, 2 doses and ≥3 doses of BioNtech or CoronaVac COVID-19 vaccines, respectively

Supplementary Table 18. Incidence rate and hazard ratio of clinical sequelae in COVID-19 and non-COVID-19 cohort after weighting during 0-30, 31-90, 91-180, 181-270, 271-365 days of SARS-CoV-2 infection (Female patients; continued)

91-180 days

	Controls (REF)		COVID-19											
			Unvaccinated			Incompletely vaccinated			Fully vaccinated			Received booster dose		
**Clinical sequelae**	**Event**	**Incidence per 1,000 person-years**	**Event**	**Incidence per 1,000 person-years**	**Hazard ratio**	**Event**	**Incidence per 1,000 person-years**	**Hazard ratio**	**Event**	**Incidence per 1,000 person-years**	**Hazard ratio**	**Event**	**Incidence per 1,000 person-years**	**Hazard ratio**
Major CVD	1,763	4.09 (3.90,4.28)	2,949	6.54 (6.31,6.78)	1.60 (1.31,1.96)	2,555	5.35 (5.14,5.55)	1.31 (1.04,1.65)	1,808	3.81 (3.63,3.98)	0.93 (0.79,1.10)	1,307	3.59 (3.40,3.79)	0.87 (0.72,1.06)
Stroke	1,134	2.56 (2.41,2.71)	1,415	3.04 (2.89,3.20)	1.19 (0.90,1.58)	1,504	3.05 (2.90,3.21)	1.20 (0.88,1.63)	1,065	2.18 (2.05,2.32)	0.86 (0.69,1.06)	594	1.58 (1.45,1.71)	0.61 (0.47,0.81)
Myocardial infarction	260	0.57 (0.51,0.64)	831	1.72 (1.61,1.84)	3.00 (2.01,4.50)	582	1.15 (1.06,1.24)	2.00 (1.25,3.19)	266	0.53 (0.47,0.60)	0.93 (0.59,1.45)	273	0.71 (0.63,0.80)	1.25 (0.79,1.98)
Heart Failure	440	0.97 (0.88,1.07)	862	1.80 (1.69,1.93)	1.86 (1.33,2.59)	670	1.33 (1.23,1.43)	1.37 (0.92,2.05)	678	1.36 (1.26,1.47)	1.40 (1.05,1.88)	405	1.06 (0.96,1.16)	1.08 (0.73,1.60)
Atrial fibrillation	336	0.74 (0.67,0.83)	598	1.26 (1.16,1.36)	1.69 (1.10,2.60)	445	0.89 (0.81,0.97)	1.20 (0.75,1.92)	414	0.83 (0.76,0.92)	1.12 (0.80,1.58)	372	0.97 (0.88,1.07)	1.30 (0.80,2.11)
Coronary Artery Disease	519	1.16 (1.06,1.26)	1,253	2.65 (2.50,2.80)	2.28 (1.66,3.11)	847	1.70 (1.59,1.82)	1.46 (1.01,2.12)	528	1.08 (0.99,1.17)	0.92 (0.68,1.25)	492	1.31 (1.20,1.43)	1.13 (0.82,1.56)
Deep vein thrombosis	83	0.18 (0.15,0.23)	213	0.44 (0.38,0.50)	2.41 (1.16,5.01)	271	0.53 (0.47,0.60)	2.92 (1.30,6.56)	54	0.11 (0.08,0.14)	0.59 (0.25,1.40)	20	0.05 (0.03,0.08)	0.28 (0.06,1.23)
Cardiovascular mortality	674	1.47 (1.37,1.59)	1,618	3.34 (3.18,3.50)	2.27 (1.76,2.92)	949	1.86 (1.74,1.98)	1.27 (0.91,1.77)	511	1.01 (0.93,1.10)	0.69 (0.49,0.97)	191	0.49 (0.43,0.57)	0.33 (0.18,0.61)
Chronic pulmonary disease	164	0.36 (0.31,0.42)	379	0.80 (0.72,0.88)	2.19 (1.18,4.07)	426	0.85 (0.77,0.93)	2.35 (1.23,4.50)	209	0.42 (0.37,0.48)	1.17 (0.72,1.88)	132	0.35 (0.29,0.41)	0.94 (0.42,2.09)
Acute respiratory distress syndrome	287	0.63 (0.56,0.70)	649	1.35 (1.25,1.45)	2.15 (1.40,3.30)	528	1.04 (0.95,1.13)	1.66 (0.99,2.78)	485	0.97 (0.88,1.06)	1.54 (1.12,2.13)	217	0.56 (0.49,0.64)	0.89 (0.36,2.18)
Seizure	180	0.40 (0.34,0.46)	386	0.81 (0.73,0.89)	2.05 (1.16,3.62)	501	0.99 (0.90,1.08)	2.53 (1.38,4.63)	215	0.43 (0.38,0.49)	1.09 (0.67,1.78)	79	0.20 (0.16,0.25)	0.51 (0.24,1.06)
End-stage renal disease	52	0.11 (0.08,0.15)	54	0.11 (0.08,0.14)	0.98 (0.26,3.64)	11	0.02 (0.01,0.04)	0.19 (0.03,1.42)	23	0.05 (0.03,0.07)	0.41 (0.09,1.79)	0	NA	NA
Acute kidney injury	166	0.36 (0.31,0.42)	287	0.59 (0.53,0.67)	1.66 (0.91,3.02)	121	0.24 (0.20,0.28)	0.67 (0.28,1.59)	192	0.38 (0.33,0.44)	1.07 (0.62,1.85)	74	0.19 (0.15,0.24)	0.51 (0.18,1.48)
Pancreatitis	86	0.19 (0.15,0.23)	98	0.20 (0.16,0.24)	1.08 (0.38,3.06)	37	0.07 (0.05,0.10)	0.39 (0.12,1.29)	91	0.18 (0.15,0.22)	0.96 (0.44,2.09)	31	0.08 (0.06,0.11)	0.43 (0.15,1.21)
All-cause mortality	4,471	9.77 (9.49,10.06)	10,306	21.25 (20.84,21.66)	2.18 (1.97,2.40)	5,830	11.43 (11.14,11.72)	1.17 (1.02,1.34)	3,995	7.94 (7.69,8.19)	0.81 (0.72,0.92)	2,183	5.63 (5.40,5.87)	0.57 (0.45,0.73)

Note: CI: confidence interval, REF: Reference group; Major CVD: Major Cardiovascular Disease; Composite outcome of stroke, heart failure and coronary artery disease
Hazard Ratio (HR) and 95% Confidence Interval (95% CI) were estimated by Cox regression, HR >1 (or <1) indicates patients with COVID-19 had higher (lower) risk of sequelae compared to the non-COVID-19 control cohort
Un-vaccinated, Incomplete vaccinated, Fully Vaccinated and Received booster dose of COVID-19 vaccines refers to patients who have received 0 dose, 1 doses, 2 doses and ≥3 doses of BioNtech or CoronaVac COVID-19 vaccines, respectively

Supplementary Table 18. Incidence rate and hazard ratio of clinical sequelae in COVID-19 and non-COVID-19 cohort after weighting during 0-30, 31-90, 91-180, 181-270, 271-365 days of SARS-CoV-2 infection (Female patients; continued)

181-270 days

	Controls (REF)		COVID-19											
			Unvaccinated			Incompletely vaccinated			Fully vaccinated			Received booster dose		
**Clinical sequelae**	**Event**	**Incidence per 1,000 person-years**	**Event**	**Incidence per 1,000 person-years**	**Hazard ratio**	**Event**	**Incidence per 1,000 person-years**	**Hazard ratio**	**Event**	**Incidence per 1,000 person-years**	**Hazard ratio**	**Event**	**Incidence per 1,000 person-years**	**Hazard ratio**
Major CVD	1,405	4.15 (3.94,4.37)	2,174	6.39 (6.12,6.66)	1.54 (1.24,1.90)	2,122	6.01 (5.76,6.27)	1.45 (1.16,1.80)	1,459	4.18 (3.97,4.40)	1.01 (0.85,1.19)	1,123	1.88 (1.77,1.99)	0.45 (0.23,0.90)
Stroke	885	2.54 (2.38,2.72)	1,293	3.68 (3.49,3.89)	1.45 (1.10,1.91)	1,167	3.21 (3.03,3.40)	1.26 (0.94,1.69)	1,060	2.95 (2.78,3.13)	1.16 (0.78,1.72)	662	1.03 (0.95,1.11)	0.40 (0.22,0.74)
Myocardial infarction	244	0.68 (0.60,0.77)	327	0.90 (0.80,1.00)	1.32 (0.83,2.08)	480	1.28 (1.17,1.40)	1.87 (1.16,3.02)	265	0.72 (0.63,0.81)	1.05 (0.68,1.63)	138	0.22 (0.19,0.26)	0.32 (0.13,0.78)
Heart Failure	381	1.07 (0.97,1.18)	1,086	3.00 (2.83,3.19)	2.81 (2.11,3.74)	738	1.98 (1.84,2.12)	1.85 (1.30,2.62)	360	0.98 (0.88,1.08)	0.92 (0.63,1.34)	661	1.00 (0.93,1.08)	0.94 (0.27,3.27)
Atrial fibrillation	268	0.75 (0.67,0.85)	601	1.67 (1.54,1.81)	2.22 (1.51,3.25)	454	1.22 (1.12,1.34)	1.62 (1.01,2.62)	421	1.15 (1.05,1.27)	1.53 (1.08,2.16)	252	0.38 (0.34,0.43)	0.50 (0.22,1.18)
Coronary Artery Disease	433	1.24 (1.12,1.36)	577	1.61 (1.49,1.75)	1.31 (0.90,1.89)	699	1.90 (1.77,2.05)	1.54 (1.05,2.26)	582	1.61 (1.48,1.75)	1.30 (0.98,1.73)	210	0.34 (0.30,0.39)	0.28 (0.13,0.61)
Deep vein thrombosis	70	0.19 (0.15,0.24)	70	0.19 (0.15,0.24)	0.99 (0.40,2.44)	42	0.11 (0.08,0.15)	0.57 (0.14,2.35)	68	0.18 (0.14,0.23)	0.95 (0.43,2.06)	0	NA	NA
Cardiovascular mortality	449	1.25 (1.14,1.37)	857	2.34 (2.19,2.50)	1.88 (1.38,2.55)	573	1.52 (1.40,1.65)	1.22 (0.84,1.78)	379	1.02 (0.92,1.13)	0.82 (0.52,1.29)	307	0.46 (0.41,0.52)	0.37 (0.13,1.03)
Chronic pulmonary disease	135	0.38 (0.32,0.45)	343	0.95 (0.86,1.06)	2.49 (1.42,4.35)	285	0.77 (0.68,0.86)	2.01 (1.09,3.68)	170	0.47 (0.40,0.54)	1.22 (0.77,1.93)	114	0.21 (0.17,0.25)	0.56 (0.25,1.26)
Acute respiratory distress syndrome	217	0.61 (0.53,0.69)	462	1.27 (1.16,1.39)	2.09 (1.29,3.38)	161	0.43 (0.37,0.50)	0.70 (0.35,1.42)	214	0.58 (0.51,0.66)	0.96 (0.62,1.48)	247	0.37 (0.33,0.42)	0.62 (0.25,1.55)
Seizure	140	0.39 (0.33,0.46)	407	1.12 (1.02,1.24)	2.88 (1.68,4.94)	293	0.78 (0.70,0.88)	2.01 (1.09,3.73)	83	0.22 (0.18,0.28)	0.58 (0.26,1.27)	21	0.03 (0.02,0.05)	0.08 (0.02,0.36)
End-stage renal disease	21	0.06 (0.04,0.09)	58	0.16 (0.12,0.20)	2.70 (0.87,8.34)	61	0.16 (0.13,0.21)	2.74 (0.88,8.50)	43	0.12 (0.08,0.15)	1.96 (0.68,5.67)	0	NA	NA
Acute kidney injury	144	0.40 (0.34,0.47)	221	0.61 (0.53,0.69)	1.51 (0.84,2.70)	185	0.49 (0.43,0.57)	1.22 (0.60,2.50)	122	0.33 (0.27,0.39)	0.82 (0.40,1.67)	19	0.03 (0.02,0.05)	0.07 (0.01,0.56)
Pancreatitis	90	0.25 (0.20,0.31)	183	0.50 (0.43,0.58)	2.01 (0.92,4.38)	48	0.13 (0.09,0.17)	0.51 (0.11,2.32)	90	0.24 (0.20,0.30)	0.97 (0.52,1.82)	47	0.07 (0.05,0.09)	0.28 (0.08,1.06)
All-cause mortality	3,737	10.40 (10.07,10.74)	7,342	20.03 (19.57,20.49)	1.93 (1.73,2.15)	4,463	11.83 (11.49,12.18)	1.14 (0.99,1.31)	2,658	7.17 (6.90,7.45)	0.69 (0.58,0.82)	3,553	5.37 (5.20,5.55)	0.52 (0.24,1.11)

Note: CI: confidence interval, REF: Reference group; Major CVD: Major Cardiovascular Disease; Composite outcome of stroke, heart failure and coronary artery disease
Hazard Ratio (HR) and 95% Confidence Interval (95% CI) were estimated by Cox regression, HR >1 (or <1) indicates patients with COVID-19 had higher (lower) risk of sequelae compared to the non-COVID-19 control cohort
Un-vaccinated, Incomplete vaccinated, Fully Vaccinated and Received booster dose of COVID-19 vaccines refers to patients who have received 0 dose, 1 doses, 2 doses and ≥3 doses of BioNtech or CoronaVac COVID-19 vaccines, respectively

Supplementary Table 18. Incidence rate and hazard ratio of clinical sequelae in COVID-19 and non-COVID-19 cohort after weighting during 0-30, 31-90, 91-180, 181-270, 271-365 days of SARS-CoV-2 infection (Female patients; continued)

271-365 days

	Controls (REF)		COVID-19												
			Unvaccinated			Incompletely vaccinated			Fully vaccinated			Received booster dose			
**Clinical sequelae**	**Event**	**Incidence per 1,000 person-years**	**Event**	**Incidence per 1,000 person-years**	**Hazard ratio**	**Event**	**Incidence per 1,000 person-years**	**Hazard ratio**	**Event**	**Incidence per 1,000 person-years**	**Hazard ratio**	**Event**	**Incidence per 1,000 person-years**	**Hazard ratio**
Major CVD	859	4.39 (4.10,4.69)	1,164	5.67 (5.35,6.00)	1.39 (1.04,1.86)	938	5.02 (4.71,5.35)	1.13 (0.82,1.55)	737	3.83 (3.56,4.11)	0.88 (0.70,1.10)	129,106	55.81 (55.50,56.11)	12.15 (1.37,108.14)
Stroke	508	2.52 (2.31,2.75)	608	2.88 (2.66,3.12)	1.21 (0.81,1.82)	440	2.29 (2.08,2.51)	0.90 (0.56,1.44)	394	1.99 (1.80,2.19)	0.79 (0.58,1.08)	129,044	51.20 (50.92,51.48)	19.49 (2.25,168.59)
Myocardial infarction	171	0.83 (0.71,0.96)	206	0.95 (0.82,1.08)	1.21 (0.64,2.30)	207	1.05 (0.91,1.20)	1.25 (0.65,2.38)	157	0.77 (0.66,0.90)	0.93 (0.54,1.60)	34	0.01 (0.01,0.02)	0.02 (0.00,0.08)
Heart Failure	274	1.33 (1.18,1.49)	617	2.85 (2.63,3.09)	2.31 (1.60,3.33)	457	2.32 (2.11,2.54)	1.72 (1.18,2.51)	329	1.63 (1.46,1.81)	1.22 (0.83,1.80)	129	0.05 (0.04,0.06)	0.04 (0.01,0.13)
Atrial fibrillation	153	0.74 (0.63,0.87)	281	1.31 (1.16,1.47)	1.93 (1.06,3.51)	205	1.05 (0.91,1.20)	1.38 (0.74,2.58)	155	0.77 (0.66,0.90)	1.04 (0.61,1.76)	61,511	23.94 (23.75,24.12)	30.68 (3.53,266.88)
Coronary Artery Disease	272	1.34 (1.19,1.51)	288	1.35 (1.20,1.51)	1.06 (0.61,1.86)	369	1.90 (1.71,2.10)	1.40 (0.84,2.32)	203	1.02 (0.89,1.17)	0.76 (0.48,1.19)	107	0.04 (0.04,0.05)	0.03 (0.01,0.11)
Deep vein thrombosis	29	0.14 (0.10,0.20)	76	0.35 (0.28,0.44)	2.59 (0.89,7.58)	31	0.16 (0.11,0.22)	1.09 (0.26,4.64)	6	0.03 (0.01,0.06)	0.19 (0.03,1.44)	12	0.01 (0.00,0.01)	0.04 (0.00,0.37)
Cardiovascular mortality	40	0.19 (0.14,0.26)	119	0.54 (0.45,0.65)	2.42 (0.96,6.09)	24	0.12 (0.08,0.17)	0.66 (0.09,4.79)	0	NA	NA	0	NA	NA
Chronic pulmonary disease	103	0.50 (0.42,0.61)	171	0.80 (0.68,0.92)	1.71 (0.80,3.63)	188	0.96 (0.83,1.10)	1.88 (0.94,3.75)	100	0.50 (0.41,0.60)	0.98 (0.51,1.87)	14	0.01 (0.00,0.01)	0.02 (0.00,0.14)
Acute respiratory distress syndrome	118	0.57 (0.47,0.68)	62	0.29 (0.22,0.36)	0.54 (0.17,1.67)	236	1.19 (1.05,1.35)	2.06 (1.11,3.82)	112	0.55 (0.45,0.66)	0.96 (0.47,1.95)	55	0.02 (0.02,0.03)	0.04 (0.01,0.20)
Seizure	78	0.38 (0.30,0.47)	107	0.49 (0.40,0.59)	1.37 (0.54,3.48)	152	0.77 (0.66,0.90)	2.04 (0.89,4.67)	53	0.26 (0.20,0.34)	0.70 (0.33,1.46)	33	0.01 (0.01,0.02)	0.03 (0.00,0.32)
End-stage renal disease	14	0.07 (0.04,0.11)	48	0.22 (0.17,0.29)	3.48 (0.92,13.24)	36	0.18 (0.13,0.24)	2.56 (0.56,11.75)	0	NA	NA	0	NA	NA
Acute kidney injury	60	0.29 (0.22,0.37)	36	0.17 (0.12,0.23)	0.62 (0.21,1.86)	49	0.25 (0.19,0.33)	0.85 (0.26,2.76)	57	0.28 (0.21,0.36)	0.96 (0.37,2.46)	975	0.38 (0.36,0.40)	1.28 (0.14,11.34)
Pancreatitis	54	0.26 (0.20,0.33)	46	0.21 (0.16,0.28)	0.87 (0.15,5.09)	64	0.32 (0.25,0.41)	1.23 (0.34,4.40)	13	0.06 (0.04,0.11)	0.25 (0.06,1.09)	23	0.01 (0.01,0.01)	0.03 (0.00,0.29)
All-cause mortality	2,030	9.78 (9.36,10.21)	4,465	20.39 (19.80,21.00)	2.22 (1.93,2.56)	1,666	8.38 (7.99,8.79)	0.85 (0.68,1.05)	1,811	8.88 (8.48,9.30)	0.90 (0.58,1.42)	488	0.19 (0.17,0.21)	0.02 (0.01,0.06)

Note: CI: confidence interval, REF: Reference group; Major CVD: Major Cardiovascular Disease; Composite outcome of stroke, heart failure and coronary artery disease
Hazard Ratio (HR) and 95% Confidence Interval (95% CI) were estimated by Cox regression, HR >1 (or <1) indicates patients with COVID-19 had higher (lower) risk of sequelae compared to the non-COVID-19 control cohort
Un-vaccinated, Incomplete vaccinated, Fully Vaccinated and Received booster dose of COVID-19 vaccines refers to patients who have received 0 dose, 1 doses, 2 doses and ≥3 doses of BioNtech or CoronaVac COVID-19 vaccines, respectively

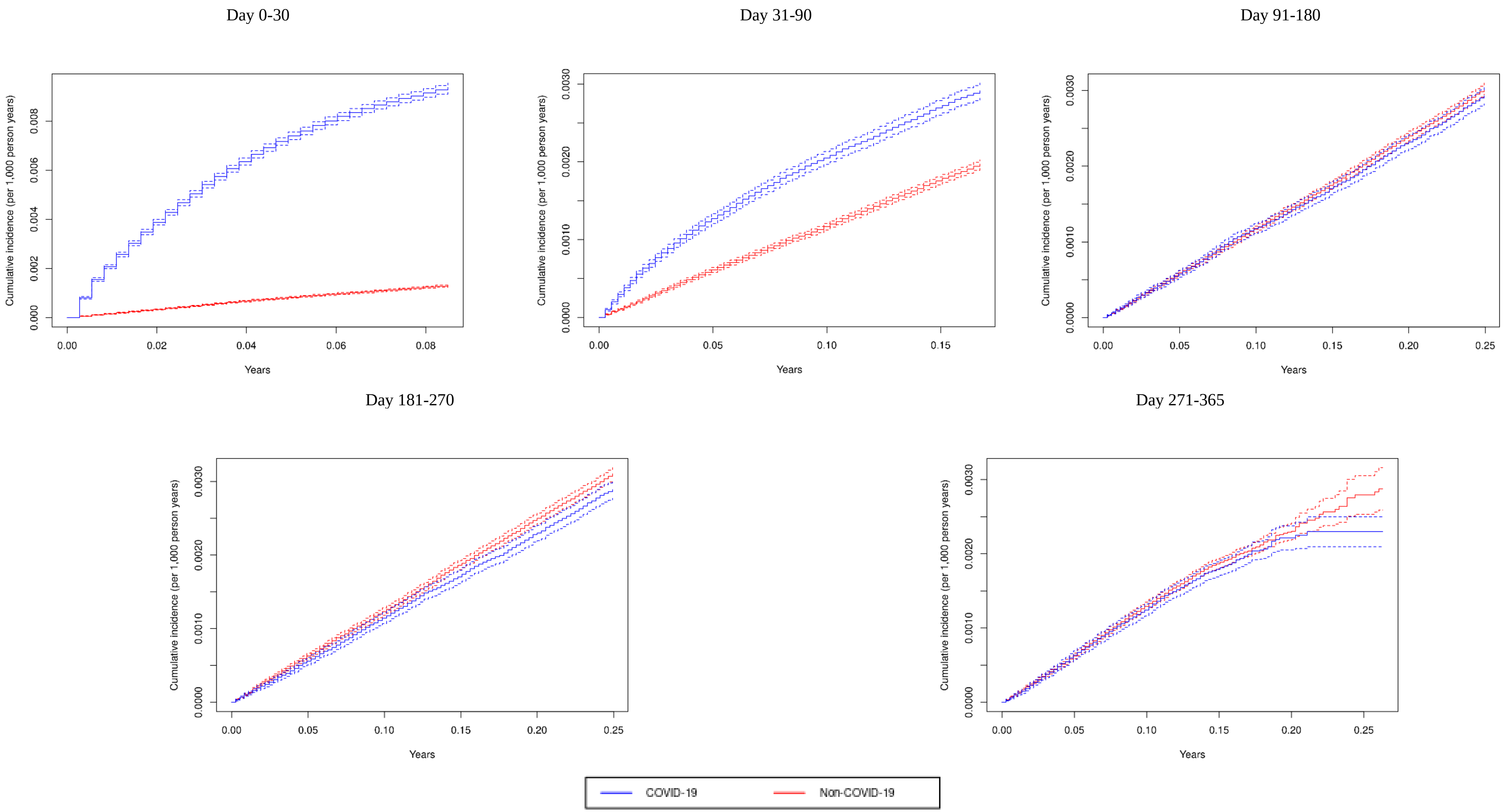

Supplementary Figure 1. Cumulative incidence plot for clinical sequelae during 0-30, 31-90, 91-180, 181-270, 271-365 days of SARS-CoV-2 infection

The number of patients at risk at each observation period between day 0-30, 31-90, 91-180, 181-270, 271-365 were n=2,815,023, n=2,801,396, n=2,764,243, n=2,119,507 and n=1,876,858, respectively.
Dotted lines represented the 95% confidence intervals
