# Supplementary material for: Persistence in risk and effect of COVID-19 vaccination on long-term health consequences after SARS-CoV-2 infection
Source: Nat Commun. 2024 Feb 26;15:1716. doi: 10.1038/s41467-024-45953-1 (PMC10894867; doi:10.1038/s41467-024-45953-1)
Supplement: Supplementary file 3 — Reporting Summary [file 41467_2024_45953_MOESM3_ESM.pdf]

nature portfolio

Corresponding author(s): Ian Chi Kei Wong

Last updated by author(s): Feb 2, 2024

Reporting Summary

Nature Portfolio wishes to improve the reproducibility of the work that we publish. This form provides structure for consistency and transparency in reporting. For further information on Nature Portfolio policies, see our Editorial Policies and the Editorial Policy Checklist.

Statistics

For all statistical analyses, confirm that the following items are present in the figure legend, table legend, main text, or Methods section.

n/a	Confirmed	
☐	☒	The exact sample size (*n*) for each experimental group/condition, given as a discrete number and unit of measurement
☒	☐	A statement on whether measurements were taken from distinct samples or whether the same sample was measured repeatedly
☐	☒	The statistical test(s) used AND whether they are one- or two-sided<br>*Only common tests should be described solely by name; describe more complex techniques in the Methods section.*
☐	☒	A description of all covariates tested
☐	☒	A description of any assumptions or corrections, such as tests of normality and adjustment for multiple comparisons
☐	☒	A full description of the statistical parameters including central tendency (e.g. means) or other basic estimates (e.g. regression coefficient) AND variation (e.g. standard deviation) or associated estimates of uncertainty (e.g. confidence intervals)
☐	☒	For null hypothesis testing, the test statistic (e.g. *F*, *t*, *r*) with confidence intervals, effect sizes, degrees of freedom and *P* value noted<br>*Give P values as exact values whenever suitable.*
☒	☐	For Bayesian analysis, information on the choice of priors and Markov chain Monte Carlo settings
☐	☒	For hierarchical and complex designs, identification of the appropriate level for tests and full reporting of outcomes
☒	☐	Estimates of effect sizes (e.g. Cohen's *d*, Pearson's *r*), indicating how they were calculated

Our web collection on statistics for biologists contains articles on many of the points above.

Software and code

Policy information about availability of computer code

Data collection	No specific software was used in the collection of data.
Data analysis	All statistical analyses were performed using R version 4·1·2 (R Foundation for Statistical Computing, Vienna, Austria). The code used in this study is available on Zenodo (https://doi.org/10.5281/zenodo.10132693).

For manuscripts utilizing custom algorithms or software that are central to the research but not yet described in published literature, software must be made available to editors and reviewers. We strongly encourage code deposition in a community repository (e.g. GitHub). See the Nature Portfolio guidelines for submitting code & software for further information.

Data

Policy information about availability of data

All manuscripts must include a data availability statement. This statement should provide the following information, where applicable:

- Accession codes, unique identifiers, or web links for publicly available datasets
- A description of any restrictions on data availability
- For clinical datasets or third party data, please ensure that the statement adheres to our policy

The data contains confidential information and hence cannot be shared with the public due to third-party use restrictions. Local academic institutions, government departments, or non-governmental organizations may apply for the access to data through the Hospital Authority's data sharing portal (https://www3.ha.org.hk/data).

Research involving human participants, their data, or biological material

Policy information about studies with human participants or human data. See also policy information about sex, gender (identity/presentation), and sexual orientation and race, ethnicity and racism.

Reporting on sex and gender	This study reported sub-group analyses on participants of male and female. The sex of each participants was determined based on electronic health record from the Hong Kong Hospital Authority (HKHA).
Reporting on race, ethnicity, or other socially relevant groupings	This manuscript did not report on race, ethnicity, or other socially relevant groupings of participants
Population characteristics	Patients with an incident SARS-CoV-2 infection (confirmed by rapid antigen test [RAT] or polymerase chain reaction [PCR] test in throat swab, nasopharyngeal aspirate, or deep throat sputum specimens) between April 1st, 2020 and October 31st, 2022 were matched to non-infected controls without a positive SARS-CoV-2 test record throughout the study period with the exact birth-year and sex. Patients with SARS-CoV-2 infection were further stratified into (1) unvaccinated (0 dose), (2) incompletely vaccinated (1 dose), (3) completely (2 doses), and (4) vaccinated with booster doses (≥3 doses) according to the number of BioNtech or CoronaVac vaccines received prior to first SARS-CoV-2 infection. We provide detailed descriptive information on these study cohorts in Table 1a and Table 1b.
Recruitment	In-patients electronic medical records were retrieved from the HKHA
Ethics oversight	Ethical approval for this study was granted by the Institutional Review Board of the University of HK/HA HK West Cluster (UW20-556 and UW21-149) and Department of Health, HK (L/M21/2021 and L/M175/2022) with an exemption for informed consent from participants as patients' confidentiality was maintained in this retrospective cohort study.

Note that full information on the approval of the study protocol must also be provided in the manuscript.

Field-specific reporting

Please select the one below that is the best fit for your research. If you are not sure, read the appropriate sections before making your selection.

☒ Life sciences ☐ Behavioural & social sciences ☐ Ecological, evolutionary & environmental sciences

For a reference copy of the document with all sections, see nature.com/documents/nr-reporting-summary-flat.pdf

Life sciences study design

All studies must disclose on these points even when the disclosure is negative.

Sample size	Sample size calculation was not conducted. This is a population-based study and all identified eligible patients from the territory-wide healthcare database were included in the analysis. A total of 1,175,277 patients with COVID-19 and 2,649,116 matched non-COVID-19 controls were included in this study.
Data exclusions	Part of the sample was excluded during the age- and sex-matching procedure. Individuals aged under 18, who died before the index date and with an incomplete COVID-19 vaccination record were excluded.
Replication	At least two investigators (ICHL, RZ, and EYFW) conducted each of the statistical analyses independently for quality assurance. All attempts at replication were successful.
Randomization	An observational study was conducted, hence exposure allocation was not random. Inverse Probability Treatment Weighting (IPTW) was applied to account for potential confounding factors including age, gender and Charlson Comorbidity index (CCI), history of medication and the number of hospital admission and doctor consultation within one year of index date between study cohorts
Blinding	An observational study was conducted, hence blinding was not possible

Reporting for specific materials, systems and methods

We require information from authors about some types of materials, experimental systems and methods used in many studies. Here, indicate whether each material, system or method listed is relevant to your study. If you are not sure if a list item applies to your research, read the appropriate section before selecting a response.

Materials & experimental systems

n/a	Involved in the study
☒	☐ Antibodies
☒	☐ Eukaryotic cell lines
☒	☐ Palaeontology and archaeology
☒	☐ Animals and other organisms
☒	☐ Clinical data
☒	☐ Dual use research of concern
☒	☐ Plants

Methods

n/a	Involved in the study
☒	☐ ChIP-seq
☒	☐ Flow cytometry
☒	☐ MRI-based neuroimaging
